# Different doses of dual orexin receptor antagonists in primary insomnia: a Bayesian network analysis

**DOI:** 10.3389/fphar.2023.1175372

**Published:** 2023-05-16

**Authors:** Tao Xue, Xin Wu, Jiaxuan Li, Shujun Chen, Zilan Wang, Xin Tan, Zhong Wang, Jianguo Zhang

**Affiliations:** ^1^ Department of Neurosurgery, Beijing Tiantan Hospital, Capital Medical University, Beijing, China; ^2^ Department of Neurosurgery & Brain and Nerve Research Laboratory, The First Affiliated Hospital of Soochow University, Suzhou, Jiangsu Province, China; ^3^ Department of Neurosurgery, Suzhou Ninth People’s Hospital, Suzhou, China; ^4^ Department of Neurology, Beijing Tiantan Hospital, Capital Medical University, Beijing, China; ^5^ Department of Neurology, Suzhou Municipal Hospital, The Affiliated Suzhou Hospital of Nanjing Medical University, Suzhou, Jiangsu Province, China; ^6^ Department of Neurosurgery, Beijing Neurosurgical Institute, Beijing, China; ^7^ Beijing Key Laboratory of Neurostimulation, Beijing, China

**Keywords:** suvorexant, lemborexant, daridorexant, network meta-analysis, DORA, dual orexin receptor antagonist, GRADE, grading of recommendations, assessment, development, and evaluation, insomnia

## Abstract

**Background:** Systematic comparisons of the doses of the Food and Drug Administration (FDA)-approved dual orexin receptor antagonists (DORAs) for people with insomnia are limited.

**Methods:** PubMed, Embase, Cochrane Library, and Clinicaltrials. gov were systematically searched to identify relevant studies published before 31 October 2022. We assessed the certainty of evidence using the confidence in network meta-analysis (CINeMA) framework.

**Results:** We pooled 7257 participants from 9 randomized controlled trials (RCTs). Moderate to high certainty evidence demonstrated suvorexant (20 and 40 mg) and daridorexant (10 and 50 mg) as the most effective in latency to persistent sleep (LPS) reduction. Lemborexant at 5 and 10 mg was the most effective in subjective sleep onset time (sTSO) reduction. For wake time after sleep onset (WASO), all drugs except daridorexant 5 mg were more effective than placebo. Lemborexant 5 mg was among the best in subjective WASO (sWASO) (moderate to high certainty) and had the highest surface under the curve ranking area (SUCRA) values for sWASO (100%). For total sleep time (TST), suvorexant and daridorexant, except the respective minimum doses, were more effective than placebo, while suvorexant 40 mg and lemborexant 10 mg may have been the most effective for subjective TST (sTST) (low to very low certainty). Suvorexant 40 mg (RR 1.09), suvorexant 80 mg (RR 1.65), and daridorexant 25 mg (RR 1.16) showed a higher safety risk than placebo.

**Conclusion:** Suvorexant 20 mg, lemborexant 5 mg, lemborexant 10 mg, and daridorexant 50 mg represent suitable approaches for insomnia.

**Clinical Trial Registration:** clinicaltrials.gov, PROSPERO (CRD42022362655).

## 1 Introduction

Insomnia is a common psychological and physiological disease. It is defined as an impairment of sleep continuity associated with difficulty in initiating sleep, having more frequent awakenings during sleep, or waking up earlier than intended (Sutton, 2021; Perlis et al., 2022). According to various criteria, the prevalence of insomnia ranges from 3.9% to 23.6% (Roth et al., 2011), and in primary care patients, the rate can reach 50% (Perlis et al., 2021). The most significant risk factor for insomnia is age, with up to 50% of older people complaining about difficulty initiating or maintaining sleep (Foley et al., 2004; Crowley, 2011). Insomnia disorder is a considerable risk factor for gastroesophageal reflux disease, cardiovascular disease, type 2 diabetes, hypertension, anxiety, and depression (Barbar et al., 2000; Javaheri and Redline, 2017; Bollu and Kaur, 2019). In addition, inadequate sleep is associated with increased mortality among elderly adults (Crowley, 2011). Insomnia is a major public issue and imposes a significant economic burden on the healthcare system (Morin and Jarrin, 2022).

According to the American College of Physicians (ACP) recommendation, insomnia can be treated with pharmacologic therapy, nonpharmacologic therapy, or a combination of both (Qaseem et al., 2016). Nonpharmacologic therapy consists of cognitive behavioral therapy for insomnia (CBT-I), multicomponent behavioral therapy, brief behavioral therapy (BBT), and interventions such as relaxation strategies, sleep restriction, and stimulus control (Qaseem et al., 2016). Current insomnia pharmacologic therapy can be classified as gamma-aminobutyric acid (GABA) modulators (including benzodiazepines and benzodiazepine receptor agonists), melatonin agonists, sedative antidepressants, and orexin receptor antagonists (ORAs) (Bollu and Kaur, 2019; Perlis et al., 2022). The orexin signaling system includes orexin A and B, as well as its G-protein-coupled receptors (GPCRs), orexin receptor 1 (OX1R), and orexin receptor 2 (OX2R) (Yin et al., 2015). The combination of orexins and their corresponding receptors can regulate sleep; therefore, inhibiting orexin receptors is an essential therapy for insomnia (Yin et al., 2015). Dual orexin receptor antagonists (DORAs) inhibit the wakefulness driven by the orexin system and promote sleep through a competitive inhibitory effect with OX1R and OX2R (Sun et al., 2021).

Currently, six DORAs have been included in clinical trials for insomnia: suvorexant (MK-4305), lemborexant (E2006), daridorexant (ACT-541468), almorexant (ACT-078573), filorexant (MK-6096), and TS-142. Suvorexant, lemborexant, and daridorexant have been approved by the Food and Drug Administration (FDA) for the treatment of primary insomnia (Yang, 2014; Scott, 2020; Markham, 2022). Due to safety issues, the clinical development of almorexant was suspended in 2011, and the drug is now mainly used in animal studies (Roecker et al., 2016; Wu et al., 2022). Although filorexant had some positive effects in the treatment of insomnia, only one randomized controlled trial (RCT) was published in recent years (Wu et al., 2022). TS-142 is a new DORA, with only one Phase 2 trial with a small sample size (Uchiyama et al., 2022). Although our previous studies have compared the safety and efficacy of DORAs in the treatment of insomnia (Xue et al., 2022), evidence about the dosage of DORAs is limited to date (Perlis et al., 2022). We included both approved and non-approved doses since some unconventional doses in clinical treatment may be used in patients who do not respond to conventional doses. Therefore, the purpose of our study is to provide additional evidence for the clinical treatment of these patients. We pooled data from previous RCTs and conducted a systematic review and network meta-analysis (NMA) to investigate the efficacy and safety of different doses of FDA-approved DORAs for the treatment of primary insomnia.

## 2 Methods

### 2.1 Study protocol

We initially drafted a research protocol according to the Cochrane collaboration format before the project started (Liberati et al., 2009). The protocol for this systematic review was prospectively registered in PROSPERO (CRD42022362655).

### 2.2 Eligibility criteria

The inclusion criteria were set as follows: 1) study type: RCT; 2) language restriction: only in English; 3) participants: adult patients diagnosed with primary insomnia according to the Diagnostic and Statistical Manual of Mental Disorders, IVth edition, text revision (DSM-IV-TR) or Diagnostic and Statistical Manual of Mental Disorders, Vth edition (DSM-V) criteria; 4) intervention: FDA-approved DORAs including suvorexant, lemborexant and daridorexant; 5) control: placebo; and 6) outcomes: outcomes including in this meta-analysis were objective and subjective sleep maintenance and onset outcomes, patients-evaluated outcomes, adverse effects (AEs), and serious adverse effects (SAEs). Objective sleep related outcomes were measured by polysomnography (PSG), which included latency to persistent sleep (LPS), wake time after sleep onset (WASO), and total sleep time (TST). Subjective sleep related outcomes were measured by patient-reported electronic morning sleep diaries, which included subjective time to sleep onset (sTSO), subjective WASO (sWASO) and subjective TST (sTST). The patients-evaluated outcome was the insomnia severity index (ISI) score, which is a reliable and valid instrument to quantify perceived insomnia severity, with a higher score representing a more serious degree of insomnia (Bastien et al., 2001). The included RCTs were not required to supply all the outcomes mentioned above.

The exclusion criteria were set as follows: 1) study type: retrospective and cohort studies, reviews, conferences, protocols and case reports; 2) participants: insomnia patients with specific physical and psychiatric comorbidities such as Alzheimer’s disease, trauma-related insomnia and major depressive disorder; 3) intervention: DORAs not approved by the FDA, such as almorexant, filorexant, TS-142 and selective orexin receptor antagonists (SORAs), such as seltorexant (JNJ-42847922); and 4) control: active control.

### 2.3 Search strategy

To find pertinent papers published until 31 October 2022, two independent researchers (TX and XW) conducted a thorough search of PubMed, Embase, the Cochrane Library, and Clinicaltrials.gov. The electronic Supplementary Material S1 contains a complete description of the search approach (Supplementary Table S1). To guarantee a more thorough search, the reference lists of RCTs, pertinent systematic reviews, and meta-analyses were also independently and manually examined.

### 2.4 Study selection and data collection

Two authors (TX and XW) independently reviewed all records searched from the electronic database, including the reference lists of RCTs and relevant systematic reviews or meta-analyses, based on the eligibility criteria described above. Duplicates and research articles with only abstracts were eliminated. Disagreements between the two writers were resolved by discussion or, if necessary, by a third author (JXL) who was not involved in data gathering. We thoroughly examined and vetted the papers included in the full-text screening according to the inclusion and exclusion criteria established above. The articles excluded in the full-text screening stage are shown in Supplementary Table S2. Following selection and evaluation, the following information was retrieved from the included RCTs: basic information, specific outcome events for each RCT, inclusion and exclusion criteria, and study design. The online Supplemental Material S1 display all efficacy and safety outcomes (Supplementary Table S3).

### 2.5 Risk of bias and quality of evidence

Review Manager 5.3 was used to assess a risk of bias plot. The risk of bias for RCTs was evaluated using the standard Cochrane collaboration criteria (Higgins et al., 2011), which took into account selection bias, performance bias, detection bias, attrition bias, reporting bias, and other potential biases. Each bias criterion was assigned one of three levels: "low," "high," or "unclear." TX and XW conducted the evaluation individually. An independent third author was consulted to resolve disagreements (JXL). We also checked for publication bias by determining whether the funnel plots were symmetric.

Using the guidelines provided by the Grading of Recommendations, Assessment, Development, and Evaluation (i.e., "GRADE") Working Group, the degree of certainty of both direct and indirect evidence of NMAs was evaluated using the Confidence in Network Meta-Analysis framework (CINeMA) (Atkins et al., 2004; Nikolakopoulou et al., 2020). TX and XW independently rated the overall quality of evidence as high, moderate, low, or very low based on an assessment of the overall risk of bias (randomization, blinding, allocation concealment, selective reporting), imprecision (95% confidence interval and sample size), inconsistency and indirectness (study population), and risk of publication bias (funding sources). Assist from a third author also helped to settle disagreements (JXL).

### 2.6 Summary measures and synthesis of results

NMA was performed by using the “gemtc” package in R 3.5.2 software within a Bayesian framework. The Markov chain Monte Carlo methods involved four chains with overdispersed initial values and Gibbs sampling based on 50,000 iterations after a burn-in phase of 20,000 iterations. The convergence of the model was evaluated by the track and density plot and the Brooks-Gelman-Rubin diagnosis plot. Fluctuation could not be recognized; the density graph was normally distributed and all potential scale reduction factor values of the various parameters that were restricted to 1 indicate an excellent convergence. We employed the best-matched model according to the deviance information criteria (DIC), which reflected the goodness-of-fit of the network results. Specifically, we chose the model with a smaller DIC between the fixed-effect model and the random-effect model, which means that it could better fit the results of the network model (Supplementary Table S4). We estimated the summary mean differences (MDs) for continuous outcomes and risk ratio (RR) for dichotomous outcomes with their 95% credible intervals (CrIs) (CrI for the Bayesian framework and confidence interval [CI] for the frequentist setting). To rank the performance of different dosages of FDA-approved DORAs and placebo in each efficacy and safety outcome, we created the surface under the curve ranking area (SUCRA). A higher SUCRA rating suggested that the intervention had performed better. Each intervention was ranked, and the ranking probabilities were computed as cumulative probabilities. We presented the findings from the NMA using league tables and Vitruvian plots. Vitruvian plots use radial bar visualization tools that synthesize the results of multiple outcomes.

We evaluated the transitivity assumption by comparing the distribution of key study characteristics across studies grouped by comparison. As NMA is based on the consistency between direct and indirect evidence, we assessed global inconsistency by comparing the DIC of the employed consistent model (fixed/random-effect model) with the corresponding inconsistent model (unrelated means model), and a difference in DIC less than 11 indicated good global consistency (Spiegelhalter et al., 2002). In addition, we used the node-splitting model to assess the differences between direct and indirect comparisons to determine the local consistency of the networks (Dias et al., 2010).

We assessed the characteristics of the included studies and discovered significant differences in their duration of follow-up, age of the participants, and study design. The time of follow-up for included studies ranged from 2 days to 12 months, with four studies with a follow-up period greater than or equal to 3 months and five studies with a follow-up period less than or equal to 1 month. The majority of the studies included participants over the age of 18, and some only included elderly individuals. The study designs included crossover RCTs and parallel-group RCTs, and only two of the nine studies were crossover RCTs. Thus, we evaluated the possible heterogeneity of treatment effects and the robustness of our findings with subgroup network meta-analyses using time of follow-up (≤1 month and ≥3 months), study design (parallel-group trials), and age (elderly individuals [≥55 for females and ≥65 for males]) as covariates. We performed sensitivity analyses including only trials at an overall low risk of bias and compared these results with the primary analysis.

The GRADE approach was also used to determine the magnitude of efficacy and safety based on the minimally contextualized framework (Brignardello-Petersen et al., 2020). For details, please see the Supplementary Materials S1.

## 3 Results

**FIGURE 1:**
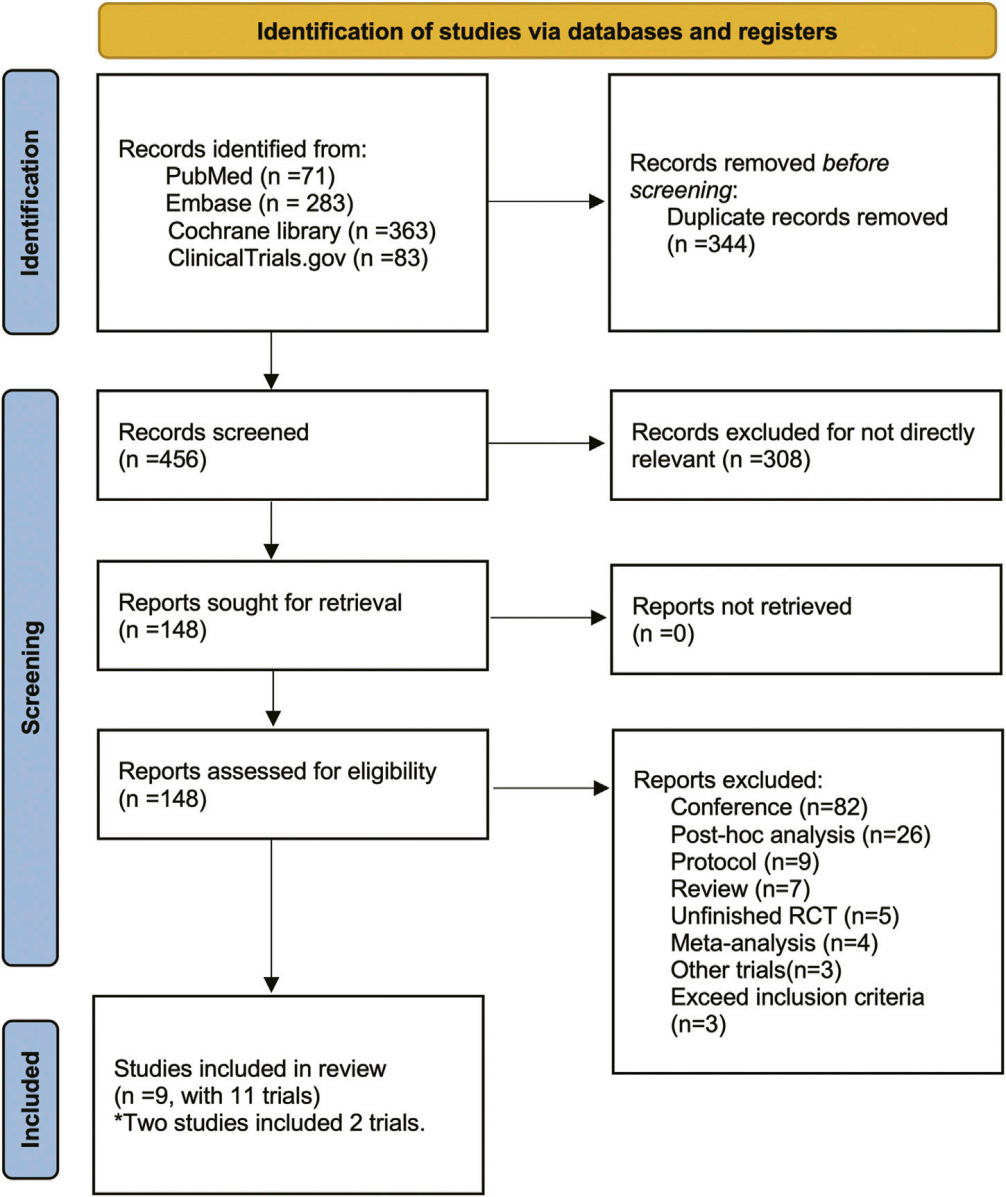
The study search, selection, and inclusion process.

**FIGURE 2:**
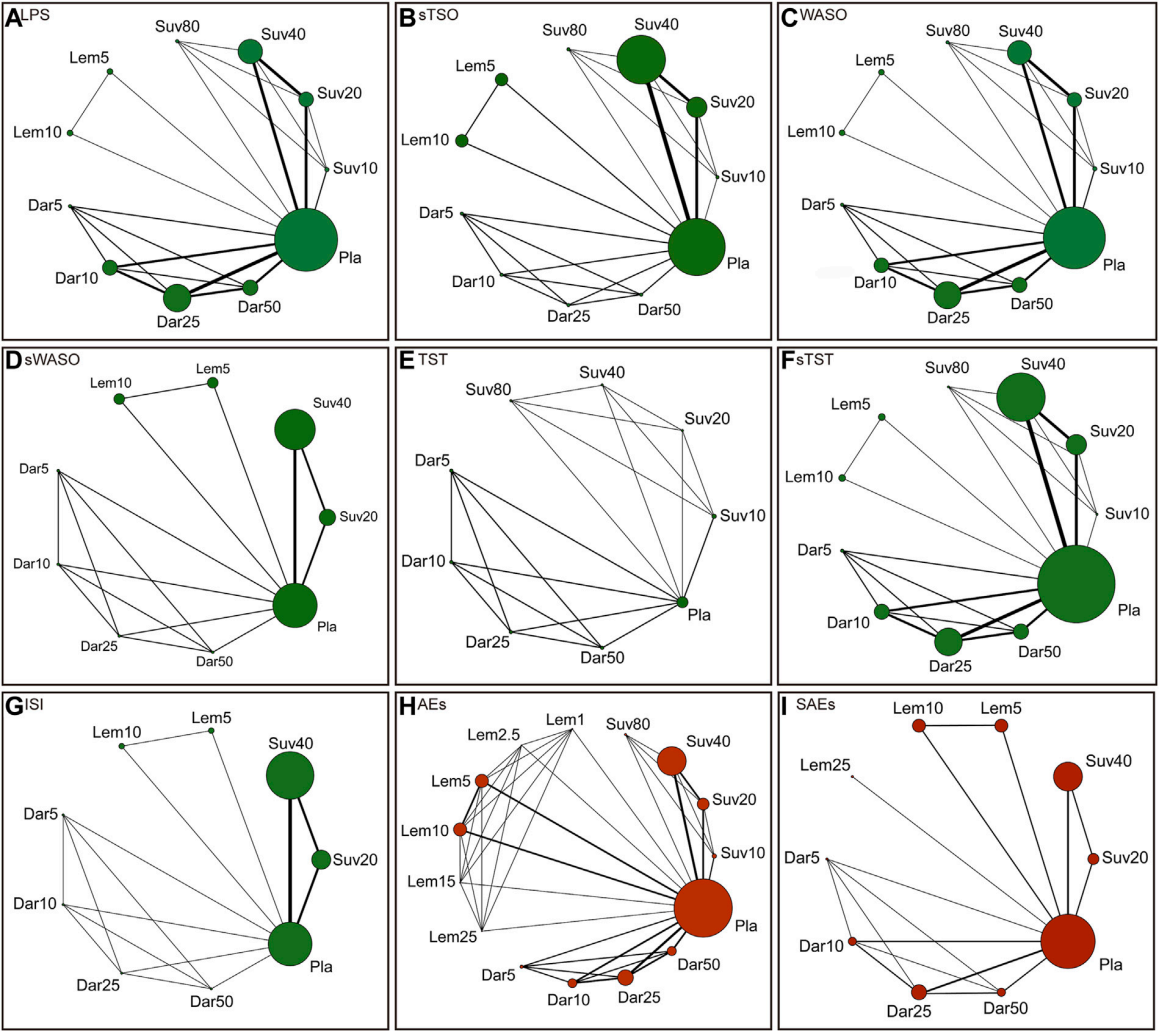
Network of randomized controlled trials (RCTs) comparing different approved dual orexin receptor antagonists (DORAs) of insomnia treatments. The size of circles represented the number of participants for each intervention and the width of lines represented the number of trials compared between treatments. Green colors represent efficacy outcomes and red colors represent safety outcomes. **(A)** latency to persistent sleep (LPS). **(B)** subjective time to sleep onset (sTSO). **(C)** wake time after sleep onset (WASO). **(D)** subjective wake time after sleep onset (sWASO). **(E)** total sleep time (TST). **(F)** subjective total sleep time (sTST). **(G)** insomnia severity index (ISI). **(H)** adverse effects (AEs). **(I)** serious adverse effects (SAEs).

There were 800 titles and abstracts altogether from PubMed, Embase, Cochrane Library, and Clinicaltrials.gov. Following a short examination, 652 articles were eliminated due to duplication and/or relevance, and 148 full-text articles had their eligibility evaluated. Due to participants or publication kinds that were not eligible, 139 of them, including the following articles, were excluded: 82 conference abstracts, 26 *post hoc* analyses, nine protocols, seven reviews, five unfinished RCTs, four meta-analyses, three non-RCTs and three RCTs that did not meet our inclusion criteria. The selection process was summarized in the flow diagram showed in Figure 1. The details of study exclusion are shown in Supplementary Table S1. Finally, a total of nine studies containing 11 RCTs were included in the present NMA. The main characteristics of the nine included studies are listed in Table 1. The network relationships between the various interventions are shown in Figure 2. The size of each circle represents the number of participants for each intervention, and the width of each line represents the number of trials compared between treatments.

**TABLE 1:** Characteristics of the included studies and outcome events.

Study	N	Countries	Centers	Design	Age range	Treatment group, (No. Of participants)	Male (%)	Mean age ±SD (years)	Race (%)	Study period	Outcome events
Herring et al. (NCT00792298)	254	USA and Japan	41	Double-blind placebo-controlled crossover RCT	18–64y	SUV 10 mg (62)	45.2	45.1 ± 11.5	White 71.0	4 weeks	LPS, sTSO, WASO, TST, sTST, AE, SAE
									Asian 17.7		
									Black 11.3		
						SUV 20 mg (61)	34.4	44.3 ± 11.1	White 63.9		
									Asian 18.0		
									Black 18.0		
						SUV 40 mg (59)	45.8	44.5 ± 11.3	White 71.2		
									Asian 16.9		
									Black 10.2		
									Multiracial 1.7		
						SUV 80 mg (61)	44.3	43.8 ± 12.1	White 73.8		
									Asian 16.4		
									Black 8.2		
									Multiracial 1.6		
						PLA (249)	41	44.3 ± 11.5	White70.3		
									Asian 17.3		
									Black 11.6		
									Multiracial 0.8		
Michelson et al. (NCT01021813)	779	USA, Australia, Europe, and South Africa	106	Double-blind placebo-controlled parallel-group RCT	18–65 and ≥65y	SUV 40/30 mg (521)	45	61.3 ± 14.5	White 91	12 months	sTSO, sWASO, sTST, ISI, AE, SAE
									Black 6		
									Other: 2		
						PLA (258)	42	62.0 ± 14.6	White 90		
									Black 9		
									Other: 1		
Herring et al.	1021	USA, Europe, Asia, and South Africa	79	Double-blind placebo-controlled parallel-group RCT	18–65y and ≥65y	SUV 20/15 mg (254)	36.2	55 ± 16	White 66.1	3 months	LPS, sTSO, WASO, sWASO, sTST, ISI, AE, SAE
									Black 5.9		
									Asian 26.0		
									Other 2.0		
						SUV 40/30 mg (383)	39.9	56 ± 15	White 66.1		
									Black 4.7		
Trial 1 (NCT01097616)									Asian 25.6		
									Other 3.7		
						PLA (384)	36.2	56 ± 15	White 63.5		
									Black 6.5		
									Asian 25.8		
									Other 4.2		
Herring et al.	1009	USA, Australia, Europe, Asia, and South Africa	91	Double-blind placebo-controlled parallel-group RCT	18–65y and ≥65y	SUV 20/15 mg (239)	34.3	56 ± 16	White 79.5	3 months	LPS, sTSO, WASO, sWASO, sTST, ISI, AE, SAE
									Black 1.7		
									Asian 11.3		
									Other: 7.5		
						SUV 40/30 mg (387)	31.0	57 ± 15	White 80.1		
									Black 5.2		
Trial 2 (NCT01097629)									Asian 6.7		
									Other: 8.0		
						PLA (383)	35.5	57 ± 15	White 80.7		
									Black 5.5		
									Asian 6.5		
									Other:7.3		
Murphy et al. (NCT01995838)	291	USA	22	Bayesian adaptive double-blind placebo-controlled parallel-group RCT	19–80 y	LEM 1 mg (32)	28.1	53.3 ± 13	White 78.1	1 month	AE, SAE
									Black or African American 21.9		
						LEM 2.5 mg (27)	37	49.7 ± 14.3	White 77.8		
									Black or African American 18.5		
									Other 3.7		
						LEM 5 mg (38)	39.5	51.1 ± 14.3	White 84.5		
									Black or African American 7.9		
									American Indian/Alaskan Native 2.6		
									Other 5.3		
						LEM 10 mg (32)	37.5	47.1 ± 13.7	White 65.6		
									Black or African American 21.9		
									American Indian/Alaskan Native 3.1		
									Other 9.4		
						LEM 15 mg (56)	42.9	44 ± 14.6	White 69.6		
									Black or African American 26.8		
									Other 3.6		
						LEM 25 mg (50)	22.0	48.9 ± 13.4	White 78.0		
									Black or African American 16.0		
									American Indian/Alaskan Native 2.0		
									Other 4.0		
						PLA (56)	35.7	47.1 ± 15.6	White 69.6		
									Black or African American 26.8		
									Other 3.6		
Rosenberg et al. (SUNRISE 1)	1006^a^	North America and Europe	67	Double-blind placebo-controlled active-comparator parallel-group RCT	Women ≥55 y and men ≥65 ys	LEM 5 mg (266)	13.9	63.7 ± 6.8	White 78.4	1 month	LPS, sTSO, WASO, sWASO, ISI, AE, SAE
									Black 23.7		
									Other Asian 0.8		
									Other 0.8		
						LEM 10 mg (269)	14.5	64.2 ± 6.9	White 75.1		
									Black 23.0		
									Chinese 0.4		
(NCT02783729)									Other Asian 1.5		
						PLA (208)	11.5	63.9 ± 6.8	White 73.6		
									Black 24.5		
									Japanese 0.5		
									Chinese 0.5		
									Other 1.0		
Dauvilliers et al. (NCT02839200)	359^b^	Germany, Hungary, Israel, Spain, Sweden, and the USA	38	Double-blind placebo-controlled active-controlled parallel-group RCT	18–64 y	DAR 5 mg (60)	37	42.4 ± 11.4	Caucasian 90	4 weeks	LPS, sTSO, WASO, sWASO, TST, sTST, ISI, AE, SAE
									Black or African American 8		
									Other 2		
						DAR 10 mg (58)	34	45.2 ± 10.9	Caucasian 84		
									Black or African American 14		
									Native Hawaiian/Other Pacific Islander 2		
						DAR 25 mg (60)	35	46.4 ± 11.9	Caucasian 93		
									Black or African American 7		
						DAR 50 mg (61)	36	45.0 ± 11.5	Caucasian 92		
									Black or African American 8		
						PLA: (60)	37	45.7 ± 10.4	Caucasian 87		
									Black or African American 12		
									Asian 2		
Karppa et al. (SUNRISE 2) (NCT02952820)	949	North America, Europe, Asia, and Oceania	119	Double-blind placebo-controlled parallel-group RCT	≥18y	LEM 5 mg (316)	33.9	54.2 ± 13.7	White 70.3	6 months	sTSO, sWASO, sTST, AE, SAE
									Black or African American 8.5		
									Japanese 16.8		
									Other 4.4		
						LEM 10 mg (315)	29.5	54.8 ± 13.7	White 71.4		
									Black or African American 8.3		
									Japanese 17.1		
									Other 3.2		
						PLA (318)	32.1	54.5 ± 14.0	White 73		
									Black or African American 7.2		
									Japanese 17.0		
									Other 2.8		
Zammit et al. (NCT02841709)	58	Germany and USA	10	Double-blind placebo-controlled crossover RCT	≥65 y	DAR 5 mg (56)	Male of all the patients: 33	Mean age of all the patients: 69	White 93	2 days	LPS, sTSO, WASO, sWASO, TST, sTST, AE
						DAR 10 mg (54)					
						DAR 25 mg (55)			Black or African American 5		
						DAR 50 mg (56)					
						PLA (54)			American Indian or Alaska Native 2		
Mignot et al.	930	Australia, Canada, Denmark, Germany, Italy, Poland, Serbia, Spain, Switzerland, and the USA	75	Double-blind, placebo-controlled parallel-group	≥18 y	DAR 25 mg (310)	31	55.8 ± 15.3	White 93	3 months	LPS, WASO, sTST, AE, SAE
									Black or African American 6		
									Asian 1		
									Other <1		
						DAR 50 mg (310)	36	55.5 ± 15.3	White 88		
									Black or African American 10		
Trial 1 NCT03545191				RCT					Asian 1		
									Other 1		
						PLA (310)	32	55.1 ± 15.4	White 90		
									Black or African American 9		
									Asian 1		
									Other 1		
Mignot et al.	924	Belgium, Bulgaria, Canada, Czech Republic, Finland, France, Germany, Hungary, South Korea, Sweden, and the USA	81	Double-blind, placebo-controlled parallel-group RCT	≥18 y	DAR 10 mg (307)	30	57.1 ± 14.0	White 89	3 months	LPS, WASO, sTST, AE, SAE
									Black or African American 5		
									Asian 5		
									Other 1		
						DAR 25 mg (309)	29	56.3 ± 14.4	White 88		
									Black or African American 8		
Trial 2 NCT03575104									Asian 4		
									Other <1		
						PLA (308)	33	56.7 ± 14.1	White 87		
									Black or African American 9		
									Asian 3		
									Other 1		

^a^ N included 263 participants in zolpidem group;

^b^ N included 60 participants in zolpidem group; The percentages of race in some articles do not sum to 100 due to rounding.

SUV, suvorexant; LEM, lemborexant; DAR, daridorexant; PLA, placebo; LPS, latency to persistent sleep; sTSO, subjective time to sleep onset; WASO, wake after sleep onset; sWASO, subjective wake after sleep onset; TST, total sleep time; sTST, subjective total sleep time; ISI, insomnia severity index score; AE, adverse events; SAE, serious adverse events.

The risks of bias for all enrolled studies are shown in Supplementary Figure S1. Only one clinical trial showed unclear risks of bias in allocation concealment, blinding of participants and personnel and blinding of outcome assessments. For incomplete outcome data, the risk of bias was also unclear in one trial. For selective reporting, the risk of bias was unclear in one study and high in another study. In addition to these items, unclear risks of bias were also observed in two RCTs. According to the symmetry of the funnel plots, no obvious publication bias was found among the included trials **(** Supplementary Table S5 **)**.

### 3.1 The efficacy and safety of different doses of FDA-approved DORAs compared with placebo

Our efficacy outcomes included objective and subjective sleep maintenance and onset outcomes, as well as the patient-evaluated outcome ISI. For the sleep onset outcomes LPS and sTSO, suvorexant 20 and 40 mg, lemborexant 5 and 10 mg, and daridorexant 10 and 50 mg were more effective than placebo, with MDs (95% CrI) ranging between −7.31 (95% CrI −10.93 to −3.68) for daridorexant 10 mg and −13.60 (95% CrI −21.47 to −5.73) for lemborexant 10 mg in terms of LPS, and MDs (95% CrI) ranging between −2.64 (95% CrI −3.73 to −1.54) for daridorexant 10 mg and −16.55 (95% CrI −21.89 to −11.20) for lemborexant 10 mg in terms of sTSO. For WASO and sWASO, most interventions were more effective than placebo, except that patients in the daridorexant 5 mg group did not show significantly shorter WASO (MD -2.96 95% CrI −10.59 to 4.66), and patients in the daridorexant 10 mg group even showed a worse sWASO (MD 1.84, 95% CrI 0.29–3.39) than those in the placebo group. For the sleep maintenance outcomes TST and sTST, suvorexant 10 mg and daridorexant 5 mg, as respective minimum doses, were “as effective as placebo”. Other interventions might have been superior to placebo with low to very low certainty. For ISI, suvorexant 20 and 40 mg and lemborexant 5 and 10 mg were more effective than placebo, with MDs ranging between −1.20 (95% CrI: -1.66 to −0.74) for suvorexant 20 mg and −1.80 (95% CrI: -3.01 to −0.58) for lemborexant 10 mg.

**FIGURE 3:**
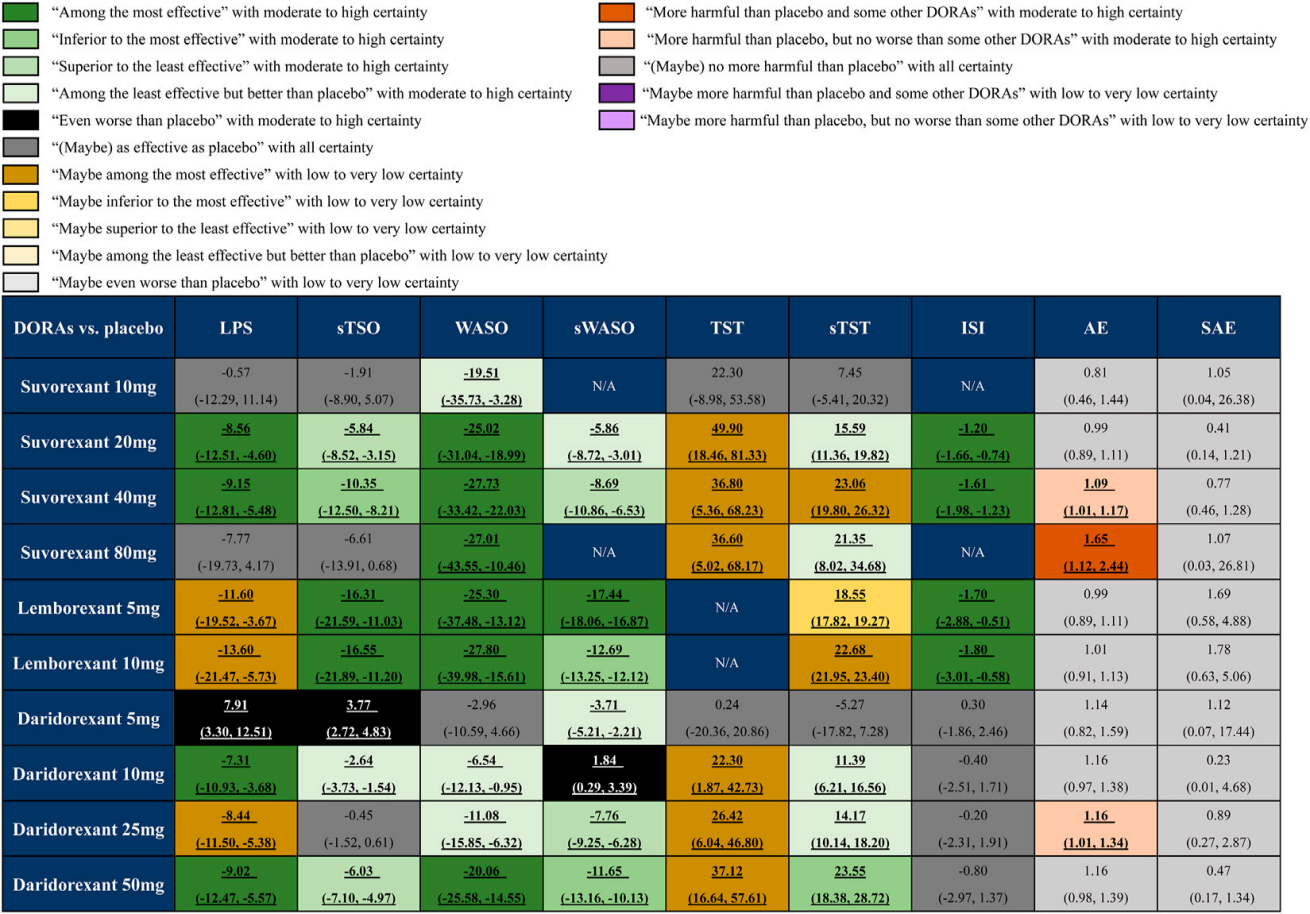
Summary of effect sizes of different approved dual orexin receptor antagonists (DORAs) on efficacy and safety outcomes against placebo. The certainty of evidence was rated by the Grading of Recommendations Assessment, Development, and Evaluation criteria. The clinical importance was classified according to the minimally contextualised framework. The different categories show how clinically important the effect is, whereas the certainty of evidence shows whether the effect is trustworthy or not. For efficacy, data are in mean difference (95% credible interval [CrI]). For latency to persistent sleep (LPS), subjective time to sleep onset (sTSO), wake time after sleep onset (WASO), subjective wake time after sleep onset (sWASO) and insomnia severity index (ISI), data below 0 favour the DORAs treatment. For total sleep time (TST) and subjective total sleep time (sTST), data above 0 favour the DORAs treatment. For safety, data are risk ratio (95% CrI), and data above 1 favour the placebo treatment. Bold and underlined text represents statistical significance.

For safety, we combined the data collected from the 11 included trials and found that only suvorexant 40 mg (RR: 1.09, 95% CrI: 1.01–1.17), suvorexant 80 mg (RR: 1.65, 95% CrI: 1.12–2.44) and daridorexant 25 mg (RR: 1.16, 95% CrI: 1.01–1.34) showed a significantly higher risk of AEs than placebo. No significant differences were found between any dose of FDA-approved DORAs and placebo in terms of SAEs. The detailed results are also presented in Figure 3.

### 3.2 The efficacy and safety of different doses of each FDA-approved DORA

**FIGURE 4:**
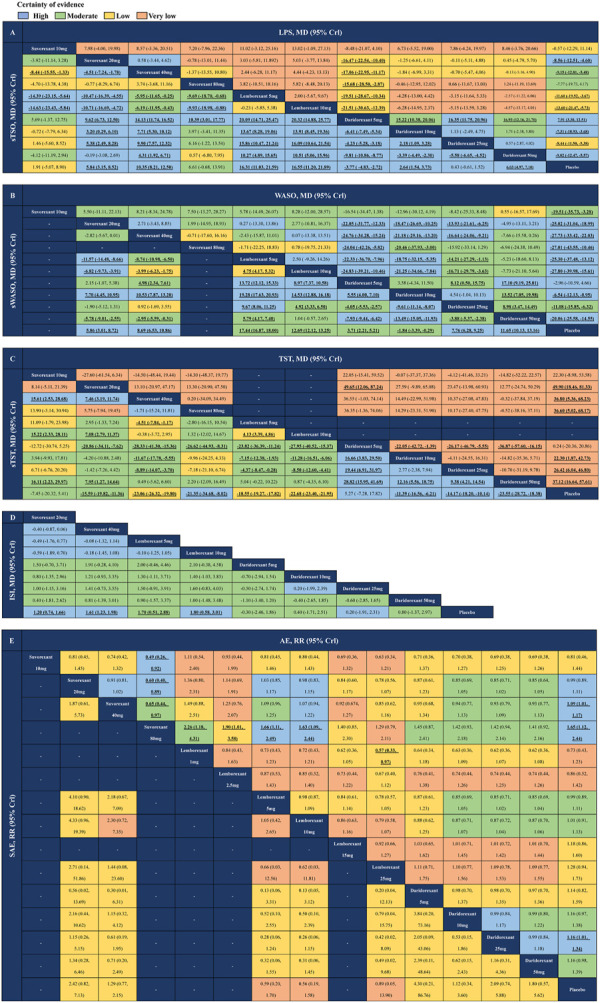
League tables of outcome analyses. Comparisons should be read from left to right. Efficacy and safety estimates are located at the intersection between the column-defining treatment and the row-defining treatment. **(A)** latency to persistent sleep (LPS) and subjective time to sleep onset (sTSO). **(B)** wake time after sleep onset (WASO) and subjective wake time after sleep onset (sWASO). **(C)** total sleep time (TST) and subjective total sleep time (sTST). **(D)** insomnia severity index (ISI). **(E)** adverse effects (AEs) and serious adverse effects (SAEs). For efficacy **(A–D)**, data are in mean difference (95% credible interval [CrI]). For **A, B** and **D**, data above 0 favour the column-defining treatment. For **C**, data above 0 favour the row-defining treatment. For safety **(E)**, data are risk ratio (95% CrI), and data above 1 favour the column-defining treatment. Bold and underlined text represents statistical significance.

The network estimates of all comparisons are illustrated in Figure 4. The color of each cell indicates the certainty of evidence according to the GRADE. Further details of the GRADE evaluation can be found in eTable 6. The results indicated that lemborexant 5 mg and 10 mg was more effective than any other approved DORA for sTSO (MDs ranging between −5.95 and 20.32, moderate to high certainty), while daridorexant 5 mg was less effective than all DORAs except suvorexant 10 mg for both sTSO and LPS (Figure 4A). For sWASO, lemborexant 5 mg was more effective than any other approved DORA, including lemborexant 10 mg (MDs ranging between −11.57 and 19.28, low to high certainty). For WASO, suvorexant 20 and 40 mg and lemborexant 5 and 10 mg were significantly more effective than daridorexant 5, 10 and 25 mg (Figure 4B). Similarly, lemborexant 5 and 10 mg was also significantly more effective than daridorexant 5, 10 and 25 mg for sTST (MDs ranging between −27.95 and −4.37, moderate certainty, Figure 4C). No significant differences were found between any of the two DORAs in terms of ISI (Figure 4D). As suggested in Figure 4E, suvorexant 80 mg resulted in more AEs than many other interventions and lemborexant 1 mg might have caused fewer AEs than lemborexant 25 mg. Except for these, there was no other significant difference between any DORA comparisons in terms of AEs and SAEs from the collected data.

### 3.3 SUCRAs of FDA-approved DORAs and placebo

**FIGURE 5:**
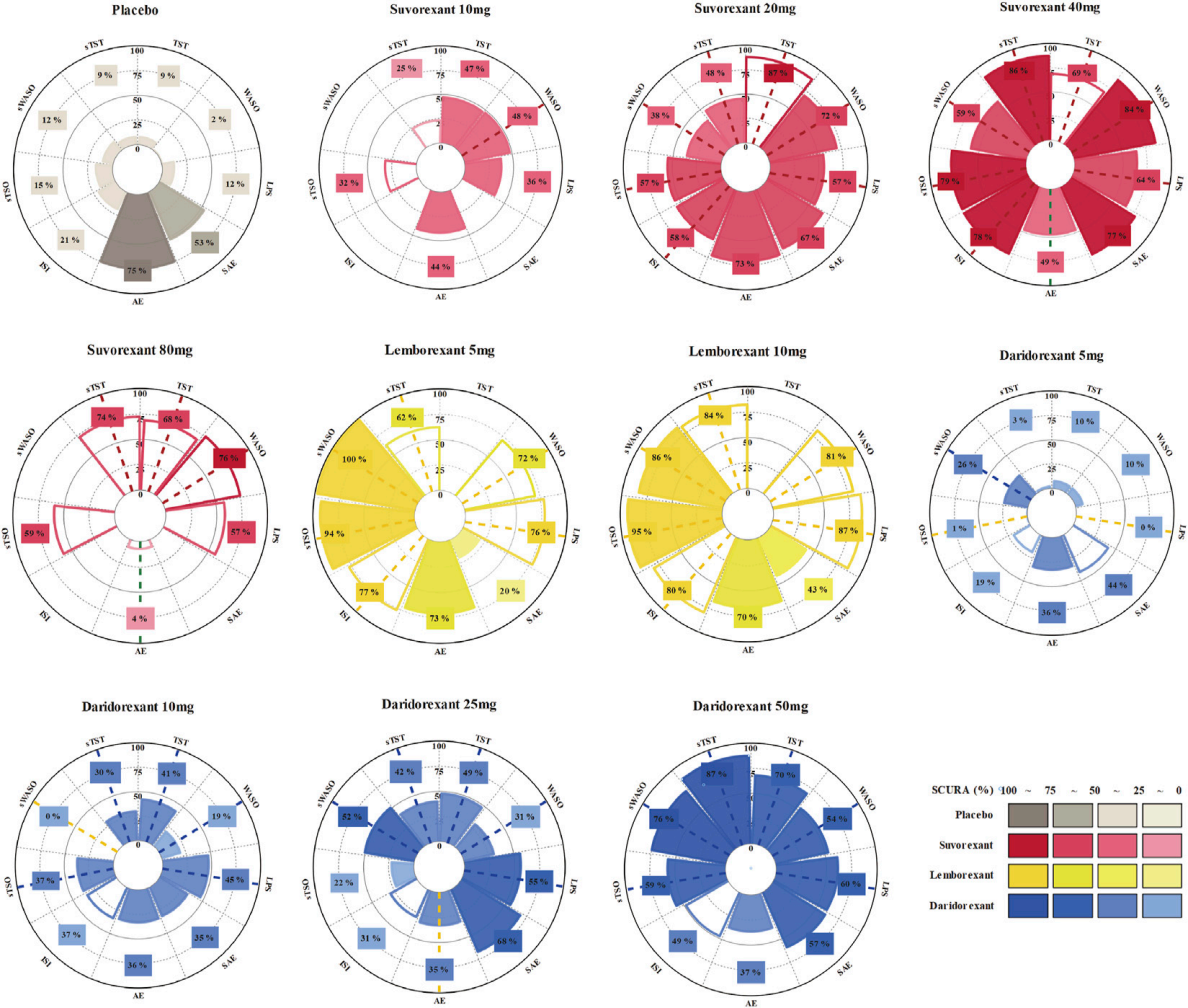
Rankings of the effects of different approved dual orexin receptor antagonists (DORAs) and placebo on efficacy and safety outcomes measured with surfaces under the curve ranking areas (SUCRAs). SUCRAs for each outcome are shown in Vitruvian (radar) graphs. The SUCRA value indicates the probability of being in the highest rank for an intervention. Consistent colored dash lines indicate interventions with significant better effect sizes (more effective/safe) compared with placebo and opposite colored dash lines indicate worse effect sizes. Blank circular sectors represent interventions of a single trial.

As shown in Figure 5, the SUCRA values of the seven efficacy and two safety outcomes of the three drugs with specific different doses and placebo demonstrated that lemborexant 10 mg had the highest SUCRA values in terms of LPS (87%), sTSO (95%) and ISI (80%); lemborexant 5 mg had the highest SUCRA values for sWASO (100%); suvorexant 40 mg had the highest SUCRA values for WASO (84%); suvorexant 20 mg had the highest SUCRA values for TST (87%); and daridorexant 50 mg had the highest SUCRA value for sTST (87%). Regarding safety, the SUCRA value of the placebo for AEs was higher than that of any of the DORAs (75%), and suvorexant 40 mg yielded the highest SUCRA value for SAEs (77%).

### 3.4 Subgroup analysis

**FIGURE 6:**
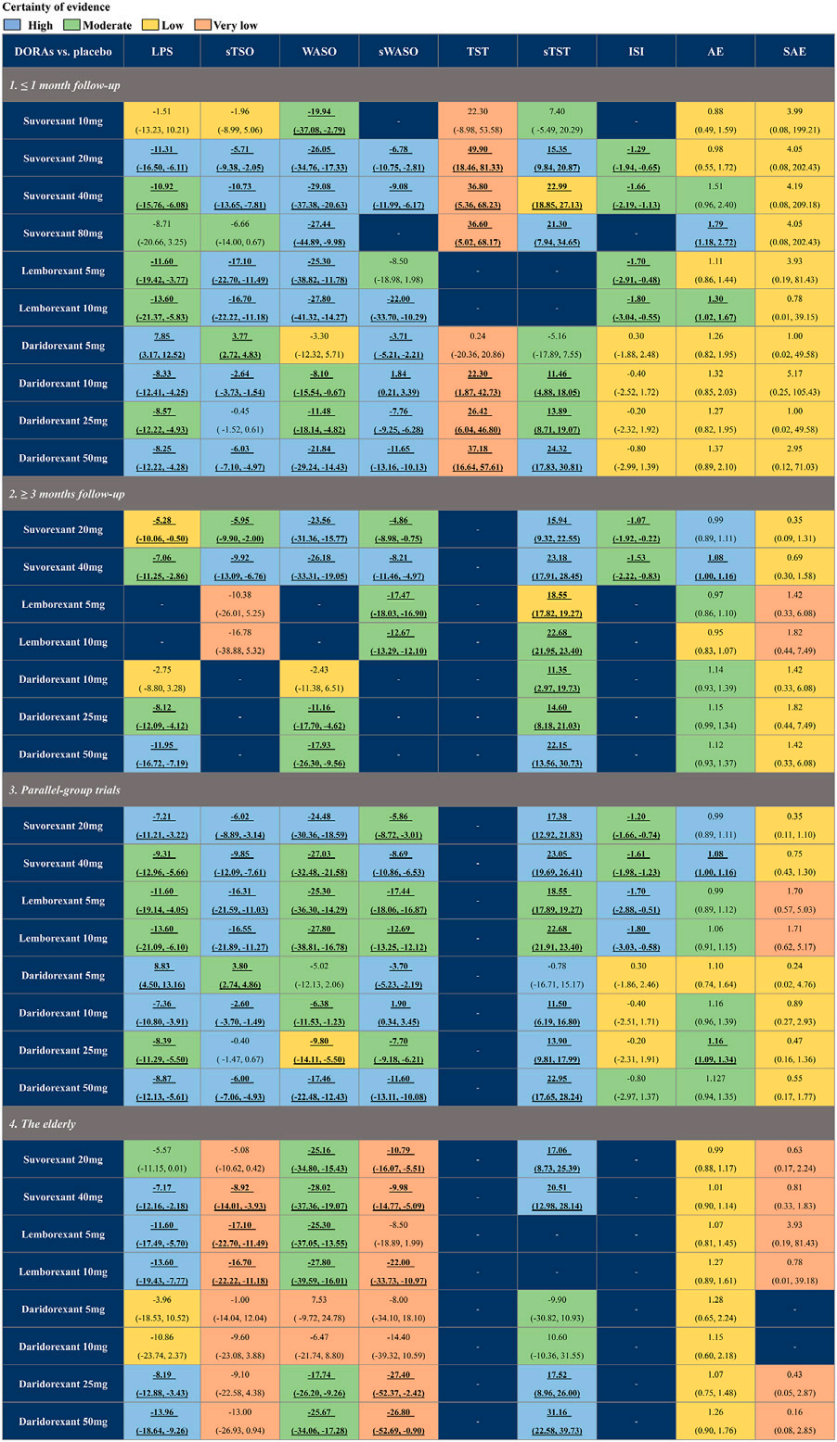
Summary of subgroup analysis of different approved dual orexin receptor antagonists (DORAs) on efficacy and safety outcomes against placebo. The certainty of evidence was rated by the Grading of Recommendations Assessment, Development, and Evaluation criteria. Bold and underlined text represents statistical significance. For efficacy, data are in mean difference (95% credible interval [CrI]). For latency to persistent sleep (LPS), subjective time to sleep onset (sTSO), wake time after sleep onset (WASO), subjective wake time after sleep onset (sWASO) and insomnia severity index (ISI), data below 0 favour the DORAs treatment. For total sleep time (TST) and subjective total sleep time (sTST), data above 0 favour the DORAs treatment. For safety, data are risk ratio (95% CrI), and data above 1 favour the placebo treatment. Bold and underlined text represents statistical significance.

To assess the influence of different times of follow-up (≤1 month and ≥3 months), study design (parallel-group trials), and age (elderly individuals [≥55 for females and ≥65 for males]), we implemented subgroup analyses at baseline (Figure 6). The results indicated that for efficacy, the comparisons in each subgroup were similar to the primary analysis. However, for safety, if the follow-up time was shortened to ≤1 month, the risk of AEs of suvorexant 40 mg would not be significantly different from that of placebo (RR: 1.51, 95% CrI: 0.96 to 2.40, moderate certainty). In the subgroup analysis of age, we found that suvorexant 40 mg (RR: 1.01, 95% CrI: 0.90 to 1.14, low certainty) and daridorexant 25 mg (RR: 1.07, 95% CrI: 0.75 to 1.48, low certainty), which were associated with a higher risk of AEs in the primary analysis, were generally well tolerated in elderly individuals. The detailed results of the subgroup analyses are shown in Supplementary Tables S7–44.

### 3.5 Convergence, heterogeneity, consistency, and sensitivity analysis of all outcomes

All potential scale reduction factor values of the various parameters that were restricted to 1 demonstrated good convergence efficiency **(** Supplementary Figure S2–10 **)**. In addition, the trace and density of Markov chains also showed that the establishment of the Bayesian network model was successful (Supplementary Figure S11–19).

To analyze the heterogeneity among the selected studies in the NMA, we performed a heterogeneity analysis on each outcome. Supplementary Figures S20–27 depict the pairwise network heterogeneity and exhibited acceptable I^2^ in most comparisons. Substantial heterogeneity was consistently present in the study carried out by Zammit et al., which was a crossover study with a two-day follow-up duration that might have caused problems.

Global inconsistency was assessed by the construction of the consistency model and the inconsistency model, which found that the DIC difference between the two models was less than 11 (Supplementary Table S4) and verified that the consistency models were reliable. In addition, node-splitting analysis demonstrated that the comparison in LPS showed some inconsistency but there were no other evident anomalies in the network model with indirect sources, as illustrated in Supplementary Figures S28–32. Overall, the consistency model’s results were reliable.

For sensitivity analysis, two studies with moderate risk of biases were excluded, and the results seemed similar to the primary network analysis except that lemborexant 5 mg was not superior to placebo in terms of sWASO (Supplementary Table S45–49). Besides, given that the substantial heterogeneity was consistently present in the study carried out by Zammit et al., we also perform a sensitivity analysis to see the effect of excluding this study. The results seemed similar to the primary network analysis as well (Supplementary Table S50–55). There was no difference between results in FDA-approved doses and the primary analysis (Supplementary Table 56–64). This might indicate that the results of the main analysis were robust.

## 4 Discussion

The present study included 9 RCTs with 7257 individuals randomly assigned to different doses of suvorexant, lemborexant, daridorexant, or placebo. Our results showed that compared to placebo, suvorexant, lemborexant, and daridorexant were generally more effective with respect to LPS, sTSO, WASO, sWASO, TST, sTST, and ISI, and higher doses appeared more effective than lower doses. The safety of FDA-approved DORAs at different doses was not inferior to that of placebo, except for suvorexant 40 mg, suvorexant 80 mg, and daridorexant 25 mg. Additionally, even with high doses, suvorexant, lemborexant, and daridorexant were not associated with a higher risk of SAEs compared with the placebo. Therefore, we found that suvorexant 20 mg, lemborexant 5 mg, lemborexant 10 mg, and daridorexant 50 mg might be good choices because they improved most of the efficacy without increasing the safety risk compared with placebo.

Suvorexant was the first DORA approved for the treatment of primary insomnia (Coleman et al., 2017). The current study indicated that suvorexant, particularly at doses of 20 mg and 40 mg, showed better results than the placebo in all efficacy measures. In addition, 20 mg and 40 mg of suvorexant were among the most effective treatments for decreasing LPS, WASO, and ISI and possibly the most effective treatment for increasing TST. Moreover, no significant differences in efficacy were found between 20 mg suvorexant and 40 mg suvorexant, except for sTSO and sTST. However, compared with the placebo, a greater incidence of AEs was seen with increasing doses of suvorexant (Herring et al., 2012; Herring et al., 2016a); the differences were statistically significant when the doses were greater than or equal to 40 mg. Thus, due to safety concerns (Sutton, 2015; Atkin et al., 2018), only doses of 5 mg, 10 mg, 15 mg, and 20 mg were approved in the United States, and doses of 15 mg and 20 mg were approved in Japan in 2014 (Coleman et al., 2017).

Lemborexant received approval in the United States and Japan as the treatment for patients with insomnia in 2019 and 2020, respectively (Scott, 2020). In the present study, 5 mg and 10 mg lemborexant were more effective than placebo in all efficacy categories, and these doses were among the most effective treatments for sTSO, sWASO, and ISI. In addition, lemborexant 5 mg ranked first in sWASO, and lemborexant 10 mg possibly ranked first in sTST. This finding was consistent with the previous conclusion that lemborexant, particularly at a dose of 10 mg/d, produced better improvement in sTSO, sWASO, and sTST than low-dose suvorexant (Kishi et al., 2020). This result also agreed with our previous meta-analysis showing that lemborexant had greater effect sizes for sTSO and sWASO than suvorexant (Wu et al., 2022; Xue et al., 2022). Although subjectively perceived sleep improvement is essential for patients, we could still not draw a firm conclusion that lemborexant was superior to any doses of suvorexant or daridorexant because there was no evidence that lemborexant was superior to other drugs in objective measures (PSG). This inconsistency may be due to a mismatch of subjective-objective measures in sleep perception among people with insomnia (Silva et al., 2007; Bianchi et al., 2013). Compared with placebo, the changes in endpoints of subjective measures tended to be less than those of objective measures for suvorexant (Herring et al., 2016b). Our results on the safety of lemborexant demonstrated that different doses of lemborexant were not associated with a higher risk of AEs and SAEs than the placebo. Additionally, there was no difference in safety among different doses of lemborexant, except that lemborexant 1 mg was safer than lemborexant 25 mg. However, we were unable to compare the efficacy data of patients who received doses of lemborexant other than 5 mg and 10 mg due to the lack of information (Murphy et al., 2017).

Daridorexant, the latest FDA-approved DORA, received approval in 2022 at doses of 25 mg and 50 mg for the treatment of patients with insomnia (Markham, 2022). In the current study, daridorexant 5 mg was not superior to placebo except for sWASO. Daridorexant 10 mg demonstrated greater efficacy than the placebo in LPS and TST. However, daridorexant 10 mg was associated with even worse efficacy in sWASO than lemborexant, suvorexant, and placebo. Daridorexant, at doses of 25 mg and 50 mg, was more effective than placebo in all efficacy outcomes except ISI. From the SUCRA data, daridorexant 50 mg had better results in increasing sTST than any other DORA or the placebo. However, it is interesting to note that daridorexant 25 mg was inferior to the placebo in terms of AEs, whereas the differences between daridorexant 50 mg and the placebo were not statistically significant. This may be attributed to the incidence of fatigue and somnolence from the daridorexant 50 mg treatment being lower than that from the daridorexant 25 mg treatment in several included studies (Dauvilliers et al., 2020; Mignot et al., 2022). However, daytime impairments, such as fatigue and somnolence, can be caused by insomnia (Buysse, 2013). Therefore, this finding may be due to daridorexant 50 mg yielding better sleep during the night, which helps reduce somnolence and fatigue (Mignot et al., 2022). Although daridorexant 50 mg showed better efficacy and safety than daridorexant 25 mg in our results, this did not mean that daridorexant 25 mg was not recommended.

Further subgroup analysis showed that suvorexant at doses of 20mg and 40 mg have excellent efficacy and safety in the short follow-up. However, suvorexant 40 mg was associated with a higher risk of AEs compared to the placebo in studies with a long-term follow-up. This might indicate that the risk of 40 mg suvorexant may differ over time (Michelson et al., 2014; Herring et al., 2016b) and explain why the FDA had approved suvorexant only at a dose below 20 mg for the treatment of primary insomnia (Coleman et al., 2017). The efficacy and safety of lemborexant differed between short- and long-term follow-ups, possibly because only one RCT was included in the short-term and long-term subgroup analyses, respectively. We found that in elderly patients, suvorexant 40 mg, lemborexant 10 mg, and daridorexant 25 mg and 50 mg had remarkable efficacy and safety. In addition, suvorexant 40 mg and daridorexant 25 mg, associated with a higher risk of AEs in primary outcomes, were generally well tolerated in elderly individuals. This conclusion is consistent with that of Fietze et al., who reported that the clinical benefit of daridorexant 50 mg was greater than that of daridorexant 25 mg in elderly patients (Fietze et al., 2022). In several included RCTs (Michelson et al., 2014; Herring et al., 2016a; Herring et al., 2016b), the suvorexant dose in the elderly group was adjusted to 30 mg (40 mg for nonelderly individuals) and 15 mg (20 mg for nonelderly individuals) while taking into account tolerance and pharmacokinetics (Herring et al., 2017). This may explain why suvorexant 40 mg is better tolerated in elderly individuals. However, based on the FDA’s view that the lowest effective dose of treatment should be used, the recommended dose for elderly people in the United States followed those for nonelderly people (the recommended dose is 10 mg to a maximum of 20 mg) (Herring et al., 2017).

Compared to our previous study (Xue et al., 2022), the current research conducts a comprehensive comparison between different doses of FDA-approved DORAs and includes two more large RCTs on daridorexant with approximately 2,000 participants, which increases the credibility of the evidence on daridorexant. Subgroup analyses are also conducted, and the GRADE approach is used to determine the certainty of evidence. The results revealed that lemborexant were more effective than daridorexant 25 mg while remaining equally safe. This finding indicates that lemborexant may be a better candidate for recommendation than daridorexant 25 mg. Contrary to the FDA recommendation (suvorexant 10 mg as the best-recommended dose), our study shows that suvorexant 10 mg has poor efficacy despite a high safety profile. In addition, we discovered that increasing lemborexant has diminishing returns, yet doubling suvorexant (20 mg) and daridorexant (50 mg) may be more meaningful. However, the pharmacokinetics of the drugs should also be considered. The times to peak concentration of the three drugs were similar and all had a 1–2 h delay in the time to peak concentration after eating high-fat, high-calorie meals. The half-life of daridorexant was shorter than lemborexant and suvorexant (Preskorn, 2022). Thus, particular prescription choices for individual patients should be considered on a case-by-case basis. Based on the findings of our subgroup analysis, an increase in the maximum therapeutic dose of suvorexant to 30 mg for elderly patients could be considered. More RCTs should be available in the future to evaluate the efficacy of suvorexant 30 mg in the nonelderly.

Inevitably, there were several limitations of the present meta-analysis. First, our NMA was based on limited data. Despite a comprehensive literature search, only nine published RCTs with eleven trials were pooled to evaluate the efficacy and safety of FDA-approved DORAs at different doses. Second, although we performed subgroup analyses based on the time of follow-up, the study design, age, variation in the inclusion and exclusion criteria, and baseline characteristics (e.g., sex, study region, race) may have also caused discrepancies. At the same time, the different variables the treatment was randomised for (e.g., the randomization of some studies was stratified by age while some were stratified by geographical region or both) may impact the bias in the meta-analysis in non-obvious ways. Third, due to the local inconsistency between direct and indirect evidence, the comparisons between suvorexant 20 mg vs. 10mg and daridorexant 50 mg vs. 10mg in LPS must be interpreted cautiously. We also performed sensitivity analyses, including trials with a low risk of bias. The sensitivity analyses for sTSO, sTST, AEs, and SAEs demonstrated that all the statistics were robust. However, when studies at moderate to high risk of bias were excluded from the primary outcomes, lemborexant 5 mg was not superior to placebo for sWASO. Finally, the limitations inherent to the meta-analysis methodology need to be emphasized; more trials are still needed to compare the efficacy and safety of FDA-approved DORAs.

## 5 Conclusion

In conclusion, we found that the FDA-approved doses of DORAs currently exhibit strong efficacy and safety, except suvorexant 10 mg, which was found to be safe but not as effective as others. Furthermore, FDA-approved doses of DORAs and suvorexant 30 mg are well tolerated in elderly individuals. Our findings suggest that suvorexant 20 mg, lemborexant 5 mg, lemborexant 10 mg, and daridorexant 50 mg represent suitable approaches for insomnia.

## Figures and Tables

**Table:** 

**ACP**	American College of Physicians
**AEs**	adverse effects
**BBT**	brief behavioral therapy
**CBT-I**	cognitive behavioral therapy for insomnia
**CI**	confidence interval
**CINeMA**	Confidence in Network Meta-Analysis framework
**CrI**	credible interval
**DIC**	deviance information criteria
**DORA**	dual orexin receptor antagonist
**DSM-IV-TR**	Diagnostic and Statistical Manual of Mental Disorders, IVth edition, text revision
**DSM-V**	Diagnostic and Statistical Manual of Mental Disorders, Vth edition
**GABA**	gamma aminobutyric acid
**GPCRs**	G-protein-coupled receptors
**GRADE**	Grading of Recommendations, Assessment, Development, and Evaluation
**ISI**	insomnia severity index score
**LPS**	latency to persistent sleep
**MD**	mean difference
**NMA**	network meta-analysis
**ORAs**	orexin receptor antagonists
**OX1R**	orexin receptor 1
**OX2R**	orexin receptor 2
**PSG**	polysomnography
**RCTs**	randomized controlled trials
**RR**	risk ratio
**SAEs**	serious adverse effects
**SORAs**	selective orexin receptor antagonists
**sTSO**	and subjective time to sleep onset
**sTST**	subjective total sleep time
**SUCRA**	surface under the curve ranking area
**sWASO**	subjective wake time after sleep onset
**TST**	total sleep time
**WASO**	wake time after sleep onset.

## Data Availability

The original contributions presented in the study are included in the article/Supplementary Material, further inquiries can be directed to the corresponding authors.

## References

[B1] Atkin T. Comai S. Gobbi G. (2018). Drugs for insomnia beyond benzodiazepines: Pharmacology, clinical applications, and discovery. Pharmacol. Rev. 70, 197–245. 10.1124/pr.117.014381 29487083

[B2] Atkins D. Best D. Briss P. A. Eccles M. Falck-Ytter Y. Flottorp S. (2004). Grading quality of evidence and strength of recommendations. BMJ Clin. Res. ed) 328, 1490. 10.1136/bmj.328.7454.1490 PMC428525 15205295

[B3] Barbar S. I. Enright P. L. Boyle P. Foley D. Sharp D. S. Petrovitch H. (2000). Sleep disturbances and their correlates in elderly Japanese American men residing in Hawaii. J. Gerontol. A Biol. Sci. Med. Sci. 55, M406–M411. 10.1093/gerona/55.7.m406 10898258

[B4] Bastien C. H. Vallieres A. Morin C. M. (2001). Validation of the Insomnia Severity Index as an outcome measure for insomnia research. Sleep. Med. 2, 297–307. 10.1016/s1389-9457(00)00065-4 11438246

[B5] Bianchi M. T. Williams K. L. McKinney S. Ellenbogen J. M. (2013). The subjective-objective mismatch in sleep perception among those with insomnia and sleep apnea. J. Sleep. Res. 22, 557–568. 10.1111/jsr.12046 23521019

[B6] Bollu P. C. Kaur H. (2019). Sleep medicine: Insomnia and sleep. Mo Med. 116, 68–75. 30862990 PMC6390785

[B7] Brignardello-Petersen R. Florez I. D. Izcovich A. Santesso N. Hazlewood G. Alhazanni W. (2020). GRADE approach to drawing conclusions from a network meta-analysis using a minimally contextualised framework. BMJ 371, m3900. 10.1136/bmj.m3900 33177059

[B8] Buysse D. J. (2013). Insomnia. Jama 309, 706–716. 10.1001/jama.2013.193 23423416 PMC3632369

[B9] Coleman P. J. Gotter A. L. Herring W. J. Winrow C. J. Renger J. J. (2017). The discovery of suvorexant, the first orexin receptor drug for insomnia. Annu. Rev. Pharmacol. Toxicol. 57, 509–533. 10.1146/annurev-pharmtox-010716-104837 27860547

[B10] Crowley K. (2011). Sleep and sleep disorders in older adults. Neuropsychol. Rev. 21, 41–53. 10.1007/s11065-010-9154-6 21225347

[B11] Dauvilliers Y. Zammit G. Fietze I. Mayleben D. Seboek Kinter D. Pain S. (2020). Daridorexant, a new dual orexin receptor antagonist to treat insomnia disorder. Ann. Neurol. 87, 347–356. 10.1002/ana.25680 31953863

[B12] Dias S. Welton N. J. Caldwell D. M. Ades A. E. (2010). Checking consistency in mixed treatment comparison meta‐analysis. Statistics Med. 29, 932–944. 10.1002/sim.3767 20213715

[B13] Fietze I. Bassetti C. L. A. Mayleben D. W. Pain S. Seboek Kinter D. McCall W. V. (2022). Efficacy and safety of daridorexant in older and younger adults with insomnia disorder: A secondary analysis of a randomised placebo-controlled trial. Drugs & Aging 39, 795–810. 10.1007/s40266-022-00977-4 36098936 PMC9553778

[B14] Foley D. Ancoli-Israel S. Britz P. Walsh J. (2004). Sleep disturbances and chronic disease in older adults: Results of the 2003 national sleep foundation sleep in America survey. J. Psychosom. Res. 56, 497–502. 10.1016/j.jpsychores.2004.02.010 15172205

[B15] Herring W. J. Snyder E. Budd K. Hutzelmann J. Snavely D. Liu K. (2012). Orexin receptor antagonism for treatment of insomnia: A randomized clinical trial of suvorexant. Neurology 79, 2265–2274. 10.1212/WNL.0b013e31827688ee 23197752

[B16] Herring W. J. Connor K. M. Ivgy-May N. Snyder E. Liu K. Snavely D. B. (2016). Suvorexant in patients with insomnia: Results from two 3-month randomized controlled clinical trials. Biol. Psychiatry 79, 136–148. 10.1016/j.biopsych.2014.10.003 25526970

[B17] Herring W. J. Connor K. M. Snyder E. Snavely D. B. Zhang Y. Hutzelmann J. (2016). Suvorexant in patients with insomnia: Pooled analyses of three-month data from phase-3 randomized controlled clinical trials. J. Clin. Sleep. Med. 12, 1215–1225. 10.5664/jcsm.6116 27397664 PMC4990943

[B18] Herring W. J. Connor K. M. Snyder E. Snavely D. B. Zhang Y. Hutzelmann J. (2017). Suvorexant in elderly patients with insomnia: Pooled analyses of data from phase III randomized controlled clinical trials. Am. J. Geriatric Psychiatry 25, 791–802. 10.1016/j.jagp.2017.03.004 28427826

[B19] Higgins J. P. Altman D. G. Gotzsche P. C. Jüni P. Moher D. Oxman A. D. (2011). The Cochrane Collaboration's tool for assessing risk of bias in randomised trials. BMJ Clin. Res. ed) 343, d5928. 10.1136/bmj.d5928 PMC3196245 22008217

[B20] Javaheri S. Redline S. (2017). Insomnia and risk of cardiovascular disease. Chest 152, 435–444. 10.1016/j.chest.2017.01.026 28153671 PMC5577359

[B21] Kishi T. Nomura I. Matsuda Y. Sakuma K. Okuya M. Ikuta T. (2020). Lemborexant vs suvorexant for insomnia: A systematic review and network meta-analysis. J. Psychiatric Res. 128, 68–74. 10.1016/j.jpsychires.2020.05.025 32531478

[B22] Liberati A. Altman D. G. Tetzlaff J. Mulrow C. Gøtzsche P. C. Ioannidis J. P. A. (2009). The PRISMA statement for reporting systematic reviews and meta-analyses of studies that evaluate healthcare interventions: Explanation and elaboration. BMJ 339, b2700. 10.1136/bmj.b2700 19622552 PMC2714672

[B23] Markham A. (2022). Daridorexant: First approval. Drugs 82, 601–607. 10.1007/s40265-022-01699-y 35298826 PMC9042981

[B24] Michelson D. Snyder E. Paradis E. Chengan-Liu M. Snavely D. B. Hutzelmann J. (2014). Safety and efficacy of suvorexant during 1-year treatment of insomnia with subsequent abrupt treatment discontinuation: A phase 3 randomised, double-blind, placebo-controlled trial. Lancet Neurology 13, 461–471. 10.1016/S1474-4422(14)70053-5 24680372

[B25] Mignot E. Mayleben D. Fietze I. Leger D. Zammit G. Bassetti C. L. A. (2022). Safety and efficacy of daridorexant in patients with insomnia disorder: Results from two multicentre, randomised, double-blind, placebo-controlled, phase 3 trials. Lancet Neurology 21, 125–139. 10.1016/S1474-4422(21)00436-1 35065036

[B26] Morin C. M. Jarrin D. C. (2022). Epidemiology of insomnia: Prevalence, course, risk factors, and public health burden. Sleep. Med. Clin. 17, 173–191. 10.1016/j.jsmc.2022.03.003 35659072

[B27] Murphy P. Moline M. Mayleben D. Rosenberg R. Zammit G. Pinner K. (2017). Lemborexant, A dual orexin receptor antagonist (DORA) for the treatment of insomnia disorder: Results from a bayesian, adaptive, randomized, double-blind, placebo-controlled study. J. Clin. Sleep. Med. 13, 1289–1299. 10.5664/jcsm.6800 29065953 PMC5656478

[B28] Nikolakopoulou A. Higgins J. P. T. Papakonstantinou T. Chaimani A. Del Giovane C. Egger M. (2020). CINeMA: An approach for assessing confidence in the results of a network meta-analysis. PLoS Med. 17, e1003082. 10.1371/journal.pmed.1003082 32243458 PMC7122720

[B29] Perlis M. L. Pigeon W. R. Grandner M. A. Bishop T. M. Riemann D. Ellis J. G. (2021). Why treat insomnia? J. Prim. Care Community Health 12, 21501327211014084. 10.1177/21501327211014084 34009054 PMC8138281

[B30] Perlis M. L. Posner D. Riemann D. Bastien C. H. Teel J. Thase M. (2022). Insomnia. Lancet 400, 1047–1060. 10.1016/S0140-6736(22)00879-0 36115372

[B31] Preskorn S. H. (2022). Comparative Pharmacology of the 3 marketed dual orexin antagonists—daridorexant, lemborexant, and suvorexant: Part 1: Pharmacokinetic profiles. J. Psychiatric Practice® 28, 478–480. 10.1097/PRA.0000000000000672 36355586

[B32] Qaseem A. Kansagara D. Forciea M. A. Cooke M. Denberg T. D. Clinical Guidelines Committee of the American College of Physicians (2016). Management of chronic insomnia disorder in adults: A clinical practice guideline from the American College of Physicians. Ann. Intern. Med. 165, 125–133. 10.7326/M15-2175 27136449

[B33] Roecker A. J. Cox C. D. Coleman P. J. (2016). Orexin receptor antagonists: New therapeutic agents for the treatment of insomnia. J. Med. Chem. 59, 504–530. 10.1021/acs.jmedchem.5b00832 26317591

[B34] Roth T. Coulouvrat C. Hajak G. Lakoma M. D. Sampson N. A. Shahly V. (2011). Prevalence and perceived health associated with insomnia based on DSM-IV-TR; international statistical classification of diseases and related health problems, tenth revision; and research diagnostic criteria/international classification of sleep disorders, second edition criteria: Results from the America insomnia survey. Biol. Psychiatry 69, 592–600. 10.1016/j.biopsych.2010.10.023 69 21195389

[B35] Scott L. J. (2020). Lemborexant: First approval. Drugs 80, 425–432. 10.1007/s40265-020-01276-1 32096020

[B36] Silva G. E. Goodwin J. L. Sherrill D. L. Arnold J. L. Bootzin R. R. Smith T. (2007). Relationship between reported and measured sleep times: The sleep heart health study (SHHS). J. Clin. Sleep. Med. 3, 622–630. 10.5664/jcsm.26974 17993045 PMC2045712

[B37] Spiegelhalter D. J. Best N. G. Carlin B. P. Van Der Linde A. (2002). Bayesian measures of model complexity and fit. J. R. Stat. Soc. Ser. b Stat. Methodol. 64, 583–639. 10.1111/1467-9868.00353

[B38] Sun Y. Tisdale R. K. Kilduff T. S. (2021). Hypocretin/orexin receptor Pharmacology and sleep phases. Front. Neurol. Neurosci. 45, 22–37. 10.1159/000514963 34052813 PMC8171809

[B39] Sutton E. L. (2015). Profile of suvorexant in the management of insomnia. Drug Des. Dev. Ther. 9, 6035–6042. 10.2147/DDDT.S73224 PMC4651361 26648692

[B40] Sutton E. L. (2021). Insomnia. Ann. Intern. Med. 174, ITC33–ITC48. 10.7326/AITC202103160 33683929

[B41] Uchiyama M. Kambe D. Imadera Y. Kajiyama Y. Ogo H. Uchimura N. (2022). Effects of TS-142, a novel dual orexin receptor antagonist, on sleep in patients with insomnia: A randomized, double-blind, placebo-controlled phase 2 study. Psychopharmacology 239, 2143–2154. 10.1007/s00213-022-06089-6 35296912 PMC9205809

[B42] Wu X. Xue T. Chen Z. Wang Z. Chen G. (2022). Orexin receptor antagonists and insomnia. Curr. Psychiatry Rep. 24, 509–521. 10.1007/s11920-022-01357-w 35972717

[B43] Xue T. Wu X. Chen S. Yang Y. Yan Z. Song Z. (2022). The efficacy and safety of dual orexin receptor antagonists in primary insomnia: A systematic review and network meta-analysis. Sleep. Med. Rev. 61, 101573. 10.1016/j.smrv.2021.101573 34902823

[B44] Yang L. P. H. (2014). Suvorexant: First global approval. Drugs 74, 1817–1822. 10.1007/s40265-014-0294-5 25227290

[B45] Yin J. Mobarec J. C. Kolb P. Rosenbaum D. M. (2015). Crystal structure of the human OX2 orexin receptor bound to the insomnia drug suvorexant. Nature 519, 247–250. 10.1038/nature14035 25533960

