# Supplementary material for: Different doses of dual orexin receptor antagonists in primary insomnia: a Bayesian network analysis
Source: Front Pharmacol. 2023 May 16;14:1175372. doi: 10.3389/fphar.2023.1175372 (PMC10228643; doi:10.3389/fphar.2023.1175372)

Different doses of dual orexin receptor antagonists in primary insomnia: A Bayesian network analysis

Tao Xue [1, #], Xin Wu [2, 3, #], Jiaxuan Li [2, #], Shujun Chen [4], Zilan Wang [2], Xin Tan [5], Zhong Wang [2, *], Jianguo Zhang [1, 6, 7, *]

[1] *Department of Neurosurgery, Beijing Tiantan Hospital, Capital Medical University, Beijing, China*

[2] *Department of Neurosurgery & Brain and Nerve Research Laboratory, The First Affiliated Hospital of Soochow University, Suzhou, Jiangsu Province, 215006, China*

[3] *Department of Neurosurgery, Suzhou Ninth People's Hospital, Suzhou, 215200, China*

[4] *Department of Neurology, Beijing Tiantan Hospital, Capital Medical University, Beijing, China*

[5] *Department of Neurology, The Affiliated Suzhou Hospital of Nanjing Medical University, Suzhou Municipal Hospital, Suzhou, Jiangsu Province, China*

[6] *Department of Neurosurgery, Beijing Neurosurgical Institute, Beijing, China*

[7] *Beijing Key Laboratory of Neurostimulation, Beijing, China*

[#] Tao Xue, Xin Wu and Jiaxuan Li contributed equally to this work.

*Corresponding author:

Jianguo Zhang, Department of Neurosurgery, Beijing Tiantan Hospital, Capital Medical University, No. 119 South 4th Ring West Road, Fengtai District, 100070, Beijing, China. Email address: zjguo73@126.com

Zhong Wang, Department of Neurosurgery, The First Affiliated Hospital of Soochow University, 188 Shizi Street, Suzhou 215006, China. E-mail address: wangzhong761@163.com.

Figure legends

eTable 1: Detailed search strategy.

eTable 2: Appendix of detailed excluded studies.

eTable 3: Inclusion, exclusion criteria, study design and outcome assessments of the included studies.

eTable 4: The deviance information criteria (DIC) and I^2 values of fixed-effects model, random-effects model and their corresponding unrelated means models in network meta-analysis.

eTable 5: Funnel plot for each outcome.

eTable 6: Detailed certainty of evidence for each outcome in league table.

eTable 7: Net plot for each outcome in studies < 1 month follow-up.

eTable 8: Network meta-analysis results of LPS in studies < 1 month follow-up.

eTable 9: Network meta-analysis results of sTSO in studies < 1 month follow-up.

Table 10: Network meta-analysis results of WASO in studies < 1 month follow-up.

eTable 11: Network meta-analysis results of sWASO in studies < 1 month follow-up.

eTable 12: Network meta-analysis results of TST in studies < 1 month follow-up.

eTable 13: Network meta-analysis results of sTST in studies < 1 month follow-up.

eTable 14: Network meta-analysis results of AEs in studies < 1 month follow-up.

eTable 15: Network meta-analysis results of SAEs in studies < 1 month follow-up.

eTable 16: Detailed certainty of evidence for each outcome in studies < 1 month follow-up.

eTable 17: Net plot for each outcome in studies > 3 months follow-up.

eTable 18: Network meta-analysis results of LPS in studies > 3 months follow-up.

eTable 19: Network meta-analysis results of sTSO in studies > 3 months follow-up.

eTable 20: Network meta-analysis results of WASO in studies > 3 months follow-up.

eTable 21: Network meta-analysis results of sWASO in studies > 3 months follow-up.

eTable 22: Network meta-analysis results of sTST in studies > 3 months follow-up.

eTable 23: Network meta-analysis results of AEs in studies > 3 months follow-up.

eTable 24: Network meta-analysis results of SAEs in studies > 3 months follow-up.

eTable 25: Detailed certainty of evidence for each outcome in studies > 3 months follow-up.

eTable 26: Net plot for each outcome in elderly people.

eTable 27: Network meta-analysis results of LPS in elderly people.

eTable 28: Network meta-analysis results of sTSO in elderly people.

eTable 29: Network meta-analysis results of WASO in elderly people.

eTable 30: Network meta-analysis results of sWASO in elderly people.

eTable 31: Network meta-analysis results of sTST in elderly people.

eTable 32: Network meta-analysis results of AEs in elderly people.

eTable 33: Network meta-analysis results of SAEs in elderly people.

eTable 34: Detailed certainty of evidence for each outcome in elderly people.

eTable 35: Net plot for each outcome in double-blinded parallel-group studies.

eTable 36: Network meta-analysis results of LPS in double-blinded parallel-group studies.

eTable 37: Network meta-analysis results of sTSO in double-blinded parallel-group studies.

eTable 38: Network meta-analysis results of WASO in double-blinded parallel-group studies.

eTable 39: Network meta-analysis results of sWASO in double-blinded parallel-group studies.

eTable 40: Network meta-analysis results of sTST in double-blinded parallel-group studies.

eTable 41: Network meta-analysis results of ISI in double-blinded parallel-group studies.

eTable 42: Network meta-analysis results of AEs in double-blinded parallel-group studies.

eTable 43: Network meta-analysis results of SAEs in double-blinded parallel-group studies.
eTable 44: Detailed certainty of evidence for each outcome in double-blinded parallel-group studies.
eTable 45: Network meta-analysis results of sTSO in studies with low bias.
eTable 46: Network meta-analysis results of sWASO in studies with low bias.
eTable 47: Network meta-analysis results of sTST in studies with low bias.
eTable 48: Network meta-analysis results of AEs in studies with low bias.
eTable 49: Network meta-analysis results of SAEs in studies with low bias.
eTable 50: Network meta-analysis results of LPS without 2020 Zammit et al.
eTable 51: Network meta-analysis results of sTSO without 2020 Zammit et al.
eTable 52: Network meta-analysis results of sTST without 2020 Zammit et al.
eTable 53: Network meta-analysis results of WASO without 2020 Zammit et al.
eTable 54: Network meta-analysis results of sWASO without 2020 Zammit et al.
eTable 55: Network meta-analysis results of AEs without 2020 Zammit et al.
eTable 56: Network meta-analysis results of LPS in FDA-approved DORAs doses.
eTable 57: Network meta-analysis results of sTSO in FDA-approved DORAs doses.
eTable 58: Network meta-analysis results of WASO in FDA-approved DORAs doses.
eTable 59: Network meta-analysis results of sWASO in FDA-approved DORAs doses.
eTable 60: Network meta-analysis results of TST in FDA-approved DORAs doses.
eTable 61: Network meta-analysis results of sTST in FDA-approved DORAs doses.
eTable 62: Network meta-analysis results of ISI in FDA-approved DORAs doses.
eTable 63: Network meta-analysis results of AEs in FDA-approved DORAs doses.
eTable 64: Network meta-analysis results of SAEs in FDA-approved DORAs doses.

eFigure 1: Risk of bias
eFigure 2: Convergence diagnostics of the network meta-analysis: LPS.
eFigure 3: Convergence diagnostics of the network meta-analysis: sTSO.
eFigure 4: Convergence diagnostics of the network meta-analysis: WASO.
eFigure 5: Convergence diagnostics of the network meta-analysis: sWASO.
eFigure 6: Convergence diagnostics of the network meta-analysis: TST.
eFigure 7: Convergence diagnostics of the network meta-analysis: sTST.
eFigure 8: Convergence diagnostics of the network meta-analysis: ISI.
eFigure 9: Convergence diagnostics of the network meta-analysis: AEs.
eFigure 10: Convergence diagnostics of the network meta-analysis: SAEs.
eFigure 11: Trace and density of the network meta-analysis: LPS.
eFigure 12: Trace and density of the network meta-analysis: sTSO.
eFigure 13: Trace and density of the network meta-analysis: WASO.
eFigure 14: Trace and density of the network meta-analysis: sWASO.
eFigure 15: Trace and density of the network meta-analysis: TST.
eFigure 16: Trace and density of the network meta-analysis: sTST.
eFigure 17: Trace and density of the network meta-analysis: ISI.
eFigure 18: Trace and density of the network meta-analysis: AEs.
eFigure 19: Trace and density of the network meta-analysis: SAEs.
eFigure 20: Forest plots for the heterogeneity: LPS.
eFigure 21: Forest plots for the heterogeneity: sTSO.
eFigure 22: Forest plots for the heterogeneity: WASO.
eFigure 23: Forest plots for the heterogeneity: sWASO.
eFigure 24: Forest plots for the heterogeneity: TST.
eFigure 25: Forest plots for the heterogeneity: sTST.

eFigure 26: Forest plots for the heterogeneity: ISI.
eFigure 27: Forest plots for the heterogeneity: AEs.
eFigure 28: Forest plots for the inconsistency: LPS.
eFigure 29: Forest plots for the inconsistency: WASO.
eFigure 30: Forest plots for the inconsistency: sTST.
eFigure 31: Forest plots for the inconsistency: AEs.
eFigure 32 Forest plots for the inconsistency: SAEs.

Evaluation of the certainty of evidence and clinical importance using the GRADE approach for each outcome.

We chose "placebo" as reference intervention and to keep the framework minimally contextualised, no effect (a RR of 1.0 and a MD of 0) was set as the threshold. Next, first and second classification of interventions was conducted based on comparison with reference and other interventions. Additionally, we used 'Maybe' to mark the low or very low certain evidence.

Take sTSO as an example, in the first classification, the CrI of suvorexant 10mg, 80mg and daridorexant 25mg crossed the decision threshold, so these interventions remained in the category 0 ("as effective as placebo" group). The CrI of daridorexant 5mg did not cross the decision threshold and showed a worsening effect, and thus it was classified into the category -1 ("even worse than placebo" group). The CrI of others (suvorexant 20mg, 40 mg, lemborexant 5mg, 10mg, daridorexant 10mg and 50mg) did not cross the decision threshold but showed improvement and upgraded to category 1 ("better than placebo" group). In the second classification, the interventions in category 1 should compare with each other. Daridorexant 10mg was found to be less effective than any other interventions in category 1. Therefore, daridorexant 10mg kept in the category 1 ("Among the least effective but better than placebo" group), and others upgraded into category 2. Next, suvorexant 20mg and daridorexant 50mg were found to be less effective than any other interventions in category 2. Therefore, suvorexant 20mg and daridorexant 50mg kept in the category 2 ("Superior to the least effective" group), and others upgraded into category 3. Finally, lemborexant 5mg and 10mg were found to be more effective than suvorexant 40mg. Therefore, lemborexant 5mg and 10mg upgraded into category 4 ("Among the most effective" group) and suvorexant 40mg kept in the category 3 ("Inferior to the most effective" group). All interventions except suvorexant 10mg exhibited moderate to high certainty, and suvorexant 10mg showed low certainty in sTSO. Therefore, only suvorexant 10mg should be marked with "Maybe" and move to "Maybe as effective as placebo" group.

eTable 1: Full search strategy.

PubMed：

Search	Query	Results
#1	"Randomized controlled trial"[Filter]	578,764
#2	"Sleep Initiation and Maintenance Disorders"[Mesh]	16,497
#3	((((((((((((((((((((((((Disorders of Initiating[Title/Abstract] AND Maintaining Sleep[Title/Abstract]) OR (DIMS [Title/Abstract])) OR (Early Awakening[Title/Abstract])) OR (Early Awakening[Title/Abstract])) OR (Nonorganic Insomnia[Title/Abstract])) OR (Insomnia, Nonorganic[Title/Abstract])) OR (Primary Insomnia[Title/Abstract])) OR (Insomnia, Primary[Title/Abstract])) OR (Transient Insomnia[Title/Abstract])) OR (Insomnia, Transient[Title/Abstract])) OR (Rebound Insomnia[Title/Abstract])) OR (Insomnia, Rebound[Title/Abstract])) OR (Secondary Insomnia[Title/Abstract])) OR (Insomnia, Secondary[Title/Abstract])) OR (Sleep Initiation Dysfunction[Title/Abstract])) OR (Dysfunction, Sleep Initiation[Title/Abstract])) OR (Dysfunctions, Sleep Initiation[Title/Abstract])) OR (Sleep Initiation Dysfunctions[Title/Abstract])) OR (Sleeplessness[Title/Abstract])) OR (Insomnia Disorder[Title/Abstract])) OR (Insomnia Disorders[Title/Abstract])) OR (Insomnia[Title/Abstract])) OR (Insomnias[Title/Abstract])) OR (Chronic Insomnia[Title/Abstract])) OR (Insomnia, Chronic[Title/Abstract])) OR (Psychophysiological Insomnia[Title/Abstract])) OR (Insomnia, Psychophysiological[Title/Abstract])	28,292
#4	#2 OR #3	33,131
#5	"Orexin Receptor Antagonists"[Mesh]	502
#6	(((((Orexin Receptor Blockers[Title/Abstract]) OR (Orexin Receptor Antagonist[Title/Abstract])) OR (Antagonist, Orexin Receptor[Title/Abstract])) OR (Receptor Antagonist, Orexin[Title/Abstract])) OR (Dual Orexin Receptor Antagonists[Title/Abstract])) OR (Dual Orexin Receptor Antagonist[Title/Abstract])	686
#7	#5 OR #6	897
#8	"suvorexant"[Supplementary Concept]	192
#9	((((((4-(5-chloro-1,3-benzoxazol-2-yl)-7-methyl-1,4-diazepan-1-yl)(5-methyl-2-(2H-1,2,3-triazol-2-yl)phenyl)methanone[Title/Abstract]) OR (methanone, ((7R)-4-(5-chloro-2-benzoxazolyl)hexahydro-7-methyl-1H-1,4-diazepin-1-yl)(5-methyl-2-(2H-1,2,3-triazol-2-yl)phenyl)-[Title/Abstract])) OR (MK 4305[Title/Abstract])) OR (MK4305[Title/Abstract])) OR (MK-4305[Title/Abstract])) OR (Belsomra[Title/Abstract])	54
#10	#8 OR #9	213
#11	"lemborexant" [Supplementary Concept]	48
#12	((dayvigo[Title/Abstract]) OR ((1R,2S)-2-(((2,4-dimethyl-5-pyrimidinyl)oxy)methyl)-2-(3-fluorophenyl)-N-(5-fluoro-2-pyridinyl)cyclopropanecarboxamide[Title/Abstract])) OR (cyclopropanecarboxamide, 2-(((2,4-dimethyl-5-pyrimidinyl)oxy)methyl)-2-(3-fluorophenyl)-N-(5-fluoro-2-pyridinyl)-, (1R,2S)-[Title/Abstract])	6
#13	#11 OR #12	51

#14	"daridorexant" [Supplementary Concept]	28
#15	(((nemorexant[Title/Abstract]) OR (methanone, ((2S)-2-(6-chloro-7-methyl-1H-benzimidazol-2-yl)-2-methyl-1-pyrrolidinyl)(5-methoxy-2-(2H-1,2,3-traizol-2-yl)phenyl)-[Title/Abstract])) OR (((S[Title/Abstract])-(2-(5-chloro-4-methyl-1H-benzo(d)imidazol-2-yl)-2-methylpyrrolidin-1-yl) (5 methoxy-2-(2H-1,2,3-triazol-2-yl)phenyl)methanone))) OR (ACT-541468[Title/Abstract])	13
#16	#14 OR #15	31
#17	#7 OR #10 OR #13 OR #16	982
#18	#4 AND #17	370
#19	#1 AND #18	**71**

Embase：

Search	Query	Results
#1	''insomnia'/exp	81,257
#2	'sleep initiation and maintenance disorder':ab,ti,kw	18
#3	'disorders of initiating and maintaining sleep':ab,ti,kw	114
#4	'dims':ab,ti,kw	508
#5	'early awakening':ab,ti,kw	229
#6	'awakening, early':ab,ti,kw	31
#7	'nonorganic insomnia':ab,ti,kw	39
#8	'insomnia, nonorganic':ab,ti,kw	1
#9	'primary insomnia':ab,ti,kw	1,670
#10	'insomnia, primary':ab,ti,kw	25
#11	'transient insomnia':ab,ti,kw	137
#12	'insomnia, transient':ab,ti,kw	7
#13	'rebound insomnia':ab,ti,kw	321
#14	'insomnia, rebound':ab,ti,kw	8
#15	'secondary insomnia':ab,ti,kw	117
#16	'insomnia, secondary':ab,ti,kw	76
#17	'sleep initiation dysfunction':ab,ti,kw	4
#18	'dysfunction, sleep initiation':ab,ti,kw	0
#19	'dysfunctions, sleep initiation':ab,ti,kw	0
#20	'sleep initiation dysfunctions':ab,ti,kw	0
#21	'sleeplessness':ab,ti,kw	1,165
#22	'insomnia disorder':ab,ti,kw	1245
#23	'insomnia disorders':ab,ti,kw	98
#24	'insomnias':ab,ti,kw	233
#25	'chronic insomnia':ab,ti,kw	2,262
#26	'insomnia, chronic':ab,ti,kw	68
#27	'psychophysiological insomnia':ab,ti,kw	225
#28	'insomnia, psychophysiological':ab,ti,kw	5
#29	#1 OR #2 OR #3 OR #4 OR #5 OR #6 OR #7 OR #8 OR #9 OR #10 OR #11 OR #12 OR #13 OR #14 OR #15 OR #16 OR #17 OR #18 OR #19 OR #20 OR #21 OR #22 OR #23 OR #24 OR #25 OR #26 OR #27 OR #28	82,572
#30	'orexin receptor antagonist'/exp	2226

#31	'orexin receptor antagonists':ab,ti,kw	309
#32	'orexin receptor blockers':ab,ti,kw	0
#33	'antagonist, orexin receptor':ab,ti,kw	0
#34	'receptor antagonist, orexin':ab,ti,kw	1
#35	'dual orexin receptor antagonists':ab,ti,kw	126
#36	'dual orexin receptor antagonist':ab,ti,kw	403
#37	#30 OR #31 OR #32 OR #33 OR #34 OR #35 OR #36	2341
#38	'suvorexant'/exp	789
#39	'(4-(5-chloro-1,3-benzoxazol-2-yl)-7-methyl-1,4-diazepan-1-yl)(5-methyl-2-(2h-1,2,3-triazol-2-yl)phenyl)methanone':ab,ti,kw	1
#40	'methanone, ((7r)-4-(5-chloro-2-benzoxazolyl)hexahydro-7-methyl-1h-1,4-diazepin-1-yl)(5-methyl-2-(2h-1,2,3-triazol-2-yl)phenyl)-':ab,ti,kw	0
#41	'mk 4305':ab,ti,kw	42
#42	'mk4305':ab,ti,kw	42
#43	'mk-4305':ab,ti,kw	42
#44	'belsomra':ab,ti,kw	43
#45	#38 OR #39 OR #40 OR #41 OR #42 OR #43 OR #44	802
#46	'lemborexant'/exp	247
#47	'dayvigo':ab,kw,ti	5
#48	'(1R,2S)-2-(((2,4-dimethyl-5-pyrimidinyl)oxy)methyl)-2-(3-fluorophenyl)-N-(5-fluoro-2-pyridinyl)cyclopropanecarboxamide':ab,kw,ti	0
#49	'cyclopropanecarboxamide, 2-(((2,4-dimethyl-5-pyrimidinyl)oxy)methyl)-2-(3-fluorophenyl)-n-(5-fluoro-2-pyridinyl)-, (1r,2s)-':ab,kw,ti	0
#50	'daridorexant'/exp	115
#51	'nemorexant':ab,kw,ti	0
#52	'methanone, ((2s)-2-(6-chloro-7-methyl-1h-benzimidazol-2-yl)-2-methyl-1-pyrrolidinyl)(5-methoxy-2-(2h-1,2,3-traizol-2-yl)phenyl)-':ab,kw,ti	0
#53	'((s)-(2-(5-chloro-4-methyl-1h-benzo(d)imidazol-2-yl)-2-methylpyrrolidin-1-yl) (5 methoxy-2-(2h-1,2,3-triazol-2-yl)phenyl)methanone)':ab,kw,ti	0
#54	'act-541468':ab,kw,ti	29
#55	#46 OR #47 OR #48 OR #49 OR #50 OR #51 OR #52 OR #53 OR #54	348
#56	#37 OR #45 OR #55	2344
#57	#29 AND #56	873
#58	'random':ab,ti OR 'control':ab,ti OR 'double-blind':ab,ti	4,373,379
#59	#57 AND #58	**283**

Cochrane:

Search	Query	Results
#1	MeSH descriptor: [Sleep Initiation and Maintenance Disorders] explode all trees	2,863
#2	(Disorders of Initiating and Maintaining Sleep):ti,ab,kw OR (DIMS):ti,ab,kw OR (Early Awakening):ti,ab,kw OR (Awakening, Early):ti,ab,kw OR (Nonorganic Insomnia):ti,ab,kw OR (Insomnia, Nonorganic):ti,ab,kw OR(Primary Insomnia):ti,ab,kw OR (Insomnia, Primary):ti,ab,kw OR (Transient Insomnia):ti,ab,kw OR (Insomnia, Transient):ti,ab,kw OR (Rebound Insomnia):ti,ab,kw OR (Insomnia, Rebound):ti,ab,kw OR (Secondary Insomnia):ti,ab,kw OR (Insomnia, Secondary):ti,ab,kw OR (Sleep Initiation Dysfunction):ti,ab,kw OR (Dysfunction, Sleep Initiation):ti,ab,kw OR (Dysfunctions, Sleep Initiation):ti,ab,kw OR (Sleep Initiation Dysfunctions):ti,ab,kw OR (Sleeplessness):ti,ab,kw OR	13194

	(Insomnia Disorder):ti,ab,kw OR (Insomnia Disorders):ti,ab,kw OR (Insomnia):ti,ab,kw OR (Insomnias):ti,ab,kw OR (Chronic Insomnia):ti,ab,kw OR (Insomnia, Chronic):ti,ab,kw OR (Psychophysiological Insomnia):ti,ab,kw OR (Insomnia, Psychophysiological):ti,ab,kw	
#3	#1 OR #2	13718
#4	MeSH descriptor: [Orexin Receptor Antagonists] explode all trees	73
#5	(Orexin Receptor Blockers):ti,ab,kw OR (Orexin Receptor Antagonist):ti,ab,kw OR (Antagonist, Orexin Receptor):ti,ab,kw OR (Receptor Antagonist, Orexin):ti,ab,kw OR (Dual Orexin Receptor Antagonists):ti,ab,kw OR (Dual Orexin Receptor Antagonist):ti,ab,kw	297
#6	#4 OR #5	301
#7	(suvorexant):ti,ab,kw	207
#8	(mk 4305):ab,ti,kw OR (mk4305):ab,ti,kw OR (mk-4305):ab,ti,kw OR (belsomra):ab,ti,kw	33
#9	7 OR #8	213
#10	(lemborexant):ab,ti,kw OR (dayvigo):ab,ti,kw	135
#11	(daridorexant):ab,kw,ti OR (act-541468):ab,kw,ti	90
#12	#6 OR #9 OR #10 OR #11	481
#13	#3 AND #12	**363**

Clinicaltrials.gov：

Search	Query	Results
#1	Status: All studies, condition or disease: Insomnia, and other terms: Orexin Receptor Antagonist, Study type: interventional (Clinical Trial)	38
#2	Status: All studies, condition or disease: Insomnia, and other terms: suvorexant, Study type: interventional (Clinical Trial)	32
#3	Status: All studies, condition or disease: Insomnia, and other terms: lemborexant, Study type: interventional (Clinical Trial)	9
#4	Status: All studies, condition or disease: Insomnia, and other terms: daridorexant, Study type: interventional (Clinical Trial)	4
**Total**		**83**

eTable 2: Appendix of detailed excluded studies.

(1). Conference abstract (n=82)

1. Cheng J, Yardley J, Pinner K, Moline M. Efficacy and safety of lemborexant in female subjects of perimenopausal age with insomnia disorder. Sleep. 2020;43(SUPPL 1):A184.
2. Connor K, Budd K, Snavely D, Liu K, Hutzelmann J, Benca R, et al. Efficacy and safety of suvorexant, an orexin receptor antagonist, in patients with primary insomnia: A 3-month phase 3 trial (trial #1). Journal of Sleep Research. 2012;21:97.
3. Connor KM, Matzura-Wolfe D, Zhang Y, Ivgy-May N, Snavely D, Snyder E, et al. Safety of suvorexant, an orexin receptor antagonist, in patients with primary insomnia: Integrated phase 3 results. Sleep. 2013;36:A223.
4. Dauvilliers Y, Zammit G, Fietze I, Mayleben D, Kinter DS, Pain S, et al. Daridorexant (Act-541468), a new dual orexin receptor antagonist, for the treatment of insomnia disorder: a randomized, double-blind, placebo-controlled, active-reference phase 2 study. Sleep Medicine. 2019;64:S85.
5. Dauvilliers Y, Zammit G, Fietze I, Mayleben D, Kinter DS, Pain S, et al. A novel dual orexin receptor antagonist (act-541468) to treat insomnia: A randomized, double-blind, placebo-controlled, activere ference phase 2 study. Sleep. 2019;42:A152-A3.
6. Drake C, Yardley J, Pinner K, Perdomo C, Moline M. Subject-reported perception of long-term effectiveness of lemborexant versus placebo in nonelderly and elderly subgroups. American Journal of Geriatric Psychiatry. 2020;28(4):S133-S4.
7. Drake C, Yardley J, Pinner K, Perdomo C, Moline M. Long-term perception of medication effectiveness in subjects receiving lemborexant for up to 12 months. Sleep. 2021;44(SUPPL 2):A135.
8. Drake C, Yardley J, Pinner K, Perdomo C, Moline M. Evaluation of Long-term Perception of Medication Effectiveness: Results from Subjects Receiving Lemborexant for up to 12 Months. Sleep Medicine. 2022;100:S120.
9. Drake C, Yardley J, Pinner K, Perdomo C, Moline M. Self-reported Perceptions of Medication Effectiveness in Subjects Receiving Lemborexant for up to 12 Months. Neurology. 2022;98(18 SUPPL).
10. Fietze I, Bassetti C, Mayleben D, Gimona A, Pain S, Kinter DS. Daridorexant is safe and improves both sleep and daytime functioning in elderly patients with insomnia. Sleep. 2021;44(SUPPL 2):A138-A9.
11. Fietze I, Bassetti C, Mayleben D, Gimona A, Pain S, Seboek Kinter D. Effects of daridorexant on sleep and daytime functioning in older patients with insomnia disorder. Sleep Medicine. 2022;100:S117-S8.
12. Herring W, Connor K, Ivgy-May N, Snavely D, Snyder E, Michelson D. Clinical profile of suvorexant over 3 months in elderly patients with insomnia: Subgroup analysis of phase-3 data. Neurology. 2015;84.
13. Herring W, Connor K, Snavely D, Zhang Y, Snyder E, Lines C, et al. Clinical profile of suvorexant for the treatment of insomnia over 3 months in men and women: Gender subgroup analysis of pooled phase-3 data. Neurology. 2016;86(16).
14. Herring W, Connor KM, Ivgy-May N, Snavely D, Snyder E, Michelson D. Clinical profile of suvorexant over 3 months in elderly patients with insomnia: Subgroup analysis of phase-3 data. Sleep. 2014;37:A202.
15. Herring W, Connor KM, Ivgy-May N, Snavely D, Snyder E, Michelson D. Clinical profile of suvorexant 20/15mg in phase-3 trials of patients with insomnia. Sleep. 2014;37:A202.
16. Herring W, Connor KM, Snavely D, Zhang Y, Snyder E, Lines C, et al. Clinical profile of suvorexant for the treatment of insomnia over 3 months in men and women: Gender subgroup analysis of pooled Phase-3 data. Sleep. 2015;38:A232.

17. Herring W, Connor KM, Snyder E, Snavely D, Lines C, Michelson D. Effects of suvorexant on the insomnia severity index in patients with insomnia: Analysis of pooled Phase-3 data. Sleep. 2015;38:A235.
18. Herring W, Snyder E, Ivgy-May N, Connor K, Snavely D, Michelson D. Clinical profile of the orexin receptor antagonist suvorexant for the treatment of insomnia in phase-3 clinical trials. Neurology. 2014;82(10).
19. Herring W, Snyder E, Snavely D, Lines C, Michelson D. Effects of suvorexant on the insomnia severity index in patients with insomnia: Analysis of pooled phase-3 data. Neurology. 2016;86(16).
20. Herring WJ. Efficacy and safety of suvorexant, an orexin receptor antagonist, in patients with primary insomnia: Results from three phase 3 trials. Neuropsychopharmacology. 2012;38:S76-S7.
21. Herring WJ. Clinical profile of suvorexant (mk-4305), an orexin receptor antagonist, in patients with primary insomnia: Results from phase-3 trials. Biological Psychiatry. 2014;75(9):35S.
22. Herring WJ, Budd K, Hutzelmann J, Snyder E, Snavely D, Liu K, et al. Efficacy and tolerability of the dual orexin receptor antagonist MK-4305 in patients with primary insomnia: A randomized, controlled, adaptive crossover polysomnography study. Journal of Sleep Research. 2010;19:44-5.
23. Herring WJ, Budd KS, Hutzelmann J, Snyder E, Snavely D, Liu K, et al. Efficacy and tolerability of the dual orexin receptor antagonist mk-4305 in patients with primary insomnia: Randomized, controlled, adaptive crossover polysomnography study. Sleep. 2010;33:A199.
24. Herring WJ, Ivgy-May N, Connor KM, Wu M, Liu K, Snyder E, et al. Effect of suvorexant, an orexin receptor antagonist, on patient-reported outcomes in patients with primary insomnia: Integrated results from two phase-3 trials. Sleep. 2013;36:A223.
25. Herring WJ, Snyder E, Paradis E, Hutzelmann J, Liu MC, Snavely D, et al. Suvorexant, an orexin receptor antagonist, in preventing symptom return in patients with insomnia after 1 year of treatment: A randomised, double-blind, placebo-controlled study. Journal of Sleep Research. 2012;21:351.
26. Herring WJ, Snyder E, Paradis E, Liu M, Snavely D, Roth T, et al. Long term safety and efficacy of suvorexant in patients with primary insomnia. Sleep. 2012;35:A217.
27. Herring WJ, Snyder E, Tao P, Svetnik V, Lines C. Performance of the patient global impressions - Severity scale as a selfreported patient assessment of insomnia severity. Neurology. 2018;90(15).
28. Hisada S, Kikuchi M, Takahashi K, Shimamoto T, Connor KM, Herring WJ, et al. Efficacy and safety of suvorexant, an orexin receptor antagonist, in patients with primary insomnia: A 3-month phase III study (P028). Sleep. 2013;36:A224.
29. Ivgy-May N, Leibensperger H, Froman S, Hutzelmann J, Snavely D, Snyder E, et al. Efficacy and safety of suvorexant, an orexin receptor antagonist, in patients with primary insomnia: A 3-month phase 3 trial (trial #2). Journal of Sleep Research. 2012;21:351-2.
30. Ivgy-May N, Snavely D, Minigh J, Wu M, Hutzelmann J, Snyder E, et al. Efficacy of suvorexant, an orexin receptor antagonist, in patients with primary insomnia: Integrated results from 2 similarly designed phase 3 trials. Sleep. 2013;36:A192.
31. Joseph Herring W. Clinical profile of suvorexant in phase-3 trials. Journal of Sleep Research. 2018;27.
32. Kärppä M, Moline M, Yardley J, Pinner K, Perdomo C, Filippov G, et al. Lemborexant treatment for insomnia: 6-month safety. Sleep. 2019;42:A149-A50.
33. Kinter DS, Mayleben D, Fietze I, Zammit G, Bassetti C, Leger D, et al. Daridorexant is efficacious in improving sleep as well as daytime functioning in insomnia patients. Neuropsychopharmacology. 2020;45:136-7.

34. Kunz D, Beneš H, García-Borreguero D, Dauvilliers Y, Plazzi G, Sassi-Sayadi M, et al. Long-term safety and efficacy of daridorexant in patients with insomnia disorder. Sleep Medicine. 2022;100:S130.
35. Moline M, Inoue Y, Pinner K, Perdomo C, Filippov G, Kubota N, et al. Efficacy and Safety of Lemborexant Across 12 Months in Elderly Subjects With Insomnia Disorder. Journal of the National Medical Association. 2020;112(5):S27.
36. Moline M, Inoue Y, Pinner K, Perdomo C, Filippov G, Kubota N, et al. Long-term efficacy and safety of lemborexant in elderly adults with insomnia disorder: Results from sunrise-2. Sleep. 2020;43(SUPPL 1):A182.
37. Moline M, Karppa M, Yardley J, Kumar D, Pinner K, Perdomo C, et al. 165 Impact of Lemborexant on Insomnia Disease Severity and Fatigue: results from the 6-Month Placebo-Controlled Period of the Phase 3 SUNRISE-2 Study. CNS spectrums. 2020;25(2):305-6.
38. Moline M, Kärppä M, Yardley J, Kumar D, Pinner K, Perdomo C, et al. Impact of lemborexant on insomnia disease severity and fatigue: Results from the 6-month placebo-controlled period of the phase 3 SUNRISE-2 study. CNS Spectrums. 2020;25(2):305-6.
39. Moline M, Murphy P, Yardley J, Kumar D, Pinner K, Perdomo C, et al. Efficacy and tolerability of lemborexant in female and male subjects with insomnia. Sleep. 2019;42:S150.
40. Moline M, Murphy PJ, Giorgi L, Bradshaw K, Berkman L, Yardley J, et al. Dual orexin receptor antagonist E2006 shows efficacy on sleep initiation and maintenance on sleep diary measures in phase 2 study. Sleep. 2015;38:A222.
41. Moline M, Pinner K, Cheng J, Perdomo C, Kumar D, Zammit G, et al. Effect of lemborexant compared with placebo and zolpidem extended release on sleep architecture in older adults with insomnia disorder. Sleep Medicine. 2019;64:S437.
42. Moline M, Yardley J, Pinner K, Kumar D, Bsharat M, Kärppä M, et al. Efficacy and safety of lemborexant in elderly subjects with insomnia: Results of a pooled analysis of two phase 3 trials. Journal of the American Geriatrics Society. 2020;68(SUPPL 1):S56.
43. Moline M, Yardley J, Pinner K, Kumar D, Bsharat M, Kärppä M, et al. Efficacy and safety of lemborexant in elderly subjects with insomnia disorder: Pooled analyses from sunrise-1 and sunrise-2. Neurology. 2020;94(15).
44. Murphy PJ, Bradshaw K, Berkman L, Moline M, Giorgi L, Satlin A. Dual orexin receptor antagonist E2006 shows equivalent efficacy in men and women in Phase 2 study. Sleep. 2015;38:A233-A4.
45. Murphy PJ, Moline M, Pinner K, Hong Q, Yardley J, Zammit G, et al. Effects of lemborexant on sleep architecture in subjects with insomnia disorder. Sleep. 2016;39:A207.
46. Pinner K, Hong Q, Murphy P, Moline M, Dhadda S, Yardley J, et al. Lemborexant effects on sleep maintenance in the second half of the night. Journal of the American Geriatrics Society. 2017;65:S47.
47. Pinner K, Hong Q, Murphy P, Moline M, Yardley J, Zammit G, et al. Effects of lemborexant on sleep maintenance in the latter half of the night. Sleep. 2016;39:A208.
48. Rosenberg R, Filippov G, LoPresti A, Kumar D, Moline M. Safety of lemborexant in elderly subjects with insomnia: Results from sunrise 1. Journal of the American Geriatrics Society. 2019;67:S218.
49. Rosenberg R, Filippov G, LoPresti A, Kumar D, Murphy P, Moline M. Safety of lemborexant in elderly subjects with insomnia: results from a phase 3 study (SUNRISE 1). American Journal of Geriatric Psychiatry. 2019;27(3):S155-S6.
50. Rosenberg R, Kumar D, Pinner K, Perdomo C, Moline M. Impact of lemborexant on fatigue severity in subjects with clinically significant levels of fatigue at baseline. Sleep. 2020;43(SUPPL 1):A184-A5.

51. Rosenberg R, Murphy P, Chou C, Dhadda S, Zammit G, Moline M. Comparison of lemborexant with zolpidem extended release and placebo: topline results from a phase 3 study in subjects 55 years and older with insomnia. Journal of Sleep Research. 2018;27:165.
52. Rosenberg R, Zammit G, Yardley J, Pinner K, Kumar D, Perdomo C, et al. Patient-reported sleep onset and sleep maintenance: pooled responder analyses of lemborexant phase 3 studies. Sleep Medicine. 2019;64:S326.
53. Rosenberg R, Zammit G, Yardley J, Pinner K, Kumar D, Perdomo C, et al. Pooled analyses of patient-reported sleep onset and maintenance from two phase 3 studies of lemborexant. CNS Spectrums. 2020;25(2):304-5.
54. Roth T, Rosenberg R, Yardley J, Kumar D, Pinner K, Perdomo C, et al. The impact of lemborexant treatment on insomnia disease severity: results from a pooled analysis of two phase 3 studies. Sleep Medicine. 2019;64:S325-S6.
55. Roth T, Zammit G, Kumar D, Pappadopulos E, Moline M. Lemborexant versus Zolpidem: An Assessment of Wake Bouts in Adults with Insomnia. Sleep Medicine. 2022;100:S128-S9.
56. Roth T, Zammit G, Mignot E, Leger D, Bassetti C, Pain S, et al. Daridorexant, a novel dual orexin receptor antagonist, delivers significant improvement in sleep parameters and daytime function for patients with insomnia disorder. Journal of Sleep Research. 2020;29(SUPPL 1).
57. Satlin A, Murphy P, Moline M, Orford C, Giorgi L, Bradshaw K. Dual orexin receptor antagonist E2006 shows efficacy on sleep initiation and maintenance in phase 2 study. Neuropsychopharmacology. 2014;39:S336-S7.
58. Snyder E, Ma J, Svetnik V, Connor KM, Lines C, Michelson D, et al. Effects of suvorexant on sleep architecture in patients with insomnia: Analysis of pooled Phase-3 data. Sleep. 2015;38:A232.
59. Yardley J, Inoue Y, Pinner K, Perdomo C, Atkins N, Filippov G, et al. Efficacy and Safety of Lemborexant in Subjects Previously Randomized to Placebo for 6 Months. Journal of the National Medical Association. 2020;112(5):S27.
60. Yardley J, Inoue Y, Pinner K, Perdomo C, Filippov G, Kubota N, et al. Effectiveness and safety of lemborexant in subjects previously treated with placebo for 6 months in sunrise-2. Sleep. 2020;43(SUPPL 1):A181-A2.
61. Yardley J, Kärppä M, Inoue Y, Pinner K, Perdomo C, Filippov G, et al. Long-term effectiveness and safety of lemborexant in adults with insomnia disorder: 12-month results from sunrise-2. Sleep Medicine. 2019;64:S263-S4.
62. Yardley J, Kärppä M, Inoue Y, Pinner K, Perdomo C, Filippov G, et al. Effectiveness and safety of lemborexant over 12 months in adults with insomnia disorder: Longterm results from the phase 3 sunrise-2 study. Neurology. 2020;94(15).
63. Yardley J, Moline M, Pinner K, Perdomo C, Fillipov G, Kärppä M. Long-term effect of lemborexant on fatigue in subjects with insomnia disorder: patient-reported outcome from the 6-month placebo-controlled treatment period of the phase 3 study sunrise-2. Sleep Medicine. 2019;64:S264.
64. Yardley J, Pinner K, Moline M, Perdomo C. Impact of lemborexant on insomnia disease severity and fatigue in elderly subjects with insomnia from SUNRISE-2. American Journal of Geriatric Psychiatry. 2020;28(4):S134-S5.
65. Zammit G, Dauvilliers Y, Pain S, Kinter DS, Kunz D. Act-541468, a dual orexin receptor antagonist, for the treatment of insomnia disorder: A randomized, double-blind, placebocontrolled, 5-period, 5-treatment crossover dose-response phase 2 study in the elderly. Sleep. 2019;42:A165.
66. Zammit G, Dauvilliers Y, Pain S, Sebök Kinter D, Kunz D. Daridorexant (ACT-541468), a new dual orexin receptor antagonist, for the treatment of insomnia disorder in the elderly: results from a randomized, double-blind, placebo-controlled, 5-period, 5-treatment crossover dose-response phase 2 study. Sleep Medicine. 2019;64:S437-S8.

67. Zammit G, Mayleben D, Fietze I, Pain S, Gimona A, Seboek Kinter D, et al. Effects of daridorexant on total sleep time (TST) and sleep stage proportions in patients with insomnia disorder. Sleep Medicine. 2022;100:S118.
68. Zammit G, Mayleben D, Fietze I, Pain S, Seboek Kinter D, Gimona A, et al. Daridorexant improves total sleep time (TST) in insomnia patients without altering the proportion of sleep stages. Sleep. 2021;44(SUPPL 2):A137.
69. Zammit G, Mayleben D, Kumar D, Moline M. Efficacy of lemborexant vs zolpidem extended release and placebo in elderly subjects with insomnia: Results from sunrise 1. Journal of the American Geriatrics Society. 2019;67:S51-S2.
70. Zammit G, Mayleben D, Kumar D, Moline M. Efficacy and safety of lemborexant vs zolpidem extended release and placebo in elderly subjects with insomnia: Results from sunrise-1. Neurology. 2020;94(15).
71. Zammit G, Mayleben D, Kumar D, Murphy P, Moline M. Efficacy of lemborexant compared with zolpidem extended release and placebo in elderly subjects with insomnia: results from a phase 3 study (SUNRISE 1). American Journal of Geriatric Psychiatry. 2019;27(3):S154-S5.
72. Zammit G, Seboek Kinter D, Bassetti C, Leger D, Hermann V, Pain S, et al. Daridorexant (ACT-541468), a dual orexin receptor antagonist for the treatment of insomnia disorder: Double blind, randomized, phase 3 studies for efficacy and safety in adult and elderly patients. Sleep. 2020;43(SUPPL 1):A199-A200.
73. Zammit G, Yardley J, Pinner K, Moline M. Sleep onset and sleep maintenance responder profiles over 12 months of treatment with lemborexant: Results from sunrise-2. Sleep. 2020;43(SUPPL 1):A184.
74. Lenderking W, Savva Y, Meier G, Chabot I, Moline M. Utility associated with response to insomnia treatment in SUNRISE-1. Journal of managed care and specialty pharmacy. 2021;27(10-B SUPPL):S72-.
75. Murphy PJ, Giorgi L, Oxford C. Preliminary efficacy of E2006, a novel dual orexin receptor antagonist for the treatment of insomnia disorder. Sleep. 2014;37:A205-A6.
76. Citrome L, Juday T, Atkins N, Frech F, Malhotra M. Assessing Lemborexant Efficacy and Safety in the Treatment of Insomnia. Journal of the National Medical Association. 2020;112(5):S34-.
77. Ikeda S, Azuma M, Fujimoto K, Shibahara H, Inoue S, Moline M, et al. PMH8 EQ-5D Analysis in Patients with Insomnia: change of Quality of Life in Lemborexant Phase 3 Trial Sunrise 1. Value in health. 2020;23:S585-.
78. Kent J, Van Der Ark P, Van Hove I, Moyer J, Luthringer R, Van Nueten L, et al. Selective orexin-2 receptor antagonism improves mood in major depresive disorder: a randomized, placebo-controlled, double-blind study. Neuropsychopharmacology. 2016;41:S502-S3.
79. Moline M, Karppa M, Yardley J, Kumar D, Pinner K, Perdomo C, et al. Impact of lemborexant treatment on insomnia disease severity and fatigue: results from the 12-month phase 3 study sunrise-2. Neurology. 2020;94(15).
80. Moline M, Murphy P, Pinner K, Cheng J, Perdomo C, Kumar D, et al. Effect of lemborexant on sleep architecture in older adults with insomnia disorder. Sleep. 2019;42:A150-.
81. Moline M, Zee P, Kumar D, Pappadopulos E, Malhotra M. Effect of lemborexant on early morning awakening in subjects with severe problems with waking too early. Sleep. 2022;45(SUPPL 1):A199-A200.
82. Roth T, Rosenberg R, Murphy P, Yardley J, Kumar D, Pinner K, et al. Lemborexant treatment for insomnia in phase 3: impact on disease severity. Sleep. 2019;42:A151-.

(2). Post-hoc analysis (n=26)

1. Roth T, Rosenberg R, Morin CM, Yardley J, Pinner K, Perdomo C, et al. Impact of lemborexant treatment on insomnia severity: analyses from a 12-month study of adults with insomnia disorder. Sleep Med. 2022;90:249-57.

2. Dash A, Pinner K, Inoue Y, Hayashida K, Lim SC, Yun CH, et al. Efficacy and safety of lemborexant over 12 months in Asian adults with insomnia disorder. Sleep Medicine: X. 2022;4.

3. Herring WJ, Connor KM, Snyder E, Snavely DB, Morin CM, Lines C, et al. Effects of suvorexant on the Insomnia Severity Index in patients with insomnia: analysis of pooled phase 3 data. Sleep Medicine. 2019;56:219-23.

4. Herring WJ, Connor KM, Snyder E, Snavely DB, Zhang Y, Hutzelmann J, et al. Suvorexant in patients with insomnia: Pooled analyses of three-month data from phase-3 randomized controlled clinical trials. Journal of Clinical Sleep Medicine. 2016;12(9):1215-25.

5*. Herring WJ, Connor KM, Snyder E, Snavely DB, Zhang Y, Hutzelmann J, et al. Suvorexant in Elderly Patients with Insomnia: Pooled Analyses of Data from Phase III Randomized Controlled Clinical Trials. American Journal of Geriatric Psychiatry. 2017;25(7):791-802.

6. Herring WJ, Connor KM, Snyder E, Snavely DB, Zhang Y, Hutzelmann J, et al. Clinical profile of suvorexant for the treatment of insomnia over 3 months in women and men: subgroup analysis of pooled phase-3 data. Psychopharmacology. 2017;234(11):1703-11.

7. Inoue Y, Watanabe T, Takashima S, Takase T, Ishikawa K, Kubota N, et al. Efficacy and safety of lemborexant in adults with insomnia: Comparing Japanese and non-Japanese subgroups from the global, phase 3, randomized, double-blind, placebo-controlled SUNRISE 2 study. Journal of Clinical Sleep Medicine. 2021;17(5):1067-74.

8. Moline M, Zammit G, Cheng JY, Perdomo C, Kumar D, Mayleben D. Comparison of the effect of lemborexant with placebo and zolpidem tartrate extended release on sleep architecture in older adults with insomnia disorder. Journal of Clinical Sleep Medicine. 2021;17(6):1167-74.

9. Snyder E, Ma J, Svetnik V, Connor KM, Lines C, Michelson D, et al. Effects of suvorexant on sleep architecture and power spectral profile in patients with insomnia: Analysis of pooled phase 3 data. Sleep Medicine. 2016;19:93-100.

10. Svetnik V, Snyder ES, Tao P, Scammell TE, Roth T, Lines C, et al. Insight into reduction of wakefulness by suvorexant in patients with insomnia: analysis of wake bouts. Sleep. 2018;41(1).

11*. Fietze I, Bassetti CLA, Mayleben DW, Pain S, Seboek Kinter D, McCall WV. Efficacy and Safety of Daridorexant in Older and Younger Adults with Insomnia Disorder: A Secondary Analysis of a Randomised Placebo-Controlled Trial. Drugs and Aging. 2022.

12. Culpepper L, Krystal AD, Pinner K, Moline M. Post Hoc Analysis of the Impact of Lemborexant on Patient- Reported Sleep and Insomnia Severity in Adults with Insomnia and Depression Histories. CNS Spectrums. 2022;27(2):243.

13. Dauvilliers Y, Beneš H, García-Borreguero D, Kunz D, Plazzi G, Thein S, et al. Efficacy of long-term treatment with daridorexant in patients with insomnia disorder on sleep and daytime functioning: a post-hoc analysis. Sleep Medicine. 2022;100:S119.

14. Di Marco T, Scammell T, Meinel M, Kinter DS, Zammit G, Dauvilliers Y. Effects of daridorexant and zolpidem on the distribution of wakefulness throughout the night in adults with insomnia: Exploratory analysis of data from an international, randomized, double-blind, placebo-controlled phase 2 trial. Neuropsychopharmacology. 2021;46:386.

15. Di Marco T, Scammell TE, Meinel M, Seboek Kinter D, Zammit G, Dauvilliers Y. Effects of daridorexant and zolpidem on night wakefulness in adults with insomnia: exploratory analysis from a randomized, double-blind, placebo-controlled phase 2 trial. Sleep Medicine. 2022;100:S117.

16. Edinger J, Krystal A, Kumar D, Pappadopulos E, Lundwall C, Moline M. Lemborexant treatment of older adults with insomnia and objective short sleep: rates of response and remission. Sleep. 2022;45(SUPPL 1):A200-A1.

17. Herring WJ, Snyder E, Snavely D, Lines C, Michelson D, Swartz J. Effects of suvorexant on the insomnia severity index in patients with insomnia: Analysis of pooled phase-3 data. Sleep Medicine. 2017;40:e322.

18. Jain R, Chepke C, Pinner K, Yardley J, Moline M, Atkins N, et al. Effects of lemborexant on fatigue severity and subjective sleep outcomes in older adults with insomnia and clinically significant fatigue. Journal of the American Geriatrics Society. 2021;69(SUPPL 1):S86-S7.

19. Jain R, Chepke C, Pinner K, Yardley J, Moline M, Malhotra M. Effect of Lemborexant Treatment on Fatigue Severity and Sleep Outcomes in Older Adults With Clinically Significant Fatigue at Baseline. Sleep Medicine. 2022;100:S116.

20. Kinter DS, Parrino L, Pain S, Krystal A. Effect of daridorexant on sleep macro-architecture by quarter of the night in patients with insomnia: Exploratory analysis of data from an international, randomized, double-blind placebo-controlled phase 3 trial. Neuropsychopharmacology. 2021;46:385-6.

21. Krystal A, Edinger J, Kumar D, Pappadopulos E, Malhotra M, Moline M. Effect of lemborexant treatment on polysomnographic sleep measures in older adults with insomnia and objective short sleep. Sleep. 2022;45(SUPPL 1):A200.

22. Moline M, Inoue Y, Kubota N, Pinner K, Perdomo C, Yardley J. Impact of intrinsic factors on efficacy of lemborexant: Subgroup analyses of sunrise-2. Sleep. 2020;43(SUPPL 1):A186.

23. Roth T, Moline M, Pinner K, Yardley J, Pappadopulos E, Malhotra M. Subjective sleep outcomes with lemborexant among subjects with insomnia and clinically meaningful decreases on the insomnia severity index. Sleep. 2022;45(SUPPL 1):A195-A6.

24. Zee PC, Murphy P, Yardley J, Pinner K, Kumar D, Perdomo C, et al. Patient-reported sleep onset and sleep maintenance: Pooled analyses of lemborexant phase 3 studies. Sleep. 2019;42:A150-A1.

25. Chepke C, Jain R, Rosenberg R, Moline M, Yardley J, Pinner K, et al. Improvement in fatigue and sleep measures with the dual orexin receptor antagonist lemborexant in adults with insomnia disorder. Postgraduate medicine. 2022;134(3):316-25.

26. Rosenberg R, Zammit G, Yardley J, Pinner K, Perdomo C, Moline M, et al. 164 Pooled Analyses of Patient-Reported Sleep Onset and Maintenance from Two Phase 3 Studies of Lemborexant. CNS spectrums. 2020;25(2):304-5.

*Data from these studies were only used to evaluate subgroup analysis, and were not included in the main study results.

(3). Protocol (n=9)

1. Euctr BE. A Phase III, Multicenter, Randomized, Double-Blind, Placebo-Controlled, Parallel-Group, Long Term Safety Study of MK-4305 in Patients with Primary Insomnia. - Safety Study of MK-4305 in Patients with Primary Insomnia. https://trialsearchwhoint/Trial2aspx?TrialID=EUCTR2009-015565-31-BE. 2009.

2. Euctr DE. Lemborexant for the treatment of insomnia disorder in older individuals. https://trialsearchwhoint/Trial2aspx?TrialID=EUCTR2015-004347-39-DE. 2016.

3. Euctr GB. A Phase IIb, Multicenter, Randomized, Double-Blind Placebo-Controlled, 2-period adaptive Crossover Polysomnography Study to Evaluate the Safety and Efficacy of MK-4305 in Patients With Primary Insomnia. https://trialsearchwhoint/Trial2aspx?TrialID=EUCTR2008-006018-97-GB. 2009.

4. Euctr GB. A Phase III, Multicenter, Randomized, Double-Blind, Placebo-Controlled, Parallel Group Study to Evaluate the Safety and Efficacy of MK-4305 in Patients with Primary Insomnia- Study B. https://trialsearchwhoint/Trial2aspx?TrialID=EUCTR2010-018414-69-GB. 2010.

5. Euctr SE. A Phase III, Multicenter, Randomized, Double-Blind, Placebo-Controlled, Parallel Group, Study to Evaluate the Safety and Efficacy of MK-4305 in Patients with Primary Insomnia – Study A. https://trialsearchwhoint/Trial2aspx?TrialID=EUCTR2010-018413-30-SE. 2010.<br>6. jRCT J. Phase 3 double blind study of ACT-541468. https://trialsearchwhoint/Trial2aspx?TrialID=JPRN-jRCT2031200452. 2021.<br>7. Euctr PL. Lemborexant for long-term treatment of insomnia disorder in adults. https://trialsearchwhoint/Trial2aspx?TrialID=EUCTR2015-001463-39-PL. 2016.<br>8. JapicCti. Phase 2 study of ACT-541468 in Japanese patients with insomnia disorder. https://trialsearchwhoint/Trial2aspx?TrialID=JPRN-JapicCTI-194667. 2019.<br>9. JapicCti. Long-term Study of ACT-541468 in patients with insomnia disorder. https://trialsearchwhoint/Trial2aspx?TrialID=JPRN-JapicCTI-205444. 2020.
**(4). Review (n=7)**<br>1. Howland RH. Suvorexant: a novel therapy for the treatment of insomnia. J Psychosoc Nurs Ment Health Serv. 2014;52(10):23-6.<br>2. Tampi R, Balachandran S, Manikkara G, Piyush T. Suvorexant for insomnia in older adults: A systematic review. American Journal of Geriatric Psychiatry. 2017;25(3):S147.<br>3. Citrome L. Suvorexant for insomnia: A systematic review of the efficacy and safety profile for this newly approved hypnotic - What is the number needed to treat, number needed to harm and likelihood to be helped or harmed? International Journal of Clinical Practice. 2014;68(12):1429-41.<br>4. Clark JW, Brian ML, Drummond SPA, Hoyer D, Jacobson LH. Effects of orexin receptor antagonism on human sleep architecture: A systematic review. Sleep Medicine Reviews. 2020;53.<br>5. Patel KV, Aspesi AV, Evoy KE. Suvorexant: A Dual Orexin Receptor Antagonist for the Treatment of Sleep Onset and Sleep Maintenance Insomnia. Annals of Pharmacotherapy. 2015;49(4):477-83.<br>6. Tampi RR, Manikkara G, Balachandran S, Taparia P, Hrisko S, Srinivasan S, et al. Suvorexant for insomnia in older adults: A perspective review. Drugs in Context. 2018;7.<br>7. Waters K. Review of the Efficacy and Safety of Lemborexant, a Dual Receptor Orexin Antagonist (DORA), in the Treatment of Adults With Insomnia Disorder. Annals of Pharmacotherapy. 2022;56(2):213-21.
**(5). Unfinished RCT (n=5)**<br>1. Eisai Co L, Eisai I. A Study of Lemborexant in Chinese Participants With Insomnia Disorder. 2022.<br>2. Eisai I. Study to Evaluate the Effect of 2 Dosage Strengths of Lemborexant (E2006) on a Multiple Sleep Latency Test in Participants With Insomnia Disorder. 2015.<br>3. Kurume U, Eisai I, Mebix I. An Open-label, Multicenter Study to Evaluate the Efficacy and Safety of Transitioning to Lemborexant in Japanese Subjects With Insomnia. 2022.<br>4. Nct. A Study of Lemborexant in Chinese Participants With Insomnia Disorder. https://clinicaltrialsgov/show/NCT04549168. 2020.<br>5. Nct. Study to Assess the Long Term Safety and Tolerability of ACT-541468 in Adult and Elderly Subjects Suffering From Difficulties to Sleep. https://clinicaltrialsgov/show/NCT03679884. 2018.
**(6). Meta-analysis (n=4)**

1. De Crescenzo F, D'Alò GL, Ostinelli EG, Ciabattini M, Di Franco V, Watanabe N, et al. Comparative effects of pharmacological interventions for the acute and long-term management of insomnia disorder in adults: a systematic review and network meta-analysis. The Lancet. 2022;400(10347):170-84.<br>2. Kishi T, Matsunaga S, Iwata N. Suvorexant for primary insomnia: A systematic review and meta-analysis of randomized placebo-controlled trials. PLoS ONE. 2015;10(8).<br>3. Kishi T, Nomura I, Matsuda Y, Sakuma K, Okuya M, Ikuta T, et al. Lemborexant vs suvorexant for insomnia: A systematic review and network meta-analysis. Journal of Psychiatric Research. 2020;128:68-74.<br>4. Soni P, Chawla E, Dabral S, Sharma S, Singh R, Goyal R. PMH9 Comparison of Approved Doses of Lemborexant for Insomnia in Placebo-Controlled Trials: A Systematic Review and Network Meta-Analysis. Value in Health Regional Issues. 2020;22:S63.
**(7). Other trials(n=3)**<br>1. Asai Y, Sano H, Miyazaki M, Iwakura M, Maeda Y, Hara M. Suvorexant (Belsomra® Tablets 10, 15, and 20 mg): Japanese Drug-Use Results Survey. Drugs in R and D. 2019;19(1):27-46.<br>2. Yardley J, Kärppä M, Inoue Y, Pinner K, Perdomo C, Ishikawa K, et al. Long-term effectiveness and safety of lemborexant in adults with insomnia disorder: results from a phase 3 randomized clinical trial. Sleep Medicine. 2021;80:333-42.<br>3. Drake CL, Cuamatzi-Castelan AS, Kalmbach DA, Cheng P, Roth T, Singh M, et al. Arousability of insomnia patients is not impacted by the orexin antagonist suvorexant (10 mg and 20 mg). Sleep. 2019;42:A151-A2.
**(8). Exceed inclusion criteria (n=3)**<br>1. Fan B, Kang J, He Y, Hao M, Du W, Ma S. Efficacy and safety of suvorexant for the treatment of primary insomnia among Chinese: A 6-month randomized double-blind controlled study. Neurology Asia. 2017;22(1):41-7.<br>2. Euctr BE. A study to find out which dose of daridorexant is safe and effective to treat insomnia in children and adolescents 10 to <18 years old. https://trialsearchwhoint/Trial2aspx?TrialID=EUCTR2021-003867-87-BE. 2022.<br>3. Nct. Dose-finding Study Assessing the Efficacy, Safety, and Pharmacokinetics of Daridorexant in Subjects Aged 10 to < 18 Years With Insomnia Disorder. https://clinicaltrialsgov/show/NCT05423717. 2022.

eTable 3: Inclusion, exclusion criteria, study design and outcome assessments of the included studies.

**Trials**	**Herring et al. 2012 (NCT00792298)**
Inclusion Criteria	Patients were men and women 18 to 64 years of age, in good physical and mental health, diagnosed with primary insomnia and were required to meet PSG inclusion criteria of LPS of > 20 minutes on both PSG nights and mean WASO of ≥60 minutes on both nights with neither night <45 minutes.
Exclusion Criteria	Breast feeding, pregnant or planning to become pregnant during the study; Within the past 6 months before starting the study you have a history of significant cardiovascular disorder such as unstable angina, congestive heart - failure or acute coronary syndrome; Currently participating or have participated in a study with an investigational compound or device within the last 30 days; Has traveled across 3 or more time zones in the last 2 weeks or plans on traveling across 3 or more time zones at any time during the study; Has done shift work within the past 2 weeks; Has donated blood products within the last 8 weeks; Has difficulty sleeping due to a medical condition.
Study Design	This was a randomized, double-blind, placebo-controlled, 2-period crossover PSG study to assess 4 doses of suvorexant (10, 20, 40, and 80 mg) in patients with primary insomnia.
Efficacy Outcomes	The SE (defined as TST divided by time in bed in minutes [fixed at 480 for this study] multiplied by 100) on night 1 and at the end of week 4; the WASO (defined as duration of wakefulness after persistent sleep onset to lights on) and LPS (defined as the duration of time from lights off to persistent sleep onset), measured on night 1 and at the end of week 4; Other exploratory end points included TST, NAW, sTST, sTSO, sQual, sFresh, ISI and SDS score.
Safety Outcomes	Adverse events and serious adverse events.

**Trials**	**Michelson et al. 2014 (NCT01021813)**
Inclusion Criteria	Diagnosis of primary insomnia; Participant is able to read, understand, and complete questionnaires and diaries; If female, participant and partner both agree to use acceptable contraception. If male partner does not use an effective form of contraception, female participant must use 2 acceptable forms of contraception; If ≥65 years of age, score of ≥25 on the Mini Mental State Examination (MMSE).
Exclusion Criteria	If female, participant is pregnant; Participant expects to donate eggs or sperm during the study; Recent and/or active history of a confounding neurological disorder; History of clinically unstable cardiovascular disorder within the last 6 months; Lifetime history of bipolar disorder; Psychiatric condition that requires treatment with a medication prohibited by the study, or any other psychiatric condition that would interfere with the participant's ability to participate in the study; History of substance abuse/dependence; History of cancer ≤5 years prior to study participation except for adequately treated basal cell or squamous cell skin cancer or in situ cervical cancer; Evidence of suicidality; Participant has travelled across >3 time zones or >3 hour time difference in the last 2 weeks; History of permanent night shift work or rotating day/night shift work in the past 2 weeks; Body Mass Index (BMI) >40 kg/m^2.
Study Design	After a 1-week single-blind placebo run-in screening phase, patients were randomly assigned to receive double-blind treatment for 1 year with suvorexant or placebo at a 2:1 ratio. The dose of suvorexant was 30 mg nightly for elderly patients and 40 mg nightly for nonelderly patients.
Efficacy Outcomes	Assessments of sTST and sTSO during the first month of treatment. Efficacy at later time points (months 2–12) were pre-specified exploratory endpoints. Other diary endpoints (sWASO, sNAW, sQual, sFresh) and rating scale endpoints (ISI) were also exploratory.
Safety Outcomes	Adverse events and serious adverse events.

**Trials**	**Herring et al. 2016**<br>**Trial 1 (NCT01097616) and Trial 2 (NCT01097629)**
Inclusion Criteria	Must be ≥18 years old on the day of signing informed consent; Diagnosed with Primary Insomnia; Good physical and mental health; Participant ≥65 years old score at least 25 on the Mini Mental State Examination; A female participant who is of reproductive potential has a negative serum pregnancy test and agrees to use contraception; Reports difficulty with initiating and maintaining sleep during the 4 weeks prior to Visit 1 (accordingly to specific protocol criteria); Reports spending 6.5 to 9 hours nightly in bed on at least 3 out of 7 nights prior to Visit 1; Regular bedtime is between 9 pm-1 am; Willing to refrain from napping while in study; Able to read, understand and complete questionnaires and all diaries; Willing to limit alcohol, caffeine, and nicotine consumption while in the study; For a portion of participants: Must be willing to stay overnight in a sleep laboratory and must be willing to stay in bed for at least 8 hours each night while at the sleep laboratory.
Exclusion Criteria	Female participant is pregnant and/or breastfeeding at Pre-study visit, or expecting to conceive while in study; History or diagnosis of another sleep disorder; Difficulty sleeping due to a medical condition; History of a neurological disorder; History of bipolar disorder, psychotic disorder, or posttraumatic stress disorder, or current psychiatric disorder that requires a prohibited medication; Ongoing depression; History of substance abuse or dependence; History or current evidence of a clinically significant cardiovascular disorder or clinically significant electrocardiogram (ECG) at Pre-study Visit; Taking certain prohibited medications; Consumption of the equivalent of >15 cigarettes a day; History of malignancy ≤5 years prior to signing informed consent, except for adequately treated basal cell or squamous cell skin cancer or in situ cervical cancer; Participant is considered morbidly obese; Previously randomized in another investigational study of suvorexant.
Study Design	Each trial included a 2-week, single-blind placebo run-in and a3-month, double-blind, placebo-controlled, three-arm treatment phase. Trial 1 included an optional 3-month double-blind extension for patients who completed the initial treatment phase, and both trials included a 1-week double-blind run-out at the end of treatment (3 months or 6 months). After the run-in, patients were randomized to 3 months of treatment with suvorexant 40/30 mg, suvorexant 20/15 mg, or placebo in a 3:2:3 ratio in trial 1 and a 1:1:1 ratio (Q-cohort) or a 2:1:2 ratio (PQ-cohort) in trial 2. Doses differed by age to adjust for previously observed plasma exposure differences (<65 years: 40 mg or 20 mg; ≥65 years: 30 mg or 15 mg).
Efficacy Outcomes	Change from baseline at months 1 and 3 for subjective and objective measures of sleep maintenance (sTST, WASO) and sleep onset (sTSO, LPS). Monthly values for the subjective end points were the mean of the daily values for the last week (month 1) or 2 weeks (month 3) of the month. Secondary end points included these same variables assessed for suvorexant 40/30 mg at week 1 for subjective end points (sTST and sTSO; mean of daily values over week 1) and night 1 for objective end points (WASO and LPS). These variables were also assessed for suvorexant 20/15 mg as secondary (trial 1) or exploratory (trial 2) end points. Other diary end points (sWASO, NAW, sQUAL, sFresh) and rating scale end points (ISI) were exploratory.
Safety Outcomes	Adverse events and serious adverse events.

Trials	Murphy et al. 2017 (NCT01995838)
Inclusion Criteria	Study participants were men and women 19 to 80 years of age who had insomnia disorder. Subjects were also required to meet the following objective inclusion criteria on 2 consecutive screening/baseline PSGs: LPS average of ≥ 30 minutes with neither night < 15 minute; and/or WASO average of ≥ 30 minutes with neither night < 20 minute; and an SE average of ≤ 85% with neither night > 87.5%.
Exclusion Criteria	Subjects with diagnosis of a sleep disorder other than insomnia were excluded. Use of sleep medication or concomitant medications to treat insomnia symptoms within 2 weeks of first screening/baseline PSG, or having a current diagnosis or being treated for major medical or psychiatric disorders excluded subjects from this study
Study Design	A multicenter, randomized, double-blind, placebo-controlled, parallel-group, Bayesian adaptive randomization design, dose response study of the efficacy of lemborexant in adults and elderly subjects with chronic insomnia.
Efficacy Outcomes	The primary study objective was SE as measured by PSG and residual morning sleepiness as rated on the Karolinska Sleepiness Scale (KSS). Secondary objectives included the change from mean at baseline to mean after dosing on day 1 and day 2 for SE, LPS, WASO, the change in SE, LPS, and WASO from mean baseline to mean after dosing on days 14 and 15, the change from baseline in mean SE, LPS, and WASO after the first 2 doses compared with change from baseline in mean SE, LPS, and WASO after the last 2 doses and the change from mean SE at baseline to mean SE after dosing (with placebo washout) on days 16 and 17. Exploratory efficacy objectives included sSE, sTSO, sWASO.
Safety Outcomes	Adverse events and serious adverse events.

Trials	Rosenberg et al. 2019<br>(SUNRISE 1)<br>(NCT02783729)
Inclusion Criteria	Women 55 years and older and men 65 years and older were eligible for participation if they met the DSM-5 criteria for insomnia disorder. Participants were required to have a history of sWASO of 60 minutes or more at least 3 nights per week in the previous 4 weeks, regular time spent in bed (7-9 hours), evidence of sleep maintenance insomnia, and an ISI score of 13 or greater.
Exclusion Criteria	A current diagnosis of sleep-related breathing disorder; history of sleep-eating or reports a history of sleep-related violent behavior, sleep-driving, or symptoms of another parasomnia that in the investigator's opinion make the participant unsuitable for the study; Apnea-Hypopnea Index > 15 or Periodic Limb Movement with Arousal Index >15; Beck Depression Inventory - II (BDI-II) score >19 at Screening; Beck Anxiety Index (BAI) score >15 at Screening; Habitually naps during the day more than 3 times per week; Excessive caffeine use, or habitually consumes caffeine-containing beverages after 18:00 and is unwilling to forego caffeine after 18:00 for the duration of his/her participation in the study; History of drug or alcohol dependency or abuse within approximately the previous 2 years; Known to be positive for human immunodeficiency virus; Active viral hepatitis (B or C) as demonstrated by positive serology at Screening ; Current evidence of clinically significant disease or chronic pain; suicidal ideation; Scheduled for surgery during the study; Used any modality of treatment for insomnia; Transmeridian travel across more than 3 time zones in the 2 weeks before Screening, or between Screening and Baseline, or plans to travel across more than 3 time zones during the study; Hypersensitivity to lemborexant or zolpidem or to their excipients; Currently enrolled in another clinical trial or previously participated in any clinical trial of lemborexant.
Study Design	The Study of the Efficacy and Safety of Lemborexant in Subjects 55 Years and Older With Insomnia Disorder (SUNRISE 1) clinical trial was a global randomized double-blind parallel-group placebo-controlled active-comparator phase 3 study.
Efficacy Outcomes	Efficacy outcomes included change from baseline in PSG measures of sleep onset and maintenance at the beginning and end of treatment. The primary end point included LPS after the last 2 nights of 1 month of treatment. Key secondary end points included SE, WASO, and WASO in the second half of the night (WASO2H; minutes of wake from 240 minutes after lights off until lights on). Additional secondary end points included sTSO, sSE, sWASO. Each sleep diary parameter was calculated as the mean of the first 7 (week 1) and last 7 (week 4) nights of treatment. Disease severity and daily functioning were assessed using the patient-rated ISI.
Safety Outcomes	Adverse events, serious adverse events and death.

Trials	Dauvilliers et al. 2020 (NCT02839200)
Inclusion Criteria	Eligible subjects were men and women 18 to 64 years of age who met the DSM-5 criteria for insomnia disorder. Subjects were required to have a self-reported history of ≥30 min LPS, ≥30 min WASO, a total sleep time ≤6.5 hours on at least three of seven consecutive nights, and a bedtime between 21: 30 and 00: 30 hours. Subjects also had to have an ISI score of at least 15.
Exclusion Criteria	Subjects with lifetime suicidal behavior, suicidal ideation, unstable medical condition or significant medical disorder (i.e., depression and anxiety) assessed by clinical judgement that could interfere with safety, treatment compliance and study assessments were excluded. Other exclusion criteria included ongoing sleep disorders other than insomnia, treatment with CNS-active drug, and cognitive behavior therapy for insomnia within one month prior to study start. Subjects were excluded before randomization based on occurrence of an apnea or hypopnea event above 10 per hour.
Study Design	A randomized, double-blind, placebo-controlled and active-controlled dose-response phase 2 study. Adults (≤64 years) with insomnia disorder were randomized (1:1:1:1:1:1) to receive daily oral placebo, daridorexant (5, 10, 25, or 50 mg), or 10 mg zolpidem for 30 days. A screening period of 14 to 28 days, including a single-blind run-in phase, was followed by a 30-day double-blind treatment period, a single-blind placebo run-out for 1 day, and a 30-day safety follow-up period.
Efficacy Outcomes	The primary efficacy outcome measure was change in WASO from baseline (mean of the two values of the run-in PSGs) to Days 1&2. Secondary efficacy outcome measures were change in LPS from baseline to Days 1&2 and change in sWASO and sTSO from baseline recording to Week 4 average. Other efficacy outcome measures included the change from baseline to all time-points of measurement in WASO, LPS, TST, sWASO, sTSO, sTST, sleep quality assessed by VAS, and ISI scores.
Safety Outcomes	Adverse events and serious adverse events.

Trials	Karppa et al. 2020<br>(SUNRISE 2)<br>(NCT02952820)
Inclusion Criteria	Adult (≥18 years of age) males and females with insomnia disorder meeting DSM-5 criteria were eligible for the study. Participants had a history of sTSO ≥30 min and/or sWASO ≥60 min at least three times a week in the previous 4 weeks before enrollment. Participants were required to score ≥15 on the ISI. In addition, participants reported a regular time in bed between 7 and 10 h at the second screening visit, a habitual bedtime between 09: 00 pm and 01: 00 am and a habitual wake time between 05: 00 am and 10: 00 am. Eligibility criteria were confirmed by sleep history, questionnaires, and sleep diary.
Exclusion Criteria	Individuals with diagnosed comorbid sleep disorders, including sleep apnea, periodic limb movement disorder, restless legs syndrome, circadian rhythm sleep disorder or narcolepsy, and individuals with a history of complex sleep-related behavior were excluded from the trial. Additional exclusion criteria included a diagnosis of a major medical or psychiatric disorder or disorder that was not, in the opinion of the investigator, adequately treated, history of abnormal nocturnal behaviors, nocturia, excessive caffeine consumption, history of drug or alcohol dependency or abuse, positive drug screen, recent use of any pharmacologic or nonpharmacologic insomnia treatment and suvorexant treatment failure.
Study Design	SUNRISE 2 was a 12-month, global, multicenter, randomized, double-blind, parallel-group phase 3 study which was placebo-controlled for the first 6 months (Period 1), then active drug only for the next 6 months (Period 2).
Efficacy Outcomes	The primary efficacy endpoint was mean change from baseline in sTSO at the end of month 6. Key secondary efficacy end points were mean changes from baseline in sSE and sWASO at the end of month 6. Additional secondary endpoints included mean change from baseline in sTST at the end of month 6, mean changes from baseline in sTSO, sSE, sWASO, and sTST at the ends of the first 7 nights, month 1 and month 3, and the proportions of sleep onset and sleep maintenance responders to LEM5 or LEM10 compared with placebo at the end of month 6. Secondary endpoints also included mean changes in subject rated morning sleepiness/alertness and subject-reported quality of sleep at the end of month 6.
Safety Outcomes	Adverse events, serious adverse events and death.

Trials	Zammit et al. 2020 (NCT02841709)
Inclusion Criteria	Eligible participants were ≥65 years of age with a diagnosis of insomnia disorder according to the DSM-V criteria and an ISI score ≥15. Participants were required to meet the following criteria for self-reported sleep parameters on at least 3 nights per week for at least 3 months before the screening period: ≥30 minutes to fall asleep, wake time during sleep ≥30 minutes, and a TST ≤6.5 hours. Participants were also required to fulfill baseline PSG sleep measurement requirements on the 2 PSG nights before randomization during the single-blind placebo run-in (mean LPS ≥20 minutes, mean WASO ≥30 minutes, and mean TST <420 minutes).
Exclusion Criteria	Exclusion criteria included any sleep disorder other than insomnia ongoing at screening; any lifetime history of related breathing disorder, periodic limb movement disorder, restless legs syndrome, circadian rhythm disorder, REM sleep behavior disorder or narcolepsy; apnea/hypopnea index ≥15 per hour; periodic limb movement with arousal index ≥15 per hour on the first PSG screening night; treatment with prohibited CNS-active drugs for 5 half-lives of the respective drug (but at least 2 weeks) before screening and until 24 hours after end of treatment (EOT), including over-the-counter medication and herbal medicines; treatment with moderate/strong CYP3A4 inhibitors/inducers; and self-reported usual daytime napping for at least 1 h/d (at least 3 d/wk.), caffeine consumption ≥600 mg/d, shift work within 2 weeks before screening, and travel across at least 3 time zones within 1 week before screening or planned travel across at least 3 time zones during the study.
Study Design	A multicenter, double-blind, randomized, placebo-controlled, 5-period, 5-treatment crossover, phase 2 study that consisted of 3 consecutive phases. Elderly (≥65 years) participants with insomnia were randomly allocated (Latin square design) to receive 5 treatments (5, 10, 25, and 50 mg daridorexant and placebo) during 5 treatment periods, each consisting of 2 treatment nights followed by a 5- to 12-day washout period.
Efficacy Outcomes	The primary efficacy endpoint was mean change from baseline in sTSO at the end of month 6. Key secondary efficacy end points were mean changes from baseline in sSE and sWASO at the end of month 6. Additional secondary endpoints included mean change from baseline in sTST at the end of month 6, mean changes from baseline in sTSO, sSE, sWASO, and sTST at the ends of the first 7 nights, month 1 and month 3, and the proportions of sleep onset and sleep maintenance responders to LEM5 or LEM10 compared with placebo at the end of month 6. Secondary endpoints also included mean changes in subject rated morning sleepiness/alertness and subject-reported quality of sleep at the end of month 6.
Safety Outcomes	Adverse events and serious adverse events.

Trials	Mignot et al. 2022<br>Trial 1 (NCT03545191) and Trial 2 (NCT03575104)
Inclusion Criteria	Eligible participants were adults (aged ≥18 years) who had insomnia disorder (according to the DSM-5) that was of moderate or severe intensity at screening (ISI score ≥15). An additional inclusion criterion was a self-reported history of disturbed sleep (i.e., all the following: ≥30 min to fall asleep, ≥30 min awakes during sleep time, and self- reported total sleep time of ≤6.5 h) on at least three nights per week for at least 3 months before screening. During the placebo run-in period, these self-reported sleep parameters were also required to be met on at least three of seven nights. Moreover, during the placebo run-in period, PSG criteria had to be met (i.e., all the following: LPS ≥20 min, WASO ≥30 min, and mean total sleep time of <7 h).
Exclusion Criteria	Exclusion criteria included self-reported daytime napping (≥1 h per day on ≥3 days per week); a history of suicidal ideation or attempt, acute or chronic psychiatric condition not controlled by therapy, severe depression, or alcohol or drug misuse; an apnea or hypopnea index of 15 events per h or higher (per American Academy of Sleep Medicine criteria) or an event associated with oxygen saturation of less than 80% (assessed by PSG); or periodic limb movement index of 15 or more events per h (assessed by PSG), restless legs syndrome, circadian rhythm disorder, rapid-eye-movement behavior disorder, or narcolepsy.
Study Design	A multicenter, randomized, double-blind, placebo-controlled, parallel-group trials to assess the safety and efficacy of daridorexant in people with insomnia. The trials consisted of a screening period (7–18 days), a single-blind placebo run-in period (13–24 days), a double-blind treatment period (3 months), and a single-blind placebo run-out period (7 days), followed by either a safety follow- up period (23 days) or participation in a 9-month placebo-controlled extension trial.
Efficacy Outcomes	The primary endpoints were change from baseline in WASO and LPS at months 1 and 3, measured by PSG in a sleep laboratory. The secondary endpoints were change from baseline in self- reported total sleep time and in the IDSIQ sleepiness domain score, using the eDiary, at months 1 and 3. Other prespecified efficacy endpoints were change from baseline in other IDSIQ domain scores and total IDSIQ score at months 1 and 3, and change from baseline in total sleep time at months 1 and 3, as measured by PSG. Exploratory endpoints included evening visual analogue scales for ability to function and daytime alertness, and morning visual analogue scales for depth and quality of sleep (mean of daily entries in the 7 days before PSG nights). The proportion of sleep spent in each sleep phase (measured by PSG), and ISI score, were also evaluated as exploratory endpoints.
Safety Outcomes	Adverse events and serious adverse events.

PSG: polysomnography; LPS: latency to persistent sleep; WASO: wake after sleep onset; SE: sleep efficiency; TST: total sleep time; NAW: number of wakenings; sTST: subjective total sleep time; sTSO: subjective time to sleep onset; sSE: subjective sleep efficiency; sQual: subjective quality of sleep; sFresh: subjective refreshed feeling on waking; sWASO: subjective wake after sleep onset; sNAW: subjective number of wakenings; ISI: insomnia severity index; SDS: sheehan disability scale; DSM-5: Diagnostic and Statistical Manual of Mental Disorders, fifth edition; IDSIQ: Insomnia Daytime Symptoms and Impacts

Questionnaire.

eTable 4: The deviance information criteria (DIC) and I^2 values of fixed-effects model, random-effects model and their corresponding unrelated means models in network meta-analysis.

Outcome measure	Estimate from network meta-analysis							
	Fixed-Effects Model		Unrelated Means Model		Random-Effects Model		Unrelated Means Model	
	DIC	I^2 (%)	DIC	I^2 (%)	DIC	I^2 (%)	DIC	I^2 (%)
LPS	87.60	33			78.80	8	70.74	0
sTSO	52.48	0	54.09	0	54.13	0		
WASO	117.78	55			81.63	7	87.68	14
sWASO	53.74	12	54.88	14	54.39	6		
TST	44.21	50			33.48	7	33.52	7
sTST	69.71	1	70.18	0	71.13	0		
ISI	45.54	22			44.77	6	44.39	3
AE	66.01	0	76.45	0	68.15	0		
SAE	58.09	39			48.58	12	46.11	2

Abbreviation: LPS: latency to persistent sleep; sTSO: subjective time to sleep onset; WASO: wake after sleep onset; sWASO: subjective wake after sleep onset; TST: total sleep time; sTST: subjective total sleep time; ISI: insomnia severity index score; AE: adverse events; SAE: serious adverse events.

eTable 5: Funnel plot for each outcome.

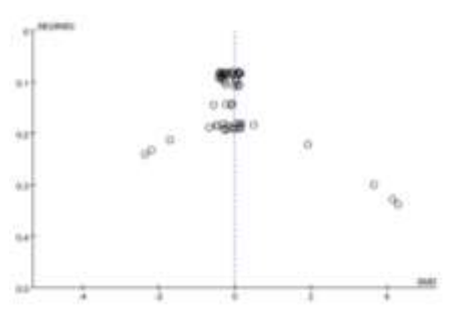	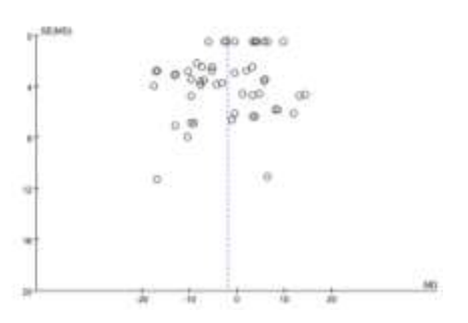	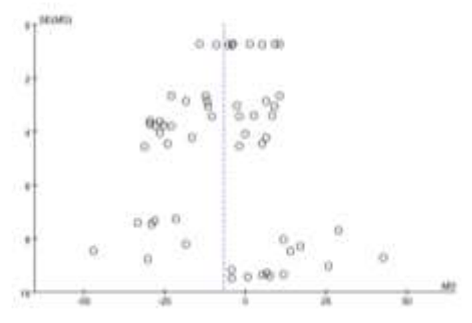
**sWASO**	**TST**	**sTST**
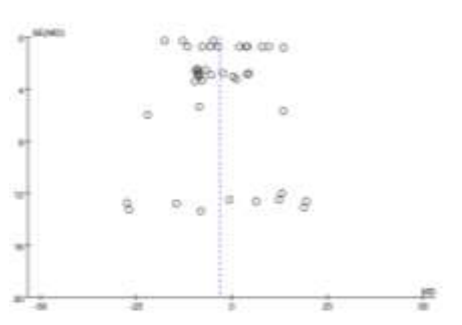	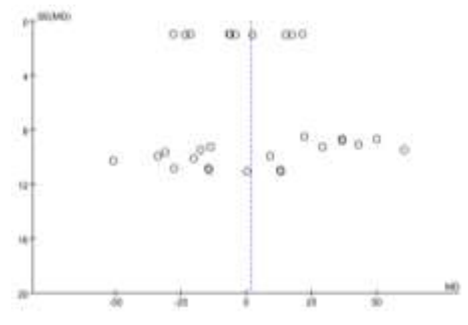	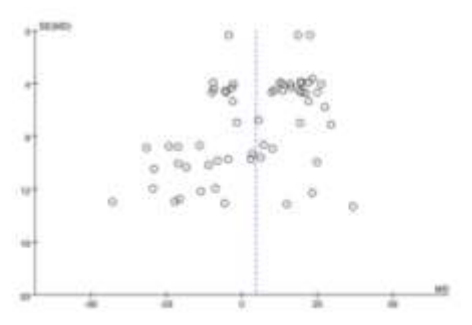
**ISI**	**AE**	**sAE**
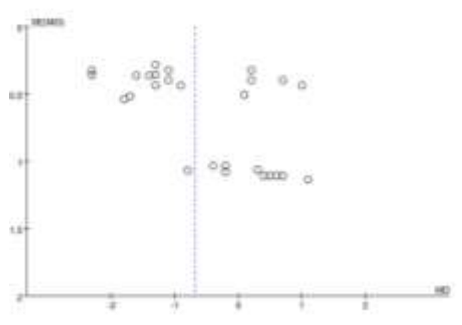	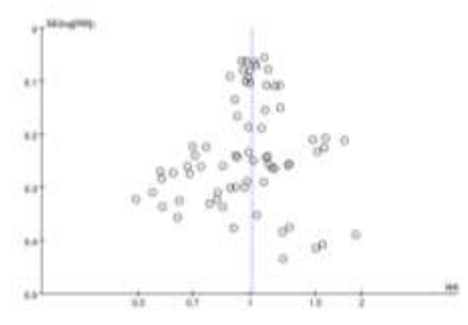	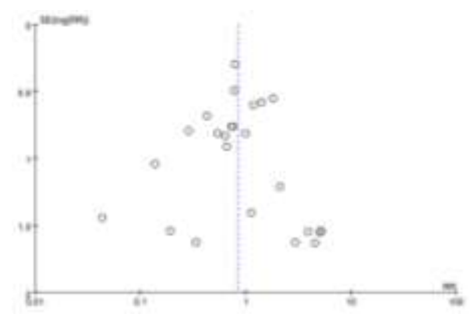

eTable 6: Detailed certainty of evidence for each outcome in league table.

Comparison	Number of studies	Within-study bias	Reporting bias	Indirectness	Imprecision	Heterogeneity	Incoherence	Confidence rating	Reason(s) for downgrading
**1. LPS**									
A_Suvorexant_10mg:B_Suvorexant_20mg	1	No concerns	Low risk	No concerns	Some concerns	No concerns	Major concerns	Very low	["Imprecision","Incoherence"]
A_Suvorexant_10mg:C_Suvorexant_40mg	1	No concerns	Low risk	No concerns	Some concerns	No concerns	No concerns	Very low	["Imprecision"]
A_Suvorexant_10mg:D_Suvorexant_80mg	1	No concerns	Low risk	No concerns	Some concerns	No concerns	Major concerns	Very low	["Imprecision","Incoherence"]
A_Suvorexant_10mg:O_Placebo	1	No concerns	Low risk	No concerns	Major concerns	No concerns	No concerns	Low	["Imprecision"]
B_Suvorexant_20mg:C_Suvorexant_40mg	5	No concerns	Low risk	No concerns	No concerns	No concerns	No concerns	High	[]
B_Suvorexant_20mg:D_Suvorexant_80mg	1	No concerns	Low risk	No concerns	Major concerns	No concerns	Major concerns	Very low	["Imprecision","Incoherence"]
B_Suvorexant_20mg:O_Placebo	5	No concerns	Low risk	No concerns	No concerns	No concerns	No concerns	High	[]
C_Suvorexant_40mg:D_Suvorexant_80mg	1	No concerns	Low risk	No concerns	Major concerns	No concerns	No concerns	Low	["Imprecision"]
C_Suvorexant_40mg:O_Placebo	5	No concerns	Low risk	No concerns	No concerns	No concerns	Some concerns	Moderate	["Incoherence"]
D_Suvorexant_80mg:O_Placebo	1	No concerns	Low risk	No concerns	Some concerns	No concerns	No concerns	Moderate	["Imprecision"]
G_Lemborexant_5mg:H_Lemborexant_10mg	1	No concerns	Low risk	No concerns	No concerns	Some concerns	Major concerns	Very low	["Heterogeneity","Incoherence"]
G_Lemborexant_5mg:O_Placebo	1	No concerns	Low risk	No concerns	No concerns	No concerns	Major concerns	Low	["Incoherence"]
H_Lemborexant_10mg:O_Placebo	1	No concerns	Low risk	No concerns	No concerns	No concerns	Major concerns	Low	["Incoherence"]
K_Daridorexant_5mg:L_Daridorexant_10mg	2	No concerns	Low risk	No concerns	No concerns	No concerns	Some concerns	Moderate	["Incoherence"]
K_Daridorexant_5mg:M_Daridorexant_25mg	2	No concerns	Low risk	No concerns	No concerns	No concerns	No concerns	High	[]
K_Daridorexant_5mg:N_Daridorexant_50mg	2	No concerns	Low risk	No concerns	No concerns	No concerns	Some concerns	Moderate	["Incoherence"]
K_Daridorexant_5mg:O_Placebo	2	No concerns	Low risk	No concerns	No concerns	No concerns	No concerns	High	[]
L_Daridorexant_10mg:M_Daridorexant_25mg	4	No concerns	Low risk	No concerns	No concerns	No concerns	No concerns	High	[]
L_Daridorexant_10mg:N_Daridorexant_50mg	2	No concerns	Low risk	No concerns	No concerns	No concerns	Some concerns	Moderate	["Incoherence"]
L_Daridorexant_10mg:O_Placebo	4	No concerns	Low risk	No concerns	No concerns	No concerns	No concerns	High	[]
M_Daridorexant_25mg:N_Daridorexant_50mg	4	No concerns	Low risk	No concerns	No concerns	No concerns	No concerns	High	[]
M_Daridorexant_25mg:O_Placebo	6	No concerns	Low risk	No concerns	No concerns	No concerns	Major concerns	Low	["Incoherence"]
N_Daridorexant_50mg:O_Placebo	4	No concerns	Low risk	No concerns	No concerns	No concerns	No concerns	High	[]
A_Suvorexant_10mg:G_Lemborexant_5mg	0	No concerns	Low risk	No concerns	Some concerns	No concerns	Major concerns	Very low	["Imprecision","Incoherence"]
A_Suvorexant_10mg:H_Lemborexant_10mg	0	No concerns	Low risk	No concerns	Some concerns	No concerns	Major concerns	Very low	["Imprecision","Incoherence"]
A_Suvorexant_10mg:K_Daridorexant_5mg	0	No concerns	Low risk	No concerns	Some concerns	No concerns	Major concerns	Very low	["Imprecision","Incoherence"]

A_Suvorexant_10mg:L_Daridorexant_10mg	0	No concerns	Low risk	No concerns	Some concerns	No concerns	Major concerns	Very low	["Imprecision","Incoherence"]
A_Suvorexant_10mg:M_Daridorexant_25mg	0	No concerns	Low risk	No concerns	Some concerns	No concerns	Major concerns	Very low	["Imprecision","Incoherence"]
A_Suvorexant_10mg:N_Daridorexant_50mg	0	No concerns	Low risk	No concerns	Some concerns	No concerns	Major concerns	Very low	["Imprecision","Incoherence"]
B_Suvorexant_20mg:G_Lemborexant_5mg	0	No concerns	Low risk	No concerns	Some concerns	No concerns	Major concerns	Very low	["Imprecision","Incoherence"]
B_Suvorexant_20mg:H_Lemborexant_10mg	0	No concerns	Low risk	No concerns	Some concerns	No concerns	Major concerns	Very low	["Imprecision","Incoherence"]
B_Suvorexant_20mg:K_Daridorexant_5mg	0	No concerns	Low risk	No concerns	No concerns	No concerns	Major concerns	Low	["Incoherence"]
B_Suvorexant_20mg:L_Daridorexant_10mg	0	No concerns	Low risk	No concerns	No concerns	No concerns	Major concerns	Low	["Incoherence"]
B_Suvorexant_20mg:M_Daridorexant_25mg	0	No concerns	Low risk	No concerns	No concerns	No concerns	Major concerns	Low	["Incoherence"]
B_Suvorexant_20mg:N_Daridorexant_50mg	0	No concerns	Low risk	No concerns	No concerns	No concerns	Major concerns	Low	["Incoherence"]
C_Suvorexant_40mg:G_Lemborexant_5mg	0	No concerns	Low risk	No concerns	Some concerns	No concerns	Major concerns	Very low	["Imprecision","Incoherence"]
C_Suvorexant_40mg:H_Lemborexant_10mg	0	No concerns	Low risk	No concerns	Some concerns	No concerns	Major concerns	Very low	["Imprecision","Incoherence"]
C_Suvorexant_40mg:K_Daridorexant_5mg	0	No concerns	Low risk	No concerns	No concerns	No concerns	Major concerns	Low	["Incoherence"]
C_Suvorexant_40mg:L_Daridorexant_10mg	0	No concerns	Low risk	No concerns	No concerns	No concerns	Major concerns	Low	["Incoherence"]
C_Suvorexant_40mg:M_Daridorexant_25mg	0	No concerns	Low risk	No concerns	No concerns	No concerns	Major concerns	Low	["Incoherence"]
C_Suvorexant_40mg:N_Daridorexant_50mg	0	No concerns	Low risk	No concerns	No concerns	No concerns	Major concerns	Low	["Incoherence"]
D_Suvorexant_80mg:G_Lemborexant_5mg	0	No concerns	Low risk	No concerns	Major concerns	No concerns	Major concerns	Very low	["Imprecision","Incoherence"]
D_Suvorexant_80mg:H_Lemborexant_10mg	0	No concerns	Low risk	No concerns	Some concerns	Some concerns	Major concerns	Very low	["Imprecision","Heterogeneity","Incoherence"]
D_Suvorexant_80mg:K_Daridorexant_5mg	0	No concerns	Low risk	No concerns	No concerns	No concerns	Major concerns	Low	["Incoherence"]
D_Suvorexant_80mg:L_Daridorexant_10mg	0	No concerns	Low risk	No concerns	Major concerns	No concerns	Major concerns	Very low	["Imprecision","Incoherence"]
D_Suvorexant_80mg:M_Daridorexant_25mg	0	No concerns	Low risk	No concerns	Major concerns	No concerns	Major concerns	Very low	["Imprecision","Incoherence"]
D_Suvorexant_80mg:N_Daridorexant_50mg	0	No concerns	Low risk	No concerns	Major concerns	No concerns	Major concerns	Very low	["Imprecision","Incoherence"]
G_Lemborexant_5mg:K_Daridorexant_5mg	0	No concerns	Low risk	No concerns	No concerns	No concerns	Major concerns	Low	["Incoherence"]
G_Lemborexant_5mg:L_Daridorexant_10mg	0	No concerns	Low risk	No concerns	Some concerns	No concerns	Major concerns	Very low	["Imprecision","Incoherence"]
G_Lemborexant_5mg:M_Daridorexant_25mg	0	No concerns	Low risk	No concerns	Some concerns	No concerns	Major concerns	Very low	["Imprecision","Incoherence"]
G_Lemborexant_5mg:N_Daridorexant_50mg	0	No concerns	Low risk	No concerns	Some concerns	No concerns	Major concerns	Very low	["Imprecision","Incoherence"]
H_Lemborexant_10mg:K_Daridorexant_5mg	0	No concerns	Low risk	No concerns	No concerns	No concerns	Major concerns	Low	["Incoherence"]
H_Lemborexant_10mg:L_Daridorexant_10mg	0	No concerns	Low risk	No concerns	Some concerns	No concerns	Major concerns	Very low	["Imprecision","Incoherence"]
H_Lemborexant_10mg:M_Daridorexant_25mg	0	No concerns	Low risk	No concerns	Some concerns	No concerns	Major concerns	Very low	["Imprecision","Incoherence"]
H_Lemborexant_10mg:N_Daridorexant_50mg	0	No concerns	Low risk	No concerns	Some concerns	No concerns	Major concerns	Very low	["Imprecision","Incoherence"]
**2. sTSO**									
A_Suvorexant_10mg:B_Suvorexant_20mg	1	No concerns	Low risk	No concerns	Some concerns	No concerns	No concerns	Moderate	["Imprecision"]

A_Suvorexant_10mg:C_Suvorexant_40mg	1	No concerns	Low risk	No concerns	No concerns	No concerns	Major concerns	Low	["Incoherence"]
A_Suvorexant_10mg:D_Suvorexant_80mg	1	No concerns	Low risk	No concerns	Some concerns	Some concerns	No concerns	Low	["Imprecision","Heterogeneity"]
A_Suvorexant_10mg:O_Placebo	1	No concerns	Low risk	No concerns	Major concerns	No concerns	No concerns	Low	["Imprecision"]
B_Suvorexant_20mg:C_Suvorexant_40mg	5	No concerns	Low risk	No concerns	No concerns	No concerns	No concerns	High	[]
B_Suvorexant_20mg:D_Suvorexant_80mg	1	No concerns	Low risk	No concerns	Major concerns	No concerns	No concerns	Low	["Imprecision"]
B_Suvorexant_20mg:O_Placebo	5	No concerns	Low risk	No concerns	No concerns	No concerns	No concerns	High	[]
C_Suvorexant_40mg:D_Suvorexant_80mg	1	No concerns	Low risk	No concerns	Some concerns	No concerns	Some concerns	Low	["Imprecision","Incoherence"]
C_Suvorexant_40mg:O_Placebo	7	No concerns	Low risk	No concerns	No concerns	No concerns	No concerns	High	[]
D_Suvorexant_80mg:O_Placebo	1	No concerns	Low risk	No concerns	Some concerns	No concerns	No concerns	Moderate	["Imprecision"]
G_Lemborexant_5mg:H_Lemborexant_10mg	2	No concerns	Low risk	No concerns	Major concerns	No concerns	No concerns	Low	["Imprecision"]
G_Lemborexant_5mg:O_Placebo	2	No concerns	Low risk	No concerns	No concerns	No concerns	No concerns	High	[]
H_Lemborexant_10mg:O_Placebo	2	No concerns	Low risk	No concerns	No concerns	No concerns	No concerns	High	[]
K_Daridorexant_5mg:L_Daridorexant_10mg	2	No concerns	Low risk	No concerns	No concerns	No concerns	No concerns	High	[]
K_Daridorexant_5mg:M_Daridorexant_25mg	2	No concerns	Low risk	No concerns	No concerns	No concerns	No concerns	High	[]
K_Daridorexant_5mg:N_Daridorexant_50mg	2	No concerns	Low risk	No concerns	No concerns	No concerns	No concerns	High	[]
K_Daridorexant_5mg:O_Placebo	2	No concerns	Low risk	No concerns	No concerns	No concerns	No concerns	High	[]
L_Daridorexant_10mg:M_Daridorexant_25mg	2	No concerns	Low risk	No concerns	No concerns	No concerns	No concerns	High	[]
L_Daridorexant_10mg:N_Daridorexant_50mg	2	No concerns	Low risk	No concerns	No concerns	No concerns	No concerns	High	[]
L_Daridorexant_10mg:O_Placebo	2	No concerns	Low risk	No concerns	No concerns	No concerns	No concerns	High	[]
M_Daridorexant_25mg:N_Daridorexant_50mg	2	No concerns	Low risk	No concerns	No concerns	No concerns	No concerns	High	[]
M_Daridorexant_25mg:O_Placebo	2	No concerns	Low risk	No concerns	No concerns	No concerns	No concerns	High	[]
N_Daridorexant_50mg:O_Placebo	2	No concerns	Low risk	No concerns	No concerns	No concerns	No concerns	High	[]
A_Suvorexant_10mg:G_Lemborexant_5mg	0	No concerns	Low risk	No concerns	No concerns	No concerns	No concerns	High	[]
A_Suvorexant_10mg:H_Lemborexant_10mg	0	No concerns	Low risk	No concerns	No concerns	No concerns	No concerns	High	[]
A_Suvorexant_10mg:K_Daridorexant_5mg	0	No concerns	Low risk	No concerns	Some concerns	No concerns	No concerns	Moderate	["Imprecision"]
A_Suvorexant_10mg:L_Daridorexant_10mg	0	No concerns	Low risk	No concerns	Major concerns	No concerns	No concerns	Low	["Imprecision"]
A_Suvorexant_10mg:M_Daridorexant_25mg	0	No concerns	Low risk	No concerns	Major concerns	No concerns	No concerns	Low	["Imprecision"]
A_Suvorexant_10mg:N_Daridorexant_50mg	0	No concerns	Low risk	No concerns	Some concerns	No concerns	No concerns	Moderate	["Imprecision"]
B_Suvorexant_20mg:G_Lemborexant_5mg	0	No concerns	Low risk	No concerns	No concerns	No concerns	No concerns	High	[]
B_Suvorexant_20mg:H_Lemborexant_10mg	0	No concerns	Low risk	No concerns	No concerns	No concerns	No concerns	High	[]
B_Suvorexant_20mg:K_Daridorexant_5mg	0	No concerns	Low risk	No concerns	No concerns	No concerns	No concerns	High	[]

B_Suvorexant_20mg:L_Daridorexant_10mg	0	No concerns	Low risk	No concerns	No concerns	No concerns	No concerns	High	[]
B_Suvorexant_20mg:M_Daridorexant_25mg	0	No concerns	Low risk	No concerns	No concerns	No concerns	No concerns	High	[]
B_Suvorexant_20mg:N_Daridorexant_50mg	0	No concerns	Low risk	No concerns	No concerns	No concerns	No concerns	High	[]
C_Suvorexant_40mg:G_Lemborexant_5mg	0	No concerns	Low risk	No concerns	No concerns	Some concerns	No concerns	Moderate	["Heterogeneity"]
C_Suvorexant_40mg:H_Lemborexant_10mg	0	No concerns	Low risk	No concerns	No concerns	Some concerns	No concerns	Moderate	["Heterogeneity"]
C_Suvorexant_40mg:K_Daridorexant_5mg	0	No concerns	Low risk	No concerns	No concerns	No concerns	No concerns	High	[]
C_Suvorexant_40mg:L_Daridorexant_10mg	0	No concerns	Low risk	No concerns	No concerns	No concerns	No concerns	High	[]
C_Suvorexant_40mg:M_Daridorexant_25mg	0	No concerns	Low risk	No concerns	No concerns	No concerns	No concerns	High	[]
C_Suvorexant_40mg:N_Daridorexant_50mg	0	No concerns	Low risk	No concerns	No concerns	No concerns	No concerns	High	[]
D_Suvorexant_80mg:G_Lemborexant_5mg	0	No concerns	Low risk	No concerns	No concerns	Some concerns	No concerns	Moderate	["Heterogeneity"]
D_Suvorexant_80mg:H_Lemborexant_10mg	0	No concerns	Low risk	No concerns	No concerns	No concerns	No concerns	High	[]
D_Suvorexant_80mg:K_Daridorexant_5mg	0	No concerns	Low risk	No concerns	No concerns	No concerns	No concerns	High	[]
D_Suvorexant_80mg:L_Daridorexant_10mg	0	No concerns	Low risk	No concerns	Some concerns	No concerns	No concerns	Moderate	["Imprecision"]
D_Suvorexant_80mg:M_Daridorexant_25mg	0	No concerns	Low risk	No concerns	Some concerns	No concerns	No concerns	Moderate	["Imprecision"]
D_Suvorexant_80mg:N_Daridorexant_50mg	0	No concerns	Low risk	No concerns	Major concerns	No concerns	No concerns	Low	["Imprecision"]
G_Lemborexant_5mg:K_Daridorexant_5mg	0	No concerns	Low risk	No concerns	No concerns	No concerns	No concerns	High	[]
G_Lemborexant_5mg:L_Daridorexant_10mg	0	No concerns	Low risk	No concerns	No concerns	No concerns	No concerns	High	[]
G_Lemborexant_5mg:M_Daridorexant_25mg	0	No concerns	Low risk	No concerns	No concerns	No concerns	No concerns	High	[]
G_Lemborexant_5mg:N_Daridorexant_50mg	0	No concerns	Low risk	No concerns	No concerns	No concerns	No concerns	High	[]
H_Lemborexant_10mg:K_Daridorexant_5mg	0	No concerns	Low risk	No concerns	No concerns	No concerns	No concerns	High	[]
H_Lemborexant_10mg:L_Daridorexant_10mg	0	No concerns	Low risk	No concerns	No concerns	No concerns	No concerns	High	[]
H_Lemborexant_10mg:M_Daridorexant_25mg	0	No concerns	Low risk	No concerns	No concerns	No concerns	No concerns	High	[]
H_Lemborexant_10mg:N_Daridorexant_50mg	0	No concerns	Low risk	No concerns	No concerns	No concerns	No concerns	High	[]
**3. WASO**									
A_Suvorexant_10mg:B_Suvorexant_20mg	1	No concerns	Low risk	No concerns	Some concerns	No concerns	No concerns	Moderate	["Imprecision"]
A_Suvorexant_10mg:C_Suvorexant_40mg	1	No concerns	Low risk	No concerns	Some concerns	No concerns	No concerns	Moderate	["Imprecision"]
A_Suvorexant_10mg:D_Suvorexant_80mg	1	No concerns	Low risk	No concerns	Some concerns	No concerns	No concerns	Moderate	["Imprecision"]
A_Suvorexant_10mg:O_Placebo	1	No concerns	Low risk	No concerns	No concerns	Some concerns	No concerns	Moderate	["Heterogeneity"]
B_Suvorexant_20mg:C_Suvorexant_40mg	5	No concerns	Low risk	No concerns	No concerns	No concerns	No concerns	High	[]
B_Suvorexant_20mg:D_Suvorexant_80mg	1	No concerns	Low risk	No concerns	No concerns	Some concerns	No concerns	Moderate	["Heterogeneity"]
B_Suvorexant_20mg:O_Placebo	5	No concerns	Low risk	No concerns	No concerns	No concerns	No concerns	High	[]

C_Suvorexant_40mg:D_Suvorexant_80mg	1	No concerns	Low risk	No concerns	No concerns	Major concerns	No concerns	Low	["Heterogeneity"]
C_Suvorexant_40mg:O_Placebo	5	No concerns	Low risk	No concerns	No concerns	No concerns	No concerns	High	[]
D_Suvorexant_80mg:O_Placebo	1	No concerns	Low risk	No concerns	No concerns	No concerns	No concerns	High	[]
G_Lemborexant_5mg:H_Lemborexant_10mg	1	No concerns	Low risk	No concerns	No concerns	No concerns	No concerns	High	[]
G_Lemborexant_5mg:O_Placebo	1	No concerns	Low risk	No concerns	No concerns	No concerns	No concerns	High	[]
H_Lemborexant_10mg:O_Placebo	1	No concerns	Low risk	No concerns	No concerns	No concerns	No concerns	High	[]
K_Daridorexant_5mg:L_Daridorexant_10mg	2	No concerns	Low risk	No concerns	No concerns	No concerns	Some concerns	Moderate	["Incoherence"]
K_Daridorexant_5mg:M_Daridorexant_25mg	2	No concerns	Low risk	No concerns	No concerns	Some concerns	No concerns	Moderate	["Heterogeneity"]
K_Daridorexant_5mg:N_Daridorexant_50mg	2	No concerns	Low risk	No concerns	No concerns	No concerns	No concerns	High	[]
K_Daridorexant_5mg:O_Placebo	2	No concerns	Low risk	No concerns	No concerns	No concerns	No concerns	High	[]
L_Daridorexant_10mg:M_Daridorexant_25mg	4	No concerns	Low risk	No concerns	No concerns	No concerns	No concerns	High	[]
L_Daridorexant_10mg:N_Daridorexant_50mg	2	No concerns	Low risk	No concerns	No concerns	Some concerns	No concerns	Moderate	["Heterogeneity"]
L_Daridorexant_10mg:O_Placebo	4	No concerns	Low risk	No concerns	No concerns	No concerns	No concerns	High	[]
M_Daridorexant_25mg:N_Daridorexant_50mg	4	No concerns	Low risk	No concerns	No concerns	Some concerns	No concerns	Moderate	["Heterogeneity"]
M_Daridorexant_25mg:O_Placebo	6	No concerns	Low risk	No concerns	No concerns	Some concerns	No concerns	Moderate	["Heterogeneity"]
N_Daridorexant_50mg:O_Placebo	4	No concerns	Low risk	No concerns	No concerns	No concerns	No concerns	High	[]
A_Suvorexant_10mg:G_Lemborexant_5mg	0	No concerns	Low risk	No concerns	Some concerns	No concerns	No concerns	Moderate	["Imprecision"]
A_Suvorexant_10mg:H_Lemborexant_10mg	0	No concerns	Low risk	No concerns	Some concerns	No concerns	No concerns	Moderate	["Imprecision"]
A_Suvorexant_10mg:K_Daridorexant_5mg	0	No concerns	Low risk	No concerns	Some concerns	No concerns	No concerns	Moderate	["Imprecision"]
A_Suvorexant_10mg:L_Daridorexant_10mg	0	No concerns	Low risk	No concerns	Some concerns	No concerns	No concerns	Moderate	["Imprecision"]
A_Suvorexant_10mg:M_Daridorexant_25mg	0	No concerns	Low risk	No concerns	Some concerns	No concerns	No concerns	Moderate	["Imprecision"]
A_Suvorexant_10mg:N_Daridorexant_50mg	0	No concerns	Low risk	No concerns	No concerns	Major concerns	No concerns	Low	["Heterogeneity"]
B_Suvorexant_20mg:G_Lemborexant_5mg	0	No concerns	Low risk	No concerns	No concerns	No concerns	No concerns	High	[]
B_Suvorexant_20mg:H_Lemborexant_10mg	0	No concerns	Low risk	No concerns	No concerns	Some concerns	No concerns	Moderate	["Heterogeneity"]
B_Suvorexant_20mg:K_Daridorexant_5mg	0	No concerns	Low risk	No concerns	No concerns	No concerns	No concerns	High	[]
B_Suvorexant_20mg:L_Daridorexant_10mg	0	No concerns	Low risk	No concerns	No concerns	No concerns	No concerns	High	[]
B_Suvorexant_20mg:M_Daridorexant_25mg	0	No concerns	Low risk	No concerns	No concerns	No concerns	No concerns	High	[]
B_Suvorexant_20mg:N_Daridorexant_50mg	0	No concerns	Low risk	No concerns	No concerns	No concerns	No concerns	High	[]
C_Suvorexant_40mg:G_Lemborexant_5mg	0	No concerns	Low risk	No concerns	No concerns	Some concerns	No concerns	Moderate	["Heterogeneity"]
C_Suvorexant_40mg:H_Lemborexant_10mg	0	No concerns	Low risk	No concerns	No concerns	No concerns	No concerns	High	[]
C_Suvorexant_40mg:K_Daridorexant_5mg	0	No concerns	Low risk	No concerns	No concerns	No concerns	No concerns	High	[]

C_Suvorexant_40mg:L_Daridorexant_10mg	0	No concerns	Low risk	No concerns	No concerns	No concerns	No concerns	High	[]
C_Suvorexant_40mg:M_Daridorexant_25mg	0	No concerns	Low risk	No concerns	No concerns	No concerns	No concerns	High	[]
C_Suvorexant_40mg:N_Daridorexant_50mg	0	No concerns	Low risk	No concerns	No concerns	Some concerns	No concerns	Moderate	["Heterogeneity"]
D_Suvorexant_80mg:G_Lemborexant_5mg	0	No concerns	Low risk	No concerns	Some concerns	Some concerns	No concerns	Low	["Imprecision","Heterogeneity"]
D_Suvorexant_80mg:H_Lemborexant_10mg	0	No concerns	Low risk	No concerns	Some concerns	Some concerns	No concerns	Low	["Imprecision","Heterogeneity"]
D_Suvorexant_80mg:K_Daridorexant_5mg	0	No concerns	Low risk	No concerns	No concerns	No concerns	No concerns	High	[]
D_Suvorexant_80mg:L_Daridorexant_10mg	0	No concerns	Low risk	No concerns	No concerns	Some concerns	No concerns	Moderate	["Heterogeneity"]
D_Suvorexant_80mg:M_Daridorexant_25mg	0	No concerns	Low risk	No concerns	Some concerns	No concerns	No concerns	Moderate	["Imprecision"]
D_Suvorexant_80mg:N_Daridorexant_50mg	0	No concerns	Low risk	No concerns	Some concerns	No concerns	No concerns	Moderate	["Imprecision"]
G_Lemborexant_5mg:K_Daridorexant_5mg	0	No concerns	Low risk	No concerns	No concerns	No concerns	No concerns	High	[]
G_Lemborexant_5mg:L_Daridorexant_10mg	0	No concerns	Low risk	No concerns	No concerns	No concerns	No concerns	High	[]
G_Lemborexant_5mg:M_Daridorexant_25mg	0	No concerns	Low risk	No concerns	No concerns	Some concerns	No concerns	Moderate	["Heterogeneity"]
G_Lemborexant_5mg:N_Daridorexant_50mg	0	No concerns	Low risk	No concerns	No concerns	Some concerns	No concerns	Moderate	["Heterogeneity"]
H_Lemborexant_10mg:K_Daridorexant_5mg	0	No concerns	Low risk	No concerns	No concerns	No concerns	No concerns	High	[]
H_Lemborexant_10mg:L_Daridorexant_10mg	0	No concerns	Low risk	No concerns	No concerns	No concerns	No concerns	High	[]
H_Lemborexant_10mg:M_Daridorexant_25mg	0	No concerns	Low risk	No concerns	No concerns	Some concerns	No concerns	Moderate	["Heterogeneity"]
H_Lemborexant_10mg:N_Daridorexant_50mg	0	No concerns	Low risk	No concerns	Some concerns	No concerns	No concerns	Moderate	["Imprecision"]
**4. sWASO**									
B_Suvorexant_20mg:C_Suvorexant_40mg	4	No concerns	Low risk	No concerns	Some concerns	No concerns	No concerns	Moderate	["Imprecision"]
B_Suvorexant_20mg:O_Placebo	4	No concerns	Low risk	No concerns	No concerns	No concerns	No concerns	High	[]
C_Suvorexant_40mg:O_Placebo	6	No concerns	Low risk	No concerns	No concerns	No concerns	No concerns	High	[]
G_Lemborexant_5mg:H_Lemborexant_10mg	2	Some concerns	Low risk	No concerns	No concerns	Some concerns	No concerns	Low	["Within-study bias","Heterogeneity"]
G_Lemborexant_5mg:O_Placebo	2	Some concerns	Low risk	No concerns	No concerns	No concerns	No concerns	Moderate	["Within-study bias"]
H_Lemborexant_10mg:O_Placebo	2	Some concerns	Low risk	No concerns	No concerns	No concerns	No concerns	Moderate	["Within-study bias"]
K_Daridorexant_5mg:L_Daridorexant_10mg	2	No concerns	Low risk	No concerns	No concerns	No concerns	No concerns	High	[]
K_Daridorexant_5mg:M_Daridorexant_25mg	2	No concerns	Low risk	No concerns	No concerns	Some concerns	No concerns	Moderate	["Heterogeneity"]
K_Daridorexant_5mg:N_Daridorexant_50mg	2	No concerns	Low risk	No concerns	No concerns	No concerns	No concerns	High	[]
K_Daridorexant_5mg:O_Placebo	2	No concerns	Low risk	No concerns	No concerns	Some concerns	No concerns	Moderate	["Heterogeneity"]
L_Daridorexant_10mg:M_Daridorexant_25mg	2	No concerns	Low risk	No concerns	No concerns	No concerns	No concerns	High	[]
L_Daridorexant_10mg:N_Daridorexant_50mg	2	No concerns	Low risk	No concerns	No concerns	No concerns	No concerns	High	[]
L_Daridorexant_10mg:O_Placebo	2	No concerns	Low risk	No concerns	No concerns	Some concerns	No concerns	Moderate	["Heterogeneity"]

M_Daridorexant_25mg:N_Daridorexant_50mg	2	No concerns	Low risk	No concerns	No concerns	Some concerns	No concerns	Moderate	["Heterogeneity"]
M_Daridorexant_25mg:O_Placebo	2	No concerns	Low risk	No concerns	No concerns	No concerns	No concerns	High	[]
N_Daridorexant_50mg:O_Placebo	2	No concerns	Low risk	No concerns	No concerns	No concerns	No concerns	High	[]
B_Suvorexant_20mg:G_Lemborexant_5mg	0	No concerns	Low risk	No concerns	No concerns	No concerns	No concerns	High	[]
B_Suvorexant_20mg:H_Lemborexant_10mg	0	No concerns	Low risk	No concerns	No concerns	No concerns	No concerns	High	[]
B_Suvorexant_20mg:K_Daridorexant_5mg	0	No concerns	Low risk	No concerns	Some concerns	No concerns	No concerns	Moderate	["Imprecision"]
B_Suvorexant_20mg:L_Daridorexant_10mg	0	No concerns	Low risk	No concerns	No concerns	No concerns	No concerns	High	[]
B_Suvorexant_20mg:M_Daridorexant_25mg	0	No concerns	Low risk	No concerns	Some concerns	No concerns	No concerns	Moderate	["Imprecision"]
B_Suvorexant_20mg:N_Daridorexant_50mg	0	No concerns	Low risk	No concerns	No concerns	Some concerns	No concerns	Moderate	["Heterogeneity"]
C_Suvorexant_40mg:G_Lemborexant_5mg	0	Some concerns	Low risk	No concerns	No concerns	No concerns	No concerns	Moderate	["Within-study bias"]
C_Suvorexant_40mg:H_Lemborexant_10mg	0	Some concerns	Low risk	No concerns	No concerns	Some concerns	No concerns	Low	["Within-study bias","Heterogeneity"]
C_Suvorexant_40mg:K_Daridorexant_5mg	0	No concerns	Low risk	No concerns	No concerns	Some concerns	No concerns	Moderate	["Heterogeneity"]
C_Suvorexant_40mg:L_Daridorexant_10mg	0	No concerns	Low risk	No concerns	No concerns	No concerns	No concerns	High	[]
C_Suvorexant_40mg:M_Daridorexant_25mg	0	No concerns	Low risk	No concerns	No concerns	Major concerns	No concerns	Low	["Heterogeneity"]
C_Suvorexant_40mg:N_Daridorexant_50mg	0	No concerns	Low risk	No concerns	No concerns	Some concerns	No concerns	Moderate	["Heterogeneity"]
G_Lemborexant_5mg:K_Daridorexant_5mg	0	No concerns	Low risk	No concerns	No concerns	No concerns	No concerns	High	[]
G_Lemborexant_5mg:L_Daridorexant_10mg	0	No concerns	Low risk	No concerns	No concerns	No concerns	No concerns	High	[]
G_Lemborexant_5mg:M_Daridorexant_25mg	0	No concerns	Low risk	No concerns	No concerns	No concerns	No concerns	High	[]
G_Lemborexant_5mg:N_Daridorexant_50mg	0	No concerns	Low risk	No concerns	No concerns	Some concerns	No concerns	Moderate	["Heterogeneity"]
H_Lemborexant_10mg:K_Daridorexant_5mg	0	No concerns	Low risk	No concerns	No concerns	No concerns	No concerns	High	[]
H_Lemborexant_10mg:L_Daridorexant_10mg	0	No concerns	Low risk	No concerns	No concerns	No concerns	No concerns	High	[]
H_Lemborexant_10mg:M_Daridorexant_25mg	0	No concerns	Low risk	No concerns	No concerns	Some concerns	No concerns	Moderate	["Heterogeneity"]
H_Lemborexant_10mg:N_Daridorexant_50mg	0	No concerns	Low risk	No concerns	No concerns	Some concerns	No concerns	Moderate	["Heterogeneity"]
**5. TST**									
A_Suvorexant_10mg:B_Suvorexant_20mg	1	No concerns	Low risk	No concerns	Some concerns	Some concerns	Major concerns	Very low	["Imprecision","Heterogeneity","Incoherence"]
A_Suvorexant_10mg:C_Suvorexant_40mg	1	No concerns	Low risk	No concerns	Some concerns	Some concerns	Major concerns	Very low	["Imprecision","Heterogeneity","Incoherence"]
A_Suvorexant_10mg:D_Suvorexant_80mg	1	No concerns	Low risk	No concerns	Some concerns	Some concerns	Major concerns	Very low	["Imprecision","Heterogeneity","Incoherence"]
A_Suvorexant_10mg:O_Placebo	1	No concerns	Low risk	No concerns	Some concerns	Some concerns	Major concerns	Very low	["Imprecision","Heterogeneity","Incoherence"]
B_Suvorexant_20mg:C_Suvorexant_40mg	1	No concerns	Low risk	No concerns	Major concerns	No concerns	Major concerns	Very low	["Imprecision","Incoherence"]
B_Suvorexant_20mg:D_Suvorexant_80mg	1	No concerns	Low risk	No concerns	Major concerns	No concerns	Major concerns	Very low	["Imprecision","Incoherence"]
B_Suvorexant_20mg:O_Placebo	1	No concerns	Low risk	No concerns	No concerns	Some concerns	Major concerns	Very low	["Heterogeneity","Incoherence"]

C_Suvorexant_40mg:D_Suvorexant_80mg	1	No concerns	Low risk	No concerns	Major concerns	No concerns	Major concerns	Very low	["Imprecision","Incoherence"]
C_Suvorexant_40mg:O_Placebo	1	No concerns	Low risk	No concerns	No concerns	Major concerns	Major concerns	Very low	["Heterogeneity","Incoherence"]
D_Suvorexant_80mg:O_Placebo	1	No concerns	Low risk	No concerns	No concerns	Major concerns	Major concerns	Very low	["Heterogeneity","Incoherence"]
K_Daridorexant_5mg:L_Daridorexant_10mg	2	No concerns	Low risk	No concerns	No concerns	Major concerns	Major concerns	Very low	["Heterogeneity","Incoherence"]
K_Daridorexant_5mg:M_Daridorexant_25mg	2	No concerns	Low risk	No concerns	No concerns	Major concerns	Major concerns	Very low	["Heterogeneity","Incoherence"]
K_Daridorexant_5mg:N_Daridorexant_50mg	2	No concerns	Low risk	No concerns	No concerns	Some concerns	Major concerns	Very low	["Heterogeneity","Incoherence"]
K_Daridorexant_5mg:O_Placebo	2	No concerns	Low risk	No concerns	Major concerns	No concerns	Major concerns	Very low	["Imprecision","Incoherence"]
L_Daridorexant_10mg:M_Daridorexant_25mg	2	No concerns	Low risk	No concerns	Some concerns	Some concerns	Major concerns	Very low	["Imprecision","Heterogeneity","Incoherence"]
L_Daridorexant_10mg:N_Daridorexant_50mg	2	No concerns	Low risk	No concerns	Some concerns	Some concerns	Major concerns	Very low	["Imprecision","Heterogeneity","Incoherence"]
L_Daridorexant_10mg:O_Placebo	2	No concerns	Low risk	No concerns	No concerns	Major concerns	Major concerns	Very low	["Heterogeneity","Incoherence"]
M_Daridorexant_25mg:N_Daridorexant_50mg	2	No concerns	Low risk	No concerns	Some concerns	Some concerns	Major concerns	Very low	["Imprecision","Heterogeneity","Incoherence"]
M_Daridorexant_25mg:O_Placebo	2	No concerns	Low risk	No concerns	No concerns	Major concerns	Major concerns	Very low	["Heterogeneity","Incoherence"]
N_Daridorexant_50mg:O_Placebo	2	No concerns	Low risk	No concerns	No concerns	Some concerns	Major concerns	Very low	["Heterogeneity","Incoherence"]
A_Suvorexant_10mg:K_Daridorexant_5mg	0	No concerns	Low risk	No concerns	Some concerns	Some concerns	Major concerns	Very low	["Imprecision","Heterogeneity","Incoherence"]
A_Suvorexant_10mg:L_Daridorexant_10mg	0	No concerns	Low risk	No concerns	Major concerns	No concerns	Major concerns	Very low	["Imprecision","Incoherence"]
A_Suvorexant_10mg:M_Daridorexant_25mg	0	No concerns	Low risk	No concerns	Major concerns	No concerns	Major concerns	Very low	["Imprecision","Incoherence"]
A_Suvorexant_10mg:N_Daridorexant_50mg	0	No concerns	Low risk	No concerns	Major concerns	No concerns	Major concerns	Very low	["Imprecision","Incoherence"]
B_Suvorexant_20mg:K_Daridorexant_5mg	0	No concerns	Low risk	No concerns	No concerns	Major concerns	Major concerns	Very low	["Heterogeneity","Incoherence"]
B_Suvorexant_20mg:L_Daridorexant_10mg	0	No concerns	Low risk	No concerns	Some concerns	Some concerns	Major concerns	Very low	["Imprecision","Heterogeneity","Incoherence"]
B_Suvorexant_20mg:M_Daridorexant_25mg	0	No concerns	Low risk	No concerns	Some concerns	Some concerns	Major concerns	Very low	["Imprecision","Heterogeneity","Incoherence"]
B_Suvorexant_20mg:N_Daridorexant_50mg	0	No concerns	Low risk	No concerns	Major concerns	No concerns	Major concerns	Very low	["Imprecision","Incoherence"]
C_Suvorexant_40mg:K_Daridorexant_5mg	0	No concerns	Low risk	No concerns	Some concerns	Some concerns	Major concerns	Very low	["Imprecision","Heterogeneity","Incoherence"]
C_Suvorexant_40mg:L_Daridorexant_10mg	0	No concerns	Low risk	No concerns	Major concerns	No concerns	Major concerns	Very low	["Imprecision","Incoherence"]
C_Suvorexant_40mg:M_Daridorexant_25mg	0	No concerns	Low risk	No concerns	Major concerns	No concerns	Major concerns	Very low	["Imprecision","Incoherence"]
C_Suvorexant_40mg:N_Daridorexant_50mg	0	No concerns	Low risk	No concerns	Major concerns	No concerns	Major concerns	Very low	["Imprecision","Incoherence"]
D_Suvorexant_80mg:K_Daridorexant_5mg	0	No concerns	Low risk	No concerns	Some concerns	Some concerns	Major concerns	Very low	["Imprecision","Heterogeneity","Incoherence"]
D_Suvorexant_80mg:L_Daridorexant_10mg	0	No concerns	Low risk	No concerns	Major concerns	No concerns	Major concerns	Very low	["Imprecision","Incoherence"]
D_Suvorexant_80mg:M_Daridorexant_25mg	0	No concerns	Low risk	No concerns	Major concerns	No concerns	Major concerns	Very low	["Imprecision","Incoherence"]
D_Suvorexant_80mg:N_Daridorexant_50mg	0	No concerns	Low risk	No concerns	Major concerns	No concerns	Major concerns	Very low	["Imprecision","Incoherence"]
**6. sTST**									
A_Suvorexant_10mg:B_Suvorexant_20mg	1	No concerns	Low risk	No concerns	Some concerns	No concerns	Major concerns	Very low	["Imprecision","Incoherence"]

A_Suvorexant_10mg:C_Suvorexant_40mg	1	No concerns	Low risk	No concerns	No concerns	No concerns	No concerns	High	[]
A_Suvorexant_10mg:D_Suvorexant_80mg	1	No concerns	Low risk	No concerns	Some concerns	No concerns	Some concerns	Low	["Imprecision","Incoherence"]
A_Suvorexant_10mg:O_Placebo	1	No concerns	Low risk	No concerns	Some concerns	No concerns	No concerns	Moderate	["Imprecision"]
B_Suvorexant_20mg:C_Suvorexant_40mg	5	No concerns	Low risk	No concerns	No concerns	No concerns	No concerns	High	[]
B_Suvorexant_20mg:D_Suvorexant_80mg	1	No concerns	Low risk	No concerns	No concerns	Some concerns	Major concerns	Very low	["Heterogeneity","Incoherence"]
B_Suvorexant_20mg:O_Placebo	5	No concerns	Low risk	No concerns	No concerns	Some concerns	No concerns	Moderate	["Heterogeneity"]
C_Suvorexant_40mg:D_Suvorexant_80mg	1	No concerns	Low risk	No concerns	No concerns	No concerns	No concerns	High	[]
C_Suvorexant_40mg:O_Placebo	7	No concerns	Low risk	No concerns	No concerns	No concerns	Major concerns	Low	["Incoherence"]
D_Suvorexant_80mg:O_Placebo	1	No concerns	Low risk	No concerns	No concerns	No concerns	No concerns	High	[]
G_Lemborexant_5mg:H_Lemborexant_10mg	1	Some concerns	Low risk	No concerns	No concerns	No concerns	Some concerns	Low	["Within-study bias","Incoherence"]
G_Lemborexant_5mg:O_Placebo	1	Some concerns	Low risk	No concerns	No concerns	No concerns	Some concerns	Low	["Within-study bias","Incoherence"]
H_Lemborexant_10mg:O_Placebo	1	Some concerns	Low risk	No concerns	No concerns	No concerns	Some concerns	Low	["Within-study bias","Incoherence"]
K_Daridorexant_5mg:L_Daridorexant_10mg	2	No concerns	Low risk	No concerns	No concerns	No concerns	No concerns	High	[]
K_Daridorexant_5mg:M_Daridorexant_25mg	2	No concerns	Low risk	No concerns	No concerns	No concerns	No concerns	High	[]
K_Daridorexant_5mg:N_Daridorexant_50mg	2	No concerns	Low risk	No concerns	No concerns	No concerns	No concerns	High	[]
K_Daridorexant_5mg:O_Placebo	2	No concerns	Low risk	No concerns	No concerns	No concerns	No concerns	High	[]
L_Daridorexant_10mg:M_Daridorexant_25mg	4	No concerns	Low risk	No concerns	No concerns	No concerns	No concerns	High	[]
L_Daridorexant_10mg:N_Daridorexant_50mg	2	No concerns	Low risk	No concerns	No concerns	No concerns	No concerns	High	[]
L_Daridorexant_10mg:O_Placebo	4	No concerns	Low risk	No concerns	No concerns	No concerns	No concerns	High	[]
M_Daridorexant_25mg:N_Daridorexant_50mg	4	No concerns	Low risk	No concerns	No concerns	No concerns	No concerns	High	[]
M_Daridorexant_25mg:O_Placebo	6	No concerns	Low risk	No concerns	No concerns	No concerns	No concerns	High	[]
N_Daridorexant_50mg:O_Placebo	4	No concerns	Low risk	No concerns	No concerns	No concerns	No concerns	High	[]
A_Suvorexant_10mg:G_Lemborexant_5mg	0	No concerns	Low risk	No concerns	Some concerns	No concerns	Some concerns	Low	["Imprecision","Incoherence"]
A_Suvorexant_10mg:H_Lemborexant_10mg	0	No concerns	Low risk	No concerns	No concerns	No concerns	Some concerns	Moderate	["Incoherence"]
A_Suvorexant_10mg:K_Daridorexant_5mg	0	No concerns	Low risk	No concerns	Some concerns	No concerns	Some concerns	Low	["Imprecision","Incoherence"]
A_Suvorexant_10mg:L_Daridorexant_10mg	0	No concerns	Low risk	No concerns	No concerns	No concerns	Some concerns	Moderate	["Incoherence"]
A_Suvorexant_10mg:M_Daridorexant_25mg	0	No concerns	Low risk	No concerns	Some concerns	No concerns	Some concerns	Low	["Imprecision","Incoherence"]
A_Suvorexant_10mg:N_Daridorexant_50mg	0	No concerns	Low risk	No concerns	No concerns	No concerns	Some concerns	Moderate	["Incoherence"]
B_Suvorexant_20mg:G_Lemborexant_5mg	0	No concerns	Low risk	No concerns	No concerns	No concerns	Some concerns	Moderate	["Incoherence"]
B_Suvorexant_20mg:H_Lemborexant_10mg	0	No concerns	Low risk	No concerns	No concerns	No concerns	Some concerns	Moderate	["Incoherence"]
B_Suvorexant_20mg:K_Daridorexant_5mg	0	No concerns	Low risk	No concerns	No concerns	No concerns	Some concerns	Moderate	["Incoherence"]

B_Suvorexant_20mg:L_Daridorexant_10mg	0	No concerns	Low risk	No concerns	No concerns	No concerns	Some concerns	Moderate	["Incoherence"]
B_Suvorexant_20mg:M_Daridorexant_25mg	0	No concerns	Low risk	No concerns	No concerns	No concerns	Some concerns	Moderate	["Incoherence"]
B_Suvorexant_20mg:N_Daridorexant_50mg	0	No concerns	Low risk	No concerns	No concerns	No concerns	Some concerns	Moderate	["Incoherence"]
C_Suvorexant_40mg:G_Lemborexant_5mg	0	Some concerns	Low risk	No concerns	No concerns	No concerns	Some concerns	Low	["Within-study bias","Incoherence"]
C_Suvorexant_40mg:H_Lemborexant_10mg	0	Some concerns	Low risk	No concerns	No concerns	No concerns	Some concerns	Low	["Within-study bias","Incoherence"]
C_Suvorexant_40mg:K_Daridorexant_5mg	0	No concerns	Low risk	No concerns	No concerns	No concerns	Some concerns	Moderate	["Incoherence"]
C_Suvorexant_40mg:L_Daridorexant_10mg	0	No concerns	Low risk	No concerns	No concerns	No concerns	Some concerns	Moderate	["Incoherence"]
C_Suvorexant_40mg:M_Daridorexant_25mg	0	No concerns	Low risk	No concerns	No concerns	No concerns	Some concerns	Moderate	["Incoherence"]
C_Suvorexant_40mg:N_Daridorexant_50mg	0	No concerns	Low risk	No concerns	No concerns	No concerns	Some concerns	Moderate	["Incoherence"]
D_Suvorexant_80mg:G_Lemborexant_5mg	0	No concerns	Low risk	No concerns	No concerns	No concerns	Some concerns	Moderate	["Incoherence"]
D_Suvorexant_80mg:H_Lemborexant_10mg	0	No concerns	Low risk	No concerns	No concerns	No concerns	Some concerns	Moderate	["Incoherence"]
D_Suvorexant_80mg:K_Daridorexant_5mg	0	No concerns	Low risk	No concerns	No concerns	No concerns	Some concerns	Moderate	["Incoherence"]
D_Suvorexant_80mg:L_Daridorexant_10mg	0	No concerns	Low risk	No concerns	Some concerns	No concerns	Some concerns	Low	["Imprecision","Incoherence"]
D_Suvorexant_80mg:M_Daridorexant_25mg	0	No concerns	Low risk	No concerns	Some concerns	No concerns	Some concerns	Low	["Imprecision","Incoherence"]
D_Suvorexant_80mg:N_Daridorexant_50mg	0	No concerns	Low risk	No concerns	No concerns	No concerns	Some concerns	Moderate	["Incoherence"]
G_Lemborexant_5mg:K_Daridorexant_5mg	0	No concerns	Low risk	No concerns	No concerns	No concerns	Some concerns	Moderate	["Incoherence"]
G_Lemborexant_5mg:L_Daridorexant_10mg	0	No concerns	Low risk	No concerns	No concerns	No concerns	Some concerns	Moderate	["Incoherence"]
G_Lemborexant_5mg:M_Daridorexant_25mg	0	No concerns	Low risk	No concerns	No concerns	No concerns	Some concerns	Moderate	["Incoherence"]
G_Lemborexant_5mg:N_Daridorexant_50mg	0	No concerns	Low risk	No concerns	No concerns	No concerns	Some concerns	Moderate	["Incoherence"]
H_Lemborexant_10mg:K_Daridorexant_5mg	0	No concerns	Low risk	No concerns	No concerns	No concerns	Some concerns	Moderate	["Incoherence"]
H_Lemborexant_10mg:L_Daridorexant_10mg	0	No concerns	Low risk	No concerns	No concerns	No concerns	Some concerns	Moderate	["Incoherence"]
H_Lemborexant_10mg:M_Daridorexant_25mg	0	No concerns	Low risk	No concerns	No concerns	No concerns	Some concerns	Moderate	["Incoherence"]
H_Lemborexant_10mg:N_Daridorexant_50mg	0	No concerns	Low risk	No concerns	No concerns	No concerns	Some concerns	Moderate	["Incoherence"]
**7. ISI**									
B_Suvorexant_20mg:C_Suvorexant_40mg	4	No concerns	Low risk	No concerns	No concerns	No concerns	No concerns	High	[]
B_Suvorexant_20mg:O_Placebo	4	No concerns	Low risk	No concerns	No concerns	No concerns	No concerns	High	[]
C_Suvorexant_40mg:O_Placebo	6	No concerns	Low risk	No concerns	No concerns	No concerns	No concerns	High	[]
G_Lemborexant_5mg:H_Lemborexant_10mg	1	No concerns	Low risk	No concerns	No concerns	No concerns	No concerns	High	[]
G_Lemborexant_5mg:O_Placebo	1	No concerns	Low risk	No concerns	No concerns	Some concerns	No concerns	Moderate	["Heterogeneity"]
H_Lemborexant_10mg:O_Placebo	1	No concerns	Low risk	No concerns	No concerns	No concerns	No concerns	High	[]
K_Daridorexant_5mg:L_Daridorexant_10mg	1	No concerns	Low risk	No concerns	No concerns	Some concerns	No concerns	Moderate	["Heterogeneity"]

K_Daridorexant_5mg:M_Daridorexant_25mg	1	No concerns	Low risk	No concerns	No concerns	Some concerns	No concerns	Moderate	["Heterogeneity"]
K_Daridorexant_5mg:N_Daridorexant_50mg	1	No concerns	Low risk	No concerns	Some concerns	No concerns	No concerns	Moderate	["Imprecision"]
K_Daridorexant_5mg:O_Placebo	1	No concerns	Low risk	No concerns	No concerns	Some concerns	No concerns	Moderate	["Heterogeneity"]
L_Daridorexant_10mg:M_Daridorexant_25mg	1	No concerns	Low risk	No concerns	No concerns	No concerns	No concerns	High	[]
L_Daridorexant_10mg:N_Daridorexant_50mg	1	No concerns	Low risk	No concerns	No concerns	Some concerns	No concerns	Moderate	["Heterogeneity"]
L_Daridorexant_10mg:O_Placebo	1	No concerns	Low risk	No concerns	No concerns	Some concerns	No concerns	Moderate	["Heterogeneity"]
M_Daridorexant_25mg:N_Daridorexant_50mg	1	No concerns	Low risk	No concerns	No concerns	Some concerns	No concerns	Moderate	["Heterogeneity"]
M_Daridorexant_25mg:O_Placebo	1	No concerns	Low risk	No concerns	No concerns	No concerns	No concerns	High	[]
N_Daridorexant_50mg:O_Placebo	1	No concerns	Low risk	No concerns	No concerns	Some concerns	No concerns	Moderate	["Heterogeneity"]
B_Suvorexant_20mg:G_Lemborexant_5mg	0	No concerns	Low risk	No concerns	No concerns	No concerns	No concerns	High	[]
B_Suvorexant_20mg:H_Lemborexant_10mg	0	No concerns	Low risk	No concerns	No concerns	No concerns	No concerns	High	[]
B_Suvorexant_20mg:K_Daridorexant_5mg	0	No concerns	Low risk	No concerns	Some concerns	No concerns	No concerns	Moderate	["Imprecision"]
B_Suvorexant_20mg:L_Daridorexant_10mg	0	No concerns	Low risk	No concerns	No concerns	Some concerns	No concerns	Moderate	["Heterogeneity"]
B_Suvorexant_20mg:M_Daridorexant_25mg	0	No concerns	Low risk	No concerns	Some concerns	No concerns	No concerns	Moderate	["Imprecision"]
B_Suvorexant_20mg:N_Daridorexant_50mg	0	No concerns	Low risk	No concerns	No concerns	Some concerns	No concerns	Moderate	["Heterogeneity"]
C_Suvorexant_40mg:G_Lemborexant_5mg	0	No concerns	Low risk	No concerns	No concerns	No concerns	No concerns	High	[]
C_Suvorexant_40mg:H_Lemborexant_10mg	0	No concerns	Low risk	No concerns	No concerns	No concerns	No concerns	High	[]
C_Suvorexant_40mg:K_Daridorexant_5mg	0	No concerns	Low risk	No concerns	Some concerns	No concerns	No concerns	Moderate	["Imprecision"]
C_Suvorexant_40mg:L_Daridorexant_10mg	0	No concerns	Low risk	No concerns	Some concerns	No concerns	No concerns	Moderate	["Imprecision"]
C_Suvorexant_40mg:M_Daridorexant_25mg	0	No concerns	Low risk	No concerns	Some concerns	No concerns	No concerns	Moderate	["Imprecision"]
C_Suvorexant_40mg:N_Daridorexant_50mg	0	No concerns	Low risk	No concerns	Some concerns	No concerns	No concerns	Moderate	["Imprecision"]
G_Lemborexant_5mg:K_Daridorexant_5mg	0	No concerns	Low risk	No concerns	Some concerns	No concerns	No concerns	Moderate	["Imprecision"]
G_Lemborexant_5mg:L_Daridorexant_10mg	0	No concerns	Low risk	No concerns	Some concerns	No concerns	No concerns	Moderate	["Imprecision"]
G_Lemborexant_5mg:M_Daridorexant_25mg	0	No concerns	Low risk	No concerns	Some concerns	No concerns	No concerns	Moderate	["Imprecision"]
G_Lemborexant_5mg:N_Daridorexant_50mg	0	No concerns	Low risk	No concerns	Some concerns	No concerns	No concerns	Moderate	["Imprecision"]
H_Lemborexant_10mg:K_Daridorexant_5mg	0	No concerns	Low risk	No concerns	Some concerns	No concerns	No concerns	Moderate	["Imprecision"]
H_Lemborexant_10mg:L_Daridorexant_10mg	0	No concerns	Low risk	No concerns	Some concerns	No concerns	No concerns	Moderate	["Imprecision"]
H_Lemborexant_10mg:M_Daridorexant_25mg	0	No concerns	Low risk	No concerns	Some concerns	No concerns	No concerns	Moderate	["Imprecision"]
H_Lemborexant_10mg:N_Daridorexant_50mg	0	No concerns	Low risk	No concerns	Some concerns	No concerns	No concerns	Moderate	["Imprecision"]
**8. AEs**									
A_Suvorexant_10mg:B_Suvorexant_20mg	1	No concerns	Low risk	No concerns	Major concerns	No concerns	No concerns	Low	["Imprecision"]

A_Suvorexant_10mg:C_Suvorexant_40mg	1	No concerns	Low risk	No concerns	Major concerns	No concerns	No concerns	Low	["Imprecision"]
A_Suvorexant_10mg:D_Suvorexant_80mg	1	No concerns	Low risk	No concerns	No concerns	No concerns	No concerns	High	[]
A_Suvorexant_10mg:O_Placebo	1	No concerns	Low risk	No concerns	Major concerns	No concerns	No concerns	Low	["Imprecision"]
B_Suvorexant_20mg:C_Suvorexant_40mg	3	No concerns	Low risk	No concerns	No concerns	No concerns	No concerns	High	[]
B_Suvorexant_20mg:D_Suvorexant_80mg	1	No concerns	Low risk	No concerns	No concerns	No concerns	No concerns	High	[]
B_Suvorexant_20mg:O_Placebo	3	No concerns	Low risk	No concerns	No concerns	No concerns	No concerns	High	[]
C_Suvorexant_40mg:D_Suvorexant_80mg	1	No concerns	Low risk	No concerns	No concerns	Some concerns	No concerns	Moderate	["Heterogeneity"]
C_Suvorexant_40mg:O_Placebo	4	No concerns	Low risk	No concerns	No concerns	No concerns	No concerns	High	[]
D_Suvorexant_80mg:O_Placebo	1	No concerns	Low risk	No concerns	No concerns	No concerns	No concerns	High	[]
E_Lemborexant_1mg:F_Lemborexant_2_5mg	1	Some concerns	Low risk	No concerns	Major concerns	No concerns	No concerns	Very low	["Within-study bias","Imprecision"]
E_Lemborexant_1mg:G_Lemborexant_5mg	1	Some concerns	Low risk	No concerns	Some concerns	Some concerns	No concerns	Very low	["Within-study bias","Imprecision","Heterogeneity"]
E_Lemborexant_1mg:H_Lemborexant_10mg	1	Some concerns	Low risk	No concerns	Some concerns	Some concerns	Some concerns	Very low	["Within-study bias","Imprecision","Heterogeneity","Incoherence"]
E_Lemborexant_1mg:I_Lemborexant_15mg	1	Some concerns	Low risk	No concerns	Some concerns	No concerns	No concerns	Low	["Within-study bias","Imprecision"]
E_Lemborexant_1mg:J_Lemborexant_25mg	1	Some concerns	Low risk	No concerns	No concerns	Some concerns	No concerns	Low	["Within-study bias","Heterogeneity"]
E_Lemborexant_1mg:O_Placebo	1	Some concerns	Low risk	No concerns	Some concerns	Some concerns	No concerns	Very low	["Within-study bias","Imprecision","Heterogeneity"]
F_Lemborexant_2_5mg:G_Lemborexant_5mg	1	Some concerns	Low risk	No concerns	Major concerns	No concerns	No concerns	Very low	["Within-study bias","Imprecision"]
F_Lemborexant_2_5mg:H_Lemborexant_10mg	1	Some concerns	Low risk	No concerns	Major concerns	No concerns	Major concerns	Very low	["Within-study bias","Imprecision","Incoherence"]
F_Lemborexant_2_5mg:I_Lemborexant_15mg	1	Some concerns	Low risk	No concerns	Some concerns	Some concerns	No concerns	Very low	["Within-study bias","Imprecision","Heterogeneity"]
F_Lemborexant_2_5mg:J_Lemborexant_25mg	1	Some concerns	Low risk	No concerns	Some concerns	No concerns	No concerns	Low	["Within-study bias","Imprecision"]
F_Lemborexant_2_5mg:O_Placebo	1	Some concerns	Low risk	No concerns	Major concerns	No concerns	No concerns	Very low	["Within-study bias","Imprecision"]
G_Lemborexant_5mg:H_Lemborexant_10mg	3	Some concerns	Low risk	No concerns	No concerns	No concerns	Some concerns	Low	["Within-study bias","Incoherence"]
G_Lemborexant_5mg:I_Lemborexant_15mg	1	Some concerns	Low risk	No concerns	Some concerns	No concerns	No concerns	Low	["Within-study bias","Imprecision"]
G_Lemborexant_5mg:J_Lemborexant_25mg	1	Some concerns	Low risk	No concerns	Some concerns	No concerns	No concerns	Low	["Within-study bias","Imprecision"]
G_Lemborexant_5mg:O_Placebo	3	Some concerns	Low risk	No concerns	No concerns	No concerns	No concerns	Moderate	["Within-study bias"]

H_Lemborexant_10mg:I_Lemborexant_15mg	1	Some concerns	Low risk	No concerns	Some concerns	No concerns	Some concerns	Very low	["Within-study bias","Imprecision","Incoherence"]
H_Lemborexant_10mg:J_Lemborexant_25mg	1	Some concerns	Low risk	No concerns	Some concerns	No concerns	Some concerns	Very low	["Within-study bias","Imprecision","Incoherence"]
H_Lemborexant_10mg:O_Placebo	3	Some concerns	Low risk	No concerns	No concerns	No concerns	No concerns	Moderate	["Within-study bias"]
I_Lemborexant_15mg:J_Lemborexant_25mg	1	Some concerns	Low risk	No concerns	Major concerns	No concerns	No concerns	Very low	["Within-study bias","Imprecision"]
I_Lemborexant_15mg:O_Placebo	1	Some concerns	Low risk	No concerns	Some concerns	No concerns	No concerns	Low	["Within-study bias","Imprecision"]
J_Lemborexant_25mg:O_Placebo	1	Some concerns	Low risk	No concerns	Some concerns	No concerns	No concerns	Low	["Within-study bias","Imprecision"]
K_Daridorexant_5mg:L_Daridorexant_10mg	2	No concerns	Low risk	No concerns	Major concerns	No concerns	No concerns	Low	["Imprecision"]
K_Daridorexant_5mg:M_Daridorexant_25mg	2	No concerns	Low risk	No concerns	Major concerns	No concerns	No concerns	Low	["Imprecision"]
K_Daridorexant_5mg:N_Daridorexant_50mg	2	No concerns	Low risk	No concerns	Major concerns	No concerns	No concerns	Low	["Imprecision"]
K_Daridorexant_5mg:O_Placebo	2	No concerns	Low risk	No concerns	Some concerns	Some concerns	No concerns	Low	["Imprecision","Heterogeneity"]
L_Daridorexant_10mg:M_Daridorexant_25mg	3	No concerns	Low risk	No concerns	No concerns	No concerns	No concerns	High	[]
L_Daridorexant_10mg:N_Daridorexant_50mg	2	No concerns	Low risk	No concerns	No concerns	Some concerns	No concerns	Moderate	["Heterogeneity"]
L_Daridorexant_10mg:O_Placebo	3	No concerns	Low risk	No concerns	Some concerns	No concerns	No concerns	Moderate	["Imprecision"]
M_Daridorexant_25mg:N_Daridorexant_50mg	3	No concerns	Low risk	No concerns	No concerns	No concerns	No concerns	High	[]
M_Daridorexant_25mg:O_Placebo	4	No concerns	Low risk	No concerns	No concerns	No concerns	No concerns	High	[]
N_Daridorexant_50mg:O_Placebo	3	No concerns	Low risk	No concerns	Some concerns	No concerns	No concerns	Moderate	["Imprecision"]
A_Suvorexant_10mg:E_Lemborexant_1mg	0	Some concerns	Low risk	No concerns	Major concerns	No concerns	No concerns	Very low	["Within-study bias","Imprecision"]
A_Suvorexant_10mg:F_Lemborexant_2_5mg	0	Some concerns	Low risk	No concerns	Major concerns	No concerns	No concerns	Very low	["Within-study bias","Imprecision"]
A_Suvorexant_10mg:G_Lemborexant_5mg	0	No concerns	Low risk	No concerns	Major concerns	No concerns	No concerns	Low	["Imprecision"]
A_Suvorexant_10mg:H_Lemborexant_10mg	0	No concerns	Low risk	No concerns	Major concerns	No concerns	No concerns	Low	["Imprecision"]
A_Suvorexant_10mg:I_Lemborexant_15mg	0	Some concerns	Low risk	No concerns	Major concerns	No concerns	No concerns	Very low	["Within-study bias","Imprecision"]
A_Suvorexant_10mg:J_Lemborexant_25mg	0	Some concerns	Low risk	No concerns	Some concerns	Some concerns	No concerns	Very low	["Within-study bias","Imprecision","Heterogeneity"]
A_Suvorexant_10mg:K_Daridorexant_5mg	0	No concerns	Low risk	No concerns	Major concerns	No concerns	No concerns	Low	["Imprecision"]
A_Suvorexant_10mg:L_Daridorexant_10mg	0	No concerns	Low risk	No concerns	Major concerns	No concerns	No concerns	Low	["Imprecision"]
A_Suvorexant_10mg:M_Daridorexant_25mg	0	No concerns	Low risk	No concerns	Major concerns	No concerns	No concerns	Low	["Imprecision"]
A_Suvorexant_10mg:N_Daridorexant_50mg	0	No concerns	Low risk	No concerns	Major concerns	No concerns	No concerns	Low	["Imprecision"]
B_Suvorexant_20mg:E_Lemborexant_1mg	0	Some concerns	Low risk	No concerns	Major concerns	No concerns	No concerns	Very low	["Within-study bias","Imprecision"]
B_Suvorexant_20mg:F_Lemborexant_2_5mg	0	Some concerns	Low risk	No concerns	Major concerns	No concerns	No concerns	Very low	["Within-study bias","Imprecision"]

B_Suvorexant_20mg:G_Lemborexant_5mg	0	No concerns	Low risk	No concerns	No concerns	No concerns	No concerns	High	[]
B_Suvorexant_20mg:H_Lemborexant_10mg	0	No concerns	Low risk	No concerns	No concerns	No concerns	No concerns	High	[]
B_Suvorexant_20mg:I_Lemborexant_15mg	0	Some concerns	Low risk	No concerns	Some concerns	No concerns	No concerns	Low	["Within-study bias","Imprecision"]
B_Suvorexant_20mg:J_Lemborexant_25mg	0	Some concerns	Low risk	No concerns	Some concerns	No concerns	No concerns	Low	["Within-study bias","Imprecision"]
B_Suvorexant_20mg:K_Daridorexant_5mg	0	No concerns	Low risk	No concerns	Some concerns	Some concerns	No concerns	Low	["Imprecision","Heterogeneity"]
B_Suvorexant_20mg:L_Daridorexant_10mg	0	No concerns	Low risk	No concerns	Some concerns	No concerns	No concerns	Moderate	["Imprecision"]
B_Suvorexant_20mg:M_Daridorexant_25mg	0	No concerns	Low risk	No concerns	Some concerns	No concerns	No concerns	Moderate	["Imprecision"]
B_Suvorexant_20mg:N_Daridorexant_50mg	0	No concerns	Low risk	No concerns	Some concerns	No concerns	No concerns	Moderate	["Imprecision"]
C_Suvorexant_40mg:E_Lemborexant_1mg	0	Some concerns	Low risk	No concerns	Some concerns	No concerns	No concerns	Low	["Within-study bias","Imprecision"]
C_Suvorexant_40mg:F_Lemborexant_2_5mg	0	Some concerns	Low risk	No concerns	Major concerns	No concerns	No concerns	Very low	["Within-study bias","Imprecision"]
C_Suvorexant_40mg:G_Lemborexant_5mg	0	No concerns	Low risk	No concerns	Some concerns	No concerns	No concerns	Moderate	["Imprecision"]
C_Suvorexant_40mg:H_Lemborexant_10mg	0	No concerns	Low risk	No concerns	No concerns	No concerns	No concerns	High	[]
C_Suvorexant_40mg:I_Lemborexant_15mg	0	Some concerns	Low risk	No concerns	Major concerns	No concerns	No concerns	Very low	["Within-study bias","Imprecision"]
C_Suvorexant_40mg:J_Lemborexant_25mg	0	Some concerns	Low risk	No concerns	Some concerns	No concerns	No concerns	Low	["Within-study bias","Imprecision"]
C_Suvorexant_40mg:K_Daridorexant_5mg	0	No concerns	Low risk	No concerns	Major concerns	No concerns	No concerns	Low	["Imprecision"]
C_Suvorexant_40mg:L_Daridorexant_10mg	0	No concerns	Low risk	No concerns	Some concerns	No concerns	No concerns	Moderate	["Imprecision"]
C_Suvorexant_40mg:M_Daridorexant_25mg	0	No concerns	Low risk	No concerns	Some concerns	No concerns	No concerns	Moderate	["Imprecision"]
C_Suvorexant_40mg:N_Daridorexant_50mg	0	No concerns	Low risk	No concerns	Some concerns	No concerns	No concerns	Moderate	["Imprecision"]
D_Suvorexant_80mg:E_Lemborexant_1mg	0	Some concerns	Low risk	No concerns	No concerns	No concerns	No concerns	Moderate	["Within-study bias"]
D_Suvorexant_80mg:F_Lemborexant_2_5mg	0	Some concerns	Low risk	No concerns	No concerns	Some concerns	No concerns	Low	["Within-study bias","Heterogeneity"]
D_Suvorexant_80mg:G_Lemborexant_5mg	0	No concerns	Low risk	No concerns	No concerns	No concerns	No concerns	High	[]
D_Suvorexant_80mg:H_Lemborexant_10mg	0	No concerns	Low risk	No concerns	No concerns	No concerns	No concerns	High	[]
D_Suvorexant_80mg:I_Lemborexant_15mg	0	Some concerns	Low risk	No concerns	Some concerns	No concerns	No concerns	Low	["Within-study bias","Imprecision"]
D_Suvorexant_80mg:J_Lemborexant_25mg	0	Some concerns	Low risk	No concerns	Major concerns	No concerns	No concerns	Very low	["Within-study bias","Imprecision"]
D_Suvorexant_80mg:K_Daridorexant_5mg	0	No concerns	Low risk	No concerns	Some concerns	No concerns	No concerns	Moderate	["Imprecision"]
D_Suvorexant_80mg:L_Daridorexant_10mg	0	No concerns	Low risk	No concerns	Some concerns	No concerns	No concerns	Moderate	["Imprecision"]
D_Suvorexant_80mg:M_Daridorexant_25mg	0	No concerns	Low risk	No concerns	Some concerns	No concerns	No concerns	Moderate	["Imprecision"]
D_Suvorexant_80mg:N_Daridorexant_50mg	0	No concerns	Low risk	No concerns	Some concerns	No concerns	No concerns	Moderate	["Imprecision"]
E_Lemborexant_1mg:K_Daridorexant_5mg	0	Some concerns	Low risk	No concerns	Some concerns	No concerns	No concerns	Low	["Within-study bias","Imprecision"]
E_Lemborexant_1mg:L_Daridorexant_10mg	0	Some concerns	Low risk	No concerns	Some concerns	No concerns	No concerns	Low	["Within-study bias","Imprecision"]
E_Lemborexant_1mg:M_Daridorexant_25mg	0	Some concerns	Low risk	No concerns	Some concerns	No concerns	No concerns	Low	["Within-study bias","Imprecision"]

E_Lemborexant_1mg:N_Daridorexant_50mg	0	Some concerns	Low risk	No concerns	Some concerns	No concerns	No concerns	Low	["Within-study bias","Imprecision"]
F_Lemborexant_2_5mg:K_Daridorexant_5mg	0	Some concerns	Low risk	No concerns	Major concerns	No concerns	No concerns	Very low	["Within-study bias","Imprecision"]
F_Lemborexant_2_5mg:L_Daridorexant_10mg	0	Some concerns	Low risk	No concerns	Major concerns	No concerns	No concerns	Very low	["Within-study bias","Imprecision"]
F_Lemborexant_2_5mg:M_Daridorexant_25mg	0	Some concerns	Low risk	No concerns	Major concerns	No concerns	No concerns	Very low	["Within-study bias","Imprecision"]
F_Lemborexant_2_5mg:N_Daridorexant_50mg	0	Some concerns	Low risk	No concerns	Major concerns	No concerns	No concerns	Very low	["Within-study bias","Imprecision"]
G_Lemborexant_5mg:K_Daridorexant_5mg	0	No concerns	Low risk	No concerns	Some concerns	Some concerns	No concerns	Low	["Imprecision","Heterogeneity"]
G_Lemborexant_5mg:L_Daridorexant_10mg	0	No concerns	Low risk	No concerns	Some concerns	No concerns	No concerns	Moderate	["Imprecision"]
G_Lemborexant_5mg:M_Daridorexant_25mg	0	No concerns	Low risk	No concerns	Some concerns	No concerns	No concerns	Moderate	["Imprecision"]
G_Lemborexant_5mg:N_Daridorexant_50mg	0	No concerns	Low risk	No concerns	Some concerns	No concerns	No concerns	Moderate	["Imprecision"]
H_Lemborexant_10mg:K_Daridorexant_5mg	0	No concerns	Low risk	No concerns	Major concerns	No concerns	No concerns	Low	["Imprecision"]
H_Lemborexant_10mg:L_Daridorexant_10mg	0	No concerns	Low risk	No concerns	Some concerns	No concerns	No concerns	Moderate	["Imprecision"]
H_Lemborexant_10mg:M_Daridorexant_25mg	0	No concerns	Low risk	No concerns	Some concerns	No concerns	No concerns	Moderate	["Imprecision"]
H_Lemborexant_10mg:N_Daridorexant_50mg	0	No concerns	Low risk	No concerns	Some concerns	No concerns	No concerns	Moderate	["Imprecision"]
I_Lemborexant_15mg:K_Daridorexant_5mg	0	Some concerns	Low risk	No concerns	Major concerns	No concerns	No concerns	Very low	["Within-study bias","Imprecision"]
I_Lemborexant_15mg:L_Daridorexant_10mg	0	Some concerns	Low risk	No concerns	Major concerns	No concerns	No concerns	Very low	["Within-study bias","Imprecision"]
I_Lemborexant_15mg:M_Daridorexant_25mg	0	Some concerns	Low risk	No concerns	Major concerns	No concerns	No concerns	Very low	["Within-study bias","Imprecision"]
I_Lemborexant_15mg:N_Daridorexant_50mg	0	Some concerns	Low risk	No concerns	Major concerns	No concerns	No concerns	Very low	["Within-study bias","Imprecision"]
J_Lemborexant_25mg:K_Daridorexant_5mg	0	Some concerns	Low risk	No concerns	Major concerns	No concerns	No concerns	Very low	["Within-study bias","Imprecision"]
J_Lemborexant_25mg:L_Daridorexant_10mg	0	Some concerns	Low risk	No concerns	Major concerns	No concerns	No concerns	Very low	["Within-study bias","Imprecision"]
J_Lemborexant_25mg:M_Daridorexant_25mg	0	Some concerns	Low risk	No concerns	Major concerns	No concerns	No concerns	Very low	["Within-study bias","Imprecision"]
J_Lemborexant_25mg:N_Daridorexant_50mg	0	Some concerns	Low risk	No concerns	Major concerns	No concerns	No concerns	Very low	["Within-study bias","Imprecision"]
**9. SAEs**									
A_Suvorexant_10mg:B_Suvorexant_20mg	1	No concerns	Low risk	No concerns	Major concerns	No concerns	No concerns	Low	["Imprecision"]
A_Suvorexant_10mg:C_Suvorexant_40mg	1	No concerns	Low risk	No concerns	Major concerns	No concerns	No concerns	Low	["Imprecision"]
A_Suvorexant_10mg:D_Suvorexant_80mg	1	No concerns	Low risk	No concerns	Major concerns	No concerns	No concerns	Low	["Imprecision"]
A_Suvorexant_10mg:O_Placebo	1	No concerns	Low risk	No concerns	Major concerns	No concerns	No concerns	Low	["Imprecision"]
B_Suvorexant_20mg:C_Suvorexant_40mg	3	No concerns	Low risk	No concerns	Major concerns	No concerns	No concerns	Low	["Imprecision"]
B_Suvorexant_20mg:D_Suvorexant_80mg	1	No concerns	Low risk	No concerns	Major concerns	No concerns	No concerns	Low	["Imprecision"]
B_Suvorexant_20mg:O_Placebo	3	No concerns	Low risk	No concerns	Some concerns	Some concerns	No concerns	Low	["Imprecision","Heterogeneity"]
C_Suvorexant_40mg:D_Suvorexant_80mg	1	No concerns	Low risk	No concerns	Major concerns	No concerns	No concerns	Low	["Imprecision"]
C_Suvorexant_40mg:O_Placebo	4	No concerns	Low risk	No concerns	Major concerns	No concerns	No concerns	Low	["Imprecision"]

D_Suvorexant_80mg:O_Placebo	1	No concerns	Low risk	No concerns	Major concerns	No concerns	No concerns	Low	["Imprecision"]
G_Lemborexant_5mg:H_Lemborexant_10mg	2	Some concerns	Low risk	No concerns	Major concerns	No concerns	No concerns	Very low	["Within-study bias","Imprecision"]
G_Lemborexant_5mg:O_Placebo	2	Some concerns	Low risk	No concerns	Major concerns	No concerns	No concerns	Very low	["Within-study bias","Imprecision"]
H_Lemborexant_10mg:O_Placebo	2	Some concerns	Low risk	No concerns	Major concerns	No concerns	No concerns	Very low	["Within-study bias","Imprecision"]
J_Lemborexant_25mg:O_Placebo	1	Some concerns	Low risk	No concerns	Major concerns	No concerns	No concerns	Very low	["Within-study bias","Imprecision"]
K_Daridorexant_5mg:L_Daridorexant_10mg	1	No concerns	Low risk	No concerns	Major concerns	No concerns	No concerns	Low	["Imprecision"]
K_Daridorexant_5mg:M_Daridorexant_25mg	1	No concerns	Low risk	No concerns	Major concerns	No concerns	No concerns	Low	["Imprecision"]
K_Daridorexant_5mg:N_Daridorexant_50mg	1	No concerns	Low risk	No concerns	Major concerns	No concerns	No concerns	Low	["Imprecision"]
K_Daridorexant_5mg:O_Placebo	1	No concerns	Low risk	No concerns	Major concerns	No concerns	No concerns	Low	["Imprecision"]
L_Daridorexant_10mg:M_Daridorexant_25mg	2	No concerns	Low risk	No concerns	Major concerns	No concerns	No concerns	Low	["Imprecision"]
L_Daridorexant_10mg:N_Daridorexant_50mg	1	No concerns	Low risk	No concerns	Major concerns	No concerns	No concerns	Low	["Imprecision"]
L_Daridorexant_10mg:O_Placebo	2	No concerns	Low risk	No concerns	Major concerns	No concerns	No concerns	Low	["Imprecision"]
M_Daridorexant_25mg:N_Daridorexant_50mg	2	No concerns	Low risk	No concerns	Major concerns	No concerns	No concerns	Low	["Imprecision"]
M_Daridorexant_25mg:O_Placebo	3	No concerns	Low risk	No concerns	Major concerns	No concerns	No concerns	Low	["Imprecision"]
N_Daridorexant_50mg:O_Placebo	2	No concerns	Low risk	No concerns	Major concerns	No concerns	No concerns	Low	["Imprecision"]
A_Suvorexant_10mg:G_Lemborexant_5mg	0	No concerns	Low risk	No concerns	Major concerns	No concerns	No concerns	Low	["Imprecision"]
A_Suvorexant_10mg:H_Lemborexant_10mg	0	No concerns	Low risk	No concerns	Major concerns	No concerns	No concerns	Low	["Imprecision"]
A_Suvorexant_10mg:J_Lemborexant_25mg	0	No concerns	Low risk	No concerns	Major concerns	No concerns	No concerns	Low	["Imprecision"]
A_Suvorexant_10mg:K_Daridorexant_5mg	0	No concerns	Low risk	No concerns	Major concerns	No concerns	No concerns	Low	["Imprecision"]
A_Suvorexant_10mg:L_Daridorexant_10mg	0	No concerns	Low risk	No concerns	Major concerns	No concerns	No concerns	Low	["Imprecision"]
A_Suvorexant_10mg:M_Daridorexant_25mg	0	No concerns	Low risk	No concerns	Major concerns	No concerns	No concerns	Low	["Imprecision"]
A_Suvorexant_10mg:N_Daridorexant_50mg	0	No concerns	Low risk	No concerns	Major concerns	No concerns	No concerns	Low	["Imprecision"]
B_Suvorexant_20mg:G_Lemborexant_5mg	0	No concerns	Low risk	No concerns	Some concerns	Some concerns	No concerns	Low	["Imprecision","Heterogeneity"]
B_Suvorexant_20mg:H_Lemborexant_10mg	0	No concerns	Low risk	No concerns	Some concerns	Some concerns	No concerns	Low	["Imprecision","Heterogeneity"]
B_Suvorexant_20mg:J_Lemborexant_25mg	0	No concerns	Low risk	No concerns	Major concerns	No concerns	No concerns	Low	["Imprecision"]
B_Suvorexant_20mg:K_Daridorexant_5mg	0	No concerns	Low risk	No concerns	Major concerns	No concerns	No concerns	Low	["Imprecision"]
B_Suvorexant_20mg:L_Daridorexant_10mg	0	No concerns	Low risk	No concerns	Major concerns	No concerns	No concerns	Low	["Imprecision"]
B_Suvorexant_20mg:M_Daridorexant_25mg	0	No concerns	Low risk	No concerns	Major concerns	No concerns	No concerns	Low	["Imprecision"]
B_Suvorexant_20mg:N_Daridorexant_50mg	0	No concerns	Low risk	No concerns	Major concerns	No concerns	No concerns	Low	["Imprecision"]
C_Suvorexant_40mg:G_Lemborexant_5mg	0	No concerns	Low risk	No concerns	Major concerns	No concerns	No concerns	Low	["Imprecision"]
C_Suvorexant_40mg:H_Lemborexant_10mg	0	Some concerns	Low risk	No concerns	Major concerns	No concerns	No concerns	Very low	["Within-study bias","Imprecision"]

C_Suvorexant_40mg:J_Lemborexant_25mg	0	No concerns	Low risk	No concerns	Major concerns	No concerns	No concerns	Low	["Imprecision"]
C_Suvorexant_40mg:K_Daridorexant_5mg	0	No concerns	Low risk	No concerns	Major concerns	No concerns	No concerns	Low	["Imprecision"]
C_Suvorexant_40mg:L_Daridorexant_10mg	0	No concerns	Low risk	No concerns	Major concerns	No concerns	No concerns	Low	["Imprecision"]
C_Suvorexant_40mg:M_Daridorexant_25mg	0	No concerns	Low risk	No concerns	Major concerns	No concerns	No concerns	Low	["Imprecision"]
C_Suvorexant_40mg:N_Daridorexant_50mg	0	No concerns	Low risk	No concerns	Major concerns	No concerns	No concerns	Low	["Imprecision"]
D_Suvorexant_80mg:G_Lemborexant_5mg	0	No concerns	Low risk	No concerns	Major concerns	No concerns	No concerns	Low	["Imprecision"]
D_Suvorexant_80mg:H_Lemborexant_10mg	0	No concerns	Low risk	No concerns	Major concerns	No concerns	No concerns	Low	["Imprecision"]
D_Suvorexant_80mg:J_Lemborexant_25mg	0	No concerns	Low risk	No concerns	Major concerns	No concerns	No concerns	Low	["Imprecision"]
D_Suvorexant_80mg:K_Daridorexant_5mg	0	No concerns	Low risk	No concerns	Major concerns	No concerns	No concerns	Low	["Imprecision"]
D_Suvorexant_80mg:L_Daridorexant_10mg	0	No concerns	Low risk	No concerns	Major concerns	No concerns	No concerns	Low	["Imprecision"]
D_Suvorexant_80mg:M_Daridorexant_25mg	0	No concerns	Low risk	No concerns	Major concerns	No concerns	No concerns	Low	["Imprecision"]
D_Suvorexant_80mg:N_Daridorexant_50mg	0	No concerns	Low risk	No concerns	Major concerns	No concerns	No concerns	Low	["Imprecision"]
G_Lemborexant_5mg:J_Lemborexant_25mg	0	Some concerns	Low risk	No concerns	Major concerns	No concerns	No concerns	Very low	["Within-study bias","Imprecision"]
G_Lemborexant_5mg:K_Daridorexant_5mg	0	No concerns	Low risk	No concerns	Major concerns	No concerns	No concerns	Low	["Imprecision"]
G_Lemborexant_5mg:L_Daridorexant_10mg	0	No concerns	Low risk	No concerns	Major concerns	No concerns	No concerns	Low	["Imprecision"]
G_Lemborexant_5mg:M_Daridorexant_25mg	0	No concerns	Low risk	No concerns	Some concerns	Some concerns	No concerns	Low	["Imprecision","Heterogeneity"]
G_Lemborexant_5mg:N_Daridorexant_50mg	0	No concerns	Low risk	No concerns	Major concerns	No concerns	No concerns	Low	["Imprecision"]
H_Lemborexant_10mg:J_Lemborexant_25mg	0	Some concerns	Low risk	No concerns	Major concerns	No concerns	No concerns	Very low	["Within-study bias","Imprecision"]
H_Lemborexant_10mg:K_Daridorexant_5mg	0	No concerns	Low risk	No concerns	Major concerns	No concerns	No concerns	Low	["Imprecision"]
H_Lemborexant_10mg:L_Daridorexant_10mg	0	No concerns	Low risk	No concerns	Major concerns	No concerns	No concerns	Low	["Imprecision"]
H_Lemborexant_10mg:M_Daridorexant_25mg	0	No concerns	Low risk	No concerns	Some concerns	Some concerns	No concerns	Low	["Imprecision","Heterogeneity"]
H_Lemborexant_10mg:N_Daridorexant_50mg	0	No concerns	Low risk	No concerns	Major concerns	No concerns	No concerns	Low	["Imprecision"]
J_Lemborexant_25mg:K_Daridorexant_5mg	0	No concerns	Low risk	No concerns	Major concerns	No concerns	No concerns	Low	["Imprecision"]
J_Lemborexant_25mg:L_Daridorexant_10mg	0	No concerns	Low risk	No concerns	Major concerns	No concerns	No concerns	Low	["Imprecision"]
J_Lemborexant_25mg:M_Daridorexant_25mg	0	No concerns	Low risk	No concerns	Major concerns	No concerns	No concerns	Low	["Imprecision"]
J_Lemborexant_25mg:N_Daridorexant_50mg	0	No concerns	Low risk	No concerns	Major concerns	No concerns	No concerns	Low	["Imprecision"]

Abbreviation: LPS: latency to persistent sleep; sTSO: subjective time to sleep onset; WASO: wake after sleep onset; sWASO: subjective wake after sleep onset; TST: total sleep time; sTST: subjective total sleep time; ISI: insomnia severity index score; AE: adverse events; SAE: serious adverse events.

eTable 7: Net plot for each outcome in studies < 1 month follow-up.

LPS	sTSO	WASO
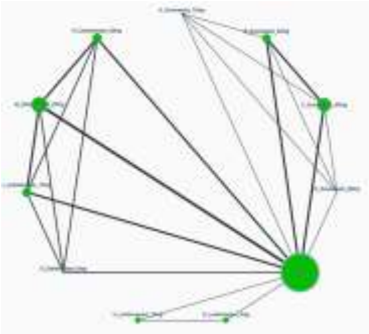	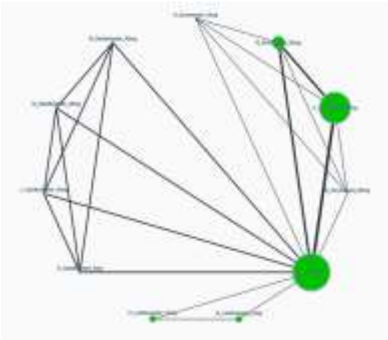	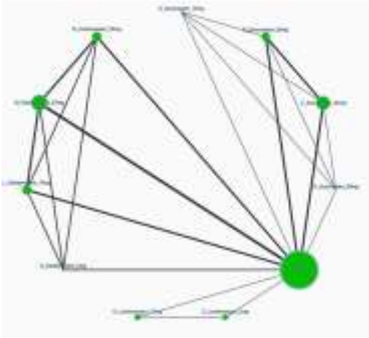
**sWASO**	**TST**	**sTST**
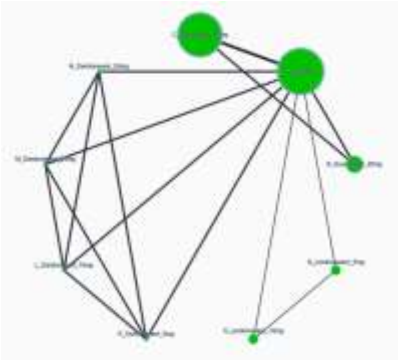	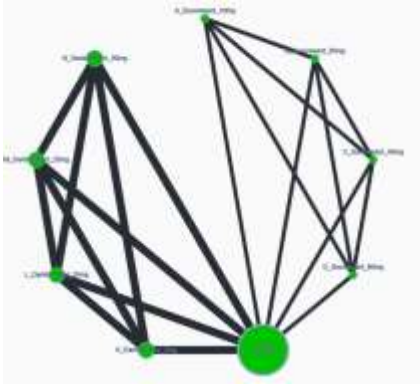	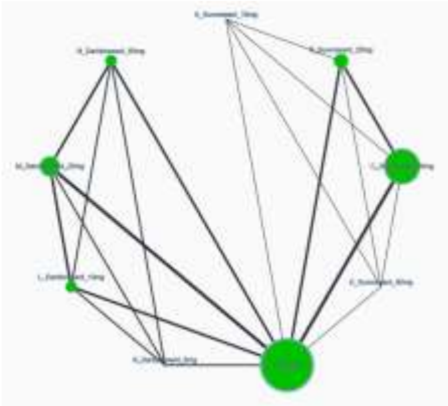
**ISI**	**AE**	**SAE**
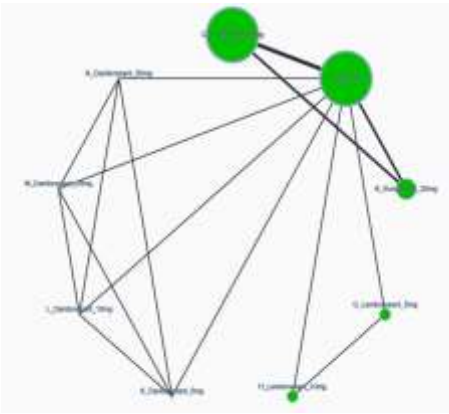	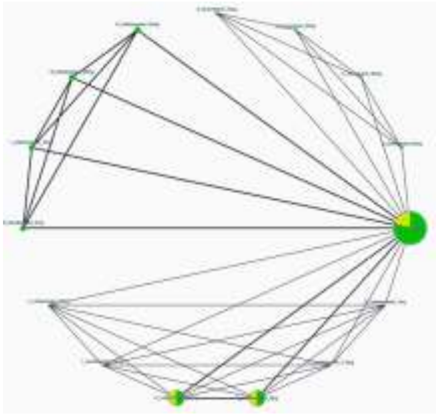	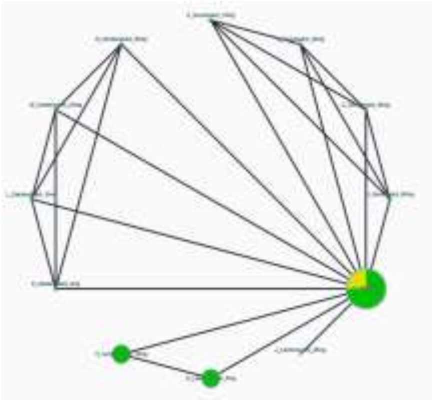

eTable 8: Network meta-analysis results of LPS in studies < 1 month follow-up.

Suvorexant 10mg	9.803 (-2.402, 22.008)	9.416 (-2.703, 21.536)	7.200 (-7.914, 22.314)	10.090 (-4.001, 24.182)	12.090 (-1.972, 26.152)
-9.803 (-22.008, 2.402)	Suvorexant 20mg	-0.386 (-5.748, 4.976)	-2.603 (-15.037, 9.832)	0.288 (-9.103, 9.678)	2.288 (-7.058, 11.633)
-9.416 (-21.536, 2.703)	0.386 (-4.976, 5.748)	Suvorexant 40mg	-2.216 (-14.567, 10.134)	0.674 (-8.526, 9.874)	2.674 (-6.481, 11.828)
-7.200 (-22.314, 7.914)	2.603 (-9.832, 15.037)	2.216 (-10.134, 14.567)	Suvorexant 80mg	2.890 (-11.401, 17.181)	4.890 (-9.372, 19.152)
-10.090 (-24.182, 4.001)	-0.288 (-9.678, 9.103)	-0.674 (-9.874, 8.526)	-2.890 (-17.181, 11.401)	Lemborexant 5mg	2.000 (-5.568, 9.568)
-12.090 (-26.152, 1.972)	-2.288 (-11.633, 7.058)	-2.674 (-11.828, 6.481)	-4.890 (-19.152, 9.372)	-2.000 (-9.568, 5.568)	Lemborexant 10mg
9.364 (-3.255, 21.982)	19.166 (12.178, 26.154)	18.780 (12.050, 25.510)	16.564 (3.723, 29.404)	19.454 (10.340, 28.567)	21.454 (12.386, 30.521)
-6.824 (-19.233, 5.585)	2.979 (-3.624, 9.581)	2.592 (-3.736, 8.921)	0.376 (-12.259, 13.011)	3.266 (-5.555, 12.087)	5.266 (-3.508, 14.040)
-7.068 (-19.341, 5.206)	2.735 (-3.609, 9.079)	2.349 (-3.710, 8.408)	0.132 (-12.370, 12.634)	3.022 (-5.607, 11.652)	5.022 (-3.559, 13.604)
-6.745 (-19.120, 5.631)	3.058 (-3.481, 9.597)	2.672 (-3.591, 8.935)	0.455 (-12.147, 13.057)	3.345 (-5.429, 12.119)	5.345 (-3.381, 14.072)
1.510 (-10.211, 13.230)	11.312 (6.119, 16.506)	10.926 (6.085, 15.768)	8.710 (-3.250, 20.669)	11.600 (3.777, 19.423)	13.600 (5.830, 21.370)

-9.364 (-21.982, 3.255)	6.824 (-5.585, 19.233)	7.068 (-5.206, 19.341)	6.745 (-5.631, 19.120)	-1.510 (-13.230, 10.211)
-19.166 (-26.154, -12.178)	-2.979 (-9.581, 3.624)	-2.735 (-9.079, 3.609)	-3.058 (-9.597, 3.481)	-11.312 (-16.506, -6.119)
-18.780 (-25.510, -12.050)	-2.592 (-8.921, 3.736)	-2.349 (-8.408, 3.710)	-2.672 (-8.935, 3.591)	-10.926 (-15.768, -6.085)
-16.564 (-29.404, -3.723)	-0.376 (-13.011, 12.259)	-0.132 (-12.634, 12.370)	-0.455 (-13.057, 12.147)	-8.710 (-20.669, 3.250)
-19.454 (-28.567, -10.340)	-3.266 (-12.087, 5.555)	-3.022 (-11.652, 5.607)	-3.345 (-12.119, 5.429)	-11.600 (-19.423, -3.777)
-21.454 (-30.521, -12.386)	-5.266 (-14.040, 3.508)	-5.022 (-13.604, 3.559)	-5.345 (-14.072, 3.381)	-13.600 (-21.370, -5.830)
Daridorexant 5mg	16.188 (11.317, 21.058)	16.431 (11.757, 21.106)	16.108 (11.282, 20.935)	7.854 (3.179, 12.528)
-16.188 (-21.058, -11.317)	Daridorexant 10mg	0.244 (-3.834, 4.322)	-0.079 (-4.529, 4.371)	-8.334 (-12.410, -4.258)
-16.431 (-21.106, -11.757)	-0.244 (-4.322, 3.834)	Daridorexant 25mg	-0.323 (-4.296, 3.650)	-8.578 (-12.221, -4.935)

-16.108 (-20.935, -11.282)	0.079 (-4.371, 4.529)	0.323 (-3.650, 4.296)	Daridorexant 50mg	-8.255 (-12.228, -4.282)
-7.854 (-12.528, -3.179)	8.334 (4.258, 12.410)	8.578 (4.935, 12.221)	8.255 (4.282, 12.228)	Placebo

eTable 9: Network meta-analysis results of sTSO in studies < 1 month follow-up.

Suvorexant 10mg	3.755 (-3.696, 11.206)	8.773 (1.504, 16.042)	4.700 (-4.388, 13.788)	15.136 (6.145, 24.128)	14.736 (5.800, 23.673)
-3.755 (-11.206, 3.696)	Suvorexant 20mg	5.018 (1.238, 8.797)	0.945 (-6.804, 8.694)	11.381 (4.681, 18.082)	10.981 (4.355, 17.608)
-8.773 (-16.042, -1.504)	-5.018 (-8.797, -1.238)	Suvorexant 40mg	-4.073 (-11.647, 3.501)	6.363 (0.040, 12.687)	5.963 (-0.282, 12.209)
-4.700 (-13.788, 4.388)	-0.945 (-8.694, 6.804)	4.073 (-3.501, 11.647)	Suvorexant 80mg	10.436 (1.197, 19.676)	10.036 (0.850, 19.222)
-15.136 (-24.128, -6.145)	-11.381 (-18.082, -4.681)	-6.363 (-12.687, -0.040)	-10.436 (-19.676, -1.197)	Lemborexant 5mg	-0.400 (-6.248, 5.448)
-14.736 (-23.673, -5.800)	-10.981 (-17.608, -4.355)	-5.963 (-12.209, 0.282)	-10.036 (-19.222, -0.850)	0.400 (-5.448, 6.248)	Lemborexant 10mg
5.742 (-1.364, 12.848)	9.497 (5.683, 13.311)	14.515 (11.410, 17.620)	10.442 (3.025, 17.860)	20.878 (15.172, 26.585)	20.478 (14.859, 26.098)
-0.677 (-7.790, 6.436)	3.078 (-0.749, 6.905)	8.096 (4.975, 11.216)	4.023 (-3.401, 11.447)	14.459 (8.744, 20.174)	14.059 (8.431, 19.687)
1.511 (-5.598, 8.619)	5.266 (1.447, 9.085)	10.283 (7.172, 13.394)	6.211 (-1.210, 13.631)	16.647 (10.937, 22.356)	16.247 (10.624, 21.869)
-4.074 (-11.183, 3.034)	-0.320 (-4.137, 3.498)	4.698 (1.589, 7.808)	0.626 (-6.794, 8.045)	11.062 (5.353, 16.771)	10.662 (5.040, 16.284)
1.964 (-5.064, 8.992)	5.719 (2.053, 9.385)	10.737 (7.815, 13.658)	6.664 (-0.679, 14.006)	17.100 (11.492, 22.709)	16.700 (11.180, 22.220)

-5.742 (-12.848, 1.364)	0.677 (-6.436, 7.790)	-1.511 (-8.619, 5.598)	4.074 (-3.034, 11.183)	-1.964 (-8.992, 5.064)
-9.497 (-13.311, -5.683)	-3.078 (-6.905, 0.749)	-5.266 (-9.085, -1.447)	0.320 (-3.498, 4.137)	-5.719 (-9.385, -2.053)
-14.515 (-17.620, -11.410)	-8.096 (-11.216, -4.975)	-10.283 (-13.394, -7.172)	-4.698 (-7.808, -1.589)	-10.737 (-13.658, -7.815)
-10.442 (-17.860, -3.025)	-4.023 (-11.447, 3.401)	-6.211 (-13.631, 1.210)	-0.626 (-8.045, 6.794)	-6.664 (-14.006, 0.679)
-20.878 (-26.585, -15.172)	-14.459 (-20.174, -8.744)	-16.647 (-22.356, -10.937)	-11.062 (-16.771, -5.353)	-17.100 (-22.709, -11.492)
-20.478 (-26.098, -14.859)	-14.059 (-19.687, -8.431)	-16.247 (-21.869, -10.624)	-10.662 (-16.284, -5.040)	-16.700 (-22.220, -11.180)
Daridorexant 5mg	6.419 (5.340, 7.498)	4.232 (3.180, 5.283)	9.817 (8.770, 10.864)	3.778 (2.726, 4.831)
-6.419 (-7.498, -5.340)	Daridorexant 10mg	-2.188 (-3.284, -1.091)	3.397 (2.305, 4.490)	-2.641 (-3.738, -1.543)
-4.232 (-5.283, -3.180)	2.188 (1.091, 3.284)	Daridorexant 25mg	5.585 (4.520, 6.650)	-0.453 (-1.523, 0.617)

-9.817 (-10.864, -8.770)	-3.397 (-4.490, -2.305)	-5.585 (-6.650, -4.520)	Daridorexant 50mg	-6.038 (-7.104, -4.972)
-3.778 (-4.831, -2.726)	2.641 (1.543, 3.738)	0.453 (-0.617, 1.523)	6.038 (4.972, 7.104)	Placebo

eTable 10: Network meta-analysis results of WASO in studies < 1 month follow-up.

Suvorexant 10mg	6.111 (-11.749, 23.971)	9.068 (-8.717, 26.852)	7.500 (-14.087, 29.087)	5.360 (-16.476, 27.196)	7.860 (-13.978, 29.698)
-6.111 (-23.971, 11.749)	Suvorexant 20mg	2.956 (-6.084, 11.997)	1.389 (-16.764, 19.542)	-0.751 (-16.838, 15.336)	1.749 (-14.341, 17.839)
-9.068 (-26.852, 8.717)	-2.956 (-11.997, 6.084)	Suvorexant 40mg	-1.567 (-19.646, 16.511)	-3.708 (-19.612, 12.197)	-1.208 (-17.115, 14.699)
-7.500 (-29.087, 14.087)	-1.389 (-19.542, 16.764)	1.567 (-16.511, 19.646)	Suvorexant 80mg	-2.140 (-24.216, 19.936)	0.360 (-21.718, 22.438)
-5.360 (-27.196, 16.476)	0.751 (-15.336, 16.838)	3.708 (-12.197, 19.612)	2.140 (-19.936, 24.216)	Lemborexant 5mg	2.500 (-10.644, 15.644)
-7.860 (-29.698, 13.978)	-1.749 (-17.839, 14.341)	1.208 (-14.699, 17.115)	-0.360 (-22.438, 21.718)	-2.500 (-15.644, 10.644)	Lemborexant 10mg
16.637 (-2.736, 36.011)	22.749 (10.205, 35.292)	25.705 (13.397, 38.013)	24.137 (4.493, 43.782)	21.997 (5.745, 38.249)	24.497 (8.242, 40.752)
11.833 (-6.855, 30.522)	17.945 (6.488, 29.401)	20.901 (9.702, 32.099)	19.333 (0.365, 38.302)	17.193 (1.765, 32.622)	19.693 (4.262, 35.125)
8.455 (-9.940, 26.850)	14.566 (3.595, 25.537)	17.523 (6.822, 28.224)	15.955 (-2.724, 34.634)	13.815 (-1.256, 28.886)	16.315 (1.241, 31.389)
-1.901 (-20.579, 16.777)	4.210 (-7.230, 15.650)	7.166 (-4.015, 18.347)	5.599 (-13.360, 24.557)	3.459 (-11.957, 18.875)	5.959 (-9.460, 21.378)
19.940 (2.793, 37.087)	26.051 (17.333, 34.769)	29.008 (20.632, 37.383)	27.440 (9.989, 44.892)	25.300 (11.780, 38.820)	27.800 (14.277, 41.323)

-16.637 (-36.011, 2.736)	-11.833 (-30.522, 6.855)	-8.455 (-26.850, 9.940)	1.901 (-16.777, 20.579)	-19.940 (-37.087, -2.793)
-22.749 (-35.292, -10.205)	-17.945 (-29.401, -6.488)	-14.566 (-25.537, -3.595)	-4.210 (-15.650, 7.230)	-26.051 (-34.769, -17.333)
-25.705 (-38.013, -13.397)	-20.901 (-32.099, -9.702)	-17.523 (-28.224, -6.822)	-7.166 (-18.347, 4.015)	-29.008 (-37.383, -20.632)
-24.137 (-43.782, -4.493)	-19.333 (-38.302, -0.365)	-15.955 (-34.634, 2.724)	-5.599 (-24.557, 13.360)	-27.440 (-44.892, -9.989)
-21.997 (-38.249, -5.745)	-17.193 (-32.622, -1.765)	-13.815 (-28.886, 1.256)	-3.459 (-18.875, 11.957)	-25.300 (-38.820, -11.780)
-24.497 (-40.752, -8.242)	-19.693 (-35.125, -4.262)	-16.315 (-31.389, -1.241)	-5.959 (-21.378, 9.460)	-27.800 (-41.323, -14.277)
Daridorexant 5mg	4.804 (-4.502, 14.110)	8.182 (-0.828, 17.192)	18.539 (9.225, 27.853)	-3.303 (-12.322, 5.716)
-4.804 (-14.110, 4.502)	Daridorexant 10mg	3.378 (-4.048, 10.805)	13.735 (5.479, 21.991)	-8.107 (-15.540, -0.674)
-8.182 (-17.192, 0.828)	-3.378 (-10.805, 4.048)	Daridorexant 25mg	10.356 (2.958, 17.755)	-11.485 (-18.145, -4.825)

-18.539 (-27.853, -9.225)	-13.735 (-21.991, -5.479)	-10.356 (-17.755, -2.958)	Daridorexant 50mg	-21.841 (-29.248, -14.434)
3.303 (-5.716, 12.322)	8.107 (0.674, 15.540)	11.485 (4.825, 18.145)	21.841 (14.434, 29.248)	Placebo

eTable 11: Network meta-analysis results of sWASO in studies < 1 month follow-up.

Suvorexant 20mg	2.296 (-1.660, 6.253)	1.714 (-9.502, 12.930)	15.214 (2.855, 27.573)	-3.073 (-7.319, 1.173)	-8.627 (-12.891, -4.363)	0.983 (-3.257, 5.223)	4.864 (0.614, 9.115)	-6.786 (-10.758, -2.814)
-2.296 (-6.253, 1.660)	Suvorexant 40mg	-0.583 (-11.468, 10.302)	12.917 (0.858, 24.976)	-5.370 (-8.642, -2.097)	-10.924 (-14.219, -7.628)	-1.314 (-4.578, 1.951)	2.568 (-0.710, 5.846)	-9.083 (-11.991, -6.174)
-1.714 (-12.930, 9.502)	0.583 (-10.302, 11.468)	Lemborexant 5mg	13.500 (2.345, 24.655)	-4.787 (-15.383, 5.809)	-10.341 (-20.944, 0.262)	-0.731 (-11.325, 9.862)	3.151 (-7.447, 13.748)	-8.500 (-18.989, 1.989)
-15.214 (-27.573, -2.855)	-12.917 (-24.976, -0.858)	-13.500 (-24.655, -2.345)	Lemborexant 10mg	-18.287 (-30.086, -6.488)	-23.841 (-35.646, -12.036)	-14.231 (-26.028, -2.435)	-10.350 (-22.150, 1.451)	-22.000 (-33.703, -10.297)
3.073 (-1.173, 7.319)	5.370 (2.097, 8.642)	4.787 (-5.809, 15.383)	18.287 (6.488, 30.086)	Daridorexant 5mg	-5.554 (-7.104, -4.004)	4.056 (2.573, 5.538)	7.937 (6.426, 9.449)	-3.713 (-5.213, -2.213)
8.627 (4.363, 12.891)	10.924 (7.628, 14.219)	10.341 (-0.262, 20.944)	23.841 (12.036, 35.646)	5.554 (4.004, 7.104)	Daridorexant 10mg	9.610 (8.077, 11.142)	13.491 (11.931, 15.052)	1.841 (0.291, 3.391)
-0.983 (-5.223, 3.257)	1.314 (-1.951, 4.578)	0.731 (-9.862, 11.325)	14.231 (2.435, 26.028)	-4.056 (-5.538, -2.573)	-9.610 (-11.142, -8.077)	Daridorexant 25mg	3.882 (2.388, 5.376)	-7.769 (-9.251, -6.286)
-4.864 (-9.115, -0.614)	-2.568 (-5.846, 0.710)	-3.151 (-13.748, 7.447)	10.350 (-1.451, 22.150)	-7.937 (-9.449, -6.426)	-13.491 (-15.052, -11.931)	-3.882 (-5.376, -2.388)	Daridorexant 50mg	-11.650 (-13.162, -10.139)
6.786 (2.814, 10.758)	9.083 (6.174, 11.991)	8.500 (-1.989, 18.989)	22.000 (10.297, 33.703)	3.713 (2.213, 5.213)	-1.841 (-3.391, -0.291)	7.769 (6.286, 9.251)	11.650 (10.139, 13.162)	Placebo

eTable 12: Network meta-analysis results of TST in studies < 1 month follow-up.

Suvorexant 10mg	-27.600 (-61.542, 6.342)	-14.500 (-48.442, 19.442)	-14.300 (-48.378, 19.778)	22.051 (-15.418, 59.521)	-0.007 (-37.375, 37.361)	-4.125 (-41.465, 33.215)	-14.828 (-52.226, 22.571)	22.300 (-8.988, 53.588)
27.600 (-6.342, 61.542)	Suvorexant 20mg	13.100 (-20.974, 47.174)	13.300 (-20.909, 47.509)	49.651 (12.063, 87.240)	27.593 (-9.894, 65.081)	23.475 (-13.984, 60.934)	12.772 (-24.746, 50.291)	49.900 (18.469, 81.331)
14.500 (-19.442, 48.442)	-13.100 (-47.174, 20.974)	Suvorexant 40mg	0.200 (-34.009, 34.409)	36.551 (-1.038, 74.140)	14.493 (-22.994, 51.981)	10.375 (-27.085, 47.834)	-0.328 (-37.846, 37.191)	36.800 (5.369, 68.231)
14.300 (-19.778, 48.378)	-13.300 (-47.509, 20.909)	-0.200 (-34.409, 34.009)	Suvorexant 80mg	36.351 (-1.360, 74.063)	14.293 (-23.317, 51.904)	10.175 (-27.408, 47.758)	-0.528 (-38.169, 37.114)	36.600 (5.022, 68.178)
-22.051 (-59.521, 15.418)	-49.651 (-87.240, -12.063)	-36.551 (-74.140, 1.038)	-36.351 (-74.063, 1.360)	Daridorexant 5mg	-22.058 (-42.726, -1.390)	-26.177 (-46.794, -5.559)	-36.879 (-57.600, -16.158)	0.249 (-20.367, 20.864)
0.007 (-37.361, 37.375)	-27.593 (-65.081, 9.894)	-14.493 (-51.981, 22.994)	-14.293 (-51.904, 23.317)	22.058 (1.390, 42.726)	Daridorexant 10mg	-4.119 (-24.551, 16.314)	-14.821 (-35.361, 5.719)	22.307 (1.875, 42.738)
4.125 (-33.215, 41.465)	-23.475 (-60.934, 13.984)	-10.375 (-47.834, 27.085)	-10.175 (-47.758, 27.408)	26.177 (5.559, 46.794)	4.119 (-16.314, 24.551)	Daridorexant 25mg	-10.703 (-31.191, 9.786)	26.425 (6.046, 46.804)
14.828 (-22.571, 52.226)	-12.772 (-50.291, 24.746)	0.328 (-37.191, 37.846)	0.528 (-37.114, 38.169)	36.879 (16.158, 57.600)	14.821 (-5.719, 35.361)	10.703 (-9.786, 31.191)	Daridorexant 50mg	37.128 (16.641, 57.614)
-22.300 (-53.588, 8.988)	-49.900 (-81.331, -18.469)	-36.800 (-68.231, -5.369)	-36.600 (-68.178, -5.022)	-0.249 (-20.864, 20.367)	-22.307 (-42.738, -1.875)	-26.425 (-46.804, -6.046)	-37.128 (-57.614, -16.641)	Placebo

eTable 13: Network meta-analysis results of sTST in studies < 1 month follow-up.

Suvorexant 10mg	-7.956 (-21.508, 5.596)	-15.594 (-28.830, -2.357)	-13.900 (-30.941, 3.141)	12.569 (-5.551, 30.688)	-4.069 (-18.550, 10.413)	-6.493 (-20.392, 7.406)	-16.925 (-31.365, -2.485)	7.400 (-5.497, 20.298)
7.956 (-5.596, 21.508)	Suvorexant 20mg	-7.638 (-13.271, -2.005)	-5.944 (-19.932, 8.044)	20.525 (6.655, 34.395)	3.887 (-4.702, 12.477)	1.463 (-6.103, 9.029)	-8.969 (-17.489, -0.449)	15.356 (9.841, 20.871)
15.594 (2.357, 28.830)	7.638 (2.005, 13.271)	Suvorexant 40mg	1.694 (-11.988, 15.376)	28.163 (14.779, 41.546)	11.525 (3.745, 19.305)	9.101 (2.468, 15.734)	-1.331 (-9.034, 6.372)	22.994 (18.851, 27.137)
13.900 (-3.141, 30.941)	5.944 (-8.044, 19.932)	-1.694 (-15.376, 11.988)	Suvorexant 80mg	26.469 (8.021, 44.916)	9.831 (-5.059, 24.721)	7.407 (-6.917, 21.731)	-3.025 (-17.875, 11.825)	21.300 (7.946, 34.655)
-12.569 (-30.688, 5.551)	-20.525 (-34.395, -6.655)	-28.163 (-41.546, -14.779)	-26.469 (-44.916, -8.021)	Daridorexant 5mg	-16.637 (-29.788, -3.487)	-19.061 (-31.749, -6.374)	-29.494 (-42.672, -16.316)	-5.169 (-17.895, 7.558)
4.069 (-10.413, 18.550)	-3.887 (-12.477, 4.702)	-11.525 (-19.305, -3.745)	-9.831 (-24.721, 5.059)	16.637 (3.487, 29.788)	Daridorexant 10mg	-2.424 (-8.989, 4.141)	-12.856 (-21.054, -4.658)	11.469 (4.884, 18.054)
6.493 (-7.406, 20.392)	-1.463 (-9.029, 6.103)	-9.101 (-15.734, -2.468)	-7.407 (-21.731, 6.917)	19.061 (6.374, 31.749)	2.424 (-4.141, 8.989)	Daridorexant 25mg	-10.432 (-16.907, -3.958)	13.893 (8.713, 19.073)
16.925 (2.485, 31.365)	8.969 (0.449, 17.489)	1.331 (-6.372, 9.034)	3.025 (-11.825, 17.875)	29.494 (16.316, 42.672)	12.856 (4.658, 21.054)	10.432 (3.958, 16.907)	Daridorexant 50mg	24.325 (17.831, 30.819)
-7.400 (-20.298, 5.497)	-15.356 (-20.871, -9.841)	-22.994 (-27.137, -18.851)	-21.300 (-34.655, -7.946)	5.169 (-7.558, 17.895)	-11.469 (-18.054, -4.884)	-13.893 (-19.073, -8.713)	-24.325 (-30.819, -17.831)	Placebo

eTable 14: Network meta-analysis results of AEs in studies < 1 month follow-up.

Suvorexant 10mg	0.902 (0.431, 1.886)	0.582 (0.301, 1.125)	0.492 (0.262, 0.925)	1.048 (0.472, 2.328)	0.884 (0.404, 1.937)
1.109 (0.530, 2.319)	Suvorexant 20mg	0.645 (0.341, 1.219)	0.545 (0.297, 1.001)	1.162 (0.533, 2.532)	0.980 (0.456, 2.106)
1.720 (0.889, 3.327)	1.551 (0.820, 2.932)	Suvorexant 40mg	0.846 (0.508, 1.408)	1.802 (0.889, 3.650)	1.520 (0.763, 3.031)
2.033 (1.081, 3.823)	1.833 (0.999, 3.365)	1.182 (0.710, 1.968)	Suvorexant 80mg	2.130 (1.080, 4.202)	1.797 (0.926, 3.487)
0.954 (0.430, 2.120)	0.861 (0.395, 1.876)	0.555 (0.274, 1.124)	0.469 (0.238, 0.926)	Lemborexant 1mg	0.844 (0.436, 1.633)
1.131 (0.516, 2.478)	1.020 (0.475, 2.192)	0.658 (0.330, 1.311)	0.556 (0.287, 1.080)	1.185 (0.612, 2.294)	Lemborexant 2.5mg
1.265 (0.664, 2.409)	1.141 (0.613, 2.122)	0.735 (0.435, 1.244)	0.622 (0.381, 1.015)	1.325 (0.774, 2.269)	1.118 (0.667, 1.874)
1.482 (0.782, 2.808)	1.336 (0.722, 2.473)	0.862 (0.513, 1.449)	0.729 (0.450, 1.181)	1.553 (0.917, 2.630)	1.310 (0.790, 2.172)
1.537 (0.778, 3.036)	1.386 (0.718, 2.677)	0.894 (0.506, 1.580)	0.756 (0.442, 1.293)	1.610 (0.945, 2.745)	1.359 (0.814, 2.268)
1.666 (0.846, 3.281)	1.502 (0.780, 2.892)	0.969 (0.550, 1.706)	0.819 (0.481, 1.396)	1.745 (1.028, 2.964)	1.473 (0.886, 2.448)
1.436 (0.690, 2.988)	1.295 (0.636, 2.639)	0.835 (0.445, 1.569)	0.707 (0.387, 1.289)	1.505 (0.755, 3.001)	1.270 (0.647, 2.492)
1.495 (0.720, 3.104)	1.348 (0.663, 2.741)	0.869 (0.464, 1.630)	0.735 (0.404, 1.338)	1.567 (0.787, 3.117)	1.322 (0.675, 2.588)
1.439 (0.692, 2.991)	1.297 (0.637, 2.642)	0.837 (0.446, 1.571)	0.708 (0.388, 1.290)	1.507 (0.756, 3.004)	1.272 (0.649, 2.494)
1.557 (0.751, 3.224)	1.404 (0.692, 2.847)	0.905 (0.484, 1.692)	0.766 (0.422, 1.389)	1.631 (0.822, 3.238)	1.376 (0.705, 2.687)
1.132 (0.627, 2.043)	1.021 (0.580, 1.795)	0.658 (0.416, 1.040)	0.557 (0.367, 0.844)	1.186 (0.693, 2.030)	1.001 (0.597, 1.676)

0.791 (0.415, 1.507)	0.675 (0.356, 1.279)	0.651 (0.329, 1.285)	0.600 (0.305, 1.182)	0.696 (0.335, 1.448)	0.669 (0.322, 1.389)
0.877 (0.471, 1.631)	0.748 (0.404, 1.384)	0.722 (0.374, 1.393)	0.666 (0.346, 1.282)	0.772 (0.379, 1.573)	0.742 (0.365, 1.508)
1.360 (0.804, 2.301)	1.160 (0.690, 1.951)	1.119 (0.633, 1.978)	1.032 (0.586, 1.818)	1.197 (0.637, 2.249)	1.150 (0.614, 2.156)
1.608 (0.985, 2.623)	1.372 (0.846, 2.223)	1.323 (0.774, 2.262)	1.220 (0.716, 2.079)	1.415 (0.776, 2.581)	1.360 (0.747, 2.474)
0.755 (0.441, 1.292)	0.644 (0.380, 1.091)	0.621 (0.364, 1.059)	0.573 (0.337, 0.973)	0.664 (0.333, 1.325)	0.638 (0.321, 1.270)

0.894 (0.533, 1.499)	0.763 (0.460, 1.265)	0.736 (0.441, 1.228)	0.679 (0.409, 1.129)	0.787 (0.401, 1.545)	0.757 (0.386, 1.481)
Lemborexant 5mg	0.853 (0.678, 1.074)	0.823 (0.586, 1.156)	0.759 (0.544, 1.060)	0.880 (0.532, 1.458)	0.846 (0.512, 1.397)
1.172 (0.931, 1.475)	Lemborexant 10mg	0.964 (0.699, 1.331)	0.890 (0.649, 1.220)	1.032 (0.627, 1.698)	0.991 (0.604, 1.626)
1.215 (0.865, 1.707)	1.037 (0.751, 1.431)	Lemborexant 15mg	0.923 (0.666, 1.279)	1.070 (0.617, 1.855)	1.028 (0.595, 1.777)
1.317 (0.944, 1.839)	1.124 (0.820, 1.542)	1.084 (0.782, 1.502)	Lemborexant 25mg	1.160 (0.672, 2.003)	1.114 (0.647, 1.919)
1.136 (0.686, 1.881)	0.969 (0.589, 1.595)	0.935 (0.539, 1.620)	0.862 (0.499, 1.489)	Daridorexant 5mg	0.961 (0.649, 1.422)
1.182 (0.716, 1.953)	1.009 (0.615, 1.655)	0.973 (0.563, 1.682)	0.897 (0.521, 1.546)	1.041 (0.703, 1.540)	Daridorexant 10mg
1.138 (0.687, 1.883)	0.971 (0.591, 1.596)	0.936 (0.540, 1.621)	0.864 (0.500, 1.490)	1.002 (0.674, 1.487)	0.962 (0.651, 1.423)
1.231 (0.748, 2.026)	1.050 (0.642, 1.717)	1.013 (0.588, 1.746)	0.934 (0.544, 1.604)	1.084 (0.737, 1.594)	1.041 (0.710, 1.528)
0.895 (0.691, 1.159)	0.764 (0.598, 0.976)	0.736 (0.525, 1.033)	0.679 (0.487, 0.948)	0.788 (0.511, 1.216)	0.757 (0.492, 1.164)

0.695 (0.334, 1.445)	0.642 (0.310, 1.331)	0.884 (0.490, 1.595)
0.771 (0.379, 1.569)	0.712 (0.351, 1.445)	0.980 (0.557, 1.723)
1.195 (0.637, 2.244)	1.105 (0.591, 2.065)	1.519 (0.961, 2.402)
1.413 (0.775, 2.575)	1.306 (0.720, 2.369)	1.796 (1.185, 2.723)
0.663 (0.333, 1.322)	0.613 (0.309, 1.217)	0.843 (0.493, 1.443)
0.786 (0.401, 1.542)	0.727 (0.372, 1.419)	0.999 (0.596, 1.674)
0.879 (0.531, 1.455)	0.812 (0.494, 1.337)	1.117 (0.863, 1.447)
1.030 (0.627, 1.693)	0.952 (0.582, 1.556)	1.309 (1.025, 1.673)
1.068 (0.617, 1.850)	0.987 (0.573, 1.702)	1.358 (0.968, 1.905)
1.158 (0.671, 1.998)	1.070 (0.623, 1.837)	1.472 (1.055, 2.052)
0.998 (0.672, 1.483)	0.923 (0.627, 1.357)	1.269 (0.823, 1.958)

1.039 (0.703, 1.537)	0.960 (0.655, 1.409)	1.321 (0.859, 2.031)
Daridorexant 25mg	0.924 (0.627, 1.362)	1.271 (0.825, 1.959)
1.082 (0.734, 1.594)	Daridorexant 50mg	1.375 (0.898, 2.106)
0.787 (0.511, 1.212)	0.727 (0.475, 1.113)	Placebo

eTable 15: Network meta-analysis results of SAEs in studies < 1 month follow-up.

Suvorexant 10mg	0.984 (0.020, 48.813)	0.952 (0.019, 47.210)	0.984 (0.020, 48.813)	1.016 (0.007, 142.994)	5.116 (0.020, 1294.785)
1.016 (0.020, 50.411)	Suvorexant 20mg	0.967 (0.020, 47.971)	1.000 (0.020, 49.600)	1.032 (0.007, 145.300)	5.199 (0.021, 1315.800)
1.050 (0.021, 52.091)	1.034 (0.021, 51.254)	Suvorexant 40mg	1.034 (0.021, 51.254)	1.067 (0.008, 150.160)	5.374 (0.021, 1359.810)
1.016 (0.020, 50.411)	1.000 (0.020, 49.600)	0.967 (0.020, 47.971)	Suvorexant 80mg	1.032 (0.007, 145.300)	5.199 (0.021, 1315.800)
0.985 (0.007, 138.629)	0.969 (0.007, 136.401)	0.937 (0.007, 131.934)	0.969 (0.007, 136.401)	Lemborexant 5mg	5.037 (0.243, 104.428)
0.195 (0.001, 49.467)	0.192 (0.001, 48.672)	0.186 (0.001, 47.078)	0.192 (0.001, 48.672)	0.199 (0.010, 4.115)	Lemborexant 10mg
0.281 (0.002, 33.338)	0.276 (0.002, 32.802)	0.267 (0.002, 31.728)	0.276 (0.002, 32.802)	0.285 (0.005, 17.015)	1.435 (0.012, 171.366)
0.250 (0.001, 62.859)	0.246 (0.001, 61.849)	0.238 (0.001, 59.824)	0.246 (0.001, 61.849)	0.254 (0.002, 35.638)	1.282 (0.005, 322.886)
1.295 (0.009, 180.603)	1.275 (0.009, 177.701)	1.233 (0.009, 171.881)	1.275 (0.009, 177.701)	1.316 (0.018, 94.576)	6.627 (0.047, 928.156)
0.250 (0.001, 62.859)	0.246 (0.001, 61.849)	0.238 (0.001, 59.824)	0.246 (0.001, 61.849)	0.254 (0.002, 35.638)	1.282 (0.005, 322.886)
0.739 (0.005, 114.263)	0.727 (0.005, 112.415)	0.704 (0.005, 108.744)	0.727 (0.005, 112.415)	0.751 (0.009, 60.770)	3.782 (0.024, 587.162)
0.250 (0.005, 12.501)	0.246 (0.005, 12.300)	0.238 (0.005, 11.896)	0.246 (0.005, 12.300)	0.254 (0.012, 5.271)	1.282 (0.026, 64.322)

3.564 (0.030, 423.562)	3.992 (0.016, 1001.746)	0.772 (0.006, 107.641)	3.992 (0.016, 1001.746)	1.353 (0.009, 209.056)	3.992 (0.080, 199.218)
3.622 (0.030, 430.394)	4.057 (0.016, 1017.903)	0.785 (0.006, 109.377)	4.057 (0.016, 1017.903)	1.375 (0.009, 212.427)	4.057 (0.081, 202.431)
3.744 (0.032, 444.745)	4.193 (0.017, 1051.949)	0.811 (0.006, 113.035)	4.193 (0.017, 1051.949)	1.421 (0.009, 219.532)	4.193 (0.084, 209.181)
3.622 (0.030, 430.394)	4.057 (0.016, 1017.903)	0.785 (0.006, 109.377)	4.057 (0.016, 1017.903)	1.375 (0.009, 212.427)	4.057 (0.081, 202.431)
3.510 (0.059, 209.558)	3.931 (0.028, 550.595)	0.760 (0.011, 54.647)	3.931 (0.028, 550.595)	1.332 (0.016, 107.802)	3.931 (0.190, 81.435)
0.697 (0.006, 83.171)	0.780 (0.003, 196.586)	0.151 (0.001, 21.132)	0.780 (0.003, 196.586)	0.264 (0.002, 41.042)	0.780 (0.016, 39.158)
Lemborexant 25mg	1.120 (0.009, 132.396)	0.217 (0.004, 12.782)	1.120 (0.009, 132.396)	0.380 (0.006, 25.353)	1.120 (0.072, 17.441)
0.893 (0.008, 105.541)	Daridorexant 5mg	0.193 (0.009, 3.943)	1.000 (0.020, 49.586)	0.339 (0.014, 8.156)	1.000 (0.020, 49.586)

4.617 (0.078, 272.435)	5.171 (0.254, 105.436)	Daridorexant 10mg	5.171 (0.254, 105.436)	1.752 (0.239, 12.848)	5.171 (0.254, 105.436)
0.893 (0.008, 105.541)	1.000 (0.020, 49.586)	0.193 (0.009, 3.943)	Daridorexant 25mg	0.339 (0.014, 8.156)	1.000 (0.020, 49.586)
2.635 (0.039, 176.038)	2.951 (0.123, 71.030)	0.571 (0.078, 4.185)	2.951 (0.123, 71.030)	Daridorexant 50mg	2.951 (0.123, 71.030)
0.893 (0.057, 13.903)	1.000 (0.020, 49.586)	0.193 (0.009, 3.943)	1.000 (0.020, 49.586)	0.339 (0.014, 8.156)	Placebo

eTable 16: Detailed certainty of evidence for each outcome in studies < 1 month follow-up.

Comparison	Number of studies	Within-study bias	Reporting bias	Indirectness	Imprecision	Heterogeneity	Incoherence	Confidence rating	Reason(s) for downgrading
**1. LPS**									
A_Suvorexant_10mg:O_Placebo	1	No concerns	Low risk	No concerns	Major concerns	No concerns	No concerns	Low	["Imprecision"]
B_Suvorexant_20mg:O_Placebo	3	No concerns	Low risk	No concerns	No concerns	No concerns	No concerns	High	[]
C_Suvorexant_40mg:O_Placebo	3	No concerns	Low risk	No concerns	No concerns	No concerns	Some concerns	Moderate	["Incoherence"]
D_Suvorexant_80mg:O_Placebo	1	No concerns	Low risk	No concerns	Some concerns	No concerns	No concerns	Moderate	["Imprecision"]
G_Lemborexant_5mg:O_Placebo	1	No concerns	Low risk	No concerns	No concerns	No concerns	Some concerns	Moderate	["Incoherence"]
H_Lemborexant_10mg:O_Placebo	1	No concerns	Low risk	No concerns	No concerns	No concerns	Some concerns	Moderate	["Incoherence"]
K_Daridorexant_5mg:O_Placebo	2	No concerns	Low risk	No concerns	No concerns	No concerns	No concerns	High	[]
L_Daridorexant_10mg:O_Placebo	3	No concerns	Low risk	No concerns	No concerns	No concerns	No concerns	High	[]
M_Daridorexant_25mg:O_Placebo	4	No concerns	Low risk	No concerns	No concerns	No concerns	Some concerns	Moderate	["Incoherence"]
N_Daridorexant_50mg:O_Placebo	3	No concerns	Low risk	No concerns	No concerns	No concerns	No concerns	High	[]
**2. sTSO**									
A_Suvorexant_10mg:O_Placebo	1	No concerns	Low risk	No concerns	Major concerns	No concerns	No concerns	Low	["Imprecision"]
B_Suvorexant_20mg:O_Placebo	3	No concerns	Low risk	No concerns	No concerns	No concerns	No concerns	High	[]
C_Suvorexant_40mg:O_Placebo	4	No concerns	Low risk	No concerns	No concerns	No concerns	No concerns	High	[]
D_Suvorexant_80mg:O_Placebo	1	No concerns	Low risk	No concerns	Some concerns	No concerns	No concerns	Moderate	["Imprecision"]
G_Lemborexant_5mg:O_Placebo	1	No concerns	Low risk	No concerns	No concerns	No concerns	No concerns	High	[]
H_Lemborexant_10mg:O_Placebo	1	No concerns	Low risk	No concerns	No concerns	No concerns	No concerns	High	[]
K_Daridorexant_5mg:O_Placebo	2	No concerns	Low risk	No concerns	No concerns	Some concerns	No concerns	Moderate	["Heterogeneity"]
L_Daridorexant_10mg:O_Placebo	2	No concerns	Low risk	No concerns	No concerns	No concerns	No concerns	High	[]
M_Daridorexant_25mg:O_Placebo	2	No concerns	Low risk	No concerns	No concerns	No concerns	No concerns	High	[]
N_Daridorexant_50mg:O_Placebo	2	No concerns	Low risk	No concerns	No concerns	No concerns	No concerns	High	[]
**3. WASO**									
A_Suvorexant_10mg:O_Placebo	1	No concerns	Low risk	No concerns	No concerns	Some concerns	No concerns	Moderate	["Heterogeneity"]
B_Suvorexant_20mg:O_Placebo	3	No concerns	Low risk	No concerns	No concerns	No concerns	No concerns	High	[]

C_Suvorexant_40mg:O_Placebo	3	No concerns	Low risk	No concerns	No concerns	No concerns	No concerns	High	[]
D_Suvorexant_80mg:O_Placebo	1	No concerns	Low risk	No concerns	No concerns	No concerns	No concerns	High	[]
G_Lemborexant_5mg:O_Placebo	1	No concerns	Low risk	No concerns	No concerns	No concerns	No concerns	High	[]
H_Lemborexant_10mg:O_Placebo	1	No concerns	Low risk	No concerns	No concerns	No concerns	No concerns	High	[]
K_Daridorexant_5mg:O_Placebo	2	No concerns	Low risk	No concerns	Some concerns	Some concerns	No concerns	Low	["Imprecision","Heterogeneity"]
L_Daridorexant_10mg:O_Placebo	3	No concerns	Low risk	No concerns	No concerns	Some concerns	No concerns	Moderate	["Heterogeneity"]
M_Daridorexant_25mg:O_Placebo	4	No concerns	Low risk	No concerns	No concerns	Some concerns	No concerns	Moderate	["Heterogeneity"]
N_Daridorexant_50mg:O_Placebo	3	No concerns	Low risk	No concerns	No concerns	No concerns	No concerns	High	[]
**4. sWASO**									
B_Suvorexant_20mg:O_Placebo	2	No concerns	Low risk	No concerns	No concerns	No concerns	No concerns	High	[]
C_Suvorexant_40mg:O_Placebo	3	No concerns	Low risk	No concerns	No concerns	No concerns	No concerns	High	[]
G_Lemborexant_5mg:O_Placebo	1	No concerns	Low risk	No concerns	Some concerns	No concerns	No concerns	Moderate	["Imprecision"]
H_Lemborexant_10mg:O_Placebo	1	No concerns	Low risk	No concerns	No concerns	No concerns	No concerns	High	[]
K_Daridorexant_5mg:O_Placebo	2	No concerns	Low risk	No concerns	No concerns	No concerns	No concerns	High	[]
L_Daridorexant_10mg:O_Placebo	2	No concerns	Low risk	No concerns	No concerns	No concerns	No concerns	High	[]
M_Daridorexant_25mg:O_Placebo	2	No concerns	Low risk	No concerns	No concerns	No concerns	No concerns	High	[]
N_Daridorexant_50mg:O_Placebo	2	No concerns	Low risk	No concerns	No concerns	No concerns	No concerns	High	[]
**5. TST**									
A_Suvorexant_10mg:O_Placebo	1	No concerns	Low risk	No concerns	Some concerns	Some concerns	Major concerns	Very low	["Imprecision","Heterogeneity","Incoherence"]
B_Suvorexant_20mg:O_Placebo	1	No concerns	Low risk	No concerns	No concerns	Some concerns	Major concerns	Very low	["Heterogeneity","Incoherence"]
C_Suvorexant_40mg:O_Placebo	1	No concerns	Low risk	No concerns	No concerns	Major concerns	Major concerns	Very low	["Heterogeneity","Incoherence"]
D_Suvorexant_80mg:O_Placebo	1	No concerns	Low risk	No concerns	No concerns	Major concerns	Major concerns	Very low	["Heterogeneity","Incoherence"]
K_Daridorexant_5mg:O_Placebo	2	No concerns	Low risk	No concerns	Major concerns	No concerns	Major concerns	Very low	["Imprecision","Incoherence"]
L_Daridorexant_10mg:O_Placebo	2	No concerns	Low risk	No concerns	No concerns	Major concerns	Major concerns	Very low	["Heterogeneity","Incoherence"]
M_Daridorexant_25mg:O_Placebo	2	No concerns	Low risk	No concerns	No concerns	Major concerns	Major concerns	Very low	["Heterogeneity","Incoherence"]
N_Daridorexant_50mg:O_Placebo	2	No concerns	Low risk	No concerns	No concerns	Some concerns	Major concerns	Very low	["Heterogeneity","Incoherence"]
**6. sTST**									
A_Suvorexant_10mg:O_Placebo	1	No concerns	Low risk	No concerns	Some concerns	No concerns	No concerns	Moderate	["Imprecision"]
B_Suvorexant_20mg:O_Placebo	3	No concerns	Low risk	No concerns	No concerns	No concerns	No concerns	High	[]

C_Suvorexant_40mg:O_Placebo	4	No concerns	Low risk	No concerns	No concerns	No concerns	Major concerns	Low	["Incoherence"]
D_Suvorexant_80mg:O_Placebo	1	No concerns	Low risk	No concerns	No concerns	No concerns	No concerns	High	[]
K_Daridorexant_5mg:O_Placebo	2	No concerns	Low risk	No concerns	No concerns	Some concerns	No concerns	Moderate	["Heterogeneity"]
L_Daridorexant_10mg:O_Placebo	3	No concerns	Low risk	No concerns	No concerns	Some concerns	No concerns	Moderate	["Heterogeneity"]
M_Daridorexant_25mg:O_Placebo	4	No concerns	Low risk	No concerns	No concerns	Some concerns	No concerns	Moderate	["Heterogeneity"]
N_Daridorexant_50mg:O_Placebo	3	No concerns	Low risk	No concerns	No concerns	No concerns	No concerns	High	[]
**7. ISI**									
B_Suvorexant_20mg:O_Placebo	2	No concerns	Low risk	No concerns	No concerns	Some concerns	No concerns	Moderate	["Heterogeneity"]
C_Suvorexant_40mg:O_Placebo	3	No concerns	Low risk	No concerns	No concerns	Some concerns	No concerns	Moderate	["Heterogeneity"]
G_Lemborexant_5mg:O_Placebo	1	No concerns	Low risk	No concerns	No concerns	Some concerns	No concerns	Moderate	["Heterogeneity"]
H_Lemborexant_10mg:O_Placebo	1	No concerns	Low risk	No concerns	No concerns	Some concerns	No concerns	Moderate	["Heterogeneity"]
K_Daridorexant_5mg:O_Placebo	1	No concerns	Low risk	No concerns	Some concerns	Some concerns	No concerns	Low	["Imprecision","Heterogeneity"]
L_Daridorexant_10mg:O_Placebo	1	No concerns	Low risk	No concerns	Some concerns	Some concerns	No concerns	Low	["Imprecision","Heterogeneity"]
M_Daridorexant_25mg:O_Placebo	1	No concerns	Low risk	No concerns	Some concerns	Some concerns	No concerns	Low	["Imprecision","Heterogeneity"]
N_Daridorexant_50mg:O_Placebo	1	No concerns	Low risk	No concerns	Some concerns	Some concerns	No concerns	Low	["Imprecision","Heterogeneity"]
**8. AEs**									
A_Suvorexant_10mg:O_Placebo	1	No concerns	Low risk	No concerns	Major concerns	No concerns	No concerns	Low	["Imprecision"]
B_Suvorexant_20mg:O_Placebo	1	No concerns	Low risk	No concerns	Major concerns	No concerns	No concerns	Low	["Imprecision"]
C_Suvorexant_40mg:O_Placebo	1	No concerns	Low risk	No concerns	Some concerns	No concerns	No concerns	Moderate	["Imprecision"]
D_Suvorexant_80mg:O_Placebo	1	No concerns	Low risk	No concerns	No concerns	No concerns	No concerns	High	[]
E_Lemborexant_1mg:O_Placebo	1	Some concerns	Low risk	No concerns	Major concerns	No concerns	No concerns	Very low	["Within-study bias","Imprecision"]
F_Lemborexant_2_5mg:O_Placebo	1	Some concerns	Low risk	No concerns	Major concerns	No concerns	No concerns	Very low	["Within-study bias","Imprecision"]
G_Lemborexant_5mg:O_Placebo	2	No concerns	Low risk	No concerns	Some concerns	Some concerns	No concerns	Low	["Imprecision","Heterogeneity"]
H_Lemborexant_10mg:O_Placebo	2	No concerns	Low risk	No concerns	No concerns	Some concerns	No concerns	Moderate	["Heterogeneity"]
I_Lemborexant_15mg:O_Placebo	1	Some concerns	Low risk	No concerns	Some concerns	No concerns	No concerns	Low	["Within-study bias","Imprecision"]
J_Lemborexant_25mg:O_Placebo	1	Some concerns	Low risk	No concerns	No concerns	Some concerns	No concerns	Low	["Within-study bias","Heterogeneity"]
K_Daridorexant_5mg:O_Placebo	2	No concerns	Low risk	No concerns	Some concerns	Some concerns	No concerns	Low	["Imprecision","Heterogeneity"]
L_Daridorexant_10mg:O_Placebo	2	No concerns	Low risk	No concerns	Some concerns	Some concerns	No concerns	Low	["Imprecision","Heterogeneity"]
M_Daridorexant_25mg:O_Placebo	2	No concerns	Low risk	No concerns	Some concerns	Some concerns	No concerns	Low	["Imprecision","Heterogeneity"]

N_Daridorexant_50mg:O_Placebo	2	No concerns	Low risk	No concerns	Some concerns	Some concerns	No concerns	Low	["Imprecision","Heterogeneity"]
**9. SAEs**									
A_Suvorexant_10mg:O_Placebo	1	No concerns	Low risk	No concerns	Major concerns	No concerns	No concerns	Low	["Imprecision"]
B_Suvorexant_20mg:O_Placebo	1	No concerns	Low risk	No concerns	Major concerns	No concerns	No concerns	Low	["Imprecision"]
C_Suvorexant_40mg:O_Placebo	1	No concerns	Low risk	No concerns	Major concerns	No concerns	No concerns	Low	["Imprecision"]
D_Suvorexant_80mg:O_Placebo	1	No concerns	Low risk	No concerns	Major concerns	No concerns	No concerns	Low	["Imprecision"]
G_Lemborexant_5mg:O_Placebo	1	No concerns	Low risk	No concerns	Major concerns	No concerns	No concerns	Low	["Imprecision"]
H_Lemborexant_10mg:O_Placebo	1	No concerns	Low risk	No concerns	Major concerns	No concerns	No concerns	Low	["Imprecision"]
J_Lemborexant_25mg:O_Placebo	1	Some concerns	Low risk	No concerns	Major concerns	No concerns	No concerns	Very low	["Within-study bias","Imprecision"]
K_Daridorexant_5mg:O_Placebo	1	No concerns	Low risk	No concerns	Major concerns	No concerns	No concerns	Low	["Imprecision"]
L_Daridorexant_10mg:O_Placebo	1	No concerns	Low risk	No concerns	Major concerns	No concerns	No concerns	Low	["Imprecision"]
M_Daridorexant_25mg:O_Placebo	1	No concerns	Low risk	No concerns	Major concerns	No concerns	No concerns	Low	["Imprecision"]
N_Daridorexant_50mg:O_Placebo	1	No concerns	Low risk	No concerns	Major concerns	No concerns	No concerns	Low	["Imprecision"]

eTable 17: Net plot for each outcome in studies > 3 months follow-up.

LPS	sTSO	WASO
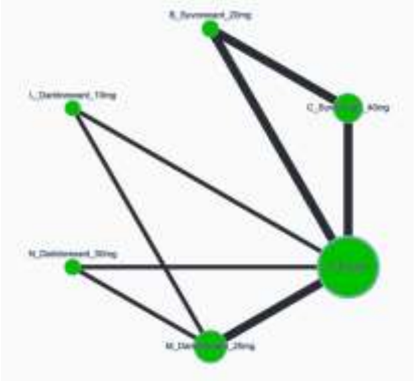	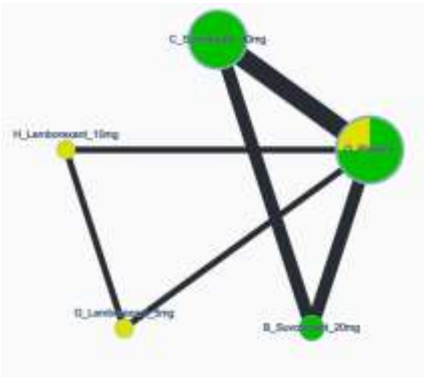	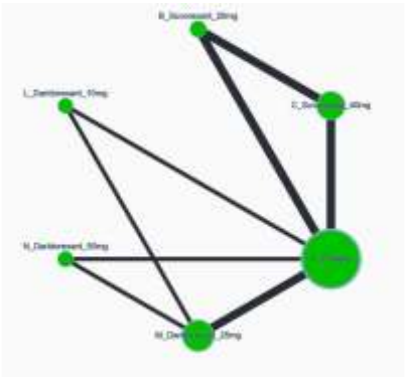
**sWASO**	**sTST**	**ISI**
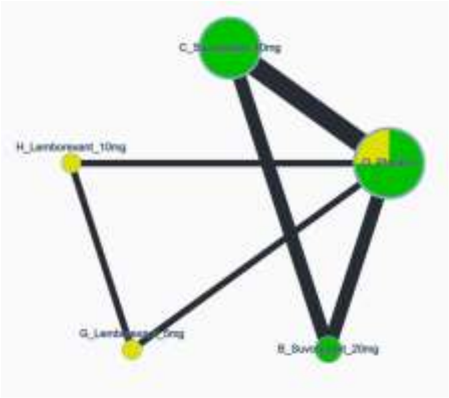	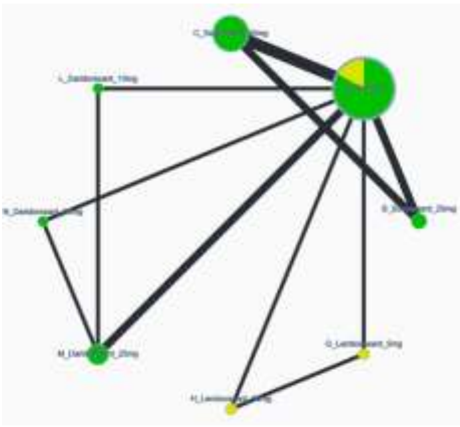	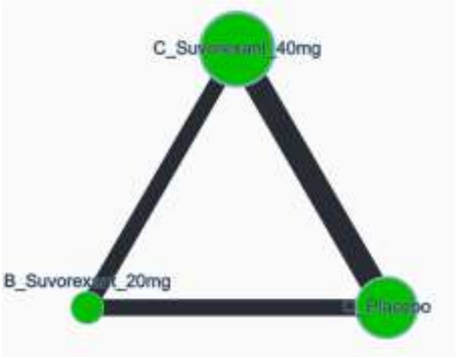
**AE**	**SAE**	
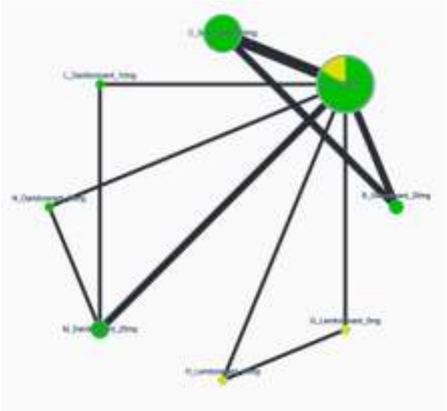	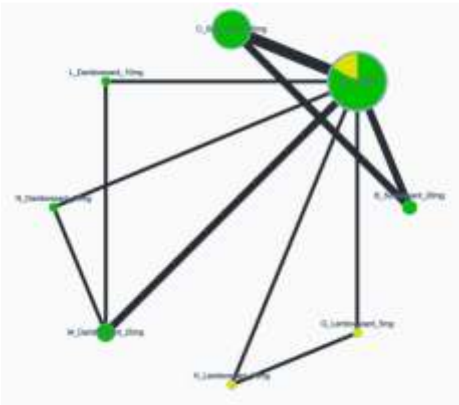	

eTable 18: Network meta-analysis results of LPS in studies > 3 months follow-up.

Suvorexant 20mg	1.782 (-3.028, 6.593)	-2.524 (-10.231, 5.182)	2.832 (-3.392, 9.055)	6.679 (-0.069, 13.427)	-5.280 (-10.061, -0.500)
-1.782 (-6.593, 3.028)	Suvorexant 40mg	-4.307 (-11.664, 3.051)	1.049 (-4.737, 6.835)	4.897 (-1.450, 11.243)	-7.063 (-11.257, -2.868)
2.524 (-5.182, 10.231)	4.307 (-3.051, 11.664)	Daridorexant 10mg	5.356 (-0.689, 11.401)	9.203 (2.049, 16.357)	-2.756 (-8.801, 3.289)
-2.832 (-9.055, 3.392)	-1.049 (-6.835, 4.737)	-5.356 (-11.401, 0.689)	Daridorexant 25mg	3.847 (-0.893, 8.588)	-8.112 (-12.097, -4.127)
-6.679 (-13.427, 0.069)	-4.897 (-11.243, 1.450)	-9.203 (-16.357, -2.049)	-3.847 (-8.588, 0.893)	Daridorexant 50mg	-11.959 (-16.722, -7.197)
5.280 (0.500, 10.061)	7.063 (2.868, 11.257)	2.756 (-3.289, 8.801)	8.112 (4.127, 12.097)	11.959 (7.197, 16.722)	Placebo

eTable 19: Network meta-analysis results of sTSO in studies > 3 months follow-up.

Suvorexant 20mg	3.976 (0.033, 7.918)	4.429 (-11.702, 20.559)	10.829 (-11.624, 33.281)	-5.951 (-9.903, -2.000)
-3.976 (-7.918, -0.033)	Suvorexant 40mg	0.453 (-15.502, 16.408)	6.853 (-15.474, 29.180)	-9.927 (-13.090, -6.764)
-4.429 (-20.559, 11.702)	-0.453 (-16.408, 15.502)	Lemborexant 5mg	6.400 (-15.320, 28.120)	-10.380 (-26.019, 5.259)
-10.829 (-33.281, 11.624)	-6.853 (-29.180, 15.474)	-6.400 (-28.120, 15.320)	Lemborexant 10mg	-16.780 (-38.882, 5.322)
5.951 (2.000, 9.903)	9.927 (6.764, 13.090)	10.380 (-5.259, 26.019)	16.780 (-5.322, 38.882)	Placebo

eTable 20: Network meta-analysis results of WASO in studies > 3 months follow-up.

Suvorexant 20mg	2.616 (-5.163, 10.394)	-21.135 (-33.005, -9.265)	-12.405 (-22.578, -2.233)	-5.637 (-17.074, 5.800)	-23.569 (-31.363, -15.774)
-2.616 (-10.394, 5.163)	Suvorexant 40mg	-23.750 (-35.197, -12.303)	-15.021 (-24.696, -5.346)	-8.253 (-19.251, 2.745)	-26.184 (-33.318, -19.051)
21.135 (9.265, 33.005)	23.750 (12.303, 35.197)	Daridorexant 10mg	8.729 (-0.211, 17.670)	15.498 (4.152, 26.844)	-2.434 (-11.386, 6.519)
12.405 (2.233, 22.578)	15.021 (5.346, 24.696)	-8.729 (-17.670, 0.211)	Daridorexant 25mg	6.768 (-1.602, 15.139)	-11.163 (-17.700, -4.627)
5.637 (-5.800, 17.074)	8.253 (-2.745, 19.251)	-15.498 (-26.844, -4.152)	-6.768 (-15.139, 1.602)	Daridorexant 50mg	-17.932 (-26.302, -9.561)
23.569 (15.774, 31.363)	26.184 (19.051, 33.318)	2.434 (-6.519, 11.386)	11.163 (4.627, 17.700)	17.932 (9.561, 26.302)	Placebo

eTable 21: Network meta-analysis results of sWASO in studies > 3 months follow-up.

Suvorexant 20mg	3.350 (-0.752, 7.451)	12.604 (8.450, 16.758)	7.804 (3.649, 11.958)	-4.866 (-8.982, -0.751)
-3.350 (-7.451, 0.752)	Suvorexant 40mg	9.254 (5.959, 12.549)	4.454 (1.159, 7.750)	-8.216 (-11.462, -4.970)
-12.604 (-16.758, -8.450)	-9.254 (-12.549, -5.959)	Lemborexant 5mg	-4.800 (-5.373, -4.226)	-17.470 (-18.036, -16.904)
-7.804 (-11.958, -3.649)	-4.454 (-7.750, -1.159)	4.800 (4.226, 5.373)	Lemborexant 10mg	-12.670 (-13.239, -12.101)
4.866 (0.751, 8.982)	8.216 (4.970, 11.462)	17.470 (16.904, 18.036)	12.670 (12.101, 13.239)	Placebo

eTable 22: Network meta-analysis results of sTST in studies > 3 months follow-up.

Suvorexant 20mg	-7.247 (-13.822, -0.671)	-2.609 (-9.260, 4.042)	-6.739 (-13.391, -0.088)	4.587 (-6.084, 15.258)	1.334 (-7.885, 10.552)
7.247 (0.671, 13.822)	Suvorexant 40mg	4.637 (-0.680, 9.955)	0.507 (-4.812, 5.826)	11.834 (1.938, 21.729)	8.580 (0.272, 16.889)
2.609 (-4.042, 9.260)	-4.637 (-9.955, 0.680)	Lemborexant 5mg	-4.130 (-4.865, -3.395)	7.196 (-1.211, 15.604)	3.943 (-2.521, 10.407)
6.739 (0.088, 13.391)	-0.507 (-5.826, 4.812)	4.130 (3.395, 4.865)	Lemborexant 10mg	11.326 (2.918, 19.735)	8.073 (1.608, 14.538)
-4.587 (-15.258, 6.084)	-11.834 (-21.729, -1.938)	-7.196 (-15.604, 1.211)	-11.326 (-19.735, -2.918)	Daridorexant 10mg	-3.253 (-11.630, 5.123)
-1.334 (-10.552, 7.885)	-8.580 (-16.889, -0.272)	-3.943 (-10.407, 2.521)	-8.073 (-14.538, -1.608)	3.253 (-5.123, 11.630)	Daridorexant 25mg
6.213 (-4.623, 17.048)	-1.034 (-11.106, 9.039)	3.604 (-5.011, 12.218)	-0.526 (-9.142, 8.089)	10.800 (-0.301, 21.901)	7.546 (-1.038, 16.131)
-15.941 (-22.552, -9.329)	-23.187 (-28.456, -17.919)	-18.550 (-19.271, -17.829)	-22.680 (-23.409, -21.951)	-11.354 (-19.730, -2.977)	-14.607 (-21.031, -8.183)

-6.213 (-17.048, 4.623)	15.941 (9.329, 22.552)
1.034 (-9.039, 11.106)	23.187 (17.919, 28.456)
-3.604 (-12.218, 5.011)	18.550 (17.829, 19.271)
0.526 (-8.089, 9.142)	22.680 (21.951, 23.409)
-10.800 (-21.901, 0.301)	11.354 (2.977, 19.730)

-7.546 (-16.131, 1.038)	14.607 (8.183, 21.031)
Daridorexant 50mg	22.154 (13.569, 30.738)
-22.154 (-30.738, -13.569)	Placebo

eTable 23: Network meta-analysis results of AEs in studies > 3 months follow-up.

Suvorexant 20mg	0.921 (0.822, 1.031)	1.023 (0.865, 1.210)	1.051 (0.887, 1.244)	0.873 (0.695, 1.096)	0.864 (0.715, 1.043)
1.086 (0.970, 1.216)	Suvorexant 40mg	1.111 (0.963, 1.283)	1.141 (0.987, 1.319)	0.948 (0.768, 1.170)	0.938 (0.794, 1.109)
0.977 (0.826, 1.155)	0.900 (0.780, 1.038)	Lemborexant 5mg	1.027 (0.904, 1.166)	0.853 (0.677, 1.075)	0.844 (0.696, 1.024)
0.952 (0.804, 1.127)	0.876 (0.758, 1.013)	0.974 (0.858, 1.106)	Lemborexant 10mg	0.831 (0.658, 1.048)	0.822 (0.677, 0.999)
1.146 (0.912, 1.439)	1.055 (0.855, 1.302)	1.173 (0.930, 1.478)	1.204 (0.954, 1.519)	Daridorexant 10mg	0.990 (0.820, 1.195)
1.157 (0.958, 1.398)	1.066 (0.902, 1.260)	1.184 (0.976, 1.437)	1.216 (1.001, 1.478)	1.010 (0.837, 1.219)	Daridorexant 25mg
1.134 (0.904, 1.423)	1.045 (0.847, 1.288)	1.161 (0.922, 1.462)	1.192 (0.945, 1.503)	0.990 (0.770, 1.273)	0.980 (0.810, 1.186)
1.002 (0.893, 1.124)	0.923 (0.856, 0.994)	1.025 (0.907, 1.159)	1.053 (0.930, 1.192)	0.874 (0.718, 1.064)	0.866 (0.745, 1.006)

0.882 (0.703, 1.106)	0.998 (0.890, 1.119)
0.957 (0.776, 1.180)	1.084 (1.006, 1.168)
0.861 (0.684, 1.085)	0.975 (0.863, 1.102)
0.839 (0.665, 1.058)	0.950 (0.839, 1.076)
1.010 (0.786, 1.298)	1.144 (0.939, 1.392)
1.020 (0.843, 1.235)	1.155 (0.995, 1.342)
Daridorexant 50mg	1.132 (0.931, 1.377)
0.883 (0.726, 1.074)	Placebo

eTable 24: Network meta-analysis results of SAEs in studies > 3 months follow-up.

Suvorexant 20mg	0.521 (0.131, 2.071)	0.252 (0.036, 1.768)	0.196 (0.029, 1.333)	0.589 (0.072, 4.821)	0.766 (0.126, 4.646)	0.723 (0.094, 5.555)	0.359 (0.098, 1.311)
1.921 (0.483, 7.644)	Suvorexant 40mg	0.485 (0.091, 2.585)	0.377 (0.073, 1.938)	1.131 (0.178, 7.212)	1.471 (0.327, 6.617)	1.388 (0.234, 8.236)	0.690 (0.301, 1.580)
3.961 (0.565, 27.752)	2.062 (0.387, 10.991)	Lemborexant 5mg	0.778 (0.206, 2.943)	2.333 (0.258, 21.132)	3.034 (0.445, 20.691)	2.863 (0.336, 24.420)	1.422 (0.332, 6.085)
5.093 (0.750, 34.570)	2.651 (0.516, 13.621)	1.286 (0.340, 4.865)	Lemborexant 10mg	3.000 (0.341, 26.425)	3.901 (0.591, 25.765)	3.681 (0.444, 30.511)	1.829 (0.446, 7.497)
1.698 (0.207, 13.897)	0.884 (0.139, 5.633)	0.429 (0.047, 3.882)	0.333 (0.038, 2.936)	Daridorexant 10mg	1.300 (0.236, 7.158)	1.227 (0.137, 11.018)	0.610 (0.116, 3.194)
1.306 (0.215, 7.920)	0.680 (0.151, 3.057)	0.330 (0.048, 2.248)	0.256 (0.039, 1.693)	0.769 (0.140, 4.233)	Daridorexant 25mg	0.944 (0.160, 5.556)	0.469 (0.134, 1.643)
1.384 (0.180, 10.634)	0.720 (0.121, 4.273)	0.349 (0.041, 2.979)	0.272 (0.033, 2.252)	0.815 (0.091, 7.317)	1.060 (0.180, 6.239)	Daridorexant 50mg	0.497 (0.103, 2.401)
2.785 (0.763, 10.168)	1.450 (0.633, 3.322)	0.703 (0.164, 3.008)	0.547 (0.133, 2.242)	1.640 (0.313, 8.595)	2.133 (0.609, 7.477)	2.013 (0.416, 9.729)	Placebo

eTable 25: Detailed certainty of evidence for each outcome in studies > 3 months follow-up.

Comparison	Number of studies	Within-study bias	Reporting bias	Indirectness	Imprecision	Heterogeneity	Incoherence	Confidence rating	Reason(s) for downgrading
**1. LPS**									
B_Suvorexant_20mg:O_Placebo	2	No concerns	Low risk	No concerns	No concerns	Major concerns	No concerns	Low	["Heterogeneity"]
C_Suvorexant_40mg:O_Placebo	2	No concerns	Low risk	No concerns	No concerns	Some concerns	No concerns	Moderate	["Heterogeneity"]
L_Daridorexant_10mg:O_Placebo	1	No concerns	Low risk	No concerns	Some concerns	Some concerns	No concerns	Low	["Imprecision","Heterogeneity"]
M_Daridorexant_25mg:O_Placebo	2	No concerns	Low risk	No concerns	No concerns	Some concerns	No concerns	Moderate	["Heterogeneity"]
N_Daridorexant_50mg:O_Placebo	1	No concerns	Low risk	No concerns	No concerns	No concerns	No concerns	High	[]
**2. sTSO**									
B_Suvorexant_20mg:O_Placebo	2	No concerns	Low risk	No concerns	No concerns	Some concerns	No concerns	Moderate	["Heterogeneity"]
C_Suvorexant_40mg:O_Placebo	3	No concerns	Low risk	No concerns	No concerns	No concerns	No concerns	High	[]
G_Lemborexant_5mg:O_Placebo	1	Some concerns	Low risk	No concerns	Major concerns	No concerns	No concerns	Very low	["Within-study bias","Imprecision"]
H_Lemborexant_10mg:O_Placebo	1	Some concerns	Low risk	No concerns	Major concerns	No concerns	No concerns	Very low	["Within-study bias","Imprecision"]
**3. WASO**									
B_Suvorexant_20mg:O_Placebo	2	No concerns	Low risk	No concerns	No concerns	No concerns	No concerns	High	[]
C_Suvorexant_40mg:O_Placebo	2	No concerns	Low risk	No concerns	No concerns	No concerns	No concerns	High	[]
L_Daridorexant_10mg:O_Placebo	1	No concerns	Low risk	No concerns	Some concerns	Some concerns	No concerns	Low	["Imprecision","Heterogeneity"]
M_Daridorexant_25mg:O_Placebo	2	No concerns	Low risk	No concerns	No concerns	Some concerns	No concerns	Moderate	["Heterogeneity"]
N_Daridorexant_50mg:O_Placebo	1	No concerns	Low risk	No concerns	No concerns	Some concerns	No concerns	Moderate	["Heterogeneity"]
**4. sWASO**									
B_Suvorexant_20mg:O_Placebo	2	No concerns	Low risk	No concerns	No concerns	Some concerns	No concerns	Moderate	["Heterogeneity"]
C_Suvorexant_40mg:O_Placebo	3	No concerns	Low risk	No concerns	No concerns	No concerns	No concerns	High	[]
G_Lemborexant_5mg:O_Placebo	1	Some concerns	Low risk	No concerns	No concerns	No concerns	No concerns	Moderate	["Within-study bias"]
H_Lemborexant_10mg:O_Placebo	1	Some concerns	Low risk	No concerns	No concerns	No concerns	No concerns	Moderate	["Within-study bias"]
**5. sTST**									
B_Suvorexant_20mg:O_Placebo	2	No concerns	Low risk	No concerns	No concerns	No concerns	No concerns	High	[]
C_Suvorexant_40mg:O_Placebo	3	No concerns	Low risk	No concerns	No concerns	No concerns	No concerns	High	[]

G_Lemborexant_5mg:O_Placebo	1	Some concerns	Low risk	No concerns	No concerns	Some concerns	No concerns	Low	["Within-study bias","Heterogeneity"]
H_Lemborexant_10mg:O_Placebo	1	Some concerns	Low risk	No concerns	No concerns	No concerns	No concerns	Moderate	["Within-study bias"]
L_Daridorexant_10mg:O_Placebo	1	No concerns	Low risk	No concerns	No concerns	Some concerns	No concerns	Moderate	["Heterogeneity"]
M_Daridorexant_25mg:O_Placebo	2	No concerns	Low risk	No concerns	No concerns	Some concerns	No concerns	Moderate	["Heterogeneity"]
N_Daridorexant_50mg:O_Placebo	1	No concerns	Low risk	No concerns	No concerns	No concerns	No concerns	High	[]
**6. ISI**									
B_Suvorexant_20mg:O_Placebo	2	No concerns	Low risk	No concerns	No concerns	Some concerns	No concerns	Moderate	["Heterogeneity"]
C_Suvorexant_40mg:O_Placebo	3	No concerns	Low risk	No concerns	No concerns	Some concerns	No concerns	Moderate	["Heterogeneity"]
**7. AE**									
B_Suvorexant_20mg:O_Placebo	2	No concerns	Low risk	No concerns	No concerns	No concerns	No concerns	High	[]
C_Suvorexant_40mg:O_Placebo	3	No concerns	Low risk	No concerns	No concerns	No concerns	No concerns	High	[]
G_Lemborexant_5mg:O_Placebo	1	Some concerns	Low risk	No concerns	No concerns	No concerns	No concerns	Moderate	["Within-study bias"]
H_Lemborexant_10mg:O_Placebo	1	Some concerns	Low risk	No concerns	No concerns	Some concerns	No concerns	Low	["Within-study bias","Heterogeneity"]
L_Daridorexant_10mg:O_Placebo	1	No concerns	Low risk	No concerns	Some concerns	No concerns	No concerns	Moderate	["Imprecision"]
M_Daridorexant_25mg:O_Placebo	2	No concerns	Low risk	No concerns	Some concerns	No concerns	No concerns	Moderate	["Imprecision"]
N_Daridorexant_50mg:O_Placebo	1	No concerns	Low risk	No concerns	Some concerns	No concerns	No concerns	Moderate	["Imprecision"]
**8. SAE**									
B_Suvorexant_20mg:O_Placebo	2	No concerns	Low risk	No concerns	Major concerns	No concerns	No concerns	Low	["Imprecision"]
C_Suvorexant_40mg:O_Placebo	3	No concerns	Low risk	No concerns	Major concerns	No concerns	No concerns	Low	["Imprecision"]
G_Lemborexant_5mg:O_Placebo	1	Some concerns	Low risk	No concerns	Major concerns	No concerns	No concerns	Very low	["Within-study bias","Imprecision"]
H_Lemborexant_10mg:O_Placebo	1	Some concerns	Low risk	No concerns	Major concerns	No concerns	No concerns	Very low	["Within-study bias","Imprecision"]
L_Daridorexant_10mg:O_Placebo	1	No concerns	Low risk	No concerns	Major concerns	No concerns	No concerns	Low	["Imprecision"]
M_Daridorexant_25mg:O_Placebo	2	No concerns	Low risk	No concerns	Major concerns	No concerns	No concerns	Low	["Imprecision"]
N_Daridorexant_50mg:O_Placebo	1	No concerns	Low risk	No concerns	Major concerns	No concerns	No concerns	Low	["Imprecision"]

eTable 26: Net plot for each outcome in elderly people.

LPS	sTSO	WASO
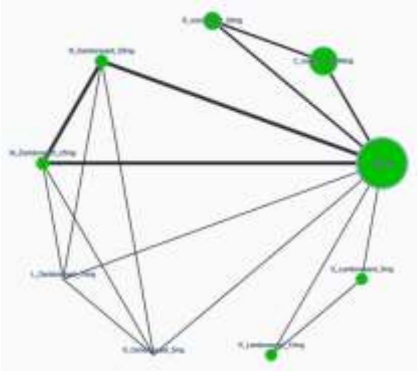	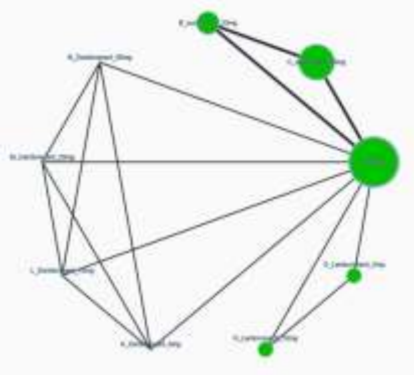	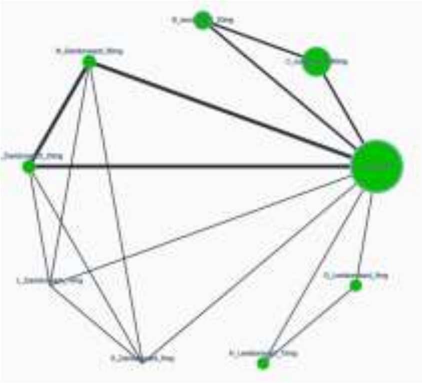
**sWASO**	**sTST**	**AE**
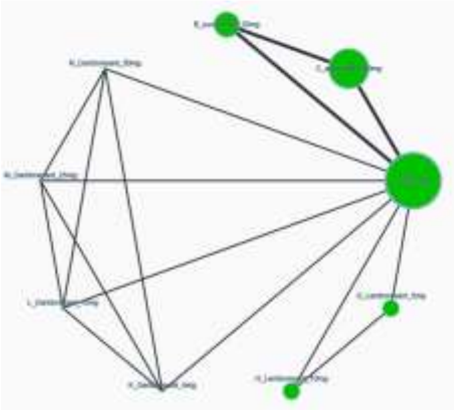	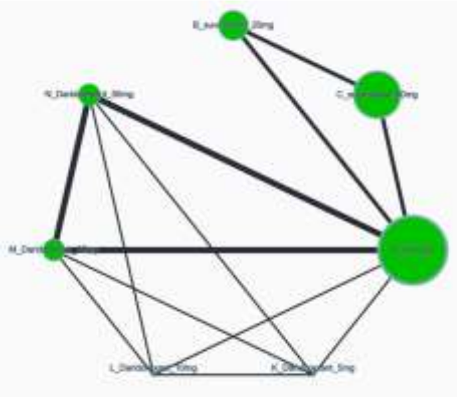	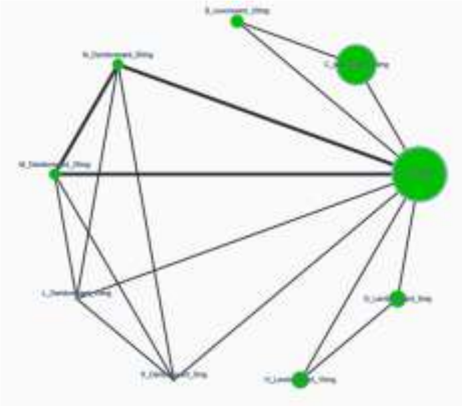
**SAE**		
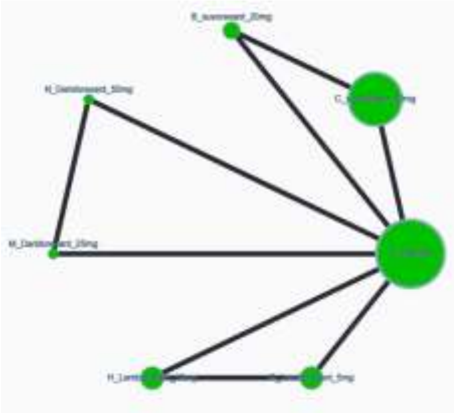		

eTable 27: Network meta-analysis results of LPS in elderly people.

Suvorexant 20mg	1.601 (-4.970, 8.171)	6.027 (-2.098, 14.151)	8.027 (-0.046, 16.100)	-1.588 (-17.201, 14.026)	5.112 (-9.092, 19.317)	2.616 (-4.686, 9.918)	8.387 (1.092, 15.681)	-5.573 (-11.165, 0.019)
-1.601 (-8.171, 4.970)	Suvorexant 40mg	4.426 (-3.294, 12.146)	6.426 (-1.240, 14.092)	-3.188 (-18.595, 12.219)	3.512 (-10.465, 17.489)	1.015 (-5.834, 7.865)	6.786 (-0.056, 13.628)	-7.174 (-12.160, -2.187)
-6.027 (-14.151, 2.098)	-4.426 (-12.146, 3.294)	Lemborexant 5mg	2.000 (-3.551, 7.551)	-7.614 (-23.338, 8.110)	-0.914 (-15.240, 13.411)	-3.411 (-10.947, 4.124)	2.360 (-5.168, 9.888)	-11.600 (-17.494, -5.706)
-8.027 (-16.100, 0.046)	-6.426 (-14.092, 1.240)	-2.000 (-7.551, 3.551)	Lemborexant 10mg	-9.614 (-25.312, 6.083)	-2.914 (-17.211, 11.382)	-5.411 (-12.891, 2.069)	0.360 (-7.113, 7.833)	-13.600 (-19.423, -7.777)
1.588 (-14.026, 17.201)	3.188 (-12.219, 18.595)	7.614 (-8.110, 23.338)	9.614 (-6.083, 25.312)	Daridorexant 5mg	6.700 (-10.173, 23.573)	4.203 (-10.373, 18.779)	9.974 (-4.627, 24.575)	-3.986 (-18.563, 10.592)
-5.112 (-19.317, 9.092)	-3.512 (-17.489, 10.465)	0.914 (-13.411, 15.240)	2.914 (-11.382, 17.211)	-6.700 (-23.573, 10.173)	Daridorexant 10mg	-2.497 (-15.552, 10.559)	3.274 (-9.809, 16.357)	-10.686 (-23.743, 2.372)
-2.616 (-9.918, 4.686)	-1.015 (-7.865, 5.834)	3.411 (-4.124, 10.947)	5.411 (-2.069, 12.891)	-4.203 (-18.779, 10.373)	2.497 (-10.559, 15.552)	Daridorexant 25mg	5.771 (1.045, 10.497)	-8.189 (-12.885, -3.493)
-8.387 (-15.681, -1.092)	-6.786 (-13.628, 0.056)	-2.360 (-9.888, 5.168)	-0.360 (-7.833, 7.113)	-9.974 (-24.575, 4.627)	-3.274 (-16.357, 9.809)	-5.771 (-10.497, -1.045)	Daridorexant 50mg	-13.960 (-18.644, -9.276)
5.573 (-0.019, 11.165)	7.174 (2.187, 12.160)	11.600 (5.706, 17.494)	13.600 (7.777, 19.423)	3.986 (-10.592, 18.563)	10.686 (-2.372, 23.743)	8.189 (3.493, 12.885)	13.960 (9.276, 18.644)	Placebo

eTable 28: Network meta-analysis results of sTSO in elderly people.

Suvorexant 20mg	3.874 (-2.451, 10.199)	12.002 (4.125, 19.878)	11.601 (3.787, 19.416)	-4.098 (-18.270, 10.073)	4.502 (-10.069, 19.072)	4.002 (-10.570, 18.573)	7.902 (-7.095, 22.898)	-5.098 (-10.629, 0.432)
-3.874 (-10.199, 2.451)	Suvorexant 40mg	8.128 (0.588, 15.667)	7.728 (0.254, 15.202)	-7.972 (-21.959, 6.014)	0.628 (-13.763, 15.018)	0.128 (-14.264, 14.520)	4.028 (-10.794, 18.850)	-8.972 (-14.011, -3.933)
-12.002 (-19.878, -4.125)	-8.128 (-15.667, -0.588)	Lemborexant 5mg	-0.400 (-6.248, 5.448)	-16.100 (-30.302, -1.898)	-7.500 (-22.100, 7.100)	-8.000 (-22.601, 6.601)	-4.100 (-19.125, 10.925)	-17.100 (-22.709, -11.492)
-11.601 (-19.416, -3.787)	-7.728 (-15.202, -0.254)	0.400 (-5.448, 6.248)	Lemborexant 10mg	-15.700 (-29.867, -1.533)	-7.100 (-21.666, 7.466)	-7.600 (-22.167, 6.967)	-3.700 (-18.693, 11.293)	-16.700 (-22.220, -11.180)
4.098 (-10.073, 18.270)	7.972 (-6.014, 21.959)	16.100 (1.898, 30.302)	15.700 (1.533, 29.867)	Daridorexant 5mg	8.600 (-2.946, 20.146)	8.100 (-3.447, 19.647)	12.000 (-0.079, 24.079)	-1.000 (-14.047, 12.047)
-4.502 (-19.072, 10.069)	-0.628 (-15.018, 13.763)	7.500 (-7.100, 22.100)	7.100 (-7.466, 21.666)	-8.600 (-20.146, 2.946)	Daridorexant 10mg	-0.500 (-12.534, 11.534)	3.400 (-9.145, 15.945)	-9.600 (-23.080, 3.880)
-4.002 (-18.573, 10.570)	-0.128 (-14.520, 14.264)	8.000 (-6.601, 22.601)	7.600 (-6.967, 22.167)	-8.100 (-19.647, 3.447)	0.500 (-11.534, 12.534)	Daridorexant 25mg	3.900 (-8.646, 16.446)	-9.100 (-22.581, 4.381)
-7.902 (-22.898, 7.095)	-4.028 (-18.850, 10.794)	4.100 (-10.925, 19.125)	3.700 (-11.293, 18.693)	-12.000 (-24.079, 0.079)	-3.400 (-15.945, 9.145)	-3.900 (-16.446, 8.646)	Daridorexant 50mg	-13.000 (-26.939, 0.940)
5.098 (-0.432, 10.629)	8.972 (3.933, 14.011)	17.100 (11.492, 22.709)	16.700 (11.180, 22.220)	1.000 (-12.047, 14.047)	9.600 (-3.880, 23.080)	9.100 (-4.381, 22.581)	13.000 (-0.940, 26.939)	Placebo

eTable 29: Network meta-analysis results of WASO in elderly people.

Suvorexant 20mg	3.040 (-7.666, 13.746)	0.138 (-15.151, 15.427)	2.638 (-12.654, 17.930)	-32.695 (-52.511, -12.878)	-18.695 (-36.806, -0.583)	-7.419 (-20.329, 5.491)	0.515 (-12.350, 13.380)	-25.162 (-34.890, -15.434)
-3.040 (-13.746, 7.666)	Suvorexant 40mg	-2.902 (-17.833, 12.029)	-0.402 (-15.336, 14.532)	-35.735 (-55.276, -16.193)	-21.735 (-39.545, -3.925)	-10.459 (-22.942, 2.025)	-2.525 (-14.962, 9.913)	-28.202 (-37.356, -19.047)
-0.138 (-15.427, 15.151)	2.902 (-12.029, 17.833)	Lemborexant 5mg	2.500 (-8.862, 13.862)	-32.833 (-53.742, -11.924)	-18.833 (-38.134, 0.468)	-7.557 (-22.088, 6.975)	0.377 (-14.114, 14.869)	-25.300 (-37.095, -13.505)
-2.638 (-17.930, 12.654)	0.402 (-14.532, 15.336)	-2.500 (-13.862, 8.862)	Lemborexant 10mg	-35.333 (-56.244, -14.421)	-21.333 (-40.636, -2.030)	-10.057 (-24.591, 4.478)	-2.123 (-16.617, 12.372)	-27.800 (-39.599, -16.001)
32.695 (12.878, 52.511)	35.735 (16.193, 55.276)	32.833 (11.924, 53.742)	35.333 (14.421, 56.244)	Daridorexant 5mg	14.000 (-4.973, 32.973)	25.276 (8.044, 42.508)	33.210 (16.075, 50.346)	7.533 (-9.732, 24.798)
18.695 (0.583, 36.806)	21.735 (3.925, 39.545)	18.833 (-0.468, 38.134)	21.333 (2.030, 40.636)	-14.000 (-32.973, 4.973)	Daridorexant 10mg	11.276 (-3.965, 26.517)	19.210 (4.079, 34.341)	-6.467 (-21.744, 8.810)
7.419 (-5.491, 20.329)	10.459 (-2.025, 22.942)	7.557 (-6.975, 22.088)	10.057 (-4.478, 24.591)	-25.276 (-42.508, -8.044)	-11.276 (-26.517, 3.965)	Daridorexant 25mg	7.934 (-0.495, 16.363)	-17.743 (-26.230, -9.256)
-0.515 (-13.380, 12.350)	2.525 (-9.913, 14.962)	-0.377 (-14.869, 14.114)	2.123 (-12.372, 16.617)	-33.210 (-50.346, -16.075)	-19.210 (-34.341, -4.079)	-7.934 (-16.363, 0.495)	Daridorexant 50mg	-25.677 (-34.096, -17.258)
25.162 (15.434, 34.890)	28.202 (19.047, 37.356)	25.300 (13.505, 37.095)	27.800 (16.001, 39.599)	-7.533 (-24.798, 9.732)	6.467 (-8.810, 21.744)	17.743 (9.256, 26.230)	25.677 (17.258, 34.096)	Placebo

eTable 30: Network meta-analysis results of sWASO in elderly people.

Suvorexant 20mg	-0.888 (-6.912, 5.136)	-2.297 (-14.040, 9.447)	11.203 (-1.636, 24.043)	-2.797 (-29.454, 23.861)	3.603 (-21.940, 29.147)	16.603 (-8.927, 42.134)	16.003 (-10.422, 42.428)	-10.797 (-16.077, -5.516)
0.888 (-5.136, 6.912)	Suvorexant 40mg	-1.408 (-12.952, 10.135)	12.092 (-0.565, 24.748)	-1.908 (-28.479, 24.662)	4.492 (-20.960, 29.944)	17.492 (-7.947, 42.931)	16.892 (-9.445, 43.228)	-9.908 (-14.727, -5.089)
2.297 (-9.447, 14.040)	1.408 (-10.135, 12.952)	Lemborexant 5mg	13.500 (2.345, 24.655)	-0.500 (-28.656, 27.656)	5.900 (-21.203, 33.004)	18.900 (-8.191, 45.991)	18.300 (-9.636, 46.236)	-8.500 (-18.989, 1.989)
-11.203 (-24.043, 1.636)	-12.092 (-24.748, 0.565)	-13.500 (-24.655, -2.345)	Lemborexant 10mg	-14.000 (-42.630, 14.630)	-7.600 (-35.196, 19.996)	5.400 (-22.184, 32.984)	4.800 (-23.614, 33.214)	-22.000 (-33.703, -10.297)
2.797 (-23.861, 29.454)	1.908 (-24.662, 28.479)	0.500 (-27.656, 28.656)	14.000 (-14.630, 42.630)	Daridorexant 5mg	6.400 (-18.342, 31.142)	19.400 (-5.329, 44.129)	18.800 (-6.851, 44.451)	-8.000 (-34.130, 18.130)
-3.603 (-29.147, 21.940)	-4.492 (-29.944, 20.960)	-5.900 (-33.004, 21.203)	7.600 (-19.996, 35.196)	-6.400 (-31.142, 18.342)	Daridorexant 10mg	13.000 (-10.523, 36.523)	12.400 (-12.091, 36.891)	-14.400 (-39.392, 10.591)
-16.603 (-42.134, 8.927)	-17.492 (-42.931, 7.947)	-18.900 (-45.991, 8.191)	-5.400 (-32.984, 22.184)	-19.400 (-44.129, 5.329)	-13.000 (-36.523, 10.523)	Daridorexant 25mg	-0.600 (-25.078, 23.878)	-27.400 (-52.378, -2.422)
-16.003 (-42.428, 10.422)	-16.892 (-43.228, 9.445)	-18.300 (-46.236, 9.636)	-4.800 (-33.214, 23.614)	-18.800 (-44.451, 6.851)	-12.400 (-36.891, 12.091)	0.600 (-23.878, 25.078)	Daridorexant 50mg	-26.800 (-52.692, -0.908)
10.797 (5.516, 16.077)	9.908 (5.089, 14.727)	8.500 (-1.989, 18.989)	22.000 (10.297, 33.703)	8.000 (-18.130, 34.130)	14.400 (-10.591, 39.392)	27.400 (2.422, 52.378)	26.800 (0.908, 52.692)	Placebo

eTable 31: Network meta-analysis results of sTST in elderly people.

Suvorexant 20mg	-3.460 (-13.000, 6.081)	26.992 (4.500, 49.483)	6.392 (-16.186, 28.969)	-0.467 (-12.390, 11.456)	-14.105 (-26.067, -2.142)	17.061 (8.733, 25.390)
3.460 (-6.081, 13.000)	Suvorexant 40mg	30.451 (8.214, 52.688)	9.851 (-12.473, 32.175)	2.993 (-8.442, 14.428)	-10.645 (-22.121, 0.831)	20.521 (12.908, 28.134)
-26.992 (-49.483, -4.500)	-30.451 (-52.688, -8.214)	Daridorexant 5mg	-20.600 (-45.568, 4.368)	-27.458 (-48.111, -6.806)	-41.096 (-61.969, -20.223)	-9.930 (-30.823, 10.963)
-6.392 (-28.969, 16.186)	-9.851 (-32.175, 12.473)	20.600 (-4.368, 45.568)	Daridorexant 10mg	-6.858 (-27.605, 13.888)	-20.496 (-41.462, 0.470)	10.670 (-10.316, 31.655)
0.467 (-11.456, 12.390)	-2.993 (-14.428, 8.442)	27.458 (6.806, 48.111)	6.858 (-13.888, 27.605)	Daridorexant 25mg	-13.638 (-22.179, -5.096)	17.528 (8.996, 26.060)
14.105 (2.142, 26.067)	10.645 (-0.831, 22.121)	41.096 (20.223, 61.969)	20.496 (-0.470, 41.462)	13.638 (5.096, 22.179)	Daridorexant 50mg	31.166 (22.578, 39.753)
-17.061 (-25.390, -8.733)	-20.521 (-28.134, -12.908)	9.930 (-10.963, 30.823)	-10.670 (-31.655, 10.316)	-17.528 (-26.060, -8.996)	-31.166 (-39.753, -22.578)	Placebo

eTable 32: Network meta-analysis results of AEs in elderly people.

Suvorexant 20mg	0.972 (0.828, 1.142)	0.903 (0.639, 1.276)	0.821 (0.585, 1.152)	0.820 (0.437, 1.538)	0.857 (0.449, 1.634)	0.938 (0.643, 1.368)	0.789 (0.548, 1.136)	0.991 (0.838, 1.172)
1.028 (0.876, 1.208)	Suvorexant 40mg	0.929 (0.671, 1.286)	0.844 (0.615, 1.160)	0.843 (0.455, 1.564)	0.881 (0.467, 1.663)	0.964 (0.673, 1.381)	0.811 (0.574, 1.146)	1.019 (0.904, 1.149)
1.107 (0.783, 1.565)	1.077 (0.778, 1.491)	Lemborexant 5mg	0.909 (0.698, 1.185)	0.908 (0.461, 1.787)	0.949 (0.474, 1.897)	1.038 (0.659, 1.635)	0.874 (0.561, 1.361)	1.097 (0.811, 1.485)
1.218 (0.868, 1.709)	1.184 (0.862, 1.627)	1.100 (0.844, 1.433)	Lemborexant 10mg	0.999 (0.509, 1.959)	1.043 (0.523, 2.079)	1.142 (0.729, 1.789)	0.961 (0.620, 1.488)	1.207 (0.899, 1.619)
1.219 (0.650, 2.287)	1.186 (0.639, 2.199)	1.101 (0.560, 2.168)	1.001 (0.511, 1.964)	Daridorexant 5mg	1.045 (0.524, 2.082)	1.144 (0.629, 2.079)	0.962 (0.541, 1.712)	1.208 (0.659, 2.214)
1.167 (0.612, 2.227)	1.135 (0.601, 2.143)	1.054 (0.527, 2.109)	0.959 (0.481, 1.911)	0.957 (0.480, 1.908)	Daridorexant 10mg	1.095 (0.591, 2.026)	0.921 (0.508, 1.670)	1.157 (0.620, 2.158)
1.066 (0.731, 1.556)	1.037 (0.724, 1.485)	0.963 (0.612, 1.517)	0.876 (0.559, 1.371)	0.875 (0.481, 1.590)	0.914 (0.494, 1.691)	Daridorexant 25mg	0.841 (0.614, 1.154)	1.057 (0.753, 1.482)
1.267 (0.880, 1.825)	1.232 (0.872, 1.741)	1.145 (0.735, 1.783)	1.041 (0.672, 1.612)	1.039 (0.584, 1.849)	1.086 (0.599, 1.968)	1.188 (0.866, 1.630)	Daridorexant 50mg	1.256 (0.908, 1.736)
1.009 (0.853, 1.194)	0.981 (0.871, 1.107)	0.912 (0.674, 1.234)	0.829 (0.617, 1.112)	0.828 (0.452, 1.517)	0.865 (0.463, 1.613)	0.946 (0.675, 1.328)	0.796 (0.576, 1.101)	Placebo

eTable 33: Network meta-analysis results of SAEs in elderly people.

Suvorexant 20mg	0.776 (0.221, 2.723)	0.161 (0.006, 4.302)	0.812 (0.013, 49.725)	1.466 (0.150, 14.326)	4.324 (0.174, 107.404)	0.633 (0.179, 2.246)
1.289 (0.367, 4.521)	Suvorexant 40mg	0.208 (0.009, 4.783)	1.046 (0.019, 57.014)	1.889 (0.240, 14.843)	5.572 (0.261, 119.009)	0.816 (0.363, 1.833)
6.207 (0.232, 165.737)	4.817 (0.209, 110.974)	Lemborexant 5mg	5.037 (0.243, 104.428)	9.097 (0.255, 324.764)	26.840 (0.390, 1846.782)	3.931 (0.190, 81.435)
1.232 (0.020, 75.498)	0.956 (0.018, 52.128)	0.199 (0.010, 4.115)	Lemborexant 10mg	1.806 (0.023, 140.008)	5.328 (0.040, 718.453)	0.780 (0.016, 39.158)
0.682 (0.070, 6.671)	0.530 (0.067, 4.162)	0.110 (0.003, 3.925)	0.554 (0.007, 42.936)	Daridorexant 25mg	2.951 (0.121, 71.708)	0.432 (0.065, 2.878)
0.231 (0.009, 5.744)	0.179 (0.008, 3.833)	0.037 (0.001, 2.564)	0.188 (0.001, 25.307)	0.339 (0.014, 8.237)	Daridorexant 50mg	0.146 (0.008, 2.805)
1.579 (0.445, 5.600)	1.225 (0.546, 2.753)	0.254 (0.012, 5.271)	1.282 (0.026, 64.322)	2.314 (0.347, 15.414)	6.828 (0.357, 130.791)	Placebo

eTable 34: Detailed certainty of evidence for each outcome in elderly people.

Comparison	Number of studies	Within-study bias	Reporting bias	Indirectness	Imprecision	Heterogeneity	Incoherence	Confidence rating	Reason(s) for downgrading
**1. LPS**									
B_suvorexant_20mg:O_Placebo	2	No concerns	Low risk	No concerns	Some concerns	No concerns	No concerns	Moderate	["Imprecision"]
C_suvorexant_40mg:O_Placebo	2	No concerns	Low risk	No concerns	No concerns	No concerns	No concerns	High	[]
G_Lemborexant_5mg:O_Placebo	1	No concerns	Low risk	No concerns	No concerns	No concerns	No concerns	High	[]
H_Lemborexant_10mg:O_Placebo	1	No concerns	Low risk	No concerns	No concerns	No concerns	No concerns	High	[]
K_Daridorexant_5mg:O_Placebo	1	No concerns	Low risk	No concerns	Major concerns	No concerns	No concerns	Low	["Imprecision"]
L_Daridorexant_10mg:O_Placebo	1	No concerns	Low risk	No concerns	Some concerns	Some concerns	No concerns	Low	["Imprecision","Heterogeneity"]
M_Daridorexant_25mg:O_Placebo	3	No concerns	Low risk	No concerns	No concerns	No concerns	No concerns	High	[]
N_Daridorexant_50mg:O_Placebo	3	No concerns	Low risk	No concerns	No concerns	No concerns	No concerns	High	[]
**2. sTSO**									
B_suvorexant_20mg:O_Placebo	2	No concerns	Low risk	No concerns	Some concerns	Some concerns	Some concerns	Very low	["Imprecision","Heterogeneity","Incoherence"]
C_suvorexant_40mg:O_Placebo	2	No concerns	Low risk	No concerns	No concerns	Major concerns	Some concerns	Very low	["Heterogeneity","Incoherence"]
G_Lemborexant_5mg:O_Placebo	1	No concerns	Low risk	No concerns	No concerns	Major concerns	Some concerns	Very low	["Heterogeneity","Incoherence"]
H_Lemborexant_10mg:O_Placebo	1	No concerns	Low risk	No concerns	No concerns	Major concerns	Some concerns	Very low	["Heterogeneity","Incoherence"]
K_Daridorexant_5mg:O_Placebo	1	No concerns	Low risk	No concerns	Major concerns	No concerns	Some concerns	Very low	["Imprecision","Incoherence"]
L_Daridorexant_10mg:O_Placebo	1	No concerns	Low risk	No concerns	Some concerns	Some concerns	Some concerns	Very low	["Imprecision","Heterogeneity","Incoherence"]
M_Daridorexant_25mg:O_Placebo	1	No concerns	Low risk	No concerns	Some concerns	Some concerns	Some concerns	Very low	["Imprecision","Heterogeneity","Incoherence"]
N_Daridorexant_50mg:O_Placebo	1	No concerns	Low risk	No concerns	Some concerns	Some concerns	Some concerns	Very low	["Imprecision","Heterogeneity","Incoherence"]
**3. WASO**									
B_suvorexant_20mg:O_Placebo	2	No concerns	Low risk	No concerns	No concerns	No concerns	Some concerns	Moderate	["Incoherence"]
C_suvorexant_40mg:O_Placebo	2	No concerns	Low risk	No concerns	No concerns	No concerns	Some concerns	Moderate	["Incoherence"]
G_Lemborexant_5mg:O_Placebo	1	No concerns	Low risk	No concerns	No concerns	No concerns	Some concerns	Moderate	["Incoherence"]
H_Lemborexant_10mg:O_Placebo	1	No concerns	Low risk	No concerns	No concerns	No concerns	Some concerns	Moderate	["Incoherence"]
K_Daridorexant_5mg:O_Placebo	1	No concerns	Low risk	No concerns	Some concerns	Some concerns	Some concerns	Very low	["Imprecision","Heterogeneity","Incoherence"]
L_Daridorexant_10mg:O_Placebo	1	No concerns	Low risk	No concerns	Some concerns	Some concerns	Major concerns	Very low	["Imprecision","Heterogeneity","Incoherence"]

M_Daridorexant_25mg:O_Placebo	3	No concerns	Low risk	No concerns	No concerns	No concerns	Some concerns	Moderate	["Incoherence"]
N_Daridorexant_50mg:O_Placebo	3	No concerns	Low risk	No concerns	No concerns	No concerns	Some concerns	Moderate	["Incoherence"]
**4. sWASO**									
B_suvorexant_20mg:O_Placebo	2	No concerns	Low risk	No concerns	No concerns	Major concerns	Some concerns	Very low	["Heterogeneity","Incoherence"]
C_suvorexant_40mg:O_Placebo	2	No concerns	Low risk	No concerns	No concerns	Major concerns	Some concerns	Very low	["Heterogeneity","Incoherence"]
G_Lemborexant_5mg:O_Placebo	1	No concerns	Low risk	No concerns	Some concerns	Some concerns	Some concerns	Very low	["Imprecision","Heterogeneity","Incoherence"]
H_Lemborexant_10mg:O_Placebo	1	No concerns	Low risk	No concerns	No concerns	Major concerns	Some concerns	Very low	["Heterogeneity","Incoherence"]
K_Daridorexant_5mg:O_Placebo	1	No concerns	Low risk	No concerns	Major concerns	No concerns	Some concerns	Very low	["Imprecision","Incoherence"]
L_Daridorexant_10mg:O_Placebo	1	No concerns	Low risk	No concerns	Major concerns	No concerns	Some concerns	Very low	["Imprecision","Incoherence"]
M_Daridorexant_25mg:O_Placebo	1	No concerns	Low risk	No concerns	No concerns	Major concerns	Some concerns	Very low	["Heterogeneity","Incoherence"]
N_Daridorexant_50mg:O_Placebo	1	No concerns	Low risk	No concerns	No concerns	Major concerns	Some concerns	Very low	["Heterogeneity","Incoherence"]
**5. sTST**									
B_suvorexant_20mg:O_Placebo	2	No concerns	Low risk	No concerns	No concerns	No concerns	No concerns	High	[]
C_suvorexant_40mg:O_Placebo	2	No concerns	Low risk	No concerns	No concerns	No concerns	No concerns	High	[]
K_Daridorexant_5mg:O_Placebo	1	No concerns	Low risk	No concerns	Some concerns	No concerns	No concerns	Moderate	["Imprecision"]
L_Daridorexant_10mg:O_Placebo	1	No concerns	Low risk	No concerns	Some concerns	No concerns	No concerns	Moderate	["Imprecision"]
M_Daridorexant_25mg:O_Placebo	3	No concerns	Low risk	No concerns	No concerns	No concerns	No concerns	High	[]
N_Daridorexant_50mg:O_Placebo	3	No concerns	Low risk	No concerns	No concerns	No concerns	No concerns	High	[]
**6. AEs**									
B_suvorexant_20mg:O_Placebo	1	No concerns	Low risk	No concerns	No concerns	Major concerns	No concerns	Low	["Heterogeneity"]
C_suvorexant_40mg:O_Placebo	1	No concerns	Low risk	No concerns	No concerns	Major concerns	No concerns	Low	["Heterogeneity"]
G_Lemborexant_5mg:O_Placebo	1	No concerns	Low risk	No concerns	Some concerns	Some concerns	No concerns	Low	["Imprecision","Heterogeneity"]
H_Lemborexant_10mg:O_Placebo	1	No concerns	Low risk	No concerns	Some concerns	Some concerns	No concerns	Low	["Imprecision","Heterogeneity"]
K_Daridorexant_5mg:O_Placebo	1	No concerns	Low risk	No concerns	Major concerns	No concerns	No concerns	Low	["Imprecision"]
L_Daridorexant_10mg:O_Placebo	1	No concerns	Low risk	No concerns	Major concerns	No concerns	No concerns	Low	["Imprecision"]
M_Daridorexant_25mg:O_Placebo	2	No concerns	Low risk	No concerns	Major concerns	No concerns	No concerns	Low	["Imprecision"]
N_Daridorexant_50mg:O_Placebo	2	No concerns	Low risk	No concerns	Some concerns	Some concerns	No concerns	Low	["Imprecision","Heterogeneity"]
**7. SAEs**									
B_suvorexant_20mg:O_Placebo	1	No concerns	Low risk	No concerns	Major concerns	No concerns	Some concerns	Very low	["Imprecision","Incoherence"]

C_suvorexant_40mg:O_Placebo	1	No concerns	Low risk	No concerns	Major concerns	No concerns	Some concerns	Very low	["Imprecision","Incoherence"]
G_Lemborexant_5mg:O_Placebo	1	No concerns	Low risk	No concerns	Major concerns	No concerns	Some concerns	Very low	["Imprecision","Incoherence"]
H_Lemborexant_10mg:O_Placebo	1	No concerns	Low risk	No concerns	Major concerns	No concerns	Some concerns	Very low	["Imprecision","Incoherence"]
M_Daridorexant_25mg:O_Placebo	1	No concerns	Low risk	No concerns	Major concerns	No concerns	Some concerns	Very low	["Imprecision","Incoherence"]
N_Daridorexant_50mg:O_Placebo	1	No concerns	Low risk	No concerns	Major concerns	No concerns	Some concerns	Very low	["Imprecision","Incoherence"]

eTable 35: Net plot for each outcome in double-blinded parallel-group studies.

**LPS**	**sTSO**	**WASO**
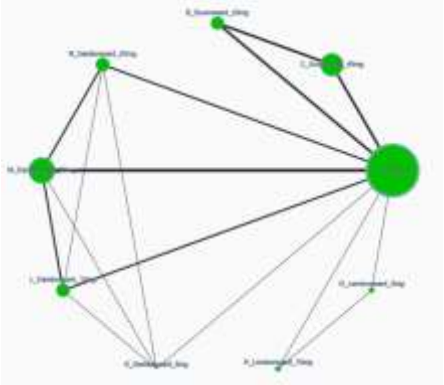	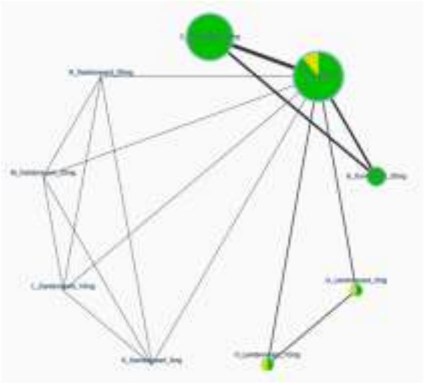	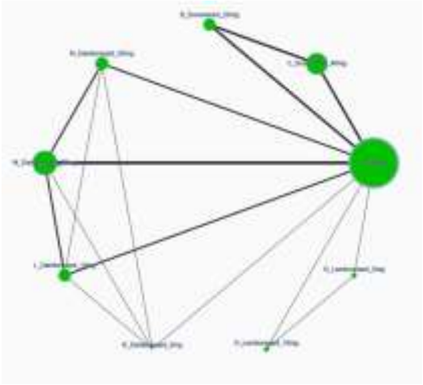
**sWASO**	**sTST**	**ISI**
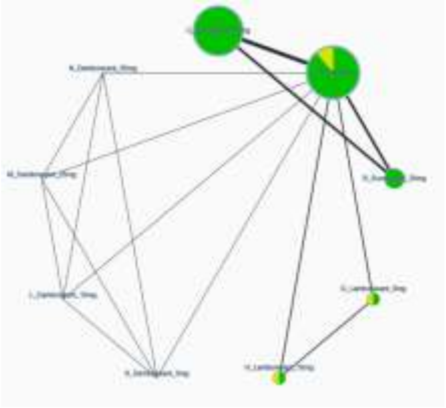	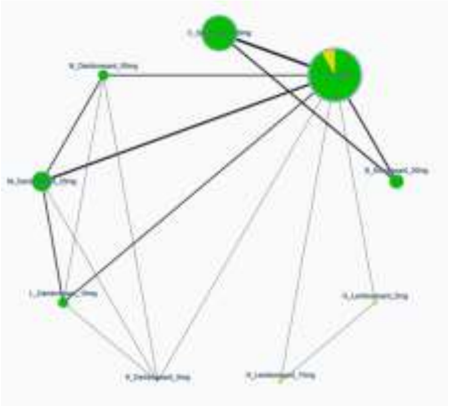	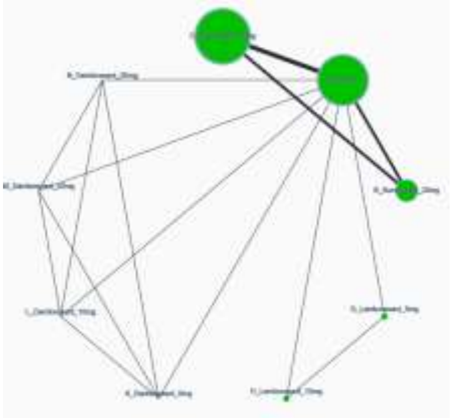
**AE**	**SAE**	
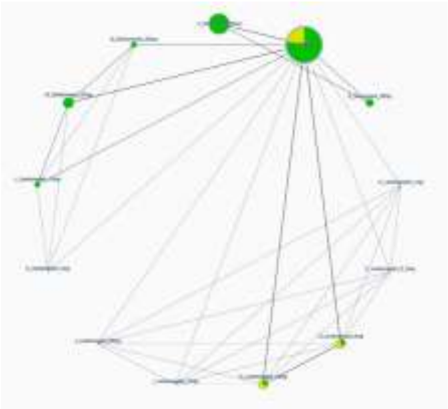	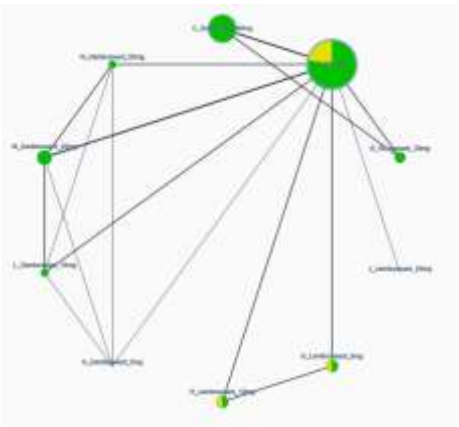	

eTable 36: Network meta-analysis results of LPS in double-blinded parallel-group studies.

Suvorexant 20mg	2.094 (-1.909, 6.096)	4.381 (-4.157, 12.920)	6.381 (-2.108, 14.871)	-16.055 (-21.947, -10.163)	0.142 (-5.132, 5.417)	1.178 (-3.756, 6.111)	1.657 (-3.498, 6.811)	-7.218 (-11.214, -3.223)
-2.094 (-6.096, 1.909)	Suvorexant 40mg	2.288 (-6.095, 10.670)	4.288 (-4.045, 12.620)	-18.149 (-23.811, -12.486)	-1.952 (-6.969, 3.066)	-0.916 (-5.574, 3.742)	-0.437 (-5.328, 4.454)	-9.312 (-12.961, -5.663)
-4.381 (-12.920, 4.157)	-2.288 (-10.670, 6.095)	Lemborexant 5mg	2.000 (-5.282, 9.282)	-20.436 (-29.137, -11.736)	-4.239 (-12.534, 4.056)	-3.204 (-11.286, 4.879)	-2.725 (-10.944, 5.495)	-11.600 (-19.146, -4.053)
-6.381 (-14.871, 2.108)	-4.288 (-12.620, 4.045)	-2.000 (-9.282, 5.282)	Lemborexant 10mg	-22.436 (-31.089, -13.784)	-6.239 (-14.484, 2.005)	-5.204 (-13.235, 2.827)	-4.725 (-12.893, 3.444)	-13.600 (-21.091, -6.109)
16.055 (10.163, 21.947)	18.149 (12.486, 23.811)	20.436 (11.736, 29.137)	22.436 (13.784, 31.089)	Daridorexant 5mg	16.197 (11.642, 20.753)	17.233 (12.903, 21.563)	17.712 (13.230, 22.194)	8.836 (4.506, 13.167)
-0.142 (-5.417, 5.132)	1.952 (-3.066, 6.969)	4.239 (-4.056, 12.534)	6.239 (-2.005, 14.484)	-16.197 (-20.753, -11.642)	Daridorexant 10mg	1.036 (-2.410, 4.481)	1.515 (-2.360, 5.390)	-7.361 (-10.804, -3.917)
-1.178 (-6.111, 3.756)	0.916 (-3.742, 5.574)	3.204 (-4.879, 11.286)	5.204 (-2.827, 13.235)	-17.233 (-21.563, -12.903)	-1.036 (-4.481, 2.410)	Daridorexant 25mg	0.479 (-2.776, 3.734)	-8.396 (-11.291, -5.502)
-1.657 (-6.811, 3.498)	0.437 (-4.454, 5.328)	2.725 (-5.495, 10.944)	4.725 (-3.444, 12.893)	-17.712 (-22.194, -13.230)	-1.515 (-5.390, 2.360)	-0.479 (-3.734, 2.776)	Daridorexant 50mg	-8.875 (-12.132, -5.619)
7.218 (3.223, 11.214)	9.312 (5.663, 12.961)	11.600 (4.053, 19.146)	13.600 (6.109, 21.091)	-8.836 (-13.167, -4.506)	7.361 (3.917, 10.804)	8.396 (5.502, 11.291)	8.875 (5.619, 12.132)	Placebo

eTable 37: Network meta-analysis results of sTSO in double-blinded parallel-group studies.

Suvorexant 20mg	3.832 (0.967, 6.698)	10.293 (4.284, 16.302)	10.530 (4.463, 16.598)	-9.820 (-12.881, -6.760)	-3.421 (-6.497, -0.344)	-5.620 (-8.687, -2.554)	-0.020 (-3.086, 3.045)	-6.021 (-8.893, -3.148)
-3.832 (-6.698, -0.967)	Suvorexant 40mg	6.460 (0.728, 12.193)	6.698 (0.905, 12.492)	-13.653 (-16.127, -11.179)	-7.253 (-9.746, -4.759)	-9.453 (-11.934, -6.971)	-3.853 (-6.332, -1.373)	-9.853 (-12.090, -7.615)
-10.293 (-16.302, -4.284)	-6.460 (-12.193, -0.728)	Lemborexant 5mg	0.238 (-5.381, 5.857)	-20.113 (-25.495, -14.731)	-13.713 (-19.105, -8.322)	-15.913 (-21.299, -10.527)	-10.313 (-15.698, -4.928)	-16.313 (-21.591, -11.035)
-10.530 (-16.598, -4.463)	-6.698 (-12.492, -0.905)	-0.238 (-5.857, 5.381)	Lemborexant 10mg	-20.351 (-25.798, -14.903)	-13.951 (-19.407, -8.495)	-16.151 (-21.602, -10.700)	-10.551 (-16.001, -5.101)	-16.551 (-21.895, -11.207)
9.820 (6.760, 12.881)	13.653 (11.179, 16.127)	20.113 (14.731, 25.495)	20.351 (14.903, 25.798)	Daridorexant 5mg	6.400 (5.316, 7.484)	4.200 (3.144, 5.256)	9.800 (8.749, 10.851)	3.800 (2.744, 4.856)
3.421 (0.344, 6.497)	7.253 (4.759, 9.746)	13.713 (8.322, 19.105)	13.951 (8.495, 19.407)	-6.400 (-7.484, -5.316)	Daridorexant 10mg	-2.200 (-3.301, -1.099)	3.400 (2.303, 4.497)	-2.600 (-3.701, -1.499)
5.620 (2.554, 8.687)	9.453 (6.971, 11.934)	15.913 (10.527, 21.299)	16.151 (10.700, 21.602)	-4.200 (-5.256, -3.144)	2.200 (1.099, 3.301)	Daridorexant 25mg	5.600 (4.531, 6.669)	-0.400 (-1.474, 0.674)
0.020 (-3.045, 3.086)	3.853 (1.373, 6.332)	10.313 (4.928, 15.698)	10.551 (5.101, 16.001)	-9.800 (-10.851, -8.749)	-3.400 (-4.497, -2.303)	-5.600 (-6.669, -4.531)	Daridorexant 50mg	-6.000 (-7.069, -4.931)
6.021 (3.148, 8.893)	9.853 (7.615, 12.090)	16.313 (11.035, 21.591)	16.551 (11.207, 21.895)	-3.800 (-4.856, -2.744)	2.600 (1.499, 3.701)	0.400 (-0.674, 1.474)	6.000 (4.931, 7.069)	Placebo

eTable 38: Network meta-analysis results of WASO in double-blinded parallel-group studies.

Suvorexant 20mg	2.556 (-3.328, 8.439)	0.820 (-11.661, 13.301)	3.320 (-9.164, 15.805)	-19.438 (-28.667, -10.209)	-18.097 (-25.915, -10.280)	-14.672 (-21.960, -7.383)	-7.017 (-14.754, 0.720)	-24.480 (-30.363, -18.597)
-2.556 (-8.439, 3.328)	Suvorexant 40mg	-1.735 (-14.019, 10.549)	0.765 (-11.523, 13.053)	-21.993 (-30.954, -13.032)	-20.653 (-28.152, -13.154)	-17.227 (-24.174, -10.281)	-9.573 (-16.988, -2.157)	-27.035 (-32.488, -21.582)
-0.820 (-13.301, 11.661)	1.735 (-10.549, 14.019)	Lemborexant 5mg	2.500 (-8.042, 13.042)	-20.258 (-33.362, -7.154)	-18.918 (-31.069, -6.766)	-15.492 (-27.311, -3.674)	-7.837 (-19.938, 4.263)	-25.300 (-36.307, -14.293)
-3.320 (-15.805, 9.164)	-0.765 (-13.053, 11.523)	-2.500 (-13.042, 8.042)	Lemborexant 10mg	-22.758 (-35.866, -9.650)	-21.418 (-33.573, -9.262)	-17.992 (-29.814, -6.170)	-10.337 (-22.441, 1.766)	-27.800 (-38.811, -16.789)
19.438 (10.209, 28.667)	21.993 (13.032, 30.954)	20.258 (7.154, 33.362)	22.758 (9.650, 35.866)	Daridorexant 5mg	1.340 (-6.096, 8.776)	4.766 (-2.345, 11.877)	12.421 (5.030, 19.811)	-5.042 (-12.153, 2.069)
18.097 (10.280, 25.915)	20.653 (13.154, 28.152)	18.918 (6.766, 31.069)	21.418 (9.262, 33.573)	-1.340 (-8.776, 6.096)	Daridorexant 10mg	3.426 (-1.722, 8.574)	11.080 (5.073, 17.087)	-6.382 (-11.530, -1.234)
14.672 (7.383, 21.960)	17.227 (10.281, 24.174)	15.492 (3.674, 27.311)	17.992 (6.170, 29.814)	-4.766 (-11.877, 2.345)	-3.426 (-8.574, 1.722)	Daridorexant 25mg	7.655 (2.630, 12.680)	-9.808 (-14.111, -5.505)
7.017 (-0.720, 14.754)	9.573 (2.157, 16.988)	7.837 (-4.263, 19.938)	10.337 (-1.766, 22.441)	-12.421 (-19.811, -5.030)	-11.080 (-17.087, -5.073)	-7.655 (-12.680, -2.630)	Daridorexant 50mg	-17.463 (-22.488, -12.438)
24.480 (18.597, 30.363)	27.035 (21.582, 32.488)	25.300 (14.293, 36.307)	27.800 (16.789, 38.811)	5.042 (-2.069, 12.153)	6.382 (1.234, 11.530)	9.808 (5.505, 14.111)	17.463 (12.438, 22.488)	Placebo

eTable 39: Network meta-analysis results of sWASO in double-blinded parallel-group studies.

Suvorexant 20mg	2.829 (-0.017, 5.676)	11.575 (8.662, 14.487)	6.823 (3.911, 9.736)	-2.167 (-5.394, 1.061)	-7.766 (-11.018, -4.515)	1.833 (-1.386, 5.053)	5.734 (2.500, 8.967)	-5.867 (-8.723, -3.010)
-2.829 (-5.676, 0.017)	Suvorexant 40mg	8.745 (6.506, 10.984)	3.994 (1.754, 6.233)	-4.996 (-7.633, -2.360)	-10.596 (-13.261, -7.931)	-0.996 (-3.622, 1.630)	2.904 (0.261, 5.547)	-8.696 (-10.862, -6.530)
-11.575 (-14.487, -8.662)	-8.745 (-10.984, -6.506)	Lemborexant 5mg	-4.751 (-5.324, -4.178)	-13.741 (-15.347, -12.135)	-19.341 (-20.994, -17.689)	-9.741 (-11.330, -8.152)	-5.841 (-7.458, -4.224)	-17.441 (-18.006, -16.876)
-6.823 (-9.736, -3.911)	-3.994 (-6.233, -1.754)	4.751 (4.178, 5.324)	Lemborexant 10mg	-8.990 (-10.597, -7.383)	-14.590 (-16.244, -12.936)	-4.990 (-6.580, -3.400)	-1.090 (-2.708, 0.528)	-12.690 (-13.258, -12.122)
2.167 (-1.061, 5.394)	4.996 (2.360, 7.633)	13.741 (12.135, 15.347)	8.990 (7.383, 10.597)	Daridorexant 5mg	-5.600 (-7.153, -4.047)	4.000 (2.515, 5.485)	7.900 (6.386, 9.415)	-3.700 (-5.203, -2.197)
7.766 (4.515, 11.018)	10.596 (7.931, 13.261)	19.341 (17.689, 20.994)	14.590 (12.936, 16.244)	5.600 (4.047, 7.153)	Daridorexant 10mg	9.600 (8.064, 11.136)	13.500 (11.936, 15.064)	1.900 (0.347, 3.453)
-1.833 (-5.053, 1.386)	0.996 (-1.630, 3.622)	9.741 (8.152, 11.330)	4.990 (3.400, 6.580)	-4.000 (-5.485, -2.515)	-9.600 (-11.136, -8.064)	Daridorexant 25mg	3.900 (2.403, 5.397)	-7.700 (-9.185, -6.215)
-5.734 (-8.967, -2.500)	-2.904 (-5.547, -0.261)	5.841 (4.224, 7.458)	1.090 (-0.528, 2.708)	-7.900 (-9.415, -6.386)	-13.500 (-15.064, -11.936)	-3.900 (-5.397, -2.403)	Daridorexant 50mg	-11.600 (-13.114, -10.085)
5.867 (3.010, 8.723)	8.696 (6.530, 10.862)	17.441 (16.876, 18.006)	12.690 (12.122, 13.258)	3.700 (2.197, 5.203)	-1.900 (-3.453, -0.347)	7.700 (6.215, 9.185)	11.600 (10.085, 13.114)	Placebo

eTable 40: Network meta-analysis results of sTST in double-blinded parallel-group studies.

Suvorexant 20mg	-5.679 (-10.111, -1.247)	-1.172 (-5.684, 3.341)	-5.302 (-9.816, -0.788)	18.136 (1.571, 34.701)	5.878 (-1.048, 12.803)	3.476 (-2.572, 9.524)	-5.572 (-12.495, 1.350)	17.378 (12.924, 21.833)
5.679 (1.247, 10.111)	Suvorexant 40mg	4.507 (1.073, 7.942)	0.377 (-3.059, 3.814)	23.815 (7.511, 40.120)	11.557 (5.280, 17.834)	9.155 (3.862, 14.448)	0.107 (-6.166, 6.380)	23.057 (19.699, 26.416)
1.172 (-3.341, 5.684)	-4.507 (-7.942, -1.073)	Lemborexant 5mg	-4.130 (-4.865, -3.395)	19.308 (3.337, 35.279)	7.050 (1.698, 12.402)	4.648 (0.494, 8.802)	-4.400 (-9.748, 0.947)	18.550 (17.829, 19.271)
5.302 (0.788, 9.816)	-0.377 (-3.814, 3.059)	4.130 (3.395, 4.865)	Lemborexant 10mg	23.438 (7.466, 39.410)	11.180 (5.827, 16.533)	8.778 (4.622, 12.934)	-0.270 (-5.619, 5.078)	22.680 (21.951, 23.409)
-18.136 (-34.701, -1.571)	-23.815 (-40.120, -7.511)	-19.308 (-35.279, -3.337)	-23.438 (-39.410, -7.466)	Daridorexant 5mg	-12.258 (-28.471, 3.955)	-14.660 (-30.622, 1.302)	-23.708 (-39.956, -7.460)	-0.758 (-16.713, 15.197)
-5.878 (-12.803, 1.048)	-11.557 (-17.834, -5.280)	-7.050 (-12.402, -1.698)	-11.180 (-16.533, -5.827)	12.258 (-3.955, 28.471)	Daridorexant 10mg	-2.402 (-7.707, 2.904)	-11.450 (-18.277, -4.623)	11.500 (6.197, 16.803)
-3.476 (-9.524, 2.572)	-9.155 (-14.448, -3.862)	-4.648 (-8.802, -0.494)	-8.778 (-12.934, -4.622)	14.660 (-1.302, 30.622)	2.402 (-2.904, 7.707)	Daridorexant 25mg	-9.048 (-14.349, -3.747)	13.902 (9.811, 17.993)
5.572 (-1.350, 12.495)	-0.107 (-6.380, 6.166)	4.400 (-0.947, 9.748)	0.270 (-5.078, 5.619)	23.708 (7.460, 39.956)	11.450 (4.623, 18.277)	9.048 (3.747, 14.349)	Daridorexant 50mg	22.950 (17.652, 28.249)
-17.378 (-21.833, -12.924)	-23.057 (-26.416, -19.699)	-18.550 (-19.271, -17.829)	-22.680 (-23.409, -21.951)	0.758 (-15.197, 16.713)	-11.500 (-16.803, -6.197)	-13.902 (-17.993, -9.811)	-22.950 (-28.249, -17.652)	Placebo

eTable 41: Network meta-analysis results of ISI in double-blinded parallel-group studies.

Suvorexant 20mg	0.407 (-0.060, 0.874)	0.496 (-0.772, 1.765)	0.596 (-0.702, 1.895)	-1.504 (-3.717, 0.709)	-0.804 (-2.964, 1.356)	-1.004 (-3.164, 1.156)	-0.404 (-2.626, 1.818)	-1.204 (-1.667, -0.741)
-0.407 (-0.874, 0.060)	Suvorexant 40mg	0.089 (-1.149, 1.328)	0.189 (-1.080, 1.458)	-1.911 (-4.107, 0.285)	-1.211 (-3.353, 0.932)	-1.411 (-3.554, 0.732)	-0.811 (-3.016, 1.394)	-1.611 (-1.984, -1.238)
-0.496 (-1.765, 0.772)	-0.089 (-1.328, 1.149)	Lemborexant 5mg	0.100 (-1.056, 1.256)	-2.000 (-4.466, 0.466)	-1.300 (-3.718, 1.118)	-1.500 (-3.918, 0.918)	-0.900 (-3.373, 1.573)	-1.700 (-2.881, -0.519)
-0.596 (-1.895, 0.702)	-0.189 (-1.458, 1.080)	-0.100 (-1.256, 1.056)	Lemborexant 10mg	-2.100 (-4.581, 0.381)	-1.400 (-3.834, 1.034)	-1.600 (-4.034, 0.834)	-1.000 (-3.489, 1.489)	-1.800 (-3.013, -0.587)
1.504 (-0.709, 3.717)	1.911 (-0.285, 4.107)	2.000 (-0.466, 4.466)	2.100 (-0.381, 4.581)	Daridorexant 5mg	0.700 (-1.546, 2.946)	0.500 (-1.746, 2.746)	1.100 (-1.206, 3.406)	0.300 (-1.864, 2.464)
0.804 (-1.356, 2.964)	1.211 (-0.932, 3.353)	1.300 (-1.118, 3.718)	1.400 (-1.034, 3.834)	-0.700 (-2.946, 1.546)	Daridorexant 10mg	-0.200 (-2.394, 1.994)	0.400 (-1.855, 2.655)	-0.400 (-2.510, 1.710)
1.004 (-1.156, 3.164)	1.411 (-0.732, 3.554)	1.500 (-0.918, 3.918)	1.600 (-0.834, 4.034)	-0.500 (-2.746, 1.746)	0.200 (-1.994, 2.394)	Daridorexant 25mg	0.600 (-1.655, 2.855)	-0.200 (-2.310, 1.910)
0.404 (-1.818, 2.626)	0.811 (-1.394, 3.016)	0.900 (-1.573, 3.373)	1.000 (-1.489, 3.489)	-1.100 (-3.406, 1.206)	-0.400 (-2.655, 1.855)	-0.600 (-2.855, 1.655)	Daridorexant 50mg	-0.800 (-2.973, 1.373)
1.204 (0.741, 1.667)	1.611 (1.238, 1.984)	1.700 (0.519, 2.881)	1.800 (0.587, 3.013)	-0.300 (-2.464, 1.864)	0.400 (-1.710, 2.510)	0.200 (-1.910, 2.310)	0.800 (-1.373, 2.973)	Placebo

eTable 42: Network meta-analysis results of AEs in double-blinded parallel-group studies.

Suvorexant 20mg	0.921 (0.822, 1.031)	1.361 (0.800, 2.315)	1.148 (0.689, 1.912)	1.003 (0.855, 1.176)	0.982 (0.838, 1.152)
1.086 (0.970, 1.216)	Suvorexant 40mg	1.478 (0.875, 2.496)	1.247 (0.754, 2.061)	1.089 (0.953, 1.244)	1.067 (0.933, 1.219)
0.735 (0.432, 1.250)	0.677 (0.401, 1.143)	Lemborexant 1mg	0.844 (0.436, 1.633)	0.737 (0.438, 1.239)	0.722 (0.430, 1.211)
0.871 (0.523, 1.450)	0.802 (0.485, 1.326)	1.185 (0.612, 2.294)	Lemborexant 2.5mg	0.873 (0.531, 1.436)	0.856 (0.521, 1.404)
0.997 (0.851, 1.170)	0.919 (0.804, 1.049)	1.357 (0.807, 2.282)	1.145 (0.696, 1.884)	Lemborexant 5mg	0.980 (0.877, 1.095)
1.018 (0.868, 1.194)	0.937 (0.820, 1.071)	1.385 (0.826, 2.324)	1.169 (0.712, 1.918)	1.021 (0.913, 1.141)	Lemborexant 10mg
1.184 (0.851, 1.645)	1.090 (0.793, 1.497)	1.610 (0.945, 2.745)	1.359 (0.814, 2.268)	1.187 (0.870, 1.618)	1.163 (0.856, 1.579)
1.283 (0.929, 1.772)	1.181 (0.865, 1.612)	1.745 (1.028, 2.964)	1.473 (0.886, 2.448)	1.286 (0.950, 1.742)	1.260 (0.934, 1.700)
1.107 (0.731, 1.676)	1.019 (0.679, 1.529)	1.506 (0.783, 2.898)	1.271 (0.672, 2.403)	1.110 (0.734, 1.678)	1.087 (0.719, 1.645)
1.162 (0.938, 1.440)	1.070 (0.880, 1.301)	1.581 (0.913, 2.739)	1.334 (0.786, 2.264)	1.165 (0.942, 1.440)	1.141 (0.923, 1.411)
1.167 (0.971, 1.403)	1.075 (0.914, 1.264)	1.588 (0.927, 2.721)	1.340 (0.799, 2.248)	1.170 (0.976, 1.402)	1.146 (0.956, 1.374)
1.129 (0.911, 1.398)	1.039 (0.855, 1.264)	1.536 (0.887, 2.660)	1.296 (0.764, 2.199)	1.132 (0.915, 1.399)	1.109 (0.897, 1.371)
1.002 (0.893, 1.124)	0.923 (0.856, 0.994)	1.363 (0.811, 2.290)	1.150 (0.700, 1.891)	1.004 (0.899, 1.122)	0.984 (0.881, 1.099)

0.845 (0.608, 1.174)	0.780 (0.564, 1.077)	0.903 (0.597, 1.368)	0.861 (0.695, 1.066)	0.857 (0.713, 1.030)	0.886 (0.715, 1.098)
0.918 (0.668, 1.261)	0.847 (0.620, 1.155)	0.981 (0.654, 1.472)	0.935 (0.768, 1.137)	0.930 (0.791, 1.094)	0.962 (0.791, 1.170)
0.621 (0.364, 1.059)	0.573 (0.337, 0.973)	0.664 (0.345, 1.277)	0.632 (0.365, 1.096)	0.630 (0.368, 1.079)	0.651 (0.376, 1.128)
0.736 (0.441, 1.228)	0.679 (0.409, 1.129)	0.787 (0.416, 1.488)	0.750 (0.442, 1.272)	0.746 (0.445, 1.252)	0.772 (0.455, 1.310)
0.843 (0.618, 1.149)	0.778 (0.574, 1.053)	0.901 (0.596, 1.363)	0.858 (0.694, 1.061)	0.855 (0.713, 1.024)	0.884 (0.715, 1.092)
0.860 (0.633, 1.168)	0.794 (0.588, 1.071)	0.920 (0.608, 1.391)	0.876 (0.709, 1.083)	0.872 (0.728, 1.046)	0.902 (0.730, 1.115)
Lemborexant 15mg	0.923 (0.666, 1.279)	1.069 (0.646, 1.770)	1.019 (0.712, 1.457)	1.014 (0.721, 1.425)	1.049 (0.733, 1.500)

1.084 (0.782, 1.502)	Lemborexant 25mg	1.159 (0.703, 1.911)	1.104 (0.776, 1.570)	1.099 (0.787, 1.535)	1.137 (0.799, 1.616)
0.935 (0.565, 1.549)	0.863 (0.523, 1.423)	Daridorexant 5mg	0.953 (0.636, 1.427)	0.948 (0.639, 1.407)	0.981 (0.654, 1.472)
0.982 (0.686, 1.404)	0.906 (0.637, 1.288)	1.050 (0.701, 1.572)	Daridorexant 10mg	0.996 (0.838, 1.183)	1.029 (0.824, 1.286)
0.986 (0.702, 1.386)	0.910 (0.651, 1.271)	1.054 (0.711, 1.565)	1.005 (0.845, 1.193)	Daridorexant 25mg	1.034 (0.868, 1.232)
0.954 (0.667, 1.364)	0.880 (0.619, 1.251)	1.020 (0.679, 1.530)	0.971 (0.778, 1.213)	0.967 (0.812, 1.152)	Daridorexant 50mg
0.847 (0.622, 1.153)	0.781 (0.577, 1.056)	0.905 (0.608, 1.348)	0.862 (0.719, 1.033)	0.858 (0.744, 0.991)	0.888 (0.741, 1.064)

0.998 (0.890, 1.119)
1.084 (1.006, 1.168)
0.734 (0.437, 1.232)
0.869 (0.529, 1.429)
0.996 (0.891, 1.112)
1.016 (0.910, 1.135)
1.181 (0.868, 1.609)
1.280 (0.947, 1.732)
1.105 (0.742, 1.646)
1.160 (0.968, 1.390)
1.165 (1.009, 1.345)
1.127 (0.940, 1.350)
Placebo

eTable 43: Network meta-analysis results of SAEs in double-blinded parallel-group studies.

Suvorexant 20mg	0.467 (0.142, 1.540)	0.206 (0.042, 0.997)	0.197 (0.041, 0.943)	0.313 (0.016, 6.199)	1.497 (0.060, 37.634)	0.391 (0.075, 2.030)	0.733 (0.155, 3.467)	0.627 (0.123, 3.194)	0.350 (0.111, 1.104)
2.142 (0.650, 7.063)	Suvorexant 40mg	0.441 (0.131, 1.486)	0.422 (0.127, 1.401)	0.670 (0.040, 11.146)	3.206 (0.150, 68.594)	0.836 (0.227, 3.084)	1.571 (0.481, 5.125)	1.342 (0.373, 4.826)	0.751 (0.433, 1.301)
4.860 (1.003, 23.556)	2.269 (0.673, 7.651)	Lemborexant 5mg	0.956 (0.370, 2.472)	1.521 (0.079, 29.415)	7.274 (0.296, 178.877)	1.898 (0.382, 9.441)	3.564 (0.790, 16.080)	3.046 (0.625, 14.847)	1.703 (0.576, 5.033)
5.081 (1.060, 24.354)	2.372 (0.714, 7.883)	1.046 (0.404, 2.702)	Lemborexant 10mg	1.590 (0.083, 30.569)	7.604 (0.311, 185.991)	1.984 (0.403, 9.763)	3.727 (0.836, 16.617)	3.184 (0.661, 15.350)	1.781 (0.612, 5.177)
3.196 (0.161, 63.320)	1.492 (0.090, 24.821)	0.658 (0.034, 12.719)	0.629 (0.033, 12.095)	Lemborexant 25mg	4.783 (0.081, 284.178)	1.248 (0.062, 25.073)	2.344 (0.123, 44.741)	2.003 (0.101, 39.805)	1.120 (0.071, 17.644)
0.668 (0.027, 16.800)	0.312 (0.015, 6.676)	0.137 (0.006, 3.381)	0.132 (0.005, 3.216)	0.209 (0.004, 12.420)	Daridorexant 5mg	0.261 (0.014, 5.012)	0.490 (0.023, 10.370)	0.419 (0.020, 8.602)	0.234 (0.012, 4.767)
2.561 (0.493, 13.311)	1.196 (0.324, 4.409)	0.527 (0.106, 2.621)	0.504 (0.102, 2.480)	0.801 (0.040, 16.096)	3.833 (0.200, 73.626)	Daridorexant 10mg	1.878 (0.530, 6.650)	1.605 (0.404, 6.376)	0.897 (0.275, 2.930)
1.364 (0.288, 6.446)	0.637 (0.195, 2.077)	0.281 (0.062, 1.266)	0.268 (0.060, 1.196)	0.427 (0.022, 8.143)	2.041 (0.096, 43.185)	0.532 (0.150, 1.885)	Daridorexant 25mg	0.855 (0.225, 3.251)	0.478 (0.168, 1.361)
1.596 (0.313, 8.131)	0.745 (0.207, 2.679)	0.328 (0.067, 1.600)	0.314 (0.065, 1.514)	0.499 (0.025, 9.921)	2.388 (0.116, 49.058)	0.623 (0.157, 2.475)	1.170 (0.308, 4.453)	Daridorexant50mg	0.559 (0.176, 1.776)
2.854 (0.906, 8.991)	1.332 (0.768, 2.310)	0.587 (0.199, 1.735)	0.562 (0.193, 1.633)	0.893 (0.057, 14.065)	4.271 (0.210, 86.947)	1.114 (0.341, 3.638)	2.093 (0.735, 5.961)	1.788 (0.563, 5.679)	Placebo

eTable 44: Detailed certainty of evidence for each outcome in double-blinded parallel-group studies.

Comparison	Number of studies	Within-study bias	Reporting bias	Indirectness	Imprecision	Heterogeneity	Incoherence	Confidence rating	Reason(s) for downgrading
**1. LPS**									
B_Suvorexant_20mg:O_Placebo	4	No concerns	Low risk	No concerns	No concerns	No concerns	No concerns	High	[]
C_Suvorexant_40mg:O_Placebo	4	No concerns	Low risk	No concerns	No concerns	No concerns	Some concerns	Moderate	["Incoherence"]
G_Lemborexant_5mg:O_Placebo	1	No concerns	Low risk	No concerns	No concerns	No concerns	Some concerns	Moderate	["Incoherence"]
H_Lemborexant_10mg:O_Placebo	1	No concerns	Low risk	No concerns	No concerns	No concerns	Some concerns	Moderate	["Incoherence"]
K_Daridorexant_5mg:O_Placebo	1	No concerns	Low risk	No concerns	No concerns	No concerns	No concerns	High	[]
L_Daridorexant_10mg:O_Placebo	3	No concerns	Low risk	No concerns	No concerns	No concerns	No concerns	High	[]
M_Daridorexant_25mg:O_Placebo	5	No concerns	Low risk	No concerns	No concerns	No concerns	Some concerns	Moderate	["Incoherence"]
N_Daridorexant_50mg:O_Placebo	3	No concerns	Low risk	No concerns	No concerns	No concerns	No concerns	High	[]
**2. sTSO**									
B_Suvorexant_20mg:O_Placebo	4	No concerns	Low risk	No concerns	No concerns	No concerns	No concerns	High	[]
C_Suvorexant_40mg:O_Placebo	6	No concerns	Low risk	No concerns	No concerns	No concerns	No concerns	High	[]
G_Lemborexant_5mg:O_Placebo	2	No concerns	Low risk	No concerns	No concerns	No concerns	No concerns	High	[]
H_Lemborexant_10mg:O_Placebo	2	No concerns	Low risk	No concerns	No concerns	No concerns	No concerns	High	[]
K_Daridorexant_5mg:O_Placebo	1	No concerns	Low risk	No concerns	No concerns	Some concerns	No concerns	Moderate	["Heterogeneity"]
L_Daridorexant_10mg:O_Placebo	1	No concerns	Low risk	No concerns	No concerns	No concerns	No concerns	High	[]
M_Daridorexant_25mg:O_Placebo	1	No concerns	Low risk	No concerns	No concerns	No concerns	No concerns	High	[]
N_Daridorexant_50mg:O_Placebo	1	No concerns	Low risk	No concerns	No concerns	No concerns	No concerns	High	[]
**3. WASO**									
B_Suvorexant_20mg:O_Placebo	4	No concerns	Low risk	No concerns	No concerns	No concerns	No concerns	High	[]
C_Suvorexant_40mg:O_Placebo	4	No concerns	Low risk	No concerns	No concerns	No concerns	Some concerns	Moderate	["Incoherence"]
G_Lemborexant_5mg:O_Placebo	1	No concerns	Low risk	No concerns	No concerns	No concerns	Some concerns	Moderate	["Incoherence"]
H_Lemborexant_10mg:O_Placebo	1	No concerns	Low risk	No concerns	No concerns	No concerns	Some concerns	Moderate	["Incoherence"]
K_Daridorexant_5mg:O_Placebo	1	No concerns	Low risk	No concerns	Some concerns	No concerns	No concerns	Moderate	["Imprecision"]
L_Daridorexant_10mg:O_Placebo	3	No concerns	Low risk	No concerns	No concerns	Some concerns	No concerns	Moderate	["Heterogeneity"]

M_Daridorexant_25mg:O_Placebo	5	No concerns	Low risk	No concerns	No concerns	Some concerns	Some concerns	Low	["Heterogeneity","Incoherence"]
N_Daridorexant_50mg:O_Placebo	3	No concerns	Low risk	No concerns	No concerns	No concerns	No concerns	High	[]
**4. sWASO**									
B_Suvorexant_20mg:O_Placebo	4	No concerns	Low risk	No concerns	No concerns	Some concerns	No concerns	Moderate	["Heterogeneity"]
C_Suvorexant_40mg:O_Placebo	6	No concerns	Low risk	No concerns	No concerns	No concerns	No concerns	High	[]
G_Lemborexant_5mg:O_Placebo	2	Some concerns	Low risk	No concerns	No concerns	No concerns	No concerns	Moderate	["Within-study bias"]
H_Lemborexant_10mg:O_Placebo	2	Some concerns	Low risk	No concerns	No concerns	No concerns	No concerns	Moderate	["Within-study bias"]
K_Daridorexant_5mg:O_Placebo	1	No concerns	Low risk	No concerns	No concerns	No concerns	No concerns	High	[]
L_Daridorexant_10mg:O_Placebo	1	No concerns	Low risk	No concerns	No concerns	No concerns	No concerns	High	[]
M_Daridorexant_25mg:O_Placebo	1	No concerns	Low risk	No concerns	No concerns	Some concerns	No concerns	Moderate	["Heterogeneity"]
N_Daridorexant_50mg:O_Placebo	1	No concerns	Low risk	No concerns	No concerns	No concerns	No concerns	High	[]
**5. sTST**									
B_Suvorexant_20mg:O_Placebo	4	No concerns	Low risk	No concerns	No concerns	No concerns	No concerns	High	[]
C_Suvorexant_40mg:O_Placebo	6	No concerns	Low risk	No concerns	No concerns	No concerns	No concerns	High	[]
G_Lemborexant_5mg:O_Placebo	1	Some concerns	Low risk	No concerns	No concerns	No concerns	No concerns	Moderate	["Within-study bias"]
H_Lemborexant_10mg:O_Placebo	1	Some concerns	Low risk	No concerns	No concerns	No concerns	No concerns	Moderate	["Within-study bias"]
K_Daridorexant_5mg:O_Placebo	1	No concerns	Low risk	No concerns	No concerns	No concerns	No concerns	High	[]
L_Daridorexant_10mg:O_Placebo	3	No concerns	Low risk	No concerns	No concerns	No concerns	No concerns	High	[]
M_Daridorexant_25mg:O_Placebo	5	No concerns	Low risk	No concerns	No concerns	No concerns	No concerns	High	[]
N_Daridorexant_50mg:O_Placebo	3	No concerns	Low risk	No concerns	No concerns	No concerns	No concerns	High	[]
**6. ISI**									
B_Suvorexant_20mg:O_Placebo	4	No concerns	Low risk	No concerns	No concerns	Some concerns	No concerns	Moderate	["Heterogeneity"]
C_Suvorexant_40mg:O_Placebo	6	No concerns	Low risk	No concerns	No concerns	Some concerns	No concerns	Moderate	["Heterogeneity"]
G_Lemborexant_5mg:O_Placebo	1	No concerns	Low risk	No concerns	No concerns	No concerns	No concerns	High	[]
H_Lemborexant_10mg:O_Placebo	1	No concerns	Low risk	No concerns	No concerns	No concerns	No concerns	High	[]
K_Daridorexant_5mg:O_Placebo	1	No concerns	Low risk	No concerns	Some concerns	Some concerns	No concerns	Low	["Imprecision","Heterogeneity"]
L_Daridorexant_10mg:O_Placebo	1	No concerns	Low risk	No concerns	Some concerns	Some concerns	No concerns	Low	["Imprecision","Heterogeneity"]
M_Daridorexant_25mg:O_Placebo	1	No concerns	Low risk	No concerns	Some concerns	Some concerns	No concerns	Low	["Imprecision","Heterogeneity"]
N_Daridorexant_50mg:O_Placebo	1	No concerns	Low risk	No concerns	Some concerns	No concerns	No concerns	Moderate	["Imprecision"]

**7. AEs**									
B_Suvorexant_20mg:O_Placebo	2	No concerns	Low risk	No concerns	No concerns	No concerns	No concerns	High	[]
C_Suvorexant_40mg:O_Placebo	3	No concerns	Low risk	No concerns	No concerns	No concerns	No concerns	High	[]
E_Lemborexant_1mg:O_Placebo	1	Some concerns	Low risk	No concerns	Some concerns	Some concerns	No concerns	Very low	["Within-study bias","Imprecision","Heterogeneity"]
F_Lemborexant_2_5mg:O_Placebo	1	Some concerns	Low risk	No concerns	Major concerns	No concerns	No concerns	Very low	["Within-study bias","Imprecision"]
G_Lemborexant_5mg:O_Placebo	3	Some concerns	Low risk	No concerns	No concerns	No concerns	No concerns	Moderate	["Within-study bias"]
H_Lemborexant_10mg:O_Placebo	3	Some concerns	Low risk	No concerns	No concerns	No concerns	No concerns	Moderate	["Within-study bias"]
I_Lemborexant_15mg:O_Placebo	1	Some concerns	Low risk	No concerns	Some concerns	No concerns	No concerns	Low	["Within-study bias","Imprecision"]
J_Lemborexant_25mg:O_Placebo	1	Some concerns	Low risk	No concerns	Some concerns	No concerns	No concerns	Low	["Within-study bias","Imprecision"]
K_Daridorexant_5mg:O_Placebo	1	No concerns	Low risk	No concerns	Major concerns	No concerns	No concerns	Low	["Imprecision"]
L_Daridorexant_10mg:O_Placebo	2	No concerns	Low risk	No concerns	Some concerns	No concerns	No concerns	Moderate	["Imprecision"]
M_Daridorexant_25mg:O_Placebo	3	No concerns	Low risk	No concerns	No concerns	Some concerns	No concerns	Moderate	["Heterogeneity"]
N_Daridorexant_50mg:O_Placebo	2	No concerns	Low risk	No concerns	Some concerns	No concerns	No concerns	Moderate	["Imprecision"]
**8. SAEs**									
B_Suvorexant_20mg:O_Placebo	2	No concerns	Low risk	No concerns	Some concerns	Some concerns	No concerns	Low	["Imprecision","Heterogeneity"]
C_Suvorexant_40mg:O_Placebo	3	No concerns	Low risk	No concerns	Major concerns	No concerns	No concerns	Low	["Imprecision"]
G_Lemborexant_5mg:O_Placebo	2	Some concerns	Low risk	No concerns	Major concerns	No concerns	No concerns	Very low	["Within-study bias","Imprecision"]
H_Lemborexant_10mg:O_Placebo	2	Some concerns	Low risk	No concerns	Major concerns	No concerns	No concerns	Very low	["Within-study bias","Imprecision"]
J_Lemborexant_25mg:O_Placebo	1	Some concerns	Low risk	No concerns	Major concerns	No concerns	No concerns	Very low	["Within-study bias","Imprecision"]
K_Daridorexant_5mg:O_Placebo	1	No concerns	Low risk	No concerns	Major concerns	No concerns	No concerns	Low	["Imprecision"]
L_Daridorexant_10mg:O_Placebo	2	No concerns	Low risk	No concerns	Major concerns	No concerns	No concerns	Low	["Imprecision"]
M_Daridorexant_25mg:O_Placebo	3	No concerns	Low risk	No concerns	Major concerns	No concerns	No concerns	Low	["Imprecision"]
N_Daridorexant_50mg:O_Placebo	2	No concerns	Low risk	No concerns	Major concerns	No concerns	No concerns	Low	["Imprecision"]

eTable 45: Network meta-analysis results of sTSO in studies with low bias.

Suvorexant 10mg	3.927 (-3.287, 11.141)	**8.444 (1.330, 15.558)**	4.700 (-4.388, 13.788)	**15.186 (6.228, 24.145)**	**14.786 (5.883, 23.690)**	-5.692 (-12.757, 1.372)	0.727 (-6.344, 7.798)	-1.461 (-8.528, 5.607)	4.125 (-2.942, 11.191)	-1.914 (-8.900, 5.072)
-3.927 (-11.141, 3.287)	Suvorexant 20mg	**4.517 (1.788, 7.245)**	0.773 (-6.748, 8.294)	**11.259 (5.040, 17.478)**	**10.859 (4.720, 16.998)**	**-9.620 (-12.505, -6.734)**	**-3.200 (-6.103, -0.298)**	**-5.388 (-8.280, -2.496)**	0.197 (-2.693, 3.088)	**-5.841 (-8.528, −3.154)**
**-8.444 (-15.558, -1.330)**	**-4.517 (-7.245, -1.788)**	Suvorexant 40mg	-3.744 (-11.169, 3.682)	**6.742 (0.737, 12.747)**	**6.342 (0.420, 12.264)**	**-14.136 (-16.526, -11.747)**	**-7.717 (-10.127, -5.307)**	**-9.905 (-12.302, -7.507)**	**-4.319 (-6.715, -1.924)**	**-10.358 (-12.503, -8.213)**
-4.700 (-13.788, 4.388)	-0.773 (-8.294, 6.748)	3.744 (-3.682, 11.169)	Suvorexant 80mg	**10.486 (1.278, 19.694)**	**10.086 (0.932, 19.240)**	**-10.392 (-17.770, -3.014)**	-3.973 (-11.357, 3.411)	-6.161 (-13.541, 1.220)	-0.576 (-7.955, 6.804)	-6.614 (-13.916, 0.689)
**-15.186 (-24.145, -6.228)**	**-11.259 (-17.478, -5.040)**	**-6.742 (-12.747, -0.737)**	**-10.486 (-19.694, -1.278)**	Lemborexant 5mg	-0.400 (-6.248, 5.448)	**-20.878 (-26.585, -15.172)**	**-14.459 (-20.174, -8.744)**	**-16.647 (-22.356, -10.937)**	**-11.062 (-16.771, -5.353)**	**-17.100 (-22.709, -11.492)**
**-14.786 (-23.690, -5.883)**	**-10.859 (-16.998, -4.720)**	**-6.342 (-12.264, -0.420)**	**-10.086 (-19.240, -0.932)**	0.400 (-5.448, 6.248)	Lemborexant 10mg	**-20.478 (-26.098, -14.859)**	**-14.059 (-19.687, -8.431)**	**-16.247 (-21.869, -10.624)**	**-10.662 (-16.284, -5.040)**	**-16.700 (-22.220, -11.180)**
5.692 (-1.372, 12.757)	**9.620 (6.734, 12.505)**	**14.136 (11.747, 16.526)**	**10.392 (3.014, 17.770)**	**20.878 (15.172, 26.585)**	**20.478 (14.859, 26.098)**	Daridorexant 5mg	**6.419 (5.340, 7.498)**	**4.232 (3.180, 5.283)**	**9.817 (8.770, 10.864)**	**3.778 (2.726, 4.831)**
-0.727 (-7.798, 6.344)	**3.200 (0.298, 6.103)**	**7.717 (5.307, 10.127)**	3.973 (-3.411, 11.357)	**14.459 (8.744, 20.174)**	**14.059 (8.431, 19.687)**	**-6.419 (-7.498, -5.340)**	Daridorexant 10mg	**-2.188 (-3.284, -1.091)**	**3.397 (2.305, 4.490)**	**-2.641 (-3.738, -1.543)**
1.461 (-5.607, 8.528)	**5.388 (2.496, 8.280)**	**9.905 (7.507, 12.302)**	6.161 (-1.220, 13.541)	**16.647 (10.937, 22.356)**	**16.247 (10.624, 21.869)**	**-4.232 (-5.283, -3.180)**	**2.188 (1.091, 3.284)**	Daridorexant 25mg	**5.585 (4.520, 6.650)**	-0.453 (-1.523, 0.617)
-4.125 (-11.191, 2.942)	-0.197 (-3.088, 2.693)	**4.319 (1.924, 6.715)**	0.576 (-6.804, 7.955)	**11.062 (5.353, 16.771)**	**10.662 (5.040, 16.284)**	**-9.817 (-10.864, -8.770)**	**-3.397 (-4.490, -2.305)**	**-5.585 (-6.650, -4.520)**	Daridorexant 50mg	**-6.038 (-7.104, -4.972)**
1.914 (-5.072, 8.900)	**5.841 (3.154, 8.528)**	**10.358 (8.213, 12.503)**	6.614 (-0.689, 13.916)	**17.100 (11.492, 22.709)**	**16.700 (11.180, 22.220)**	**-3.778 (-4.831, -2.726)**	**2.641 (1.543, 3.738)**	0.453 (-0.617, 1.523)	**6.038 (4.972, 7.104)**	Placebo

eTable 46: Network meta-analysis results of sWASO in studies with low bias.

Suvorexant 20mg	2.829 (-0.017, 5.676)	2.634 (-8.238, 13.505)	**16.134 (4.087, 28.180)**	-2.154 (-5.380, 1.073)	**-7.707 (-10.958, -4.457)**	1.902 (-1.316, 5.121)	**5.784 (2.552, 9.016)**	**-5.867 (-8.723, -3.010)**
-2.829 (-5.676, 0.017)	Suvorexant 40mg	-0.196 (-10.907, 10.514)	**13.304 (1.402, 25.206)**	**-4.983 (-7.618, -2.348)**	**-10.537 (-13.201, -7.873)**	-0.927 (-3.552, 1.698)	**2.954 (0.313, 5.596)**	**-8.696 (-10.862, -6.530)**
-2.634 (-13.505, 8.238)	0.196 (-10.514, 10.907)	Lemborexant 5mg	**13.500 (2.345, 24.655)**	-4.787 (-15.383, 5.809)	-10.341 (-20.944, 0.262)	-0.731 (-11.325, 9.862)	3.151 (-7.447, 13.748)	-8.500 (-18.989, 1.989)
**-16.134 (-28.180, -4.087)**	**-13.304 (-25.206, -1.402)**	**-13.500 (-24.655, -2.345)**	Lemborexant 10mg	**-18.287 (-30.086, -6.488)**	**-23.841 (-35.646, -12.036)**	**-14.231 (-26.028, -2.435)**	-10.350 (-22.150, 1.451)	**-22.000 (-33.703, -10.297)**
2.154 (-1.073, 5.380)	**4.983 (2.348, 7.618)**	4.787 (-5.809, 15.383)	**18.287 (6.488, 30.086)**	Daridorexant 5mg	**-5.554 (-7.104, -4.004)**	**4.056 (2.573, 5.538)**	**7.937 (6.426, 9.449)**	**-3.713 (-5.213, -2.213)**
**7.707 (4.457, 10.958)**	**10.537 (7.873, 13.201)**	10.341 (-0.262, 20.944)	**23.841 (12.036, 35.646)**	**5.554 (4.004, 7.104)**	Daridorexant 10mg	**9.610 (8.077, 11.142)**	**13.491 (11.931, 15.052)**	**1.841 (0.291, 3.391)**
-1.902 (-5.121, 1.316)	0.927 (-1.698, 3.552)	0.731 (-9.862, 11.325)	**14.231 (2.435, 26.028)**	**-4.056 (-5.538, -2.573)**	**-9.610 (-11.142, -8.077)**	Daridorexant 25mg	**3.882 (2.388, 5.376)**	**-7.769 (-9.251, -6.286)**
**-5.784 (-9.016, -2.552)**	**-2.954 (-5.596, -0.313)**	-3.151 (-13.748, 7.447)	10.350 (-1.451, 22.150)	**-7.937 (-9.449, -6.426)**	**-13.491 (-15.052, -11.931)**	**-3.882 (-5.376, -2.388)**	Daridorexant 50mg	**-11.650 (-13.162, -10.139)**
**5.867 (3.010, 8.723)**	**8.696 (6.530, 10.862)**	8.500 (-1.989, 18.989)	**22.000 (10.297, 33.703)**	**3.713 (2.213, 5.213)**	**-1.841 (-3.391, -0.291)**	**7.769 (6.286, 9.251)**	**11.650 (10.139, 13.162)**	Placebo

eTable 47: Network meta-analysis results of sTST in studies with low bias.

Suvorexant 10mg	-8.143 (-21.397, 5.112)	**-15.611 (-28.689, -2.533)**	-13.900 (-30.941, 3.141)	12.726 (-5.252, 30.704)	-3.941 (-17.812, 9.931)	-6.719 (-20.206, 6.768)	**-16.101 (-29.973, -2.230)**	7.453 (-5.417, 20.323)
8.143 (-5.112, 21.397)	Suvorexant 20mg	**-7.469 (-11.746, -3.191)**	-5.758 (-19.457, 7.942)	**20.869 (7.621, 34.116)**	4.202 (-2.482, 10.886)	1.423 (-4.422, 7.268)	**-7.959 (-14.643, -1.274)**	**15.595 (11.364, 19.827)**
**15.611 (2.533, 28.689)**	**7.469 (3.191, 11.746)**	Suvorexant 40mg	1.711 (-11.818, 15.240)	**28.337 (15.368, 41.306)**	**11.671 (5.557, 17.784)**	**8.892 (3.709, 14.075)**	-0.490 (-6.604, 5.624)	**23.064 (19.808, 26.320)**
13.900 (-3.141, 30.941)	5.758 (-7.942, 19.457)	-1.711 (-15.240, 11.818)	Suvorexant 80mg	**26.626 (8.317, 44.935)**	9.960 (-4.338, 24.257)	7.181 (-6.744, 21.105)	-2.201 (-16.498, 12.096)	**21.353 (8.025, 34.681)**
-12.726 (-30.704, 5.252)	**-20.869 (-34.116, -7.621)**	**-28.337 (-41.306, -15.368)**	**-26.626 (-44.935, -8.317)**	Daridorexant 5mg	**-16.667 (-29.502, -3.832)**	**-19.445 (-31.976, -6.915)**	**-28.827 (-41.697, -15.958)**	-5.273 (-17.826, 7.280)
3.941 (-9.931, 17.812)	-4.202 (-10.886, 2.482)	**-11.671 (-17.784, -5.557)**	-9.960 (-24.257, 4.338)	**16.667 (3.832, 29.502)**	Daridorexant 10mg	-2.779 (-7.943, 2.386)	**-12.161 (-18.754, -5.568)**	**11.393 (6.219, 16.568)**
6.719 (-6.768, 20.206)	-1.423 (-7.268, 4.422)	**-8.892 (-14.075, -3.709)**	-7.181 (-21.105, 6.744)	**19.445 (6.915, 31.976)**	2.779 (-2.386, 7.943)	Daridorexant 25mg	**-9.382 (-14.547, -4.216)**	**14.172 (10.140, 18.204)**
**16.101 (2.230, 29.973)**	**7.959 (1.274, 14.643)**	0.490 (-5.624, 6.604)	2.201 (-12.096, 16.498)	**28.827 (15.958, 41.697)**	**12.161 (5.568, 18.754)**	**9.382 (4.216, 14.547)**	Daridorexant 50mg	**23.554 (18.380, 28.729)**
-7.453 (-20.323, 5.417)	**-15.595 (-19.827, -11.364)**	**-23.064 (-26.320, -19.808)**	**-21.353 (-34.681, -8.025)**	5.273 (-7.280, 17.826)	**-11.393 (-16.568, -6.219)**	**-14.172 (-18.204, -10.140)**	**-23.554 (-28.729, -18.380)**	Placebo

eTable 48: Network meta-analysis results of AEs in studies with low bias.

Suvorexant 10mg	0.817 (0.459, 1.455)	0.746 (0.421, 1.322)	**0.492 (0.262, 0.925)**	0.744 (0.390, 1.418)	0.676 (0.356, 1.284)
1.223 (0.687, 2.178)	Suvorexant 20mg	0.913 (0.817, 1.021)	**0.602 (0.405, 0.895)**	0.910 (0.659, 1.256)	0.827 (0.604, 1.134)
1.340 (0.756, 2.374)	1.095 (0.980, 1.224)	Suvorexant 40mg	**0.659 (0.447, 0.973)**	0.996 (0.730, 1.360)	0.906 (0.669, 1.227)
**2.033 (1.081, 3.823)**	**1.661 (1.118, 2.470)**	**1.517 (1.028, 2.239)**	Suvorexant 80mg	1.512 (0.925, 2.470)	1.374 (0.845, 2.234)
1.345 (0.705, 2.565)	1.099 (0.796, 1.518)	1.004 (0.735, 1.371)	0.662 (0.405, 1.081)	Lemborexant 5mg	0.909 (0.698, 1.185)
1.479 (0.779, 2.810)	1.209 (0.882, 1.656)	1.104 (0.815, 1.495)	0.728 (0.448, 1.183)	1.100 (0.844, 1.433)	Lemborexant 10mg
1.402 (0.725, 2.710)	1.146 (0.809, 1.624)	1.047 (0.746, 1.468)	0.690 (0.415, 1.147)	1.043 (0.666, 1.632)	0.948 (0.609, 1.475)
1.424 (0.784, 2.586)	1.164 (0.945, 1.434)	1.063 (0.879, 1.286)	0.701 (0.458, 1.071)	1.059 (0.746, 1.502)	0.963 (0.684, 1.356)
1.430 (0.794, 2.573)	1.168 (0.975, 1.400)	1.067 (0.910, 1.252)	0.703 (0.466, 1.062)	1.063 (0.761, 1.485)	0.967 (0.697, 1.340)
1.432 (0.789, 2.600)	1.171 (0.951, 1.440)	1.069 (0.885, 1.292)	0.705 (0.461, 1.077)	1.065 (0.751, 1.510)	0.968 (0.688, 1.363)
1.226 (0.693, 2.168)	1.002 (0.895, 1.121)	**0.915 (0.850, 0.985)**	**0.603 (0.410, 0.888)**	0.912 (0.674, 1.234)	0.829 (0.617, 1.112)

0.713 (0.369, 1.378)	0.702 (0.387, 1.275)	0.699 (0.389, 1.259)	0.698 (0.385, 1.268)	0.816 (0.461, 1.443)
0.873 (0.616, 1.237)	0.859 (0.698, 1.058)	0.856 (0.714, 1.025)	0.854 (0.694, 1.051)	0.998 (0.892, 1.117)
0.955 (0.681, 1.340)	0.941 (0.778, 1.138)	0.937 (0.799, 1.099)	0.935 (0.774, 1.130)	**1.093 (1.015, 1.176)**
1.450 (0.872, 2.411)	1.427 (0.933, 2.183)	1.422 (0.942, 2.147)	1.419 (0.929, 2.169)	**1.658 (1.126, 2.441)**
0.959 (0.613, 1.501)	0.944 (0.666, 1.340)	0.941 (0.674, 1.314)	0.939 (0.662, 1.331)	1.097 (0.811, 1.485)
1.055 (0.678, 1.641)	1.039 (0.737, 1.463)	1.035 (0.746, 1.434)	1.033 (0.734, 1.454)	1.207 (0.899, 1.619)
Daridorexant 5mg	0.985 (0.704, 1.377)	0.981 (0.708, 1.359)	0.979 (0.701, 1.367)	1.144 (0.822, 1.591)
1.016 (0.726, 1.420)	Daridorexant 10mg	0.996 (0.843, 1.177)	0.994 (0.806, 1.227)	1.162 (0.975, 1.385)
1.020 (0.736, 1.412)	1.004 (0.849, 1.186)	Daridorexant 25mg	0.998 (0.843, 1.181)	**1.166 (1.012, 1.344)**

1.021 (0.731, 1.426)	1.006 (0.815, 1.241)	1.002 (0.847, 1.186)	Daridorexant 50mg	1.168 (0.981, 1.391)
0.874 (0.628, 1.216)	0.861 (0.722, 1.026)	**0.857 (0.744, 0.988)**	0.856 (0.719, 1.019)	Placebo

eTable 49: Network meta-analysis results of SAEs in studies with low bias.

Suvorexant 10mg	2.550 (0.096, 67.505)	1.374 (0.054, 34.761)	0.984 (0.020, 49.274)	0.269 (0.003, 22.712)	1.357 (0.008, 218.263)
0.392 (0.015, 10.383)	Suvorexant 20mg	0.539 (0.173, 1.678)	0.386 (0.015, 10.216)	0.106 (0.004, 2.680)	0.532 (0.009, 31.299)
0.728 (0.029, 18.408)	1.855 (0.596, 5.778)	Suvorexant 40mg	0.716 (0.028, 18.111)	0.196 (0.009, 4.318)	0.987 (0.019, 51.972)
1.016 (0.020, 50.892)	2.591 (0.098, 68.587)	1.397 (0.055, 35.322)	Suvorexant 80mg	0.274 (0.003, 23.078)	1.379 (0.009, 221.783)
3.713 (0.044, 313.093)	9.467 (0.373, 240.159)	5.102 (0.232, 112.415)	3.654 (0.043, 308.062)	Lemborexant 5mg	5.037 (0.240, 105.710)
0.737 (0.005, 118.581)	1.879 (0.032, 110.542)	1.013 (0.019, 53.319)	0.725 (0.005, 116.676)	0.199 (0.009, 4.166)	Lemborexant 10mg
0.221 (0.003, 18.320)	0.565 (0.023, 13.952)	0.304 (0.014, 6.522)	0.218 (0.003, 18.024)	0.060 (0.001, 4.325)	0.300 (0.002, 42.398)
0.848 (0.027, 26.375)	2.163 (0.431, 10.849)	1.166 (0.316, 4.308)	0.835 (0.027, 25.948)	0.228 (0.009, 5.987)	1.151 (0.019, 69.477)
0.451 (0.015, 13.421)	1.151 (0.253, 5.236)	0.620 (0.190, 2.028)	0.444 (0.015, 13.204)	0.122 (0.005, 3.040)	0.613 (0.011, 35.613)
0.529 (0.017, 16.284)	1.348 (0.274, 6.627)	0.727 (0.202, 2.619)	0.520 (0.017, 16.023)	0.142 (0.005, 3.695)	0.717 (0.012, 42.966)
0.945 (0.038, 23.781)	2.408 (0.807, 7.186)	1.298 (0.749, 2.250)	0.930 (0.037, 23.397)	0.254 (0.012, 5.335)	1.282 (0.025, 64.929)

4.515 (0.055, 373.531)	1.179 (0.038, 36.642)	2.215 (0.075, 65.851)	1.891 (0.061, 58.236)	1.059 (0.042, 26.650)
1.771 (0.072, 43.759)	0.462 (0.092, 2.319)	0.869 (0.191, 3.952)	0.742 (0.151, 3.646)	0.415 (0.139, 1.239)
3.286 (0.153, 70.422)	0.858 (0.232, 3.169)	1.612 (0.493, 5.269)	1.376 (0.382, 4.959)	0.770 (0.444, 1.335)
4.589 (0.055, 379.555)	1.198 (0.039, 37.233)	2.251 (0.076, 66.913)	1.922 (0.062, 59.175)	1.076 (0.043, 27.077)
16.767 (0.231, 1215.852)	4.377 (0.167, 114.686)	8.224 (0.329, 205.614)	7.022 (0.271, 182.181)	3.931 (0.187, 82.426)
3.328 (0.024, 469.656)	0.869 (0.014, 52.442)	1.633 (0.028, 94.926)	1.394 (0.023, 83.471)	0.780 (0.015, 39.532)
Daridorexant 5mg	0.261 (0.014, 5.022)	0.491 (0.023, 10.397)	0.419 (0.020, 8.616)	0.234 (0.011, 4.780)
3.831 (0.199, 73.700)	Daridorexant 10mg	1.879 (0.530, 6.668)	1.604 (0.403, 6.389)	0.898 (0.274, 2.939)
2.038 (0.096, 43.207)	0.532 (0.150, 1.888)	Daridorexant 25mg	0.854 (0.224, 3.255)	0.478 (0.167, 1.364)

2.388 (0.116, 49.127)	0.623 (0.157, 2.482)	1.171 (0.307, 4.466)	Daridorexant 50mg	0.560 (0.176, 1.782)
4.266 (0.209, 86.982)	1.113 (0.340, 3.644)	2.093 (0.733, 5.973)	1.786 (0.561, 5.687)	Placebo

eTable 50: Network meta-analysis results of LPS without 2020 Zammit et al.

Suvorexant 10mg	8.030 (-4.076, 20.136)	8.584 (-3.469, 20.637)	7.200 (-8.075, 22.475)	11.052 (-3.298, 25.403)	13.052 (-1.269, 27.374)
-8.030 (-20.136, 4.076)	Suvorexant 20mg	0.554 (-3.571, 4.679)	-0.830 (-13.167, 11.508)	3.023 (-6.059, 12.105)	5.023 (-4.014, 14.059)
-8.584 (-20.637, 3.469)	-0.554 (-4.679, 3.571)	Suvorexant 40mg	-1.384 (-13.669, 10.901)	2.469 (-6.491, 11.428)	4.469 (-4.444, 13.381)
-7.200 (-22.475, 8.075)	0.830 (-11.508, 13.167)	1.384 (-10.901, 13.669)	Suvorexant 80mg	3.852 (-10.694, 18.399)	5.852 (-8.665, 20.370)
-11.052 (-25.403, 3.298)	-3.023 (-12.105, 6.059)	-2.469 (-11.428, 6.491)	-3.852 (-18.399, 10.694)	Lemborexant 5mg	2.000 (-5.887, 9.887)
-13.052 (-27.374, 1.269)	-5.023 (-14.059, 4.014)	-4.469 (-13.381, 4.444)	-5.852 (-20.370, 8.665)	-2.000 (-9.887, 5.887)	Lemborexant 10mg
9.447 (-3.404, 22.298)	**17.477 (11.019, 23.934)**	**18.031 (11.747, 24.314)**	**16.647 (3.578, 29.717)**	**20.500 (10.936, 30.063)**	**22.500 (12.980, 32.019)**
-6.507 (-18.947, 5.934)	1.523 (-4.072, 7.119)	2.077 (-3.317, 7.471)	0.693 (-11.973, 13.359)	4.546 (-4.458, 13.550)	6.546 (-2.412, 15.503)
-7.796 (-20.053, 4.460)	0.233 (-4.941, 5.407)	0.788 (-4.168, 5.743)	-0.596 (-13.082, 11.889)	3.256 (-5.492, 12.004)	5.256 (-3.444, 13.957)
-8.439 (-20.821, 3.943)	-0.409 (-5.874, 5.055)	0.145 (-5.113, 5.402)	-1.239 (-13.848, 11.370)	2.613 (-6.309, 11.536)	4.613 (-4.263, 13.490)
0.548 (-11.277, 12.372)	**8.577 (4.532, 12.623)**	**9.131 (5.370, 12.893)**	7.748 (-4.314, 19.809)	**11.600 (3.469, 19.732)**	**13.600 (5.520, 21.680)**

-9.447 (-22.298, 3.404)	6.507 (-5.934, 18.947)	7.796 (-4.460, 20.053)	8.439 (-3.943, 20.821)	-0.548 (-12.372, 11.277)
**-17.477 (-23.934, -11.019)**	-1.523 (-7.119, 4.072)	-0.233 (-5.407, 4.941)	0.409 (-5.055, 5.874)	**-8.577 (-12.623, -4.532)**
**-18.031 (-24.314, -11.747)**	-2.077 (-7.471, 3.317)	-0.788 (-5.743, 4.168)	-0.145 (-5.402, 5.113)	**-9.131 (-12.893, -5.370)**
**-16.647 (-29.717, -3.578)**	-0.693 (-13.359, 11.973)	0.596 (-11.889, 13.082)	1.239 (-11.370, 13.848)	-7.748 (-19.809, 4.314)
**-20.500 (-30.063, -10.936)**	-4.546 (-13.550, 4.458)	-3.256 (-12.004, 5.492)	-2.613 (-11.536, 6.309)	**-11.600 (-19.732, -3.469)**
**-22.500 (-32.019, -12.980)**	-6.546 (-15.503, 2.412)	-5.256 (-13.957, 3.444)	-4.613 (-13.490, 4.263)	**-13.600 (-21.680, -5.520)**
Daridorexant 5mg	**15.954 (10.665, 21.243)**	**17.243 (12.210, 22.277)**	**17.886 (12.670, 23.102)**	**8.899 (3.866, 13.933)**
**-15.954 (-21.243, -10.665)**	Daridorexant 10mg	1.290 (-2.578, 5.157)	1.932 (-2.470, 6.335)	**-7.054 (-10.920, -3.188)**
**-17.243 (-22.277, -12.210)**	-1.290 (-5.157, 2.578)	Daridorexant 25mg	0.643 (-3.029, 4.315)	**-8.344 (-11.570, -5.118)**
**-17.886 (-23.102, -12.670)**	-1.932 (-6.335, 2.470)	-0.643 (-4.315, 3.029)	Daridorexant 50mg	**-8.987 (-12.660, -5.313)**
**-8.899 (-13.933, -3.866)**	**7.054 (3.188, 10.920)**	**8.344 (5.118, 11.570)**	**8.987 (5.313, 12.660)**	Placebo

eTable 51: Network meta-analysis results of sTSO without 2020 Zammit et al.

Suvorexant 10mg	3.927 (-3.287, 11.141)	**8.444 (1.330, 15.558)**	4.700 (-4.388, 13.788)	**14.399 (5.644, 23.155)**	**14.637 (5.841, 23.433)**	-5.714 (-12.779, 1.351)	0.686 (-6.386, 7.758)	-1.514 (-8.582, 5.554)	4.086 (-2.981, 11.153)	-1.914 (-8.900, 5.072)
-3.927 (-11.141, 3.287)	Suvorexant 20mg	**4.517 (1.788, 7.245)**	0.773 (-6.748, 8.294)	**10.472 (4.550, 16.394)**	**10.710 (4.728, 16.691)**	**-9.641 (-12.528, -6.754)**	**-3.241 (-6.145, -0.337)**	**-5.441 (-8.335, -2.548)**	0.159 (-2.733, 3.051)	**-5.841 (-8.528, -3.154)**
**-8.444 (-15.558, -1.330)**	**-4.517 (-7.245, -1.788)**	Suvorexant 40mg	-3.744 (-11.169, 3.682)	**5.955 (0.258, 11.652)**	**6.193 (0.434, 11.952)**	**-14.158 (-16.549, -11.767)**	**-7.758 (-10.169, -5.346)**	**-9.958 (-12.357, -7.559)**	**-4.358 (-6.755, -1.961)**	**-10.358 (-12.503, -8.213)**
-4.700 (-13.788, 4.388)	-0.773 (-8.294, 6.748)	3.744 (-3.682, 11.169)	Suvorexant 80mg	**9.699 (0.689, 18.709)**	**9.937 (0.888, 18.986)**	**-10.414 (-17.792, -3.035)**	-4.014 (-11.399, 3.371)	-6.214 (-13.595, 1.167)	-0.614 (-7.994, 6.766)	-6.614 (-13.916, 0.689)
**-14.399 (-23.155, -5.644)**	**-10.472 (-16.394, -4.550)**	**-5.955 (-11.652, -0.258)**	**-9.699 (-18.709, -0.689)**	Lemborexant 5mg	0.238 (-5.381, 5.857)	**-20.113 (-25.495, -14.731)**	**-13.713 (-19.105, -8.322)**	**-15.913 (-21.299, -10.527)**	**-10.313 (-15.698, -4.928)**	**-16.313 (-21.591, -11.035)**
**-14.637 (-23.433, -5.841)**	**-10.710 (-16.691, -4.728)**	**-6.193 (-11.952, -0.434)**	**-9.937 (-18.986, -0.888)**	-0.238 (-5.857, 5.381)	Lemborexant 10mg	**-20.351 (-25.798, -14.903)**	**-13.951 (-19.407, -8.495)**	**-16.151 (-21.602, -10.700)**	**-10.551 (-16.001, -5.101)**	**-16.551 (-21.895, -11.207)**
5.714 (-1.351, 12.779)	**9.641 (6.754, 12.528)**	**14.158 (11.767, 16.549)**	**10.414 (3.035, 17.792)**	**20.113 (14.731, 25.495)**	**20.351 (14.903, 25.798)**	Daridorexant 5mg	**6.400 (5.316, 7.484)**	**4.200 (3.144, 5.256)**	**9.800 (8.749, 10.851)**	**3.800 (2.744, 4.856)**
-0.686 (-7.758, 6.386)	**3.241 (0.337, 6.145)**	**7.758 (5.346, 10.169)**	4.014 (-3.371, 11.399)	**13.713 (8.322, 19.105)**	**13.951 (8.495, 19.407)**	**-6.400 (-7.484, -5.316)**	Daridorexant 10mg	**-2.200 (-3.301, -1.099)**	**3.400 (2.303, 4.497)**	**-2.600 (-3.701, -1.499)**
1.514 (-5.554, 8.582)	**5.441 (2.548, 8.335)**	**9.958 (7.559, 12.357)**	6.214 (-1.167, 13.595)	**15.913 (10.527, 21.299)**	**16.151 (10.700, 21.602)**	**-4.200 (-5.256, -3.144)**	**2.200 (1.099, 3.301)**	Daridorexant 25mg	**5.600 (4.531, 6.669)**	-0.400 (-1.474, 0.674)
-4.086 (-11.153, 2.981)	-0.159 (-3.051, 2.733)	**4.358 (1.961, 6.755)**	0.614 (-6.766, 7.994)	**10.313 (4.928, 15.698)**	**10.551 (5.101, 16.001)**	**-9.800 (-10.851, -8.749)**	**-3.400 (-4.497, -2.303)**	**-5.600 (-6.669, -4.531)**	Daridorexant 50mg	**-6.000 (-7.069, -4.931)**
1.914 (-5.072, 8.900)	**5.841 (3.154, 8.528)**	**10.358 (8.213, 12.503)**	6.614 (-0.689, 13.916)	**16.313 (11.035, 21.591)**	**16.551 (11.207, 21.895)**	**-3.800 (-4.856, -2.744)**	**2.600 (1.499, 3.701)**	0.400 (-0.674, 1.474)	**6.000 (4.931, 7.069)**	Placebo

eTable 52: Network meta-analysis results of sTST without 2020 Zammit et al.

Suvorexant 10mg	-8.143 (-21.397, 5.112)	**-15.611 (-28.689, -2.533)**	-13.900 (-30.941, 3.141)	-11.097 (-23.988, 1.793)	**-15.227 (-28.118, -2.336)**
8.143 (-5.112, 21.397)	Suvorexant 20mg	**-7.469 (-11.746, -3.191)**	-5.758 (-19.457, 7.942)	-2.955 (-7.247, 1.338)	**-7.085 (-11.378, -2.791)**
**15.611 (2.533, 28.689)**	**7.469 (3.191, 11.746)**	Suvorexant 40mg	1.711 (-11.818, 15.240)	**4.514 (1.179, 7.849)**	0.384 (-2.953, 3.721)
13.900 (-3.141, 30.941)	5.758 (-7.942, 19.457)	-1.711 (-15.240, 11.818)	Suvorexant 80mg	2.803 (-10.545, 16.151)	-1.327 (-14.675, 12.021)
11.097 (-1.793, 23.988)	2.955 (-1.338, 7.247)	**-4.514 (-7.849, -1.179)**	-2.803 (-16.151, 10.545)	Lemborexant 5mg	**-4.130 (-4.865, -3.395)**
**15.227 (2.336, 28.118)**	**7.085 (2.791, 11.378)**	-0.384 (-3.721, 2.953)	1.327 (-12.021, 14.675)	**4.130 (3.395, 4.865)**	Lemborexant 10mg
-8.211 (-28.710, 12.288)	-16.353 (-32.860, 0.153)	**-23.822 (-40.106, -7.538)**	**-22.111 (-42.900, -1.321)**	**-19.308 (-35.279, -3.337)**	**-23.438 (-39.410, -7.466)**
4.047 (-9.873, 17.967)	-4.095 (-10.879, 2.689)	**-11.564 (-17.787, -5.341)**	-9.853 (-24.197, 4.492)	**-7.050 (-12.402, -1.698)**	**-11.180 (-16.533, -5.827)**
6.449 (-7.056, 19.954)	-1.693 (-7.579, 4.192)	**-9.162 (-14.391, -3.933)**	-7.451 (-21.393, 6.491)	**-4.648 (-8.802, -0.494)**	**-8.778 (-12.934, -4.622)**
**15.497 (1.579, 29.416)**	**7.355 (0.574, 14.136)**	-0.114 (-6.333, 6.106)	1.597 (-12.745, 15.940)	4.400 (-0.947, 9.748)	0.270 (-5.078, 5.619)
-7.453 (-20.323, 5.417)	**-15.595 (-19.827, -11.364)**	**-23.064 (-26.320, -19.808)**	**-21.353 (-34.681, -8.025)**	**-18.550 (-19.271, -17.829)**	**-22.680 (-23.409, -21.951)**

8.211 (-12.288, 28.710)	-4.047 (-17.967, 9.873)	-6.449 (-19.954, 7.056)	**-15.497 (-29.416, -1.579)**	7.453 (-5.417, 20.323)
16.353 (-0.153, 32.860)	4.095 (-2.689, 10.879)	1.693 (-4.192, 7.579)	**-7.355 (-14.136, -0.574)**	**15.595 (11.364, 19.827)**
**23.822 (7.538, 40.106)**	**11.564 (5.341, 17.787)**	**9.162 (3.933, 14.391)**	0.114 (-6.106, 6.333)	**23.064 (19.808, 26.320)**
**22.111 (1.321, 42.900)**	9.853 (-4.492, 24.197)	7.451 (-6.491, 21.393)	-1.597 (-15.940, 12.745)	**21.353 (8.025, 34.681)**
**19.308 (3.337, 35.279)**	**7.050 (1.698, 12.402)**	**4.648 (0.494, 8.802)**	-4.400 (-9.748, 0.947)	**18.550 (17.829, 19.271)**
**23.438 (7.466, 39.410)**	**11.180 (5.827, 16.533)**	**8.778 (4.622, 12.934)**	-0.270 (-5.619, 5.078)	**22.680 (21.951, 23.409)**
Daridorexant 5mg	-12.258 (-28.471, 3.955)	-14.660 (-30.622, 1.302)	**-23.708 (-39.956, -7.460)**	-0.758 (-16.713, 15.197)
12.258 (-3.955, 28.471)	Daridorexant 10mg	-2.402 (-7.707, 2.904)	**-11.450 (-18.277, -4.623)**	**11.500 (6.197, 16.803)**
14.660 (-1.302, 30.622)	2.402 (-2.904, 7.707)	Daridorexant 25mg	**-9.048 (-14.349, -3.747)**	**13.902 (9.811, 17.993)**
**23.708 (7.460, 39.956)**	**11.450 (4.623, 18.277)**	**9.048 (3.747, 14.349)**	Daridorexant 50mg	**22.950 (17.652, 28.249)**
0.758 (-15.197, 16.713)	**-11.500 (-16.803, -6.197)**	**-13.902 (-17.993, -9.811)**	**-22.950 (-28.249, -17.652)**	Placebo

eTable 53: Network meta-analysis results of WASO without 2020 Zammit et al.

Suvorexant 10mg	5.355 (-10.524, 21.233)	8.049 (-7.762, 23.860)	7.500 (-12.516, 27.516)	5.681 (-13.219, 24.582)	8.181 (-10.722, 27.084)
-5.355 (-21.233, 10.524)	Suvorexant 20mg	2.694 (-2.878, 8.265)	2.145 (-14.062, 18.352)	0.326 (-11.809, 12.462)	2.826 (-9.312, 14.965)
-8.049 (-23.860, 7.762)	-2.694 (-8.265, 2.878)	Suvorexant 40mg	-0.549 (-16.690, 15.592)	-2.367 (-14.340, 9.605)	0.133 (-11.844, 12.109)
-7.500 (-27.516, 12.516)	-2.145 (-18.352, 14.062)	0.549 (-15.592, 16.690)	Suvorexant 80mg	-1.819 (-20.996, 17.359)	0.681 (-18.499, 19.861)
-5.681 (-24.582, 13.219)	-0.326 (-12.462, 11.809)	2.367 (-9.605, 14.340)	1.819 (-17.359, 20.996)	Lemborexant 5mg	2.500 (-7.864, 12.864)
-8.181 (-27.084, 10.722)	-2.826 (-14.965, 9.312)	-0.133 (-12.109, 11.844)	-0.681 (-19.861, 18.499)	-2.500 (-12.864, 7.864)	Lemborexant 10mg
14.578 (-2.384, 31.540)	**19.933 (11.116, 28.750)**	**22.627 (14.035, 31.219)**	**22.078 (4.808, 39.348)**	**20.259 (7.401, 33.118)**	**22.759 (9.897, 35.621)**
13.200 (-3.084, 29.483)	**18.555 (11.126, 25.983)**	**21.248 (14.088, 28.409)**	**20.700 (4.095, 37.304)**	**18.881 (6.931, 30.830)**	**21.381 (9.428, 33.334)**
9.833 (-6.216, 25.881)	**15.188 (8.290, 22.085)**	**17.881 (11.274, 24.489)**	**17.333 (0.959, 33.706)**	**15.514 (3.887, 27.140)**	**18.014 (6.384, 29.644)**
2.177 (-14.069, 18.423)	**7.532 (0.187, 14.877)**	**10.226 (3.152, 17.299)**	9.677 (-6.890, 26.244)	7.859 (-4.039, 19.756)	10.358 (-1.543, 22.260)
**19.619 (4.133, 35.104)**	**24.974 (19.512, 30.435)**	**27.667 (22.577, 32.758)**	**27.119 (11.296, 42.941)**	**25.300 (14.463, 36.137)**	**27.800 (16.959, 38.641)**

-14.578 (-31.540, 2.384)	-13.200 (-29.483, 3.084)	-9.833 (-25.881, 6.216)	-2.177 (-18.423, 14.069)	**-19.619 (-35.104, -4.133)**
**-19.933 (-28.750, -11.116)**	**-18.555 (-25.983, -11.126)**	**-15.188 (-22.085, -8.290)**	**-7.532 (-14.877, -0.187)**	**-24.974 (-30.435, -19.512)**
**-22.627 (-31.219, -14.035)**	**-21.248 (-28.409, -14.088)**	**-17.881 (-24.489, -11.274)**	**-10.226 (-17.299, -3.152)**	**-27.667 (-32.758, -22.577)**
**-22.078 (-39.348, -4.808)**	**-20.700 (-37.304, -4.095)**	**-17.333 (-33.706, -0.959)**	-9.677 (-26.244, 6.890)	**-27.119 (-42.941, -11.296)**
**-20.259 (-33.118, -7.401)**	**-18.881 (-30.830, -6.931)**	**-15.514 (-27.140, -3.887)**	-7.859 (-19.756, 4.039)	**-25.300 (-36.137, -14.463)**
**-22.759 (-35.621, -9.897)**	**-21.381 (-33.334, -9.428)**	**-18.014 (-29.644, -6.384)**	-10.358 (-22.260, 1.543)	**-27.800 (-38.641, -16.959)**
Daridorexant 5mg	1.379 (-5.861, 8.618)	4.745 (-2.176, 11.667)	**12.401 (5.207, 19.595)**	-5.041 (-11.962, 1.881)
-1.379 (-8.618, 5.861)	Daridorexant 10mg	3.367 (-1.669, 8.402)	**11.022 (5.156, 16.889)**	**-6.419 (-11.455, -1.384)**
-4.745 (-11.667, 2.176)	-3.367 (-8.402, 1.669)	Daridorexant 25mg	**7.656 (2.744, 12.567)**	**-9.786 (-13.998, -5.574)**
**-12.401 (-19.595, -5.207)**	**-11.022 (-16.889, -5.156)**	**-7.656 (-12.567, -2.744)**	Daridorexant 50mg	**-17.442 (-22.353, -12.530)**
5.041 (-1.881, 11.962)	**6.419 (1.384, 11.455)**	**9.786 (5.574, 13.998)**	**17.442 (12.530, 22.353)**	Placebo

eTable 54: Network meta-analysis results of sWASO without 2020 Zammit et al.

Suvorexant 20mg	2.829 (-0.017, 5.676)	**11.575 (8.662, 14.487)**	**6.823 (3.911, 9.736)**	-2.167 (-5.394, 1.061)
-2.829 (-5.676, 0.017)	Suvorexant 40mg	**8.745 (6.506, 10.984)**	**3.994 (1.754, 6.233)**	**-4.996 (-7.633, -2.360)**
**-11.575 (-14.487, -8.662)**	**-8.745 (-10.984, -6.506)**	Lemborexant 5mg	**-4.751 (-5.324, -4.178)**	**-13.741 (-15.347, -12.135)**
**-6.823 (-9.736, -3.911)**	**-3.994 (-6.233, -1.754)**	**4.751 (4.178, 5.324)**	Lemborexant 10mg	**-8.990 (-10.597, -7.383)**
2.167 (-1.061, 5.394)	**4.996 (2.360, 7.633)**	**13.741 (12.135, 15.347)**	**8.990 (7.383, 10.597)**	Daridorexant 5mg
**7.766 (4.515, 11.018)**	**10.596 (7.931, 13.261)**	**19.341 (17.689, 20.994)**	**14.590 (12.936, 16.244)**	**5.600 (4.047, 7.153)**
-1.833 (-5.053, 1.386)	0.996 (-1.630, 3.622)	**9.741 (8.152, 11.330)**	**4.990 (3.400, 6.580)**	**-4.000 (-5.485, -2.515)**
**-5.734 (-8.967, -2.500)**	**-2.904 (-5.547, -0.261)**	**5.841 (4.224, 7.458)**	1.090 (-0.528, 2.708)	**-7.900 (-9.415, -6.386)**
**5.867 (3.010, 8.723)**	**8.696 (6.530, 10.862)**	**17.441 (16.876, 18.006)**	**12.690 (12.122, 13.258)**	**3.700 (2.197, 5.203)**

**-7.766 (-11.018, -4.515)**	1.833 (-1.386, 5.053)	**5.734 (2.500, 8.967)**	**-5.867 (-8.723, -3.010)**
**-10.596 (-13.261, -7.931)**	-0.996 (-3.622, 1.630)	**2.904 (0.261, 5.547)**	**-8.696 (-10.862, -6.530)**
**-19.341 (-20.994, -17.689)**	**-9.741 (-11.330, -8.152)**	**-5.841 (-7.458, -4.224)**	**-17.441 (-18.006, -16.876)**
**-14.590 (-16.244, -12.936)**	**-4.990 (-6.580, -3.400)**	-1.090 (-2.708, 0.528)	**-12.690 (-13.258, -12.122)**
**-5.600 (-7.153, -4.047)**	**4.000 (2.515, 5.485)**	**7.900 (6.386, 9.415)**	**-3.700 (-5.203, -2.197)**
Daridorexant 10mg	**9.600 (8.064, 11.136)**	**13.500 (11.936, 15.064)**	**1.900 (0.347, 3.453)**
**-9.600 (-11.136, -8.064)**	Daridorexant 25mg	**3.900 (2.403, 5.397)**	**-7.700 (-9.185, -6.215)**
**-13.500 (-15.064, -11.936)**	**-3.900 (-5.397, -2.403)**	Daridorexant 50mg	**-11.600 (-13.114, -10.085)**
**-1.900 (-3.453, -0.347)**	**7.700 (6.215, 9.185)**	**11.600 (10.085, 13.114)**	Placebo

eTable 55: Network meta-analysis results of AEs without 2020 Zammit et al.

Suvorexant 10mg	0.817 (0.459, 1.455)	0.746 (0.421, 1.322)	**0.492 (0.262, 0.925)**	1.112 (0.514, 2.404)	0.938 (0.440, 1.999)	0.819 (0.458, 1.465)	0.803 (0.449, 1.435)
1.223 (0.687, 2.178)	Suvorexant 20mg	0.913 (0.817, 1.021)	**0.602 (0.405, 0.895)**	1.361 (0.800, 2.314)	1.148 (0.690, 1.911)	1.003 (0.856, 1.174)	0.982 (0.839, 1.150)
1.340 (0.756, 2.374)	1.095 (0.980, 1.224)	Suvorexant 40mg	**0.659 (0.447, 0.973)**	1.490 (0.882, 2.516)	1.257 (0.761, 2.078)	1.098 (0.961, 1.254)	1.076 (0.942, 1.229)
**2.033 (1.081, 3.823)**	**1.661 (1.118, 2.470)**	**1.517 (1.028, 2.239)**	Suvorexant 80mg	**2.261 (1.184, 4.318)**	**1.907 (1.016, 3.580)**	**1.666 (1.114, 2.490)**	**1.632 (1.091, 2.440)**
0.899 (0.416, 1.944)	0.735 (0.432, 1.250)	0.671 (0.397, 1.134)	**0.442 (0.232, 0.845)**	Lemborexant 1mg	0.844 (0.436, 1.633)	0.737 (0.438, 1.239)	0.722 (0.430, 1.211)
1.066 (0.500, 2.271)	0.871 (0.523, 1.450)	0.795 (0.481, 1.315)	**0.524 (0.279, 0.984)**	**1.185 (0.612, 2.294)**	Lemborexant 2.5mg	0.873 (0.531, 1.436)	0.856 (0.521, 1.404)
1.220 (0.683, 2.182)	0.998 (0.852, 1.168)	0.911 (0.798, 1.040)	**0.600 (0.402, 0.898)**	1.357 (0.807, 2.282)	1.145 (0.696, 1.884)	Lemborexant 5mg	0.980 (0.877, 1.095)
1.246 (0.697, 2.227)	1.018 (0.870, 1.192)	0.930 (0.814, 1.062)	**0.613 (0.410, 0.916)**	1.385 (0.826, 2.324)	1.169 (0.712, 1.918)	1.021 (0.913, 1.141)	Lemborexant 10mg
1.448 (0.757, 2.770)	1.184 (0.852, 1.644)	1.081 (0.787, 1.485)	0.712 (0.434, 1.169)	1.610 (0.945, 2.745)	1.359 (0.814, 2.268)	1.187 (0.870, 1.618)	1.163 (0.856, 1.579)
1.570 (0.823, 2.992)	1.283 (0.929, 1.771)	1.172 (0.858, 1.599)	0.772 (0.473, 1.261)	**1.745 (1.028, 2.964)**	1.473 (0.886, 2.448)	1.286 (0.950, 1.742)	1.260 (0.934, 1.700)
1.354 (0.675, 2.716)	1.107 (0.732, 1.675)	1.011 (0.674, 1.516)	0.666 (0.382, 1.161)	1.506 (0.783, 2.898)	1.271 (0.672, 2.403)	1.110 (0.734, 1.678)	1.087 (0.719, 1.645)
1.422 (0.782, 2.586)	1.162 (0.939, 1.438)	1.061 (0.873, 1.290)	0.699 (0.456, 1.072)	1.581 (0.913, 2.739)	1.334 (0.786, 2.264)	1.165 (0.942, 1.440)	1.141 (0.923, 1.411)
1.428 (0.793, 2.571)	1.167 (0.973, 1.401)	1.066 (0.907, 1.253)	0.703 (0.465, 1.061)	1.588 (0.927, 2.721)	1.340 (0.799, 2.248)	1.170 (0.976, 1.402)	1.146 (0.956, 1.374)
1.381 (0.759, 2.512)	1.129 (0.912, 1.397)	1.031 (0.848, 1.253)	0.679 (0.443, 1.041)	1.536 (0.887, 2.660)	1.296 (0.764, 2.199)	1.132 (0.915, 1.399)	1.109 (0.897, 1.371)
1.226 (0.693, 2.168)	1.002 (0.895, 1.121)	**0.915 (0.850, 0.985)**	**0.603 (0.410, 0.888)**	1.363 (0.811, 2.290)	1.150 (0.700, 1.891)	1.004 (0.899, 1.122)	0.984 (0.881, 1.099)

0.691 (0.361, 1.321)	0.637 (0.334, 1.215)	0.738 (0.368, 1.481)	0.703 (0.387, 1.279)	0.700 (0.389, 1.261)	0.724 (0.398, 1.317)	0.816 (0.461, 1.443)
0.845 (0.608, 1.174)	0.780 (0.565, 1.076)	0.903 (0.597, 1.367)	0.861 (0.695, 1.065)	0.857 (0.714, 1.028)	0.886 (0.716, 1.096)	0.998 (0.892, 1.117)
0.925 (0.674, 1.271)	0.854 (0.626, 1.165)	0.989 (0.659, 1.483)	0.942 (0.775, 1.146)	0.938 (0.798, 1.102)	0.970 (0.798, 1.179)	**1.093 (1.015, 1.176)**
1.404 (0.856, 2.303)	1.295 (0.793, 2.116)	1.501 (0.861, 2.615)	1.430 (0.933, 2.191)	1.423 (0.942, 2.150)	1.472 (0.960, 2.256)	**1.658 (1.126, 2.441)**
0.621 (0.364, 1.059)	0.573 (0.337, 0.973)	0.664 (0.345, 1.277)	0.632 (0.365, 1.096)	0.630 (0.368, 1.079)	0.651 (0.376, 1.128)	0.734 (0.437, 1.232)
0.736 (0.441, 1.228)	0.679 (0.409, 1.129)	0.787 (0.416, 1.488)	0.750 (0.442, 1.272)	0.746 (0.445, 1.252)	0.772 (0.455, 1.310)	0.869 (0.529, 1.429)
0.843 (0.618, 1.149)	0.778 (0.574, 1.053)	0.901 (0.596, 1.363)	0.858 (0.694, 1.061)	0.855 (0.713, 1.024)	0.884 (0.715, 1.092)	0.996 (0.891, 1.112)
0.860 (0.633, 1.168)	0.794 (0.588, 1.071)	0.920 (0.608, 1.391)	0.876 (0.709, 1.083)	0.872 (0.728, 1.046)	0.902 (0.730, 1.115)	1.016 (0.910, 1.135)
Lemborexant 15mg	0.923 (0.666, 1.279)	1.069 (0.646, 1.770)	1.019 (0.712, 1.457)	1.014 (0.721, 1.425)	1.049 (0.733, 1.500)	1.181 (0.868, 1.609)
1.084 (0.782, 1.502)	Lemborexant 25mg	1.159 (0.703, 1.911)	1.104 (0.776, 1.570)	1.099 (0.787, 1.535)	1.137 (0.799, 1.616)	1.280 (0.947, 1.732)
0.935 (0.565, 1.549)	0.863 (0.523, 1.423)	Daridorexant 5mg	0.953 (0.636, 1.427)	0.948 (0.639, 1.407)	0.981 (0.654, 1.472)	1.105 (0.742, 1.646)
0.982 (0.686, 1.404)	0.906 (0.637, 1.288)	1.050 (0.701, 1.572)	Daridorexant 10mg	0.996 (0.838, 1.183)	1.029 (0.824, 1.286)	1.160 (0.968, 1.390)

0.986 (0.702, 1.386)	0.910 (0.651, 1.271)	1.054 (0.711, 1.565)	1.005 (0.845, 1.193)	Daridorexant 25mg	1.034 (0.868, 1.232)	**1.165 (1.009, 1.345)**
0.954 (0.667, 1.364)	0.880 (0.619, 1.251)	1.020 (0.679, 1.530)	0.971 (0.778, 1.213)	0.967 (0.812, 1.152)	Daridorexant 50mg	1.127 (0.940, 1.350)
0.847 (0.622, 1.153)	0.781 (0.577, 1.056)	0.905 (0.608, 1.348)	0.862 (0.719, 1.033)	**0.858 (0.744, 0.991)**	0.888 (0.741, 1.064)	Placebo

eTable 56: Network meta-analysis results of LPS in FDA-approved DORAs doses.

Suvorexant 10mg	9.695 (-2.216, 21.607)	12.601 (-1.125, 26.327)	**14.601 (0.905, 28.297)**	9.498 (-2.428, 21.425)	10.399 (-1.610, 22.408)	1.001 (-10.612, 12.613)
-9.695 (-21.607, 2.216)	Suvorexant 20mg	2.906 (-5.295, 11.105)	4.906 (-3.244, 13.055)	-0.197 (-4.786, 4.393)	0.704 (-4.097, 5.504)	**-8.694 (-12.393, -4.996)**
-12.601 (-26.327, 1.125)	-2.906 (-11.105, 5.295)	Lemborexant 5mg	2.000 (-5.046, 9.046)	-3.102 (-10.909, 4.705)	-2.202 (-10.135, 5.731)	**-11.600 (-18.919, -4.281)**
**-14.601 (-28.297, -0.905)**	-4.906 (-13.055, 3.244)	-2.000 (-9.046, 5.046)	Lemborexant 10mg	-5.102 (-12.856, 2.651)	-4.202 (-12.082, 3.678)	**-13.600 (-20.862, -6.338)**
-9.498 (-21.425, 2.428)	0.197 (-4.393, 4.786)	3.102 (-4.705, 10.909)	5.102 (-2.651, 12.856)	Daridorexant 25mg	0.900 (-2.157, 3.958)	**-8.498 (-11.215, -5.780)**
-10.399 (-22.408, 1.610)	-0.704 (-5.504, 4.097)	2.202 (-5.731, 10.135)	4.202 (-3.678, 12.082)	-0.900 (-3.958, 2.157)	Daridorexant 50mg	**-9.398 (-12.458, -6.338)**
-1.001 (-12.613, 10.612)	**8.694 (4.996, 12.393)**	**11.600 (4.281, 18.919)**	**13.600 (6.338, 20.862)**	**8.498 (5.780, 11.215)**	**9.398 (6.338, 12.458)**	Placebo

eTable 57: Network meta-analysis results of sTSO in FDA-approved DORAs doses.

Suvorexant 10mg	2.368 (-5.010, 9.745)	**12.921 (4.046, 21.797)**	**13.159 (4.244, 22.074)**	-2.939 (-10.154, 4.277)	2.647 (-4.569, 9.862)	-3.392 (-10.528, 3.744)
-2.368 (-9.745, 5.010)	Suvorexant 20mg	**10.553 (4.602, 16.505)**	**10.791 (4.781, 16.802)**	-**5.306 (-8.258, -2.354)**	0.279 (-2.672, 3.229)	**-5.760 (-8.511, -3.008)**
**-12.921 (-21.797, -4.046)**	**-10.553 (-16.505, -4.602)**	Lemborexant 5mg	0.238 (-5.381, 5.857)	**-15.860(-21.245, -10.475)**	**-10.275 (-15.659, -4.890)**	**-16.313 (-21.591, -11.035)**
**-13.159 (-22.074, -4.244)**	**-10.791 (-16.802, -4.781)**	-0.238 (-5.857, 5.381)	Lemborexant 10mg	**-16.098(-21.548, -10.648)**	**-10.512 (-15.962, -5.063)**	**-16.551 (-21.895, -11.207)**
2.939 (-4.277, 10.154)	**5.306 (2.354, 8.258)**	**15.860 (10.475, 21.245)**	**16.098 (10.648, 21.548)**	Daridorexant 25mg	**5.585 (4.520, 6.650)**	-0.453 (-1.523, 0.617)
-2.647 (-9.862, 4.569)	-0.279 (-3.229, 2.672)	**10.275 (4.890, 15.659)**	**10.512 (5.063, 15.962)**	**-5.585 (-6.650, -4.520)**	Daridorexant 50mg	**-6.038 (-7.104, -4.972)**
3.392 (-3.744, 10.528)	**5.760 (3.008, 8.511)**	**16.313 (11.035, 21.591)**	**16.551 (11.207, 21.895)**	0.453 (-0.617, 1.523)	**6.038 (4.972, 7.104)**	Placebo

eTable 58: Network meta-analysis results of WASO in FDA-approved DORAs doses.

Suvorexant 10mg	4.350 (-12.176, 20.877)	4.778 (-14.896, 24.453)	7.278 (-12.399, 26.956)	-9.648 (-26.304, 7.008)	-0.150 (-17.013, 16.713)	**-20.522 (-36.597, -4.447)**
-4.350 (-20.877,12.176)	Suvorexant 20mg	0.428 (-12.260, 13.117)	2.928 (-9.764, 15.620)	**-13.998 (-21.163, -6.834)**	-4.500 (-12.132, 3.132)	**-24.872 (-30.555, -19.189)**
-4.778 (-24.453, 14.896)	-0.428 (-13.117, 12.260)	Lemborexant 5mg	2.500 (-8.393, 13.393)	**-14.426 (-26.581, -2.272)**	-4.928 (-17.364, 7.507)	**-25.300 (-36.645, -13.956)**
-7.278 (-26.956, 12.399)	-2.928 (-15.620, 9.764)	-2.500 (-13.393, 8.393)	Lemborexant 10mg	**-16.926 (-29.084, -4.768)**	-7.428 (-19.868, 5.011)	**-27.800 (-39.148, -16.452)**
9.648 (-7.008, 26.304)	**13.998 (6.834, 21.163)**	**14.426 (2.272, 26.581)**	**16.926 (4.768, 29.084)**	Daridorexant 25mg	**9.498 (4.408, 14.588)**	**-10.874 (-15.236, -6.511)**
0.150 (-16.713, 17.013)	4.500 (-3.132, 12.132)	4.928 (-7.507, 17.364)	7.428 (-5.011, 19.868)	**-9.498 (-14.588, -4.408)**	Daridorexant 50mg	**-20.372 (-25.465, -15.278)**
**20.522 (4.447, 36.597)**	**24.872 (19.189, 30.555)**	**25.300 (13.956, 36.645)**	**27.800 (16.452, 39.148)**	**10.874 (6.511, 15.236)**	**20.372 (15.278, 25.465)**	O_Placebo

eTable 59: Network meta-analysis results of sWASO in FDA-approved DORAs doses.

Suvorexant 20mg	**11.685 (8.685, 14.685)**	**6.934 (3.934, 9.934)**	2.013 (-1.285, 5.311)	**5.894 (2.583, 9.206)**	**-5.756 (-8.702, -2.810)**
**-11.685 (-14.685, -8.685)**	Lemborexant 5mg	**-4.751 (-5.324, -4.178)**	**-9.672 (-11.259, -8.086)**	**-5.791 (-7.405, -4.177)**	**-17.441 (-18.006, -16.876)**
**-6.934 (-9.934, -3.934)**	**4.751 (4.178, 5.324)**	Lemborexant 10mg	**-4.922 (-6.509, -3.334)**	-1.040 (-2.655, 0.575)	**-12.690 (-13.258, -12.122)**
-2.013 (-5.311, 1.285)	**9.672 (8.086, 11.259)**	**4.922 (3.334, 6.509)**	Daridorexant 25mg	**3.882 (2.388, 5.376)**	**-7.769 (-9.251, -6.286)**
**-5.894 (-9.206, -2.583)**	**5.791 (4.177, 7.405)**	1.040 (-0.575, 2.655)	**-3.882 (-5.376, -2.388)**	Daridorexant 50mg	**-11.650 (-13.162, -10.138)**
**5.756 (2.810, 8.702)**	**17.441 (16.876, 18.006)**	**12.690 (12.122, 13.258)**	**7.769 (6.286, 9.251)**	**11.650 (10.138, 13.162)**	Placebo

eTable 60: Network meta-analysis results of TST in FDA-approved DORAs doses.

Suvorexant 10mg	-27.600 (-70.074, 14.874)	-5.215 (-53.984, 43.554)	-16.467 (-65.291, 32.357)	22.300 (-18.085, 62.685)
27.600 (-14.874, 70.074)	Suvorexant 20mg	22.385 (-26.476, 71.246)	11.133 (-37.782, 60.049)	**49.900 (9.404, 90.396)**
5.215 (-43.554, 53.984)	-22.385 (-71.246, 26.476)	Daridorexant 25mg	-11.252 (-38.691, 16.188)	**27.515 (0.175, 54.855)**
16.467 (-32.357, 65.291)	-11.133 (-60.049, 37.782)	11.252 (-16.188, 38.691)	Daridorexant 50mg	**38.767 (11.329, 66.205)**
-22.300 (-62.685, 18.085)	**-49.900 (-90.396, -9.404)**	**-27.515 (-54.855, -0.175)**	**-38.767 (-66.205, -11.329)**	Placebo

eTable 61: Network meta-analysis results of sTST in FDA-approved DORAs doses.

Suvorexant 10mg	-6.087 (-19.555, 7.381)	-9.563 (-22.631, 3.505)	**-13.693 (-26.762, -0.625)**	-5.156 (-18.813, 8.501)	**-14.435 (-28.481, -0.390)**	8.987 (-4.061, 22.035)
6.087 (-7.381, 19.555)	Suvorexant 20mg	-3.476 (-7.891, 0.939)	**-7.606 (-12.022, -3.190)**	0.931 (-5.005, 6.867)	**-8.348 (-15.130, -1.567)**	**15.074 (10.719, 19.430)**
9.563 (-3.505, 22.631)	3.476 (-0.939, 7.891)	Lemborexant 5mg	**-4.130 (-4.865, -3.395)**	**4.407 (0.310, 8.504)**	-4.872 (-10.120, 0.375)	**18.550 (17.829, 19.271)**
**13.693 (0.625, 26.762)**	**7.606 (3.190, 12.022)**	**4.130 (3.395, 4.865)**	Lemborexant 10mg	**8.537 (4.438, 12.635)**	-0.742 (-5.991, 4.507)	**22.680 (21.951, 23.409)**
5.156 (-8.501, 18.813)	-0.931 (-6.867, 5.005)	**-4.407 (-8.504, -0.310)**	**-8.537 (-12.635, -4.438)**	Daridorexant 25mg	**-9.279 (-14.465, -4.093)**	**14.143 (10.111, 18.176)**
**14.435 (0.390, 28.481)**	**8.348 (1.567, 15.130)**	4.872 (-0.375, 10.120)	0.742 (-4.507, 5.991)	**9.279 (4.093, 14.465)**	Daridorexant 50mg	**23.422 (18.224, 28.620)**
-8.987 (-22.035, 4.061)	**-15.074 (-19.430, -10.719)**	**-18.55 (-19.271, -17.829)**	**-22.68 (-23.409, -21.951)**	**-14.143 (-18.176, -10.111)**	**-23.422 (-28.62, -18.224)**	Placebo

eTable 62: Network meta-analysis results of ISI in FDA-approved DORAs doses.

Suvorexant 20mg	0.432 (-0.651, 1.516)	0.532 (-0.586, 1.651)	-1.067 (-3.125, 0.990)	-0.468 (-2.589, 1.654)	**-1.268 (-1.636, -0.899)**
-0.432 (-1.516, 0.651)	Lemborexant 5mg	0.100 (-0.890, 1.090)	-1.500 (-3.766, 0.766)	-0.900 (-3.225, 1.425)	**-1.700 (-2.719, -0.681)**
-0.532 (-1.651, 0.586)	-0.100 (-1.090, 0.890)	Lemborexant 10mg	-1.600 (-3.883, 0.683)	-1.000 (-3.341, 1.341)	**-1.800 (-2.856, -0.744)**
1.067 (-0.990, 3.125)	1.500 (-0.766, 3.766)	1.600 (-0.683, 3.883)	Daridorexant 25mg	0.600 (-1.574, 2.774)	-0.200 (-2.224, 1.824)
0.468 (-1.654, 2.589)	0.900 (-1.425, 3.225)	1.000 (-1.341, 3.341)	-0.600 (-2.774, 1.574)	Daridorexant 50mg	-0.800 (-2.890, 1.290)
**1.268 (0.899, 1.636)**	**1.700 (0.681, 2.719)**	**1.800 (0.744, 2.856)**	0.200 (-1.824, 2.224)	0.800 (-1.290, 2.890)	Placebo

eTable 63: Network meta-analysis results of AEs in FDA-approved DORAs doses.

Suvorexant 10mg	0.892 (0.495, 1.607)	0.890 (0.493, 1.607)	0.872 (0.483, 1.575)	0.760 (0.418, 1.381)	0.756 (0.412, 1.387)	0.886 (0.496, 1.583)
1.121 (0.622, 2.018)	Suvorexant 20mg	0.997 (0.848, 1.172)	0.977 (0.831, 1.149)	0.851 (0.708, 1.024)	0.847 (0.685, 1.047)	0.993 (0.882, 1.117)
1.124 (0.622, 2.030)	1.003 (0.853, 1.179)	Lemborexant 5mg	0.980 (0.877, 1.095)	0.854 (0.713, 1.022)	0.849 (0.690, 1.046)	0.996 (0.891, 1.112)
1.147 (0.635, 2.072)	1.023 (0.870, 1.203)	1.021 (0.913, 1.141)	Lemborexant 10mg	0.871 (0.728, 1.043)	0.867 (0.704, 1.068)	1.016 (0.910, 1.135)
1.317 (0.724, 2.394)	1.175 (0.977, 1.412)	1.171 (0.979, 1.402)	1.148 (0.959, 1.374)	Daridorexant 25mg	0.995 (0.839, 1.180)	**1.166 (1.012, 1.344)**
1.323 (0.721, 2.428)	1.180 (0.955, 1.460)	1.177 (0.956, 1.450)	1.153 (0.937, 1.420)	1.005 (0.847, 1.192)	Daridorexant 50mg	1.172 (0.982, 1.398)
1.129 (0.632, 2.018)	1.007 (0.895, 1.133)	1.004 (0.899, 1.122)	0.984 (0.881, 1.099)	**0.857 (0.744, 0.988)**	0.853 (0.715, 1.018)	Placebo

eTable 64: Network meta-analysis results of SAEs in FDA-approved DORAs doses.

Suvorexant 10mg	2.962 (0.095, 92.038)	0.778 (0.021, 28.400)	0.737 (0.020, 26.741)	2.796 (0.077, 101.230)	2.025 (0.053, 76.884)	1.317 (0.042, 40.956)
0.338 (0.011, 10.491)	Suvorexant 20mg	0.263 (0.053, 1.315)	0.249 (0.050, 1.230)	0.944 (0.192, 4.643)	0.684 (0.125, 3.734)	0.445 (0.132, 1.494)
1.285 (0.035, 46.876)	3.805 (0.760, 19.047)	Lemborexant 5mg	0.947 (0.377, 2.381)	3.593 (0.817, 15.800)	2.602 (0.529, 12.801)	1.692 (0.586, 4.888)
1.356 (0.037, 49.210)	4.018 (0.813, 19.852)	1.056 (0.420, 2.655)	Lemborexant 10mg	3.793 (0.875, 16.450)	2.747 (0.566, 13.340)	1.787 (0.631, 5.061)
0.358 (0.010, 12.946)	1.059 (0.215, 5.209)	0.278 (0.063, 1.224)	0.264 (0.061, 1.143)	Daridorexant 25mg	0.724 (0.183, 2.860)	0.471 (0.168, 1.324)
0.494 (0.013, 18.752)	1.463 (0.268, 7.989)	0.384 (0.078, 1.891)	0.364 (0.075, 1.768)	1.381 (0.350, 5.455)	Daridorexant 50mg	0.651 (0.198, 2.136)
0.759 (0.024, 23.606)	2.249 (0.669, 7.554)	0.591 (0.205, 1.707)	0.560 (0.198, 1.585)	2.123 (0.755, 5.969)	1.537 (0.468, 5.048)	Placebo

eFigure 1: Risk of bias.

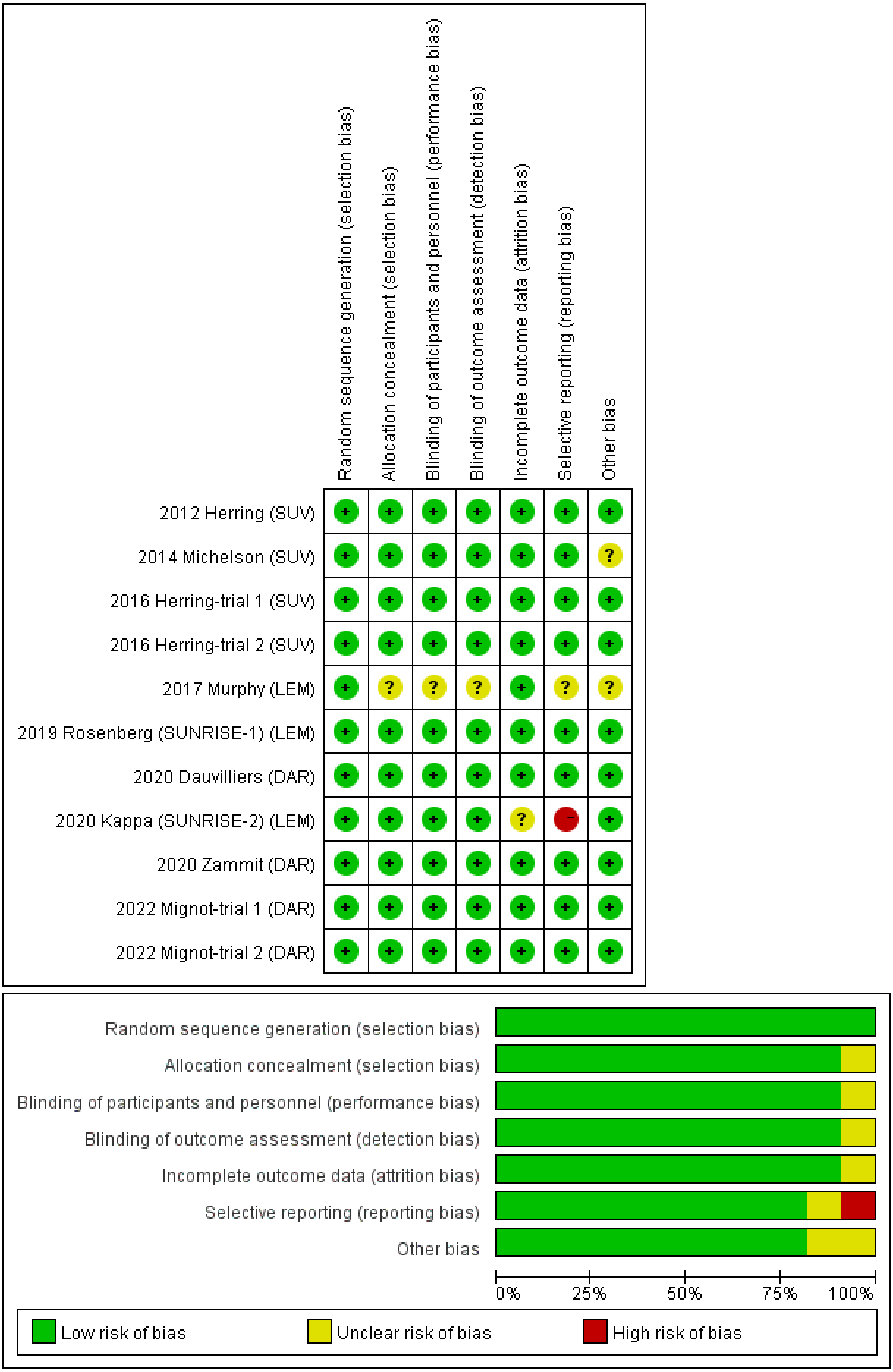

eFigure 2: Convergence diagnostics of the network meta-analysis: LPS.

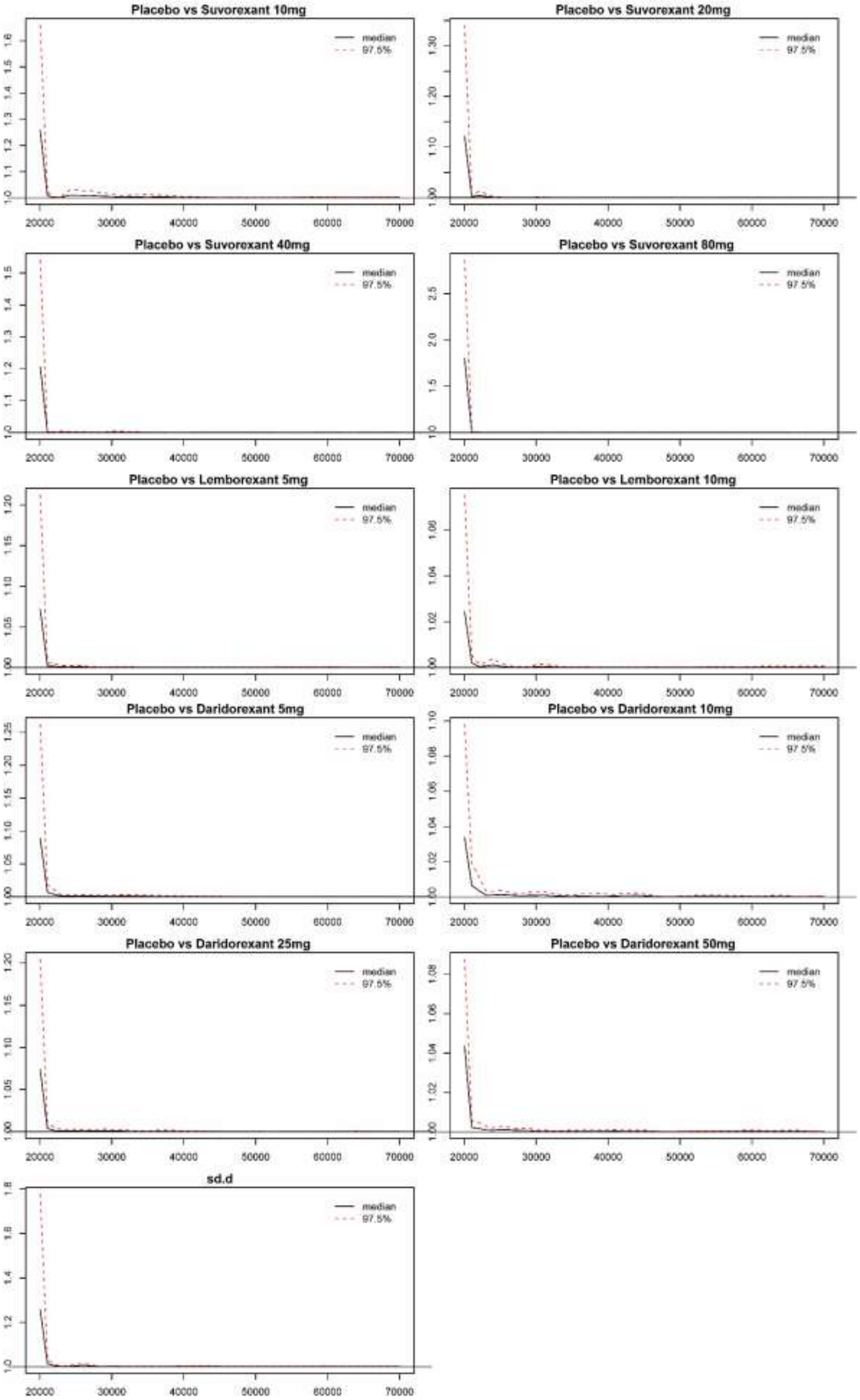

eFigure 3: Convergence diagnostics of the network meta-analysis: sTSO.

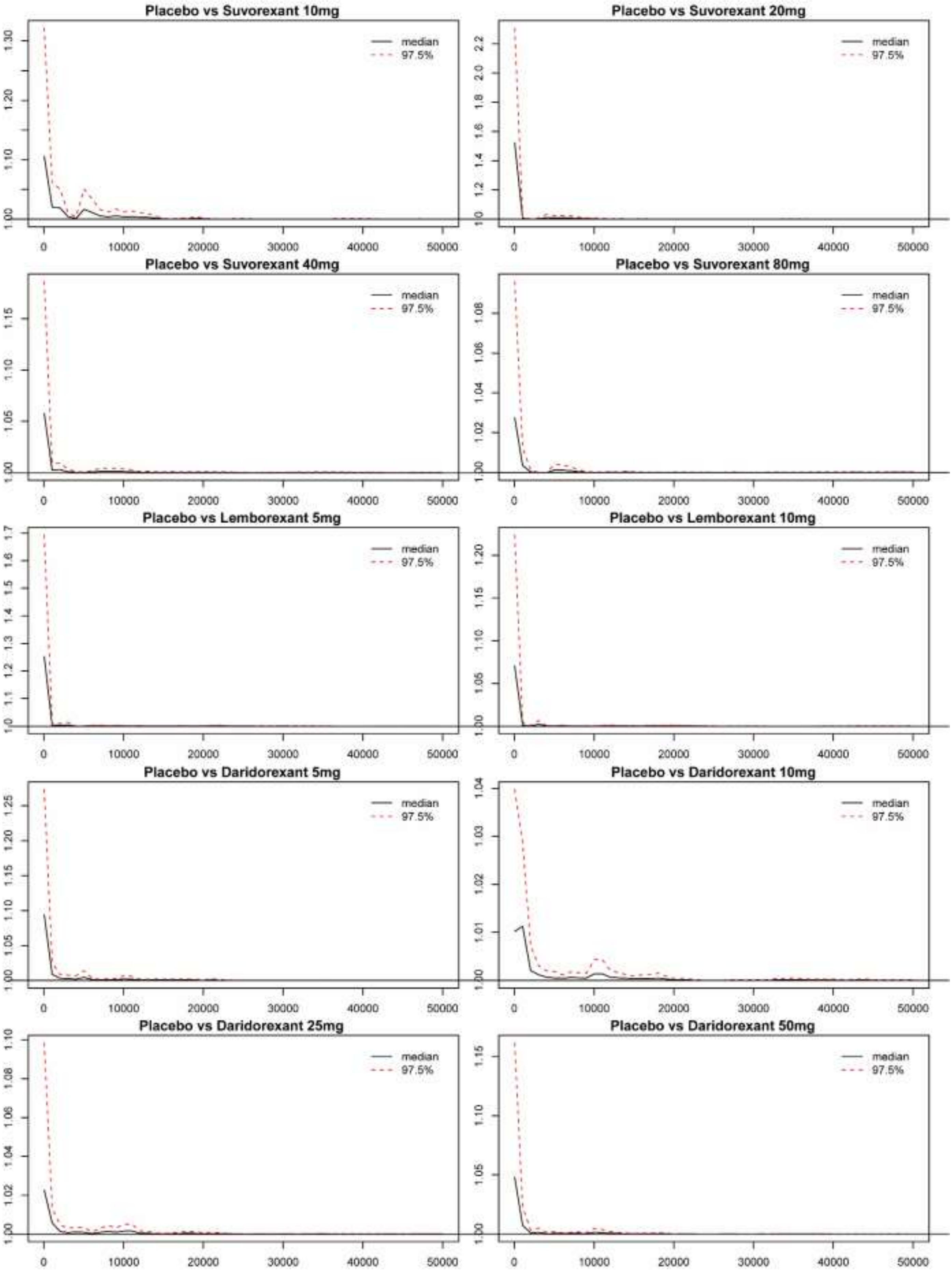

eFigure 4: Convergence diagnostics of the network meta-analysis: WASO.

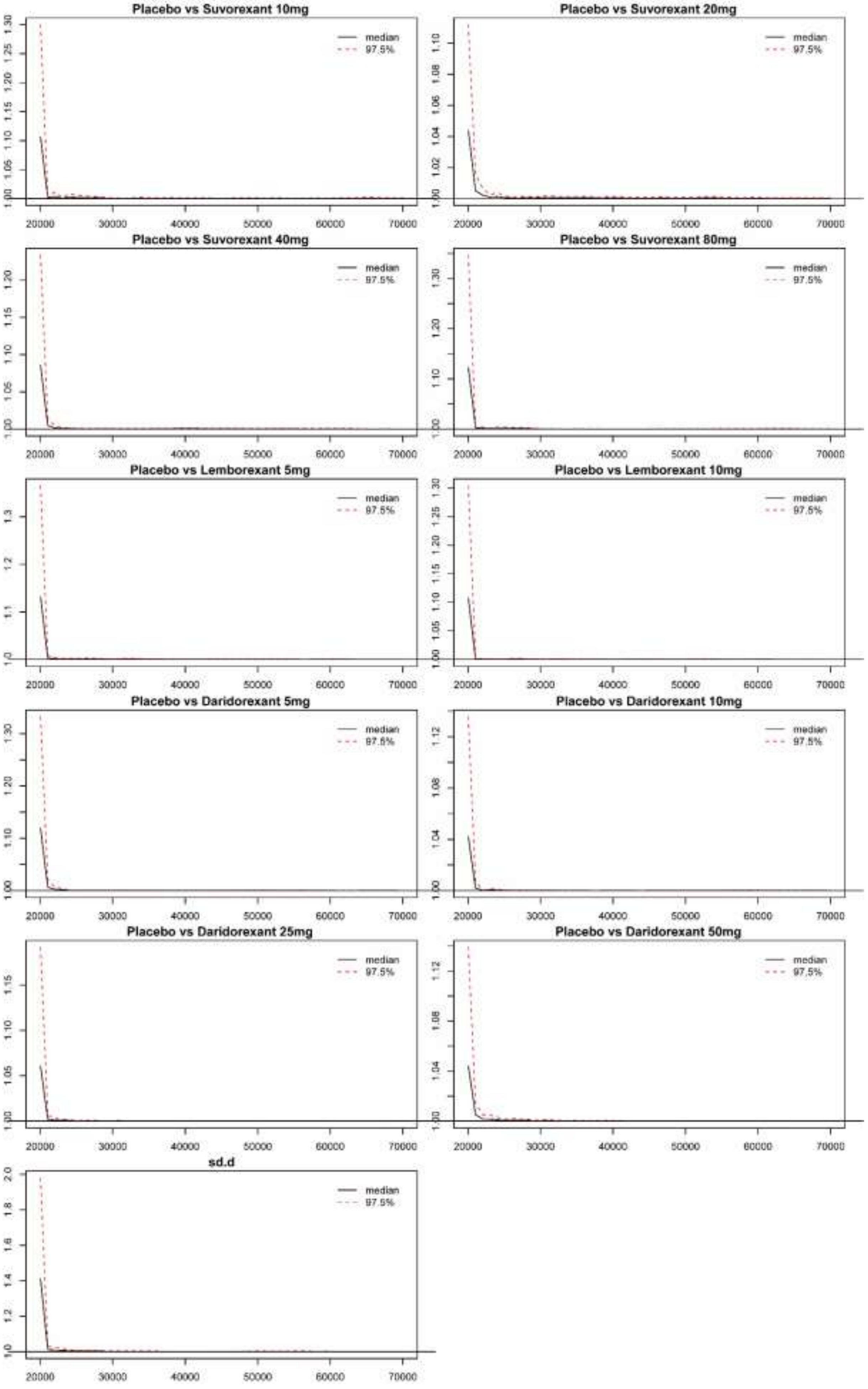

eFigure 5: Convergence diagnostics of the network meta-analysis: sWASO.

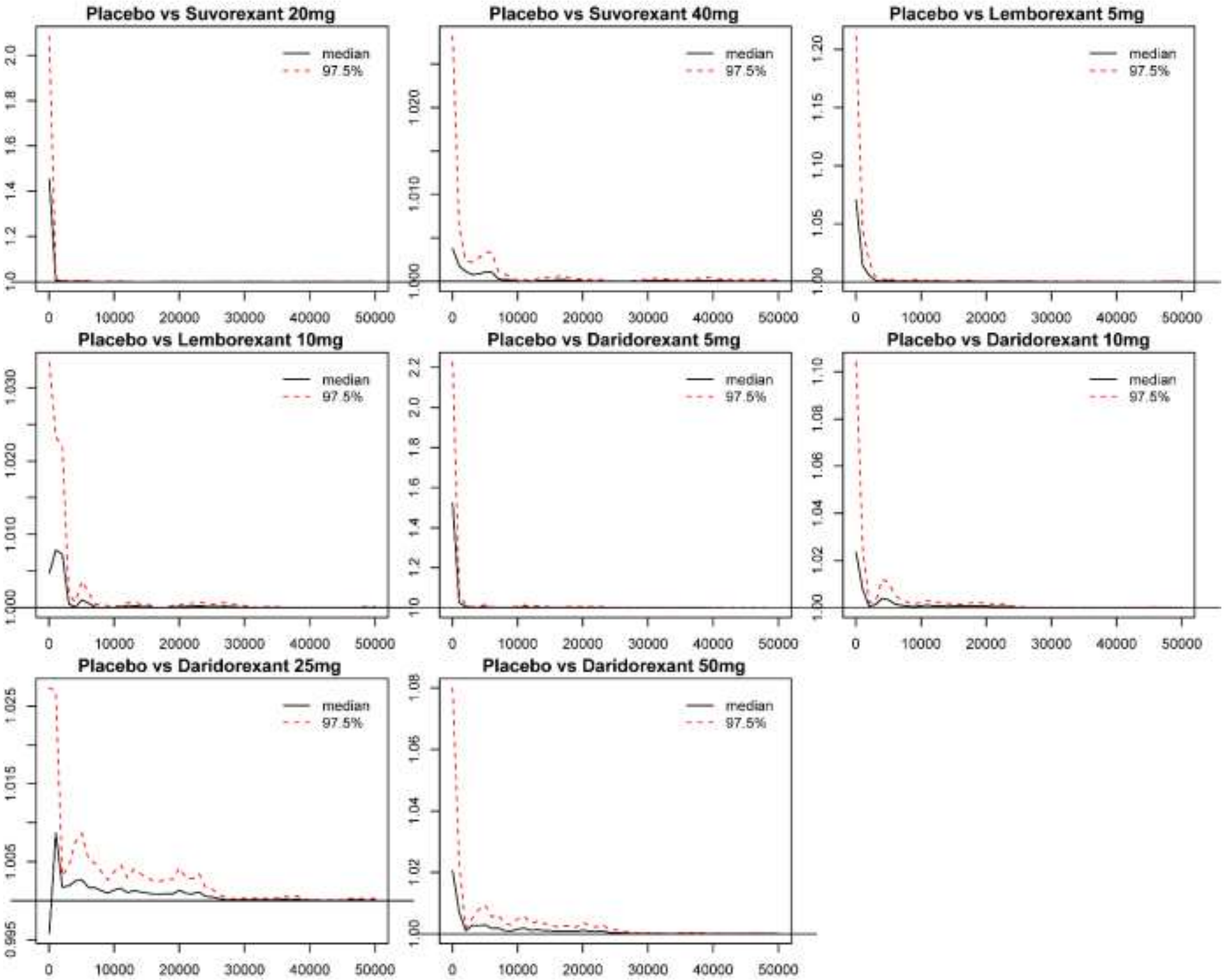

eFigure 6: Convergence diagnostics of the network meta-analysis: TST.

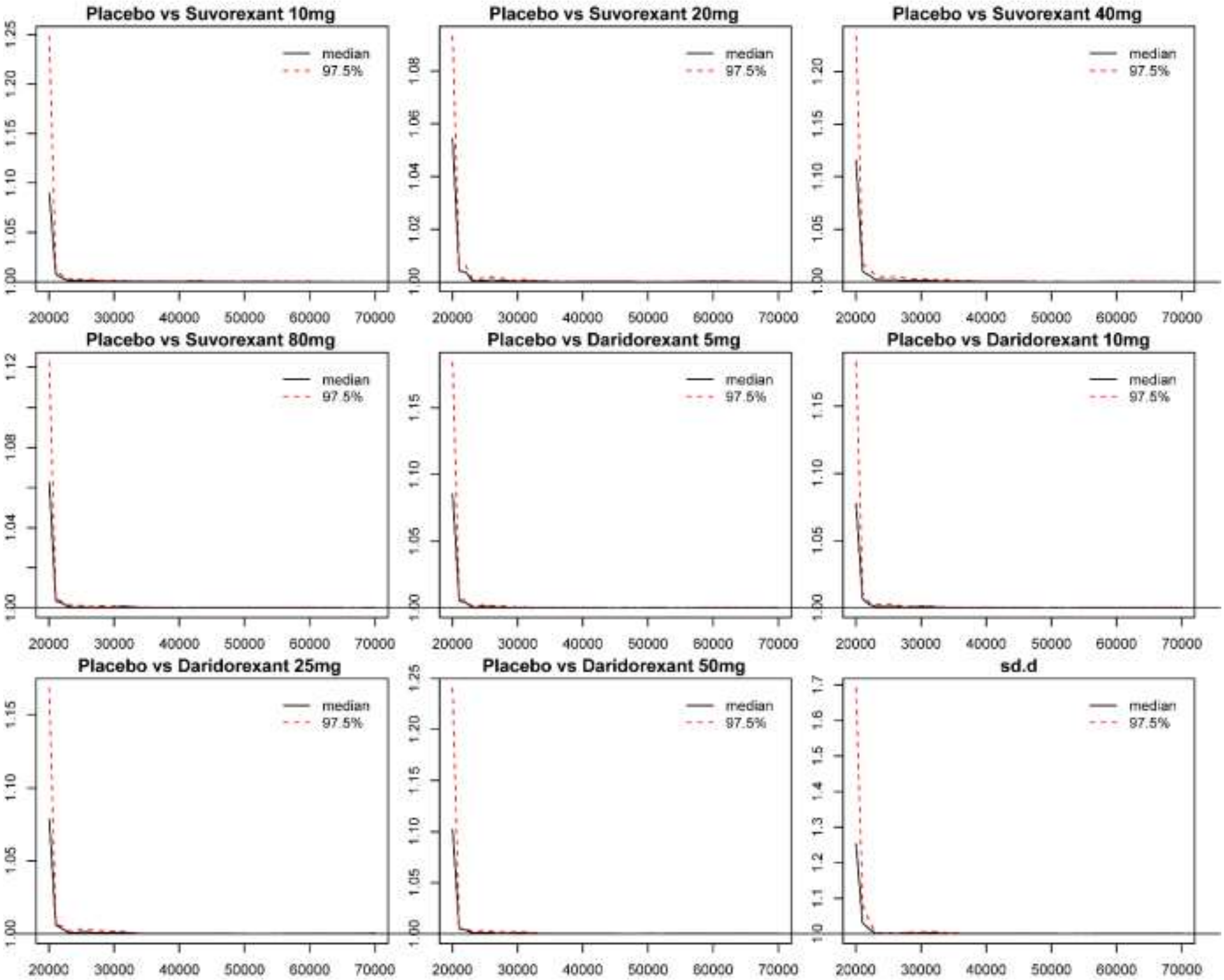

eFigure 7: Convergence diagnostics of the network meta-analysis: sTST.

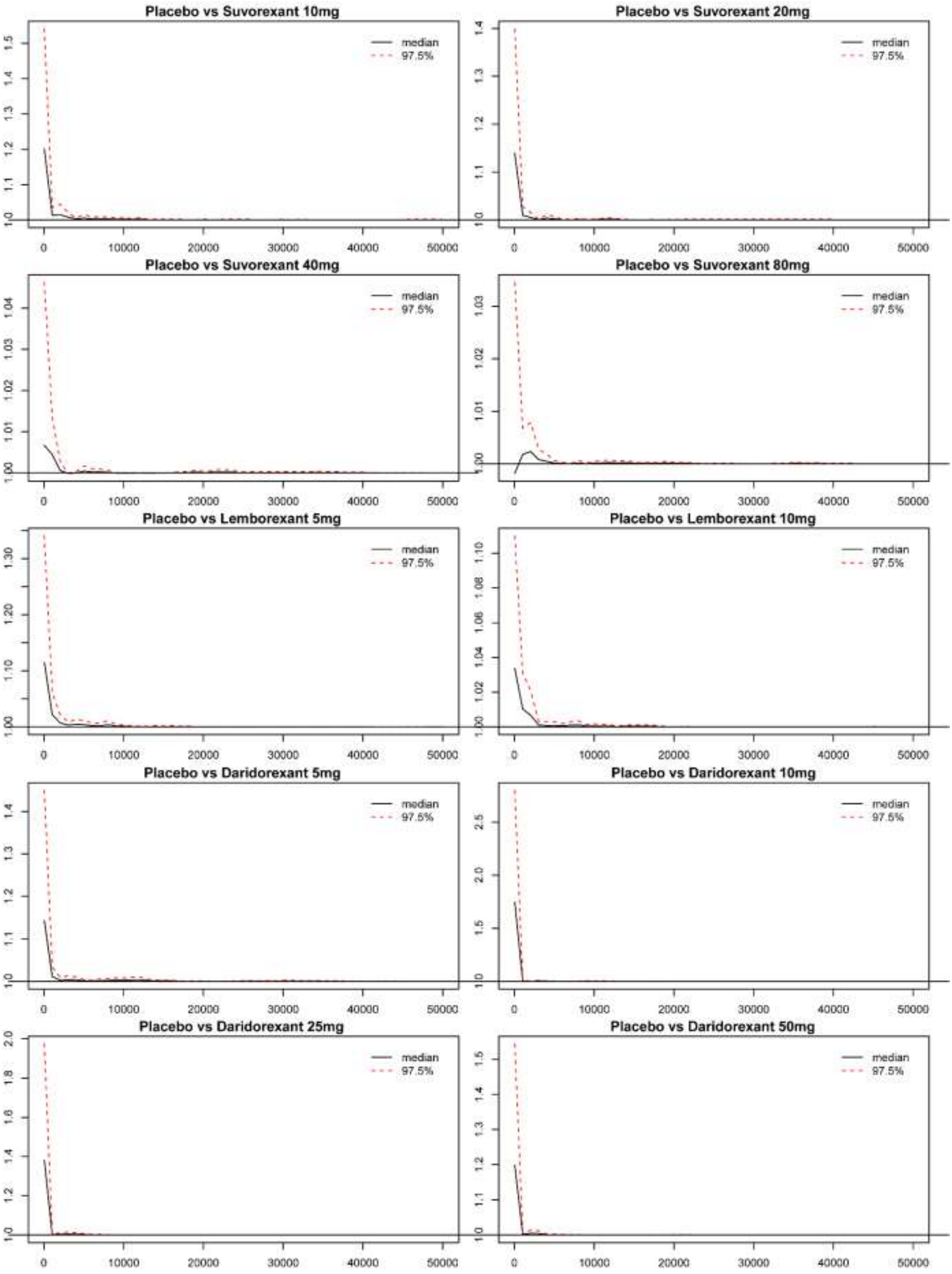

eFigure 8: Convergence diagnostics of the network meta-analysis: ISI.

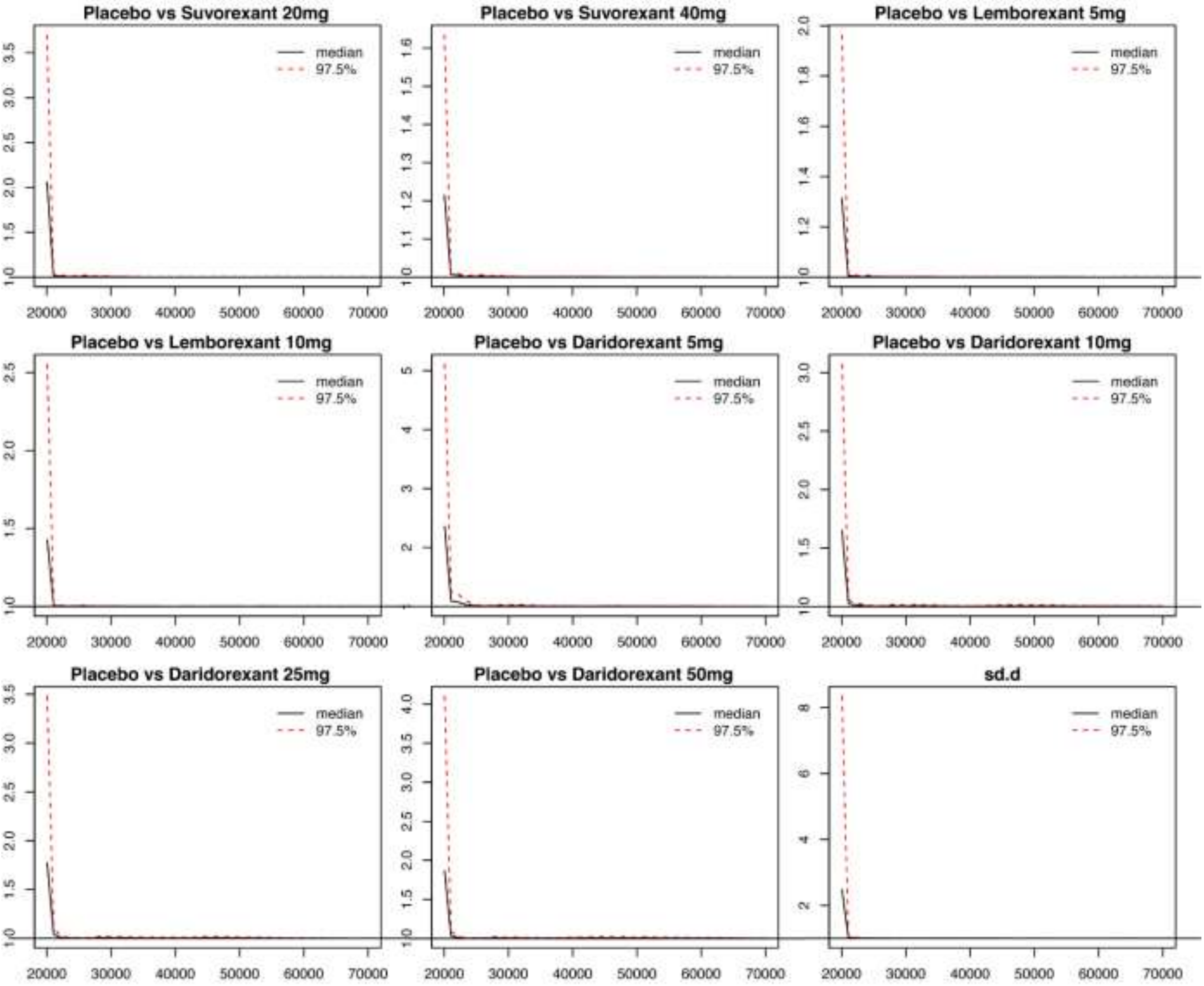

eFigure 9: Convergence diagnostics of the network meta-analysis: AE.

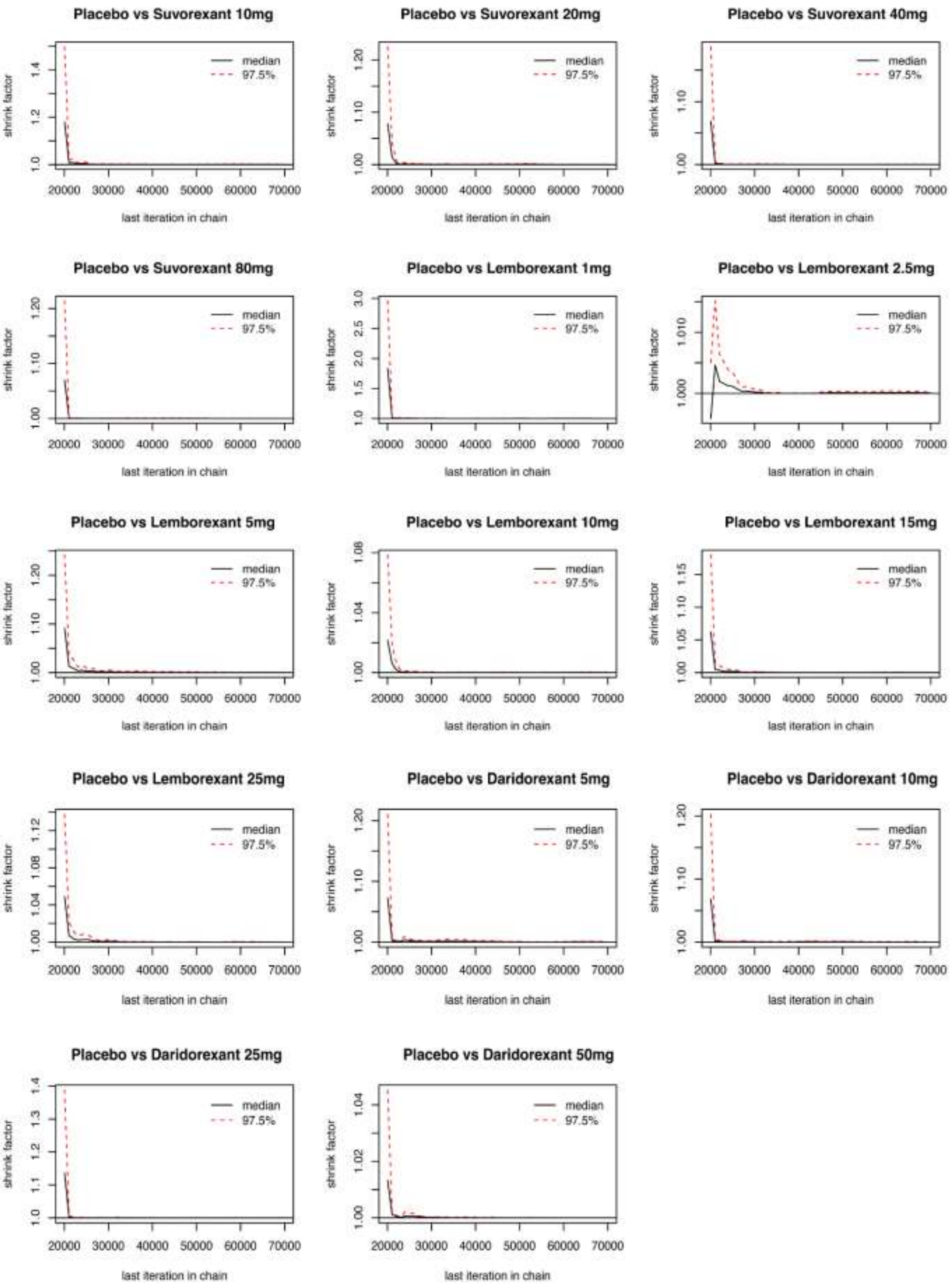

eFigure 10: Convergence diagnostics of the network meta-analysis: SAE.

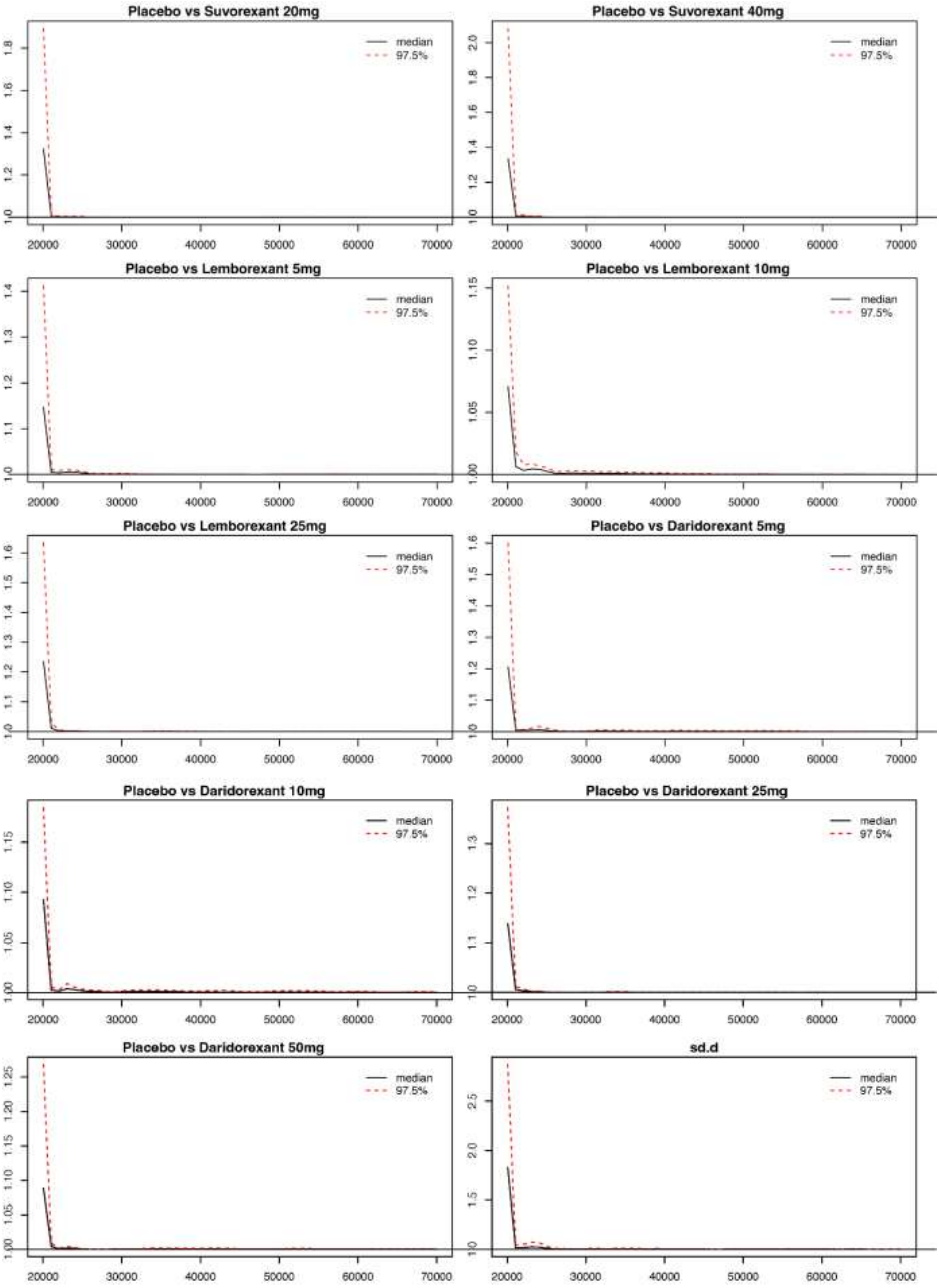

eFigure 11: Trace and density of the network meta-analysis: LPS.

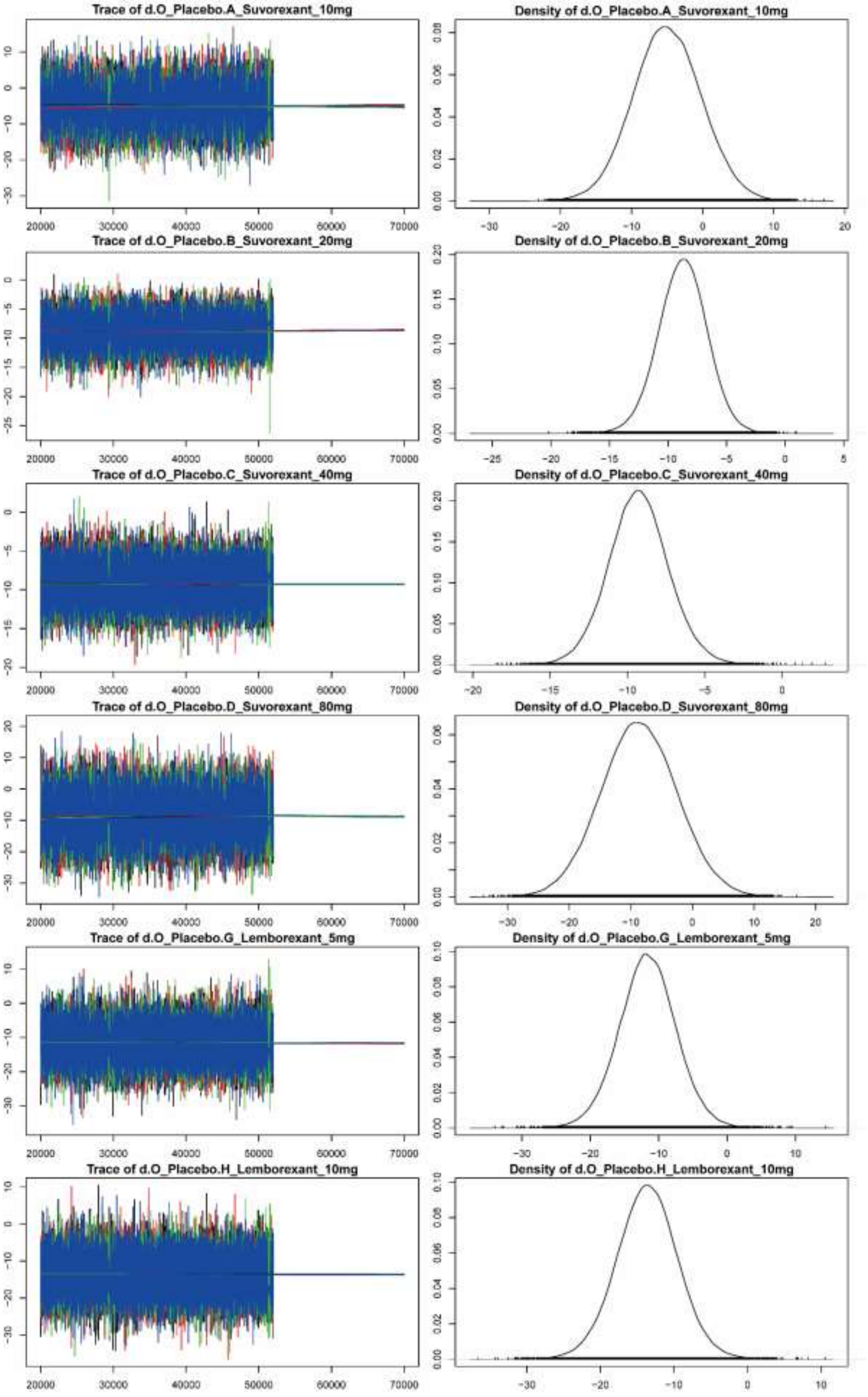

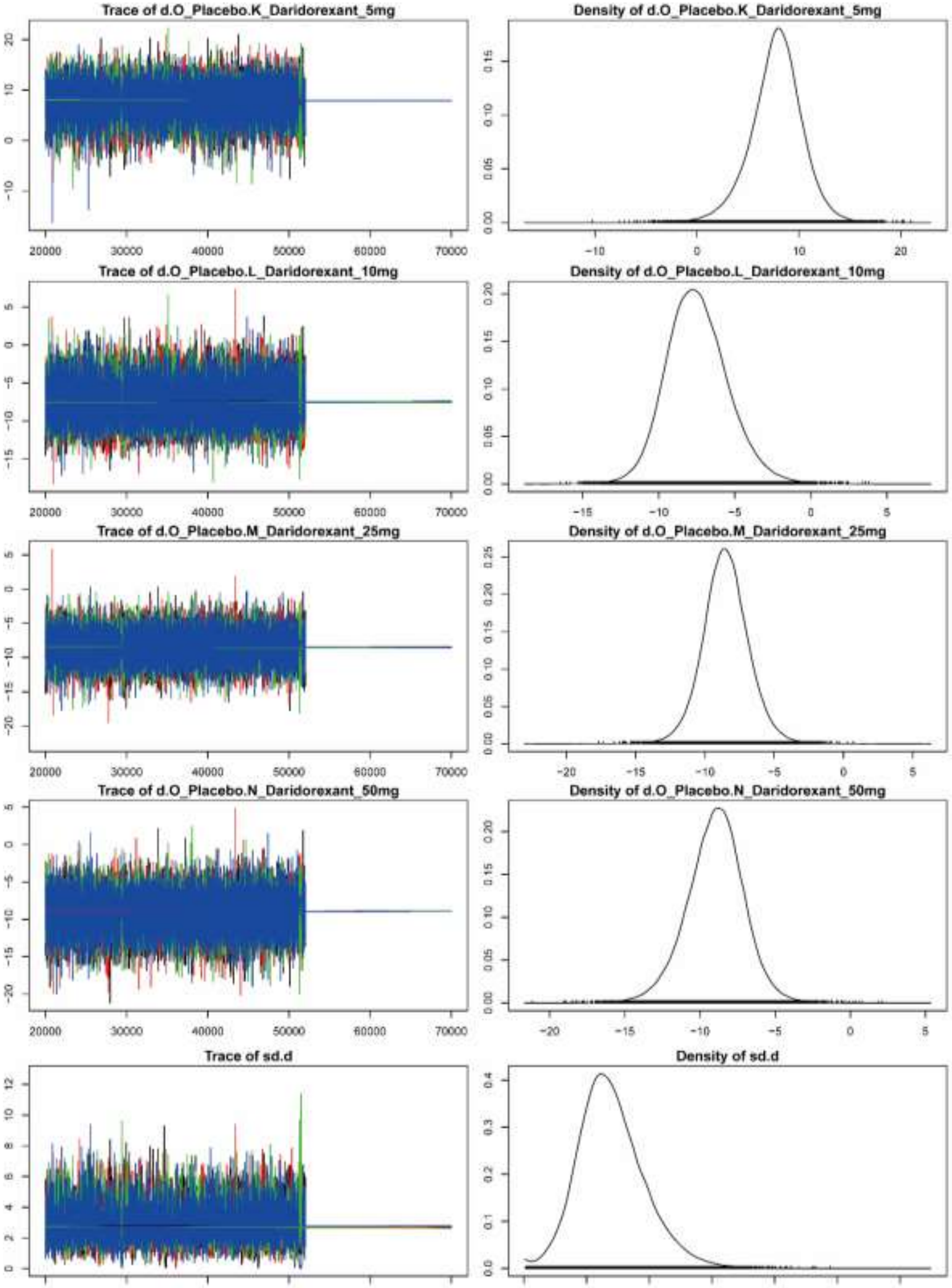

Trace of d.O_Placebo.K_Daridorexant_5mg
Density of d.O_Placebo.K_Daridorexant_5mg
Trace of d.O_Placebo.L_Daridorexant_10mg
Density of d.O_Placebo.L_Daridorexant_10mg
Trace of d.O_Placebo.M_Daridorexant_25mg
Density of d.O_Placebo.M_Daridorexant_25mg
Trace of d.O_Placebo.N_Daridorexant_50mg
Density of d.O_Placebo.N_Daridorexant_50mg
Trace of sd.d
Density of sd.d

eFigure 12: Trace and density of the network meta-analysis: sTSO.

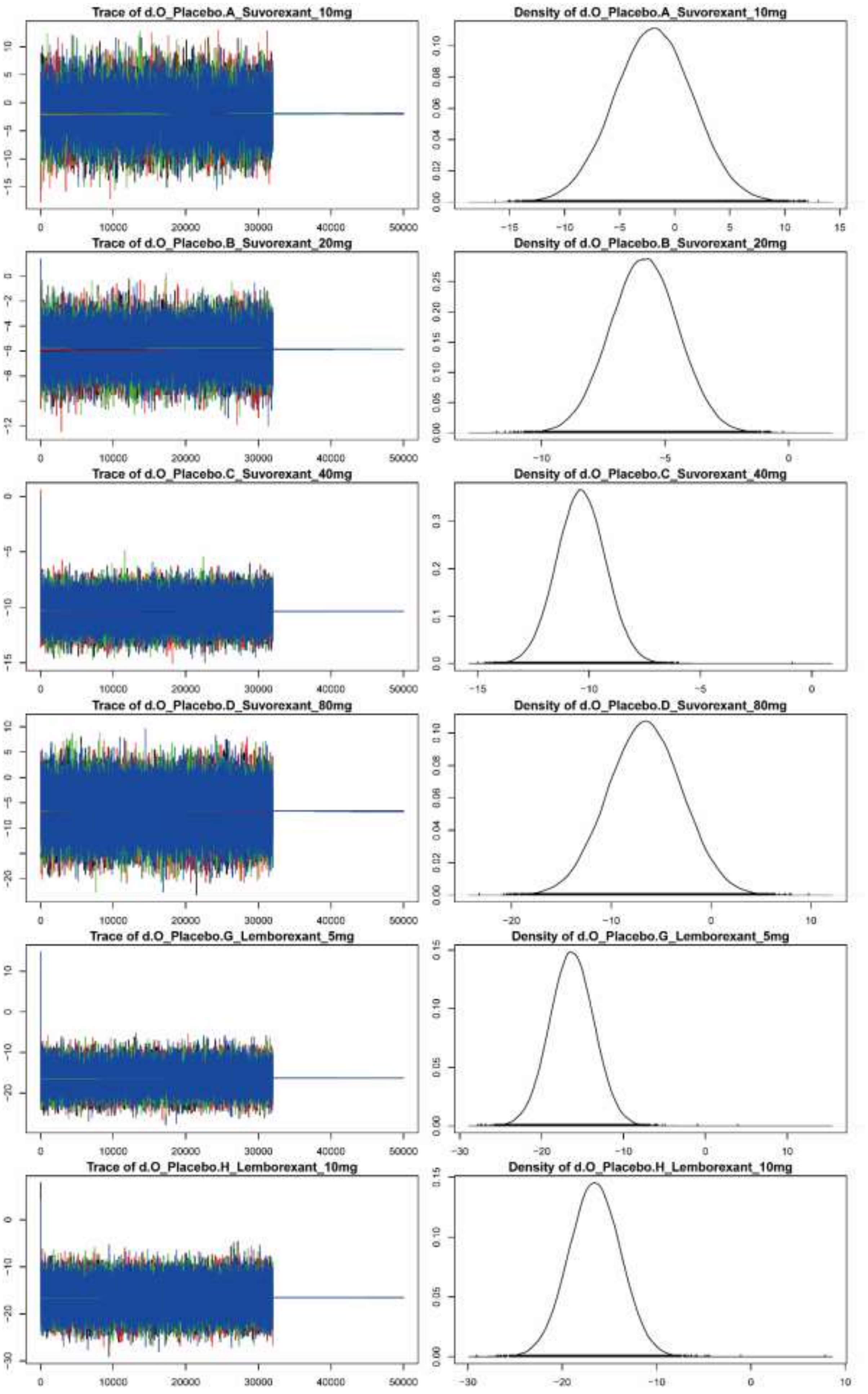

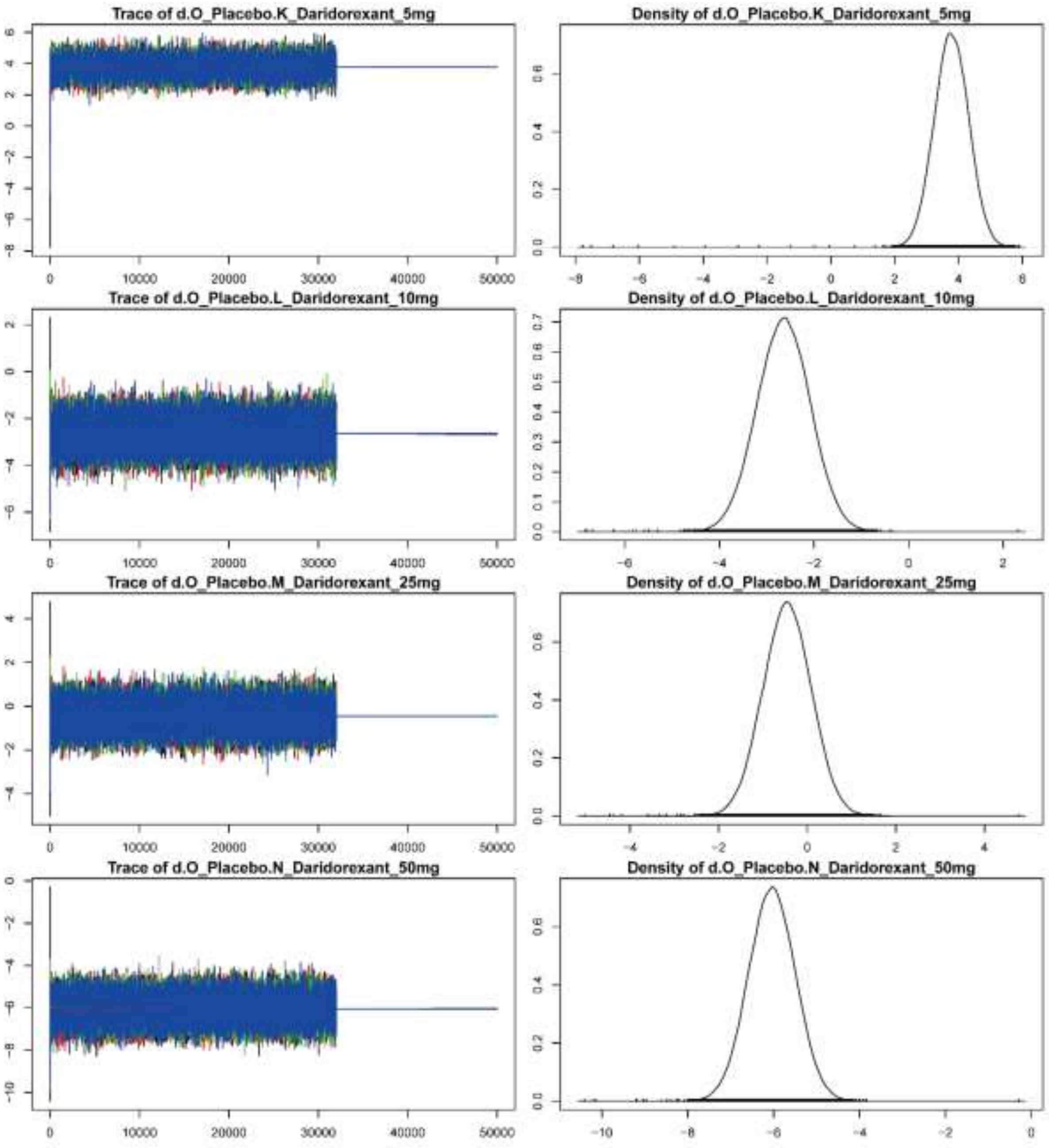
Trace of d.O_Placebo.K_Daridorexant_5mg
Density of d.O_Placebo.K_Daridorexant_5mg
Trace of d.O_Placebo.L_Daridorexant_10mg
Density of d.O_Placebo.L_Daridorexant_10mg
Trace of d.O_Placebo.M_Daridorexant_25mg
Density of d.O_Placebo.M_Daridorexant_25mg
Trace of d.O_Placebo.N_Daridorexant_50mg
Density of d.O_Placebo.N_Daridorexant_50mg

eFigure 13: Trace and density of the network meta-analysis: WASO.

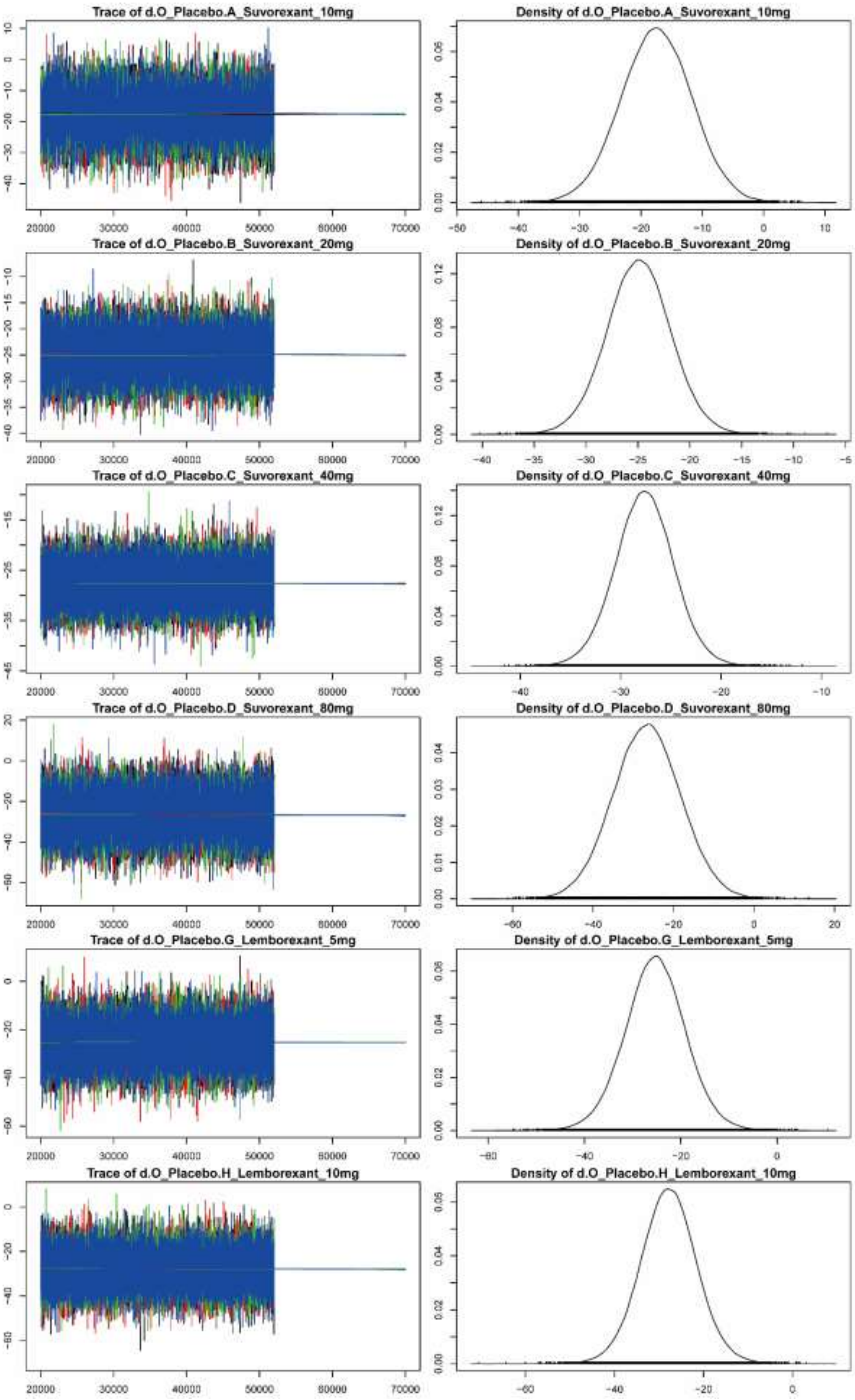

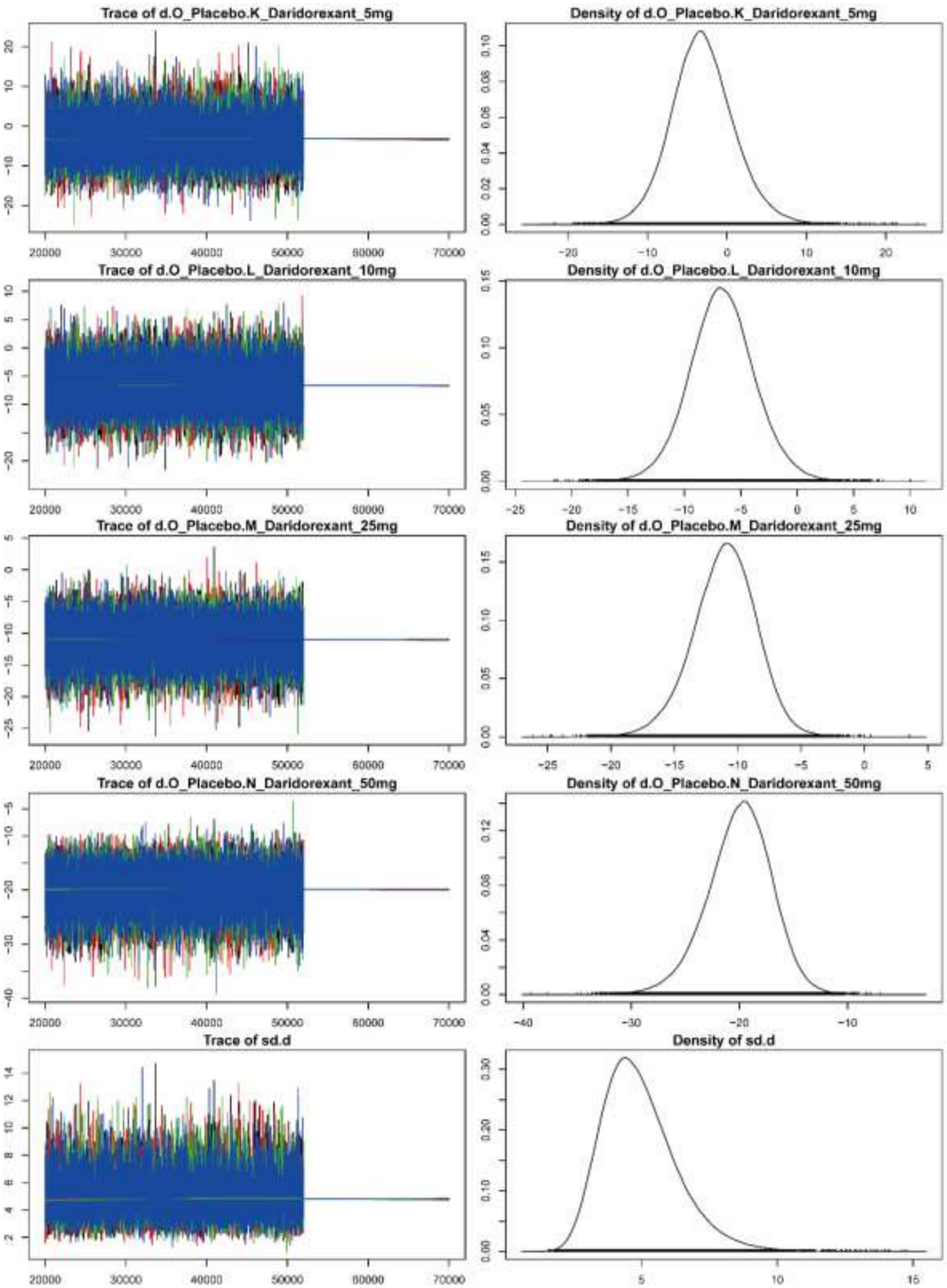
Trace of d.O_Placebo.K_Daridorexant_5mg
Density of d.O_Placebo.K_Daridorexant_5mg
Trace of d.O_Placebo.L_Daridorexant_10mg
Density of d.O_Placebo.L_Daridorexant_10mg
Trace of d.O_Placebo.M_Daridorexant_25mg
Density of d.O_Placebo.M_Daridorexant_25mg
Trace of d.O_Placebo.N_Daridorexant_50mg
Density of d.O_Placebo.N_Daridorexant_50mg
Trace of sd.d
Density of sd.d

eFigure 14: Trace and density of the network meta-analysis: sWASO.

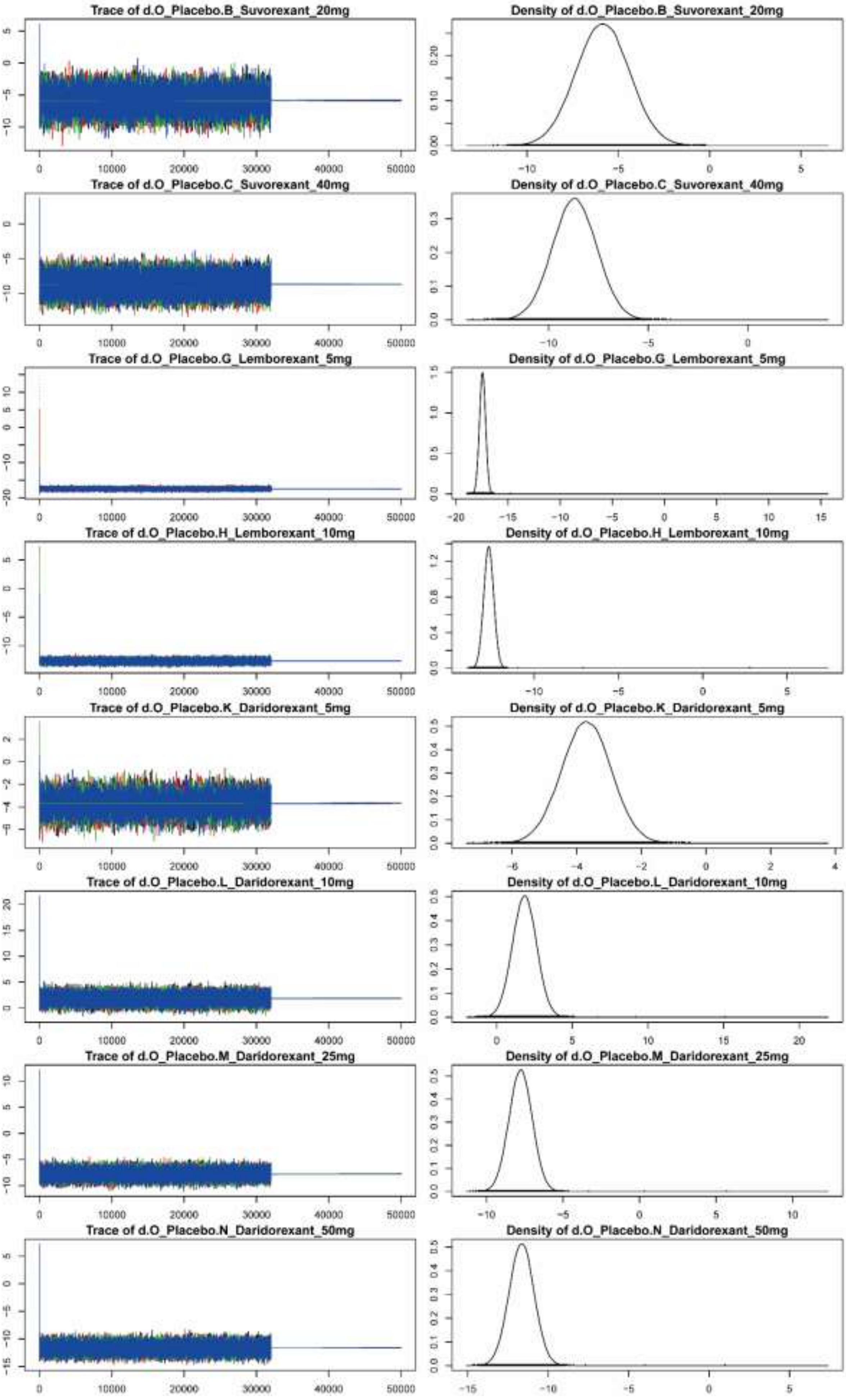

eFigure 15: Trace and density of the network meta-analysis: TST.

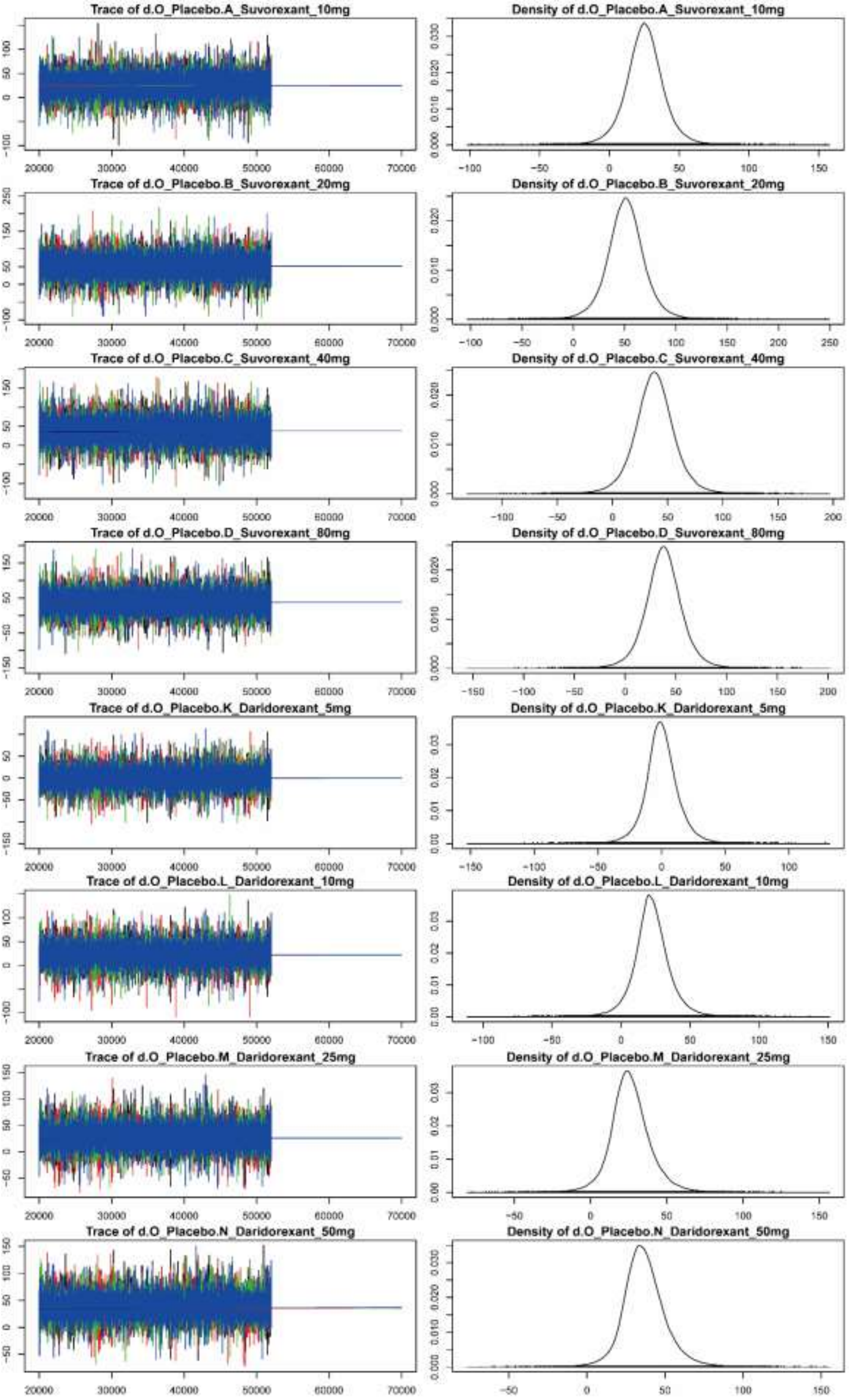

eFigure 16: Trace and density of the network meta-analysis: sTST.

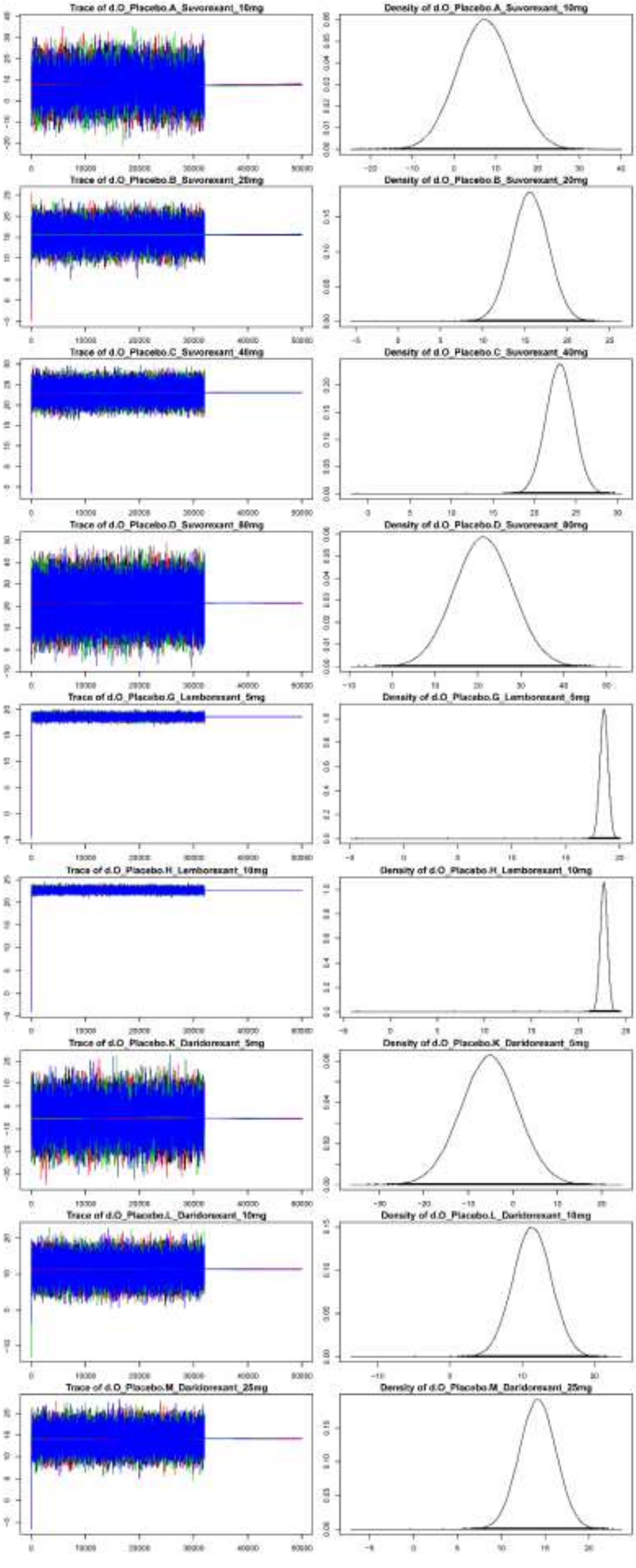

eFigure 17: Trace and density of the network meta-analysis: ISI.

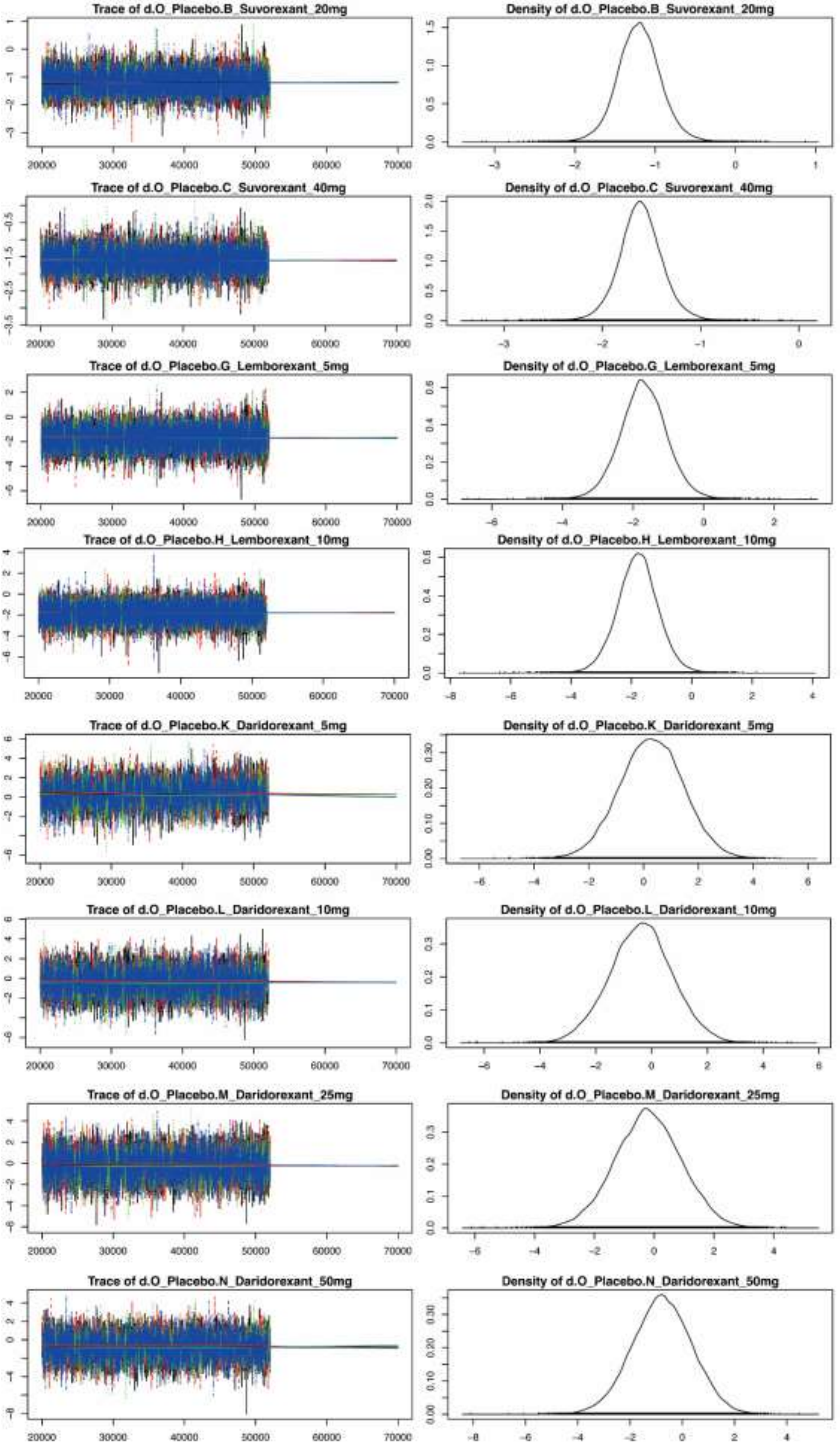

eFigure 18: Trace and density of the network meta-analysis: AEs.

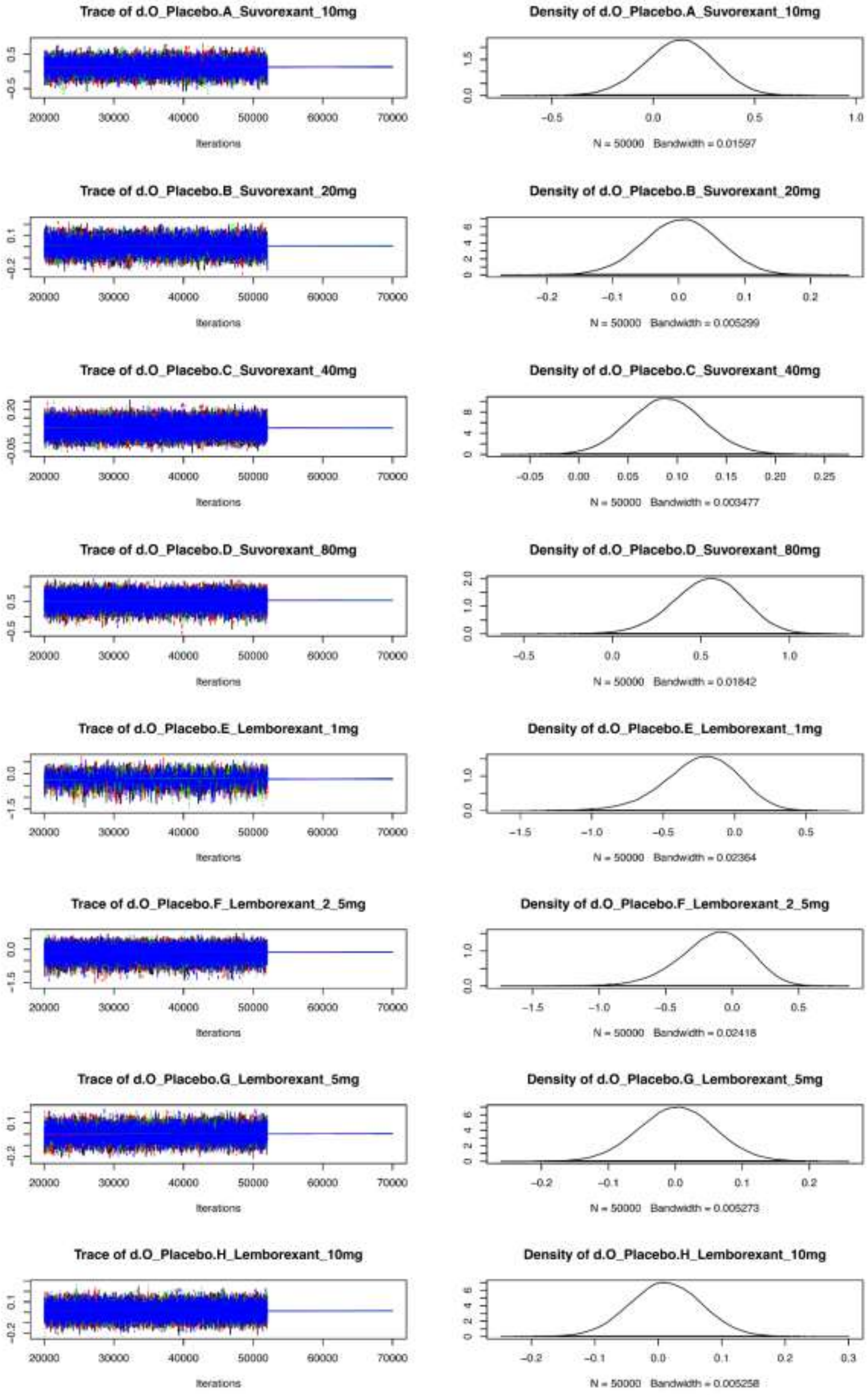

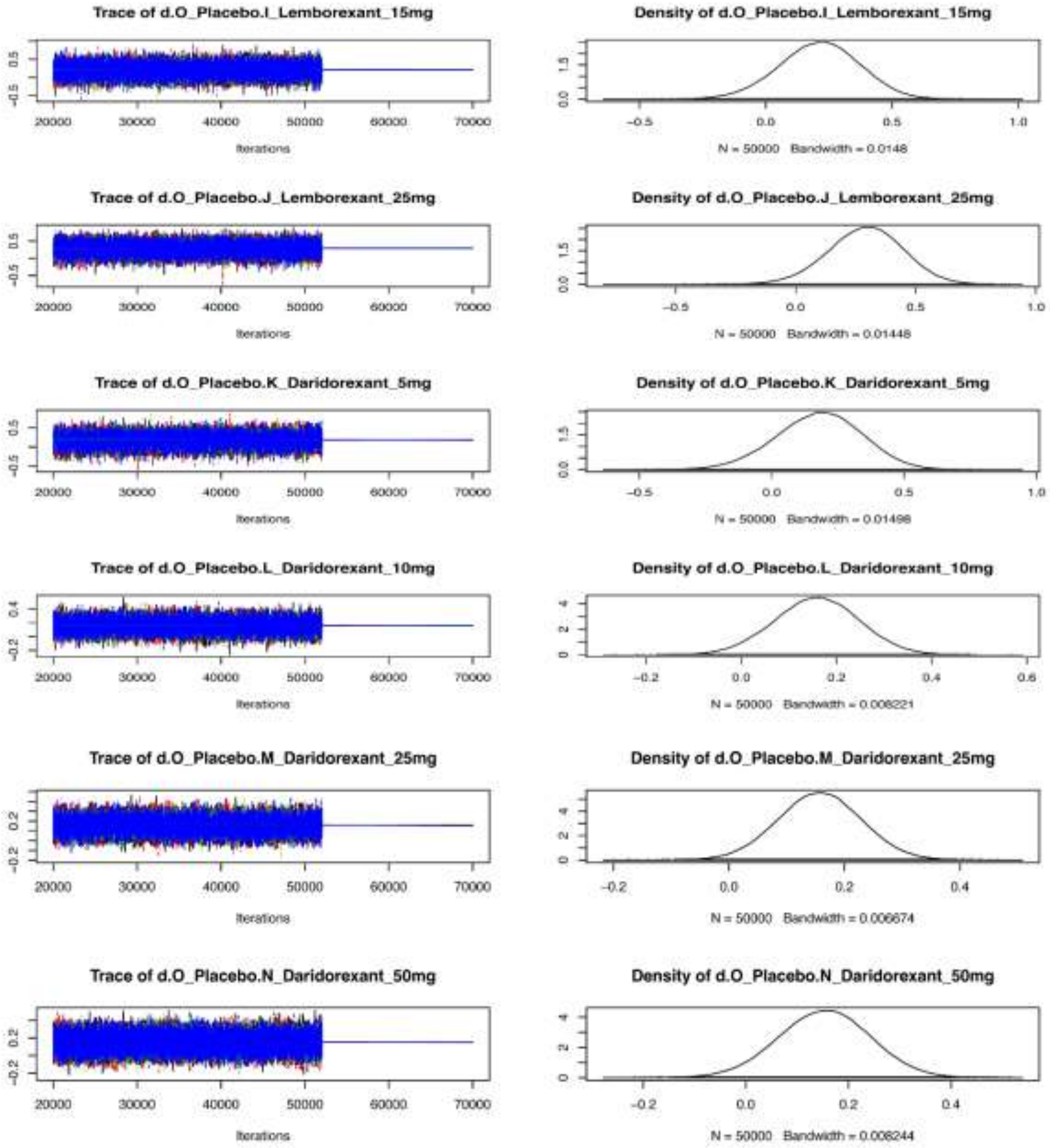
Trace of d.O_Placebo.I_Lemborexant_15mg
Iterations
Density of d.O_Placebo.I_Lemborexant_15mg
N = 50000 Bandwidth = 0.0148
Trace of d.O_Placebo.J_Lemborexant_25mg
Iterations
Density of d.O_Placebo.J_Lemborexant_25mg
N = 50000 Bandwidth = 0.01448
Trace of d.O_Placebo.K_Daridorexant_5mg
Iterations
Density of d.O_Placebo.K_Daridorexant_5mg
N = 50000 Bandwidth = 0.01498
Trace of d.O_Placebo.L_Daridorexant_10mg
Iterations
Density of d.O_Placebo.L_Daridorexant_10mg
N = 50000 Bandwidth = 0.008221
Trace of d.O_Placebo.M_Daridorexant_25mg
Iterations
Density of d.O_Placebo.M_Daridorexant_25mg
N = 50000 Bandwidth = 0.006674
Trace of d.O_Placebo.N_Daridorexant_50mg
Iterations
Density of d.O_Placebo.N_Daridorexant_50mg
N = 50000 Bandwidth = 0.008244

eFigure 19: Trace and density of the network meta-analysis: SAEs.

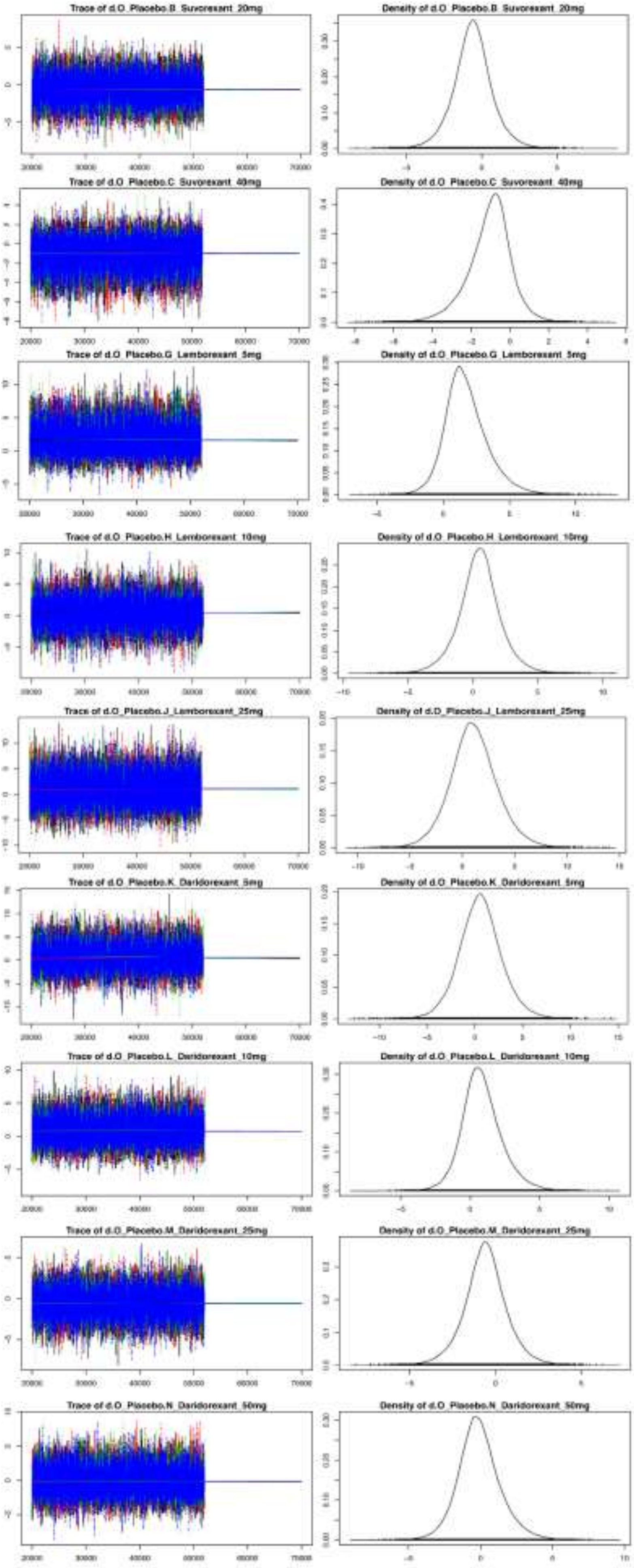

eFigure 20: Forest plots for the heterogeneity: LPS.

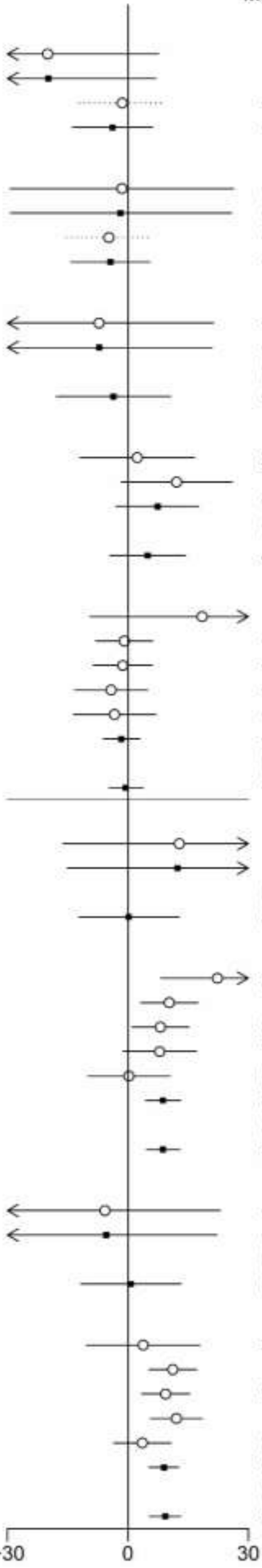

Study	I^2	Mean Difference (95% CrI)
**Suvorexant 20mg vs Suvorexant 10mg**		
2012 Herring		−20. (−48., 7.6)
Pooled (pair−wise)		−20. (−48., 6.9)
Indirect (back−calculated)		−1.4 (−12., 9.3)
Pooled (network)	33.8%	−3.8 (−14., 6.1)
**Suvorexant 40mg vs Suvorexant 10mg**		
2012 Herring		−1.5 (−29., 26.)
Pooled (pair−wise)		−1.9 (−29., 26.)
Indirect (back−calculated)		−4.7 (−15., 5.8)
Pooled (network)	0.0%	−4.4 (−14., 5.5)
**Suvorexant 80mg vs Suvorexant 10mg**		
2012 Herring		−7.2 (−36., 21.)
Pooled (pair−wise)		−7.1 (−35., 21.)
Indirect (back−calculated)		NA
Pooled (network)		−3.6 (−18., 11.)
**Placebo vs Suvorexant 10mg**		
2012 Herring		2.3 (−12., 17.)
2019 Herring		12. (−1.7, 26.)
Pooled (pair−wise)	0.0%	7.4 (−3.0, 18.)
Indirect (back−calculated)		NA
Pooled (network)	15.1%	4.9 (−4.5, 14.)
**Suvorexant 40mg vs Suvorexant 20mg**		
2012 Herring		19. (−9.5, 46.)
2016 Herring 1 (1 month)		−0.90 (−8., 6.2)
2016 Herring 1 (3 month)		−1.3 (−8.6, 6.0)
2016 Herring 2 (1 month)		−4.2 (−13., 4.9)
2016 Herring 2 (3 month)		−3.3 (−14., 6.9)
Pooled (pair−wise)	0.0%	−1.6 (−6.1, 3.)
Indirect (back−calculated)		NA
Pooled (network)	0.0%	−0.57 (−4.7, 3.8)
**Suvorexant 80mg vs Suvorexant 20mg**		
2012 Herring		13. (−16., 42.)
Pooled (pair−wise)		12. (−15., 41.)
Indirect (back−calculated)		NA
Pooled (network)		0.19 (−12., 13.)
**Placebo vs Suvorexant 20mg**		
2012 Herring		22. (8.2, 36.)
2016 Herring 1 (1 month)		10. (3.2, 17.)
2016 Herring 1 (3 month)		8.1 (1.0, 15.)
2016 Herring 2 (1 month)		7.9 (−1.3, 17.)
2016 Herring 2 (3 month)		0.27 (−10., 10.)
Pooled (pair−wise)	37.7%	8.7 (4.4, 13.)
Indirect (back−calculated)		NA
Pooled (network)	37.7%	8.7 (4.7, 13.)
**Suvorexant 80mg vs Suvorexant 40mg**		
2012 Herring		−5.7 (−34., 23.)
Pooled (pair−wise)		−5.4 (−33., 22.)
Indirect (back−calculated)		NA
Pooled (network)		0.73 (−12., 13.)
**Placebo vs Suvorexant 40mg**		
2012 Herring		3.8 (−10., 18.)
2016 Herring 1 (1 month)		11. (5.3, 17.)
2016 Herring 1 (3 month)		9.4 (3.4, 15.)
2016 Herring 2 (1 month)		12. (5.6, 19.)
2016 Herring 2 (3 month)		3.6 (−3.5, 11.)
Pooled (pair−wise)	4.2%	9.0 (5.3, 13.)
Indirect (back−calculated)		NA
Pooled (network)	4.3%	9.3 (5.4, 13.)

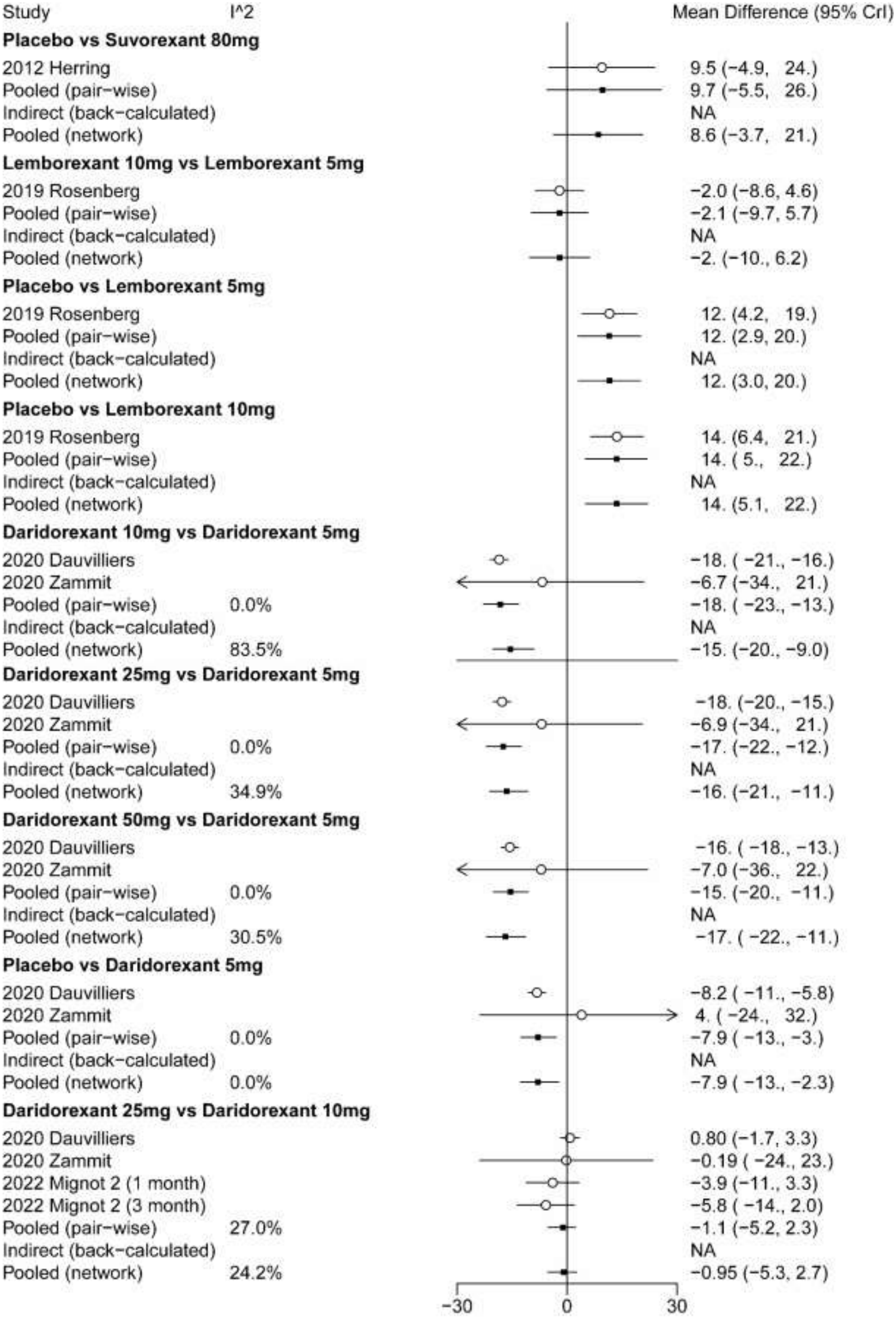
Study
I^2
Mean Difference (95% CrI)
Placebo vs Suvorexant 80mg
2012 Herring
9.5 (−4.9, 24.)
Pooled (pair−wise)
9.7 (−5.5, 26.)
Indirect (back−calculated)
NA
Pooled (network)
8.6 (−3.7, 21.)
Lemborexant 10mg vs Lemborexant 5mg
2019 Rosenberg
−2.0 (−8.6, 4.6)
Pooled (pair−wise)
−2.1 (−9.7, 5.7)
Indirect (back−calculated)
NA
Pooled (network)
−2. (−10., 6.2)
Placebo vs Lemborexant 5mg
2019 Rosenberg
12. (4.2, 19.)
Pooled (pair−wise)
12. (2.9, 20.)
Indirect (back−calculated)
NA
Pooled (network)
12. (3.0, 20.)
Placebo vs Lemborexant 10mg
2019 Rosenberg
14. (6.4, 21.)
Pooled (pair−wise)
14. (5., 22.)
Indirect (back−calculated)
NA
Pooled (network)
14. (5.1, 22.)
Daridorexant 10mg vs Daridorexant 5mg
2020 Dauvilliers
−18. (−21., −16.)
2020 Zammit
−6.7 (−34., 21.)
Pooled (pair−wise) 0.0%
−18. (−23., −13.)
Indirect (back−calculated)
NA
Pooled (network) 83.5%
−15. (−20., −9.0)
Daridorexant 25mg vs Daridorexant 5mg
2020 Dauvilliers
−18. (−20., −15.)
2020 Zammit
−6.9 (−34., 21.)
Pooled (pair−wise) 0.0%
−17. (−22., −12.)
Indirect (back−calculated)
NA
Pooled (network) 34.9%
−16. (−21., −11.)
Daridorexant 50mg vs Daridorexant 5mg
2020 Dauvilliers
−16. (−18., −13.)
2020 Zammit
−7.0 (−36., 22.)
Pooled (pair−wise) 0.0%
−15. (−20., −11.)
Indirect (back−calculated)
NA
Pooled (network) 30.5%
−17. (−22., −11.)
Placebo vs Daridorexant 5mg
2020 Dauvilliers
−8.2 (−11., −5.8)
2020 Zammit
4. (−24., 32.)
Pooled (pair−wise) 0.0%
−7.9 (−13., −3.)
Indirect (back−calculated)
NA
Pooled (network) 0.0%
−7.9 (−13., −2.3)
Daridorexant 25mg vs Daridorexant 10mg
2020 Dauvilliers
0.80 (−1.7, 3.3)
2020 Zammit
−0.19 (−24., 23.)
2022 Mignot 2 (1 month)
−3.9 (−11., 3.3)
2022 Mignot 2 (3 month)
−5.8 (−14., 2.0)
Pooled (pair−wise) 27.0%
−1.1 (−5.2, 2.3)
Indirect (back−calculated)
NA
Pooled (network) 24.2%
−0.95 (−5.3, 2.7)
−30
0
30

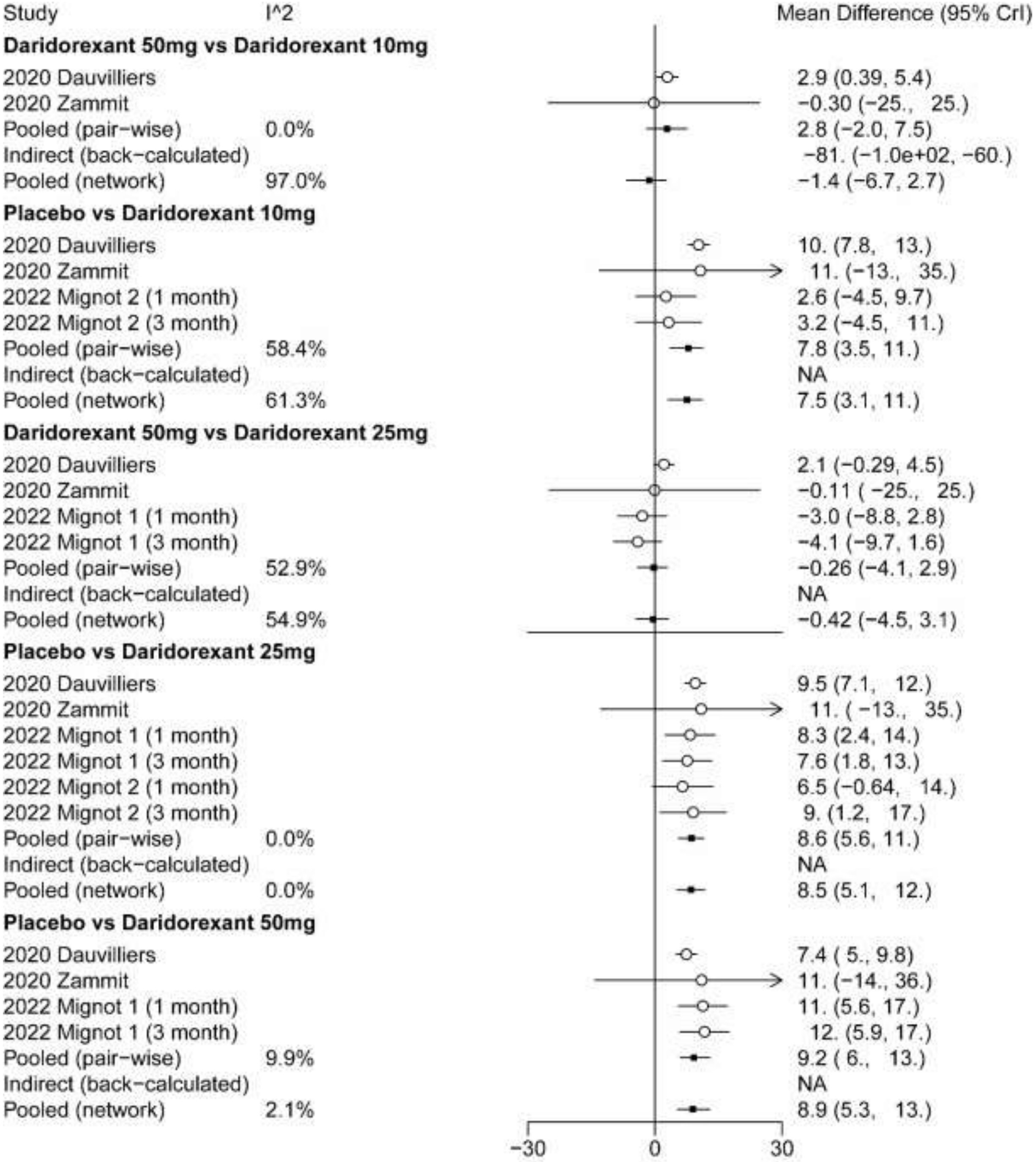
Study
I^2
Mean Difference (95% CrI)
Daridorexant 50mg vs Daridorexant 10mg
2020 Dauvilliers
2.9 (0.39, 5.4)
2020 Zammit
−0.30 (−25., 25.)
Pooled (pair−wise)
0.0%
2.8 (−2.0, 7.5)
Indirect (back−calculated)
−81. (−1.0e+02, −60.)
Pooled (network)
97.0%
−1.4 (−6.7, 2.7)
Placebo vs Daridorexant 10mg
2020 Dauvilliers
10. (7.8, 13.)
2020 Zammit
11. (−13., 35.)
2022 Mignot 2 (1 month)
2.6 (−4.5, 9.7)
2022 Mignot 2 (3 month)
3.2 (−4.5, 11.)
Pooled (pair−wise)
58.4%
7.8 (3.5, 11.)
Indirect (back−calculated)
NA
Pooled (network)
61.3%
7.5 (3.1, 11.)
Daridorexant 50mg vs Daridorexant 25mg
2020 Dauvilliers
2.1 (−0.29, 4.5)
2020 Zammit
−0.11 (−25., 25.)
2022 Mignot 1 (1 month)
−3.0 (−8.8, 2.8)
2022 Mignot 1 (3 month)
−4.1 (−9.7, 1.6)
Pooled (pair−wise)
52.9%
−0.26 (−4.1, 2.9)
Indirect (back−calculated)
NA
Pooled (network)
54.9%
−0.42 (−4.5, 3.1)
Placebo vs Daridorexant 25mg
2020 Dauvilliers
9.5 (7.1, 12.)
2020 Zammit
11. (−13., 35.)
2022 Mignot 1 (1 month)
8.3 (2.4, 14.)
2022 Mignot 1 (3 month)
7.6 (1.8, 13.)
2022 Mignot 2 (1 month)
6.5 (−0.64, 14.)
2022 Mignot 2 (3 month)
9. (1.2, 17.)
Pooled (pair−wise)
0.0%
8.6 (5.6, 11.)
Indirect (back−calculated)
NA
Pooled (network)
0.0%
8.5 (5.1, 12.)
Placebo vs Daridorexant 50mg
2020 Dauvilliers
7.4 (5., 9.8)
2020 Zammit
11. (−14., 36.)
2022 Mignot 1 (1 month)
11. (5.6, 17.)
2022 Mignot 1 (3 month)
12. (5.9, 17.)
Pooled (pair−wise)
9.9%
9.2 (6., 13.)
Indirect (back−calculated)
NA
Pooled (network)
2.1%
8.9 (5.3, 13.)
−30
0
30

eFigure 21: Forest plots for the heterogeneity: sTSO.

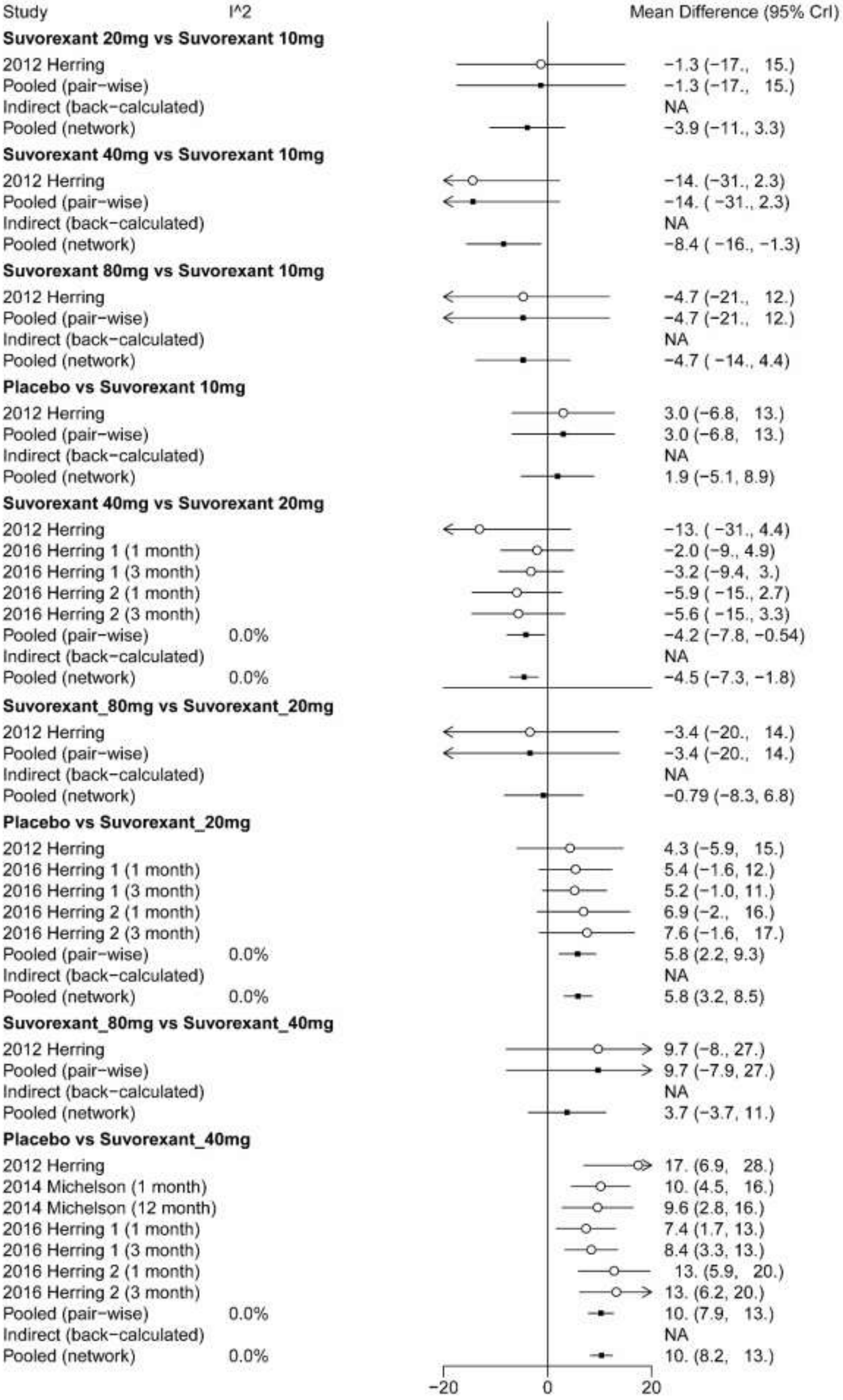

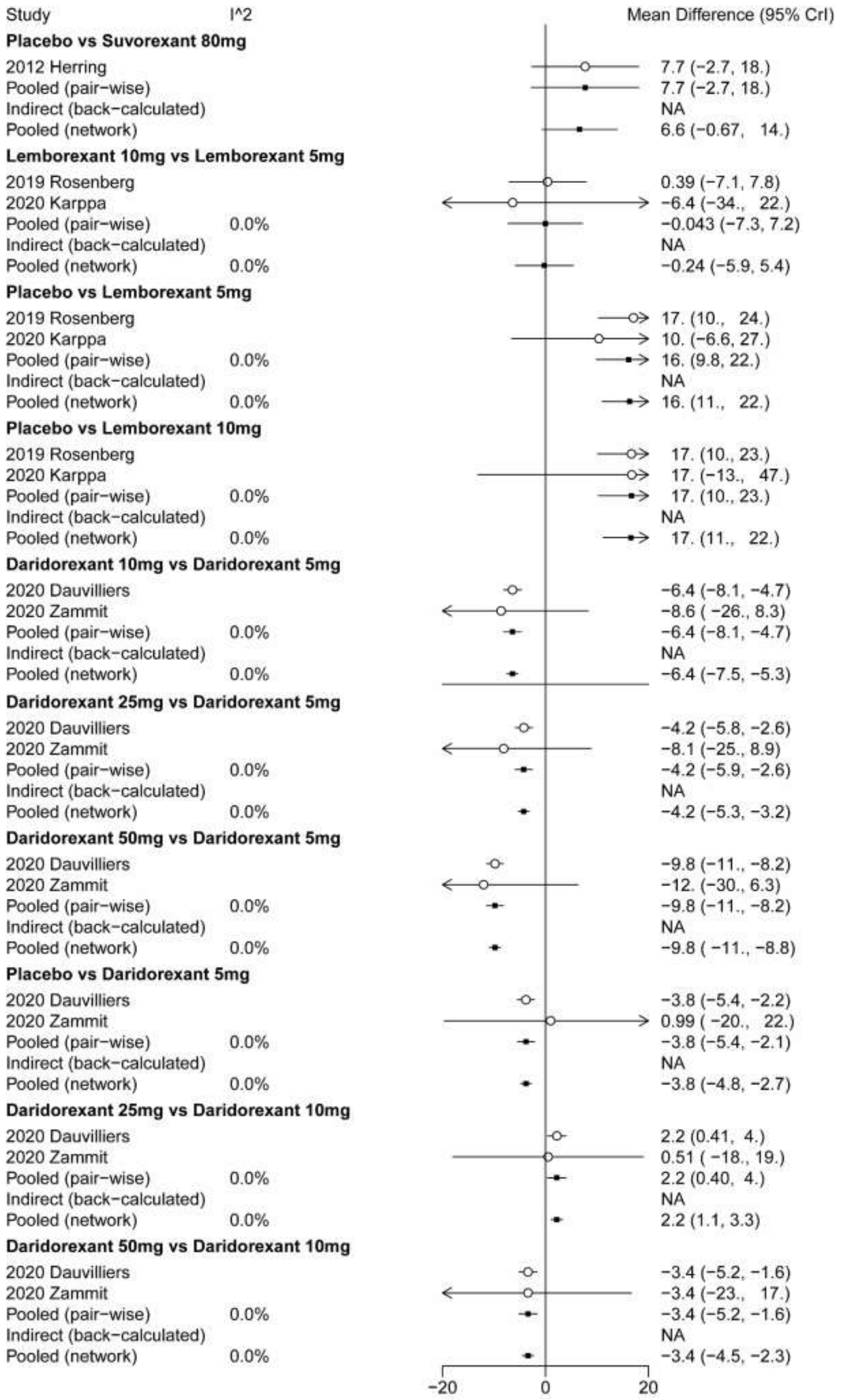
Study I^2 Mean Difference (95% CrI)
Placebo vs Suvorexant 80mg
2012 Herring 7.7 (−2.7, 18.)
Pooled (pair−wise) 7.7 (−2.7, 18.)
Indirect (back−calculated) NA
Pooled (network) 6.6 (−0.67, 14.)
Lemborexant 10mg vs Lemborexant 5mg
2019 Rosenberg 0.39 (−7.1, 7.8)
2020 Karppa −6.4 (−34., 22.)
Pooled (pair−wise) 0.0% −0.043 (−7.3, 7.2)
Indirect (back−calculated) NA
Pooled (network) 0.0% −0.24 (−5.9, 5.4)
Placebo vs Lemborexant 5mg
2019 Rosenberg 17. (10., 24.)
2020 Karppa 10. (−6.6, 27.)
Pooled (pair−wise) 0.0% 16. (9.8, 22.)
Indirect (back−calculated) NA
Pooled (network) 0.0% 16. (11., 22.)
Placebo vs Lemborexant 10mg
2019 Rosenberg 17. (10., 23.)
2020 Karppa 17. (−13., 47.)
Pooled (pair−wise) 0.0% 17. (10., 23.)
Indirect (back−calculated) NA
Pooled (network) 0.0% 17. (11., 22.)
Daridorexant 10mg vs Daridorexant 5mg
2020 Dauvilliers −6.4 (−8.1, −4.7)
2020 Zammit −8.6 (−26., 8.3)
Pooled (pair−wise) 0.0% −6.4 (−8.1, −4.7)
Indirect (back−calculated) NA
Pooled (network) 0.0% −6.4 (−7.5, −5.3)
Daridorexant 25mg vs Daridorexant 5mg
2020 Dauvilliers −4.2 (−5.8, −2.6)
2020 Zammit −8.1 (−25., 8.9)
Pooled (pair−wise) 0.0% −4.2 (−5.9, −2.6)
Indirect (back−calculated) NA
Pooled (network) 0.0% −4.2 (−5.3, −3.2)
Daridorexant 50mg vs Daridorexant 5mg
2020 Dauvilliers −9.8 (−11., −8.2)
2020 Zammit −12. (−30., 6.3)
Pooled (pair−wise) 0.0% −9.8 (−11., −8.2)
Indirect (back−calculated) NA
Pooled (network) 0.0% −9.8 (−11., −8.8)
Placebo vs Daridorexant 5mg
2020 Dauvilliers −3.8 (−5.4, −2.2)
2020 Zammit 0.99 (−20., 22.)
Pooled (pair−wise) 0.0% −3.8 (−5.4, −2.1)
Indirect (back−calculated) NA
Pooled (network) 0.0% −3.8 (−4.8, −2.7)
Daridorexant 25mg vs Daridorexant 10mg
2020 Dauvilliers 2.2 (0.41, 4.)
2020 Zammit 0.51 (−18., 19.)
Pooled (pair−wise) 0.0% 2.2 (0.40, 4.)
Indirect (back−calculated) NA
Pooled (network) 0.0% 2.2 (1.1, 3.3)
Daridorexant 50mg vs Daridorexant 10mg
2020 Dauvilliers −3.4 (−5.2, −1.6)
2020 Zammit −3.4 (−23., 17.)
Pooled (pair−wise) 0.0% −3.4 (−5.2, −1.6)
Indirect (back−calculated) NA
Pooled (network) 0.0% −3.4 (−4.5, −2.3)
−20 0 20

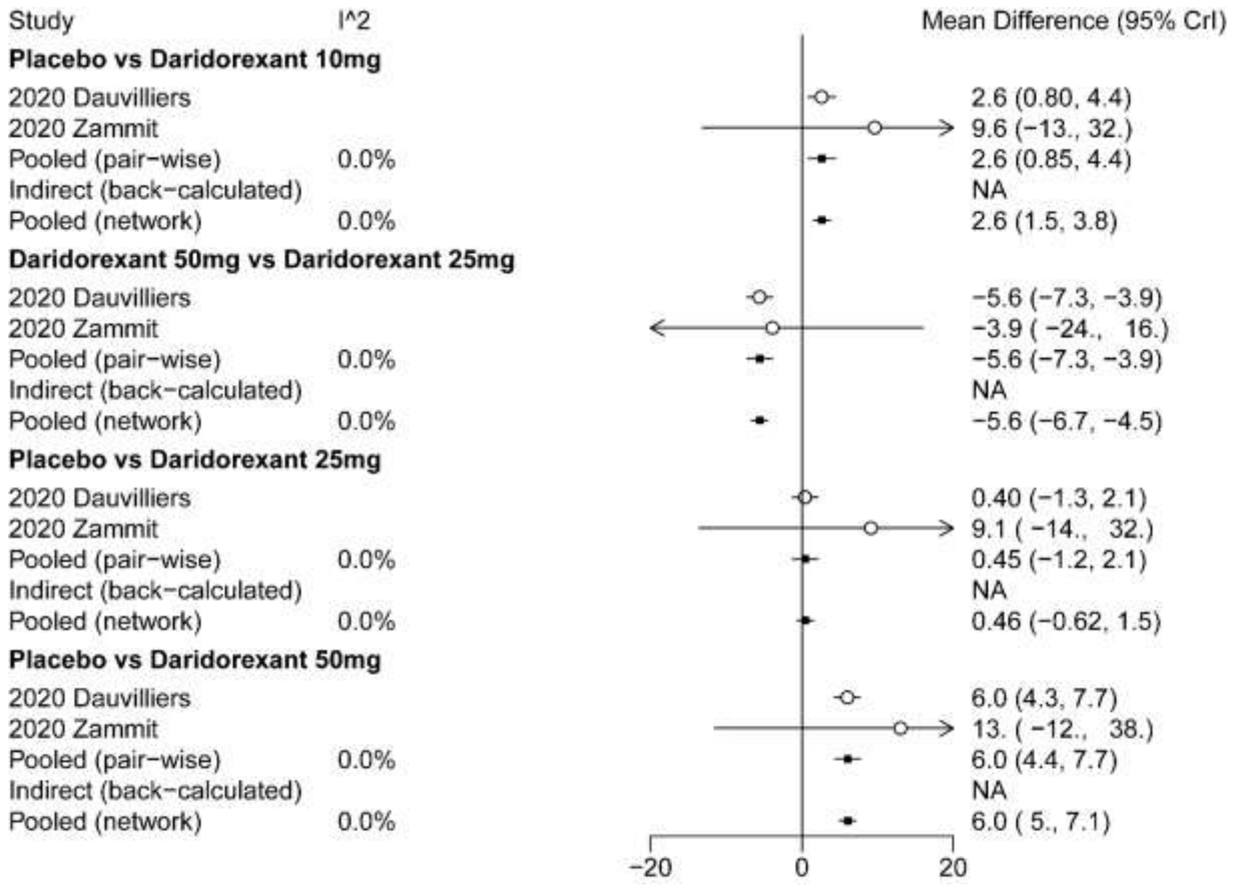
Study
I^2
Mean Difference (95% CrI)
Placebo vs Daridorexant 10mg
2020 Dauvilliers
2.6 (0.80, 4.4)
2020 Zammit
9.6 (−13., 32.)
Pooled (pair−wise)
0.0%
2.6 (0.85, 4.4)
Indirect (back−calculated)
NA
Pooled (network)
0.0%
2.6 (1.5, 3.8)
Daridorexant 50mg vs Daridorexant 25mg
2020 Dauvilliers
−5.6 (−7.3, −3.9)
2020 Zammit
−3.9 (−24., 16.)
Pooled (pair−wise)
0.0%
−5.6 (−7.3, −3.9)
Indirect (back−calculated)
NA
Pooled (network)
0.0%
−5.6 (−6.7, −4.5)
Placebo vs Daridorexant 25mg
2020 Dauvilliers
0.40 (−1.3, 2.1)
2020 Zammit
9.1 (−14., 32.)
Pooled (pair−wise)
0.0%
0.45 (−1.2, 2.1)
Indirect (back−calculated)
NA
Pooled (network)
0.0%
0.46 (−0.62, 1.5)
Placebo vs Daridorexant 50mg
2020 Dauvilliers
6.0 (4.3, 7.7)
2020 Zammit
13. (−12., 38.)
Pooled (pair−wise)
0.0%
6.0 (4.4, 7.7)
Indirect (back−calculated)
NA
Pooled (network)
0.0%
6.0 (5., 7.1)
−20
0
20

eFigure 22: Forest plots for the heterogeneity: WASO.

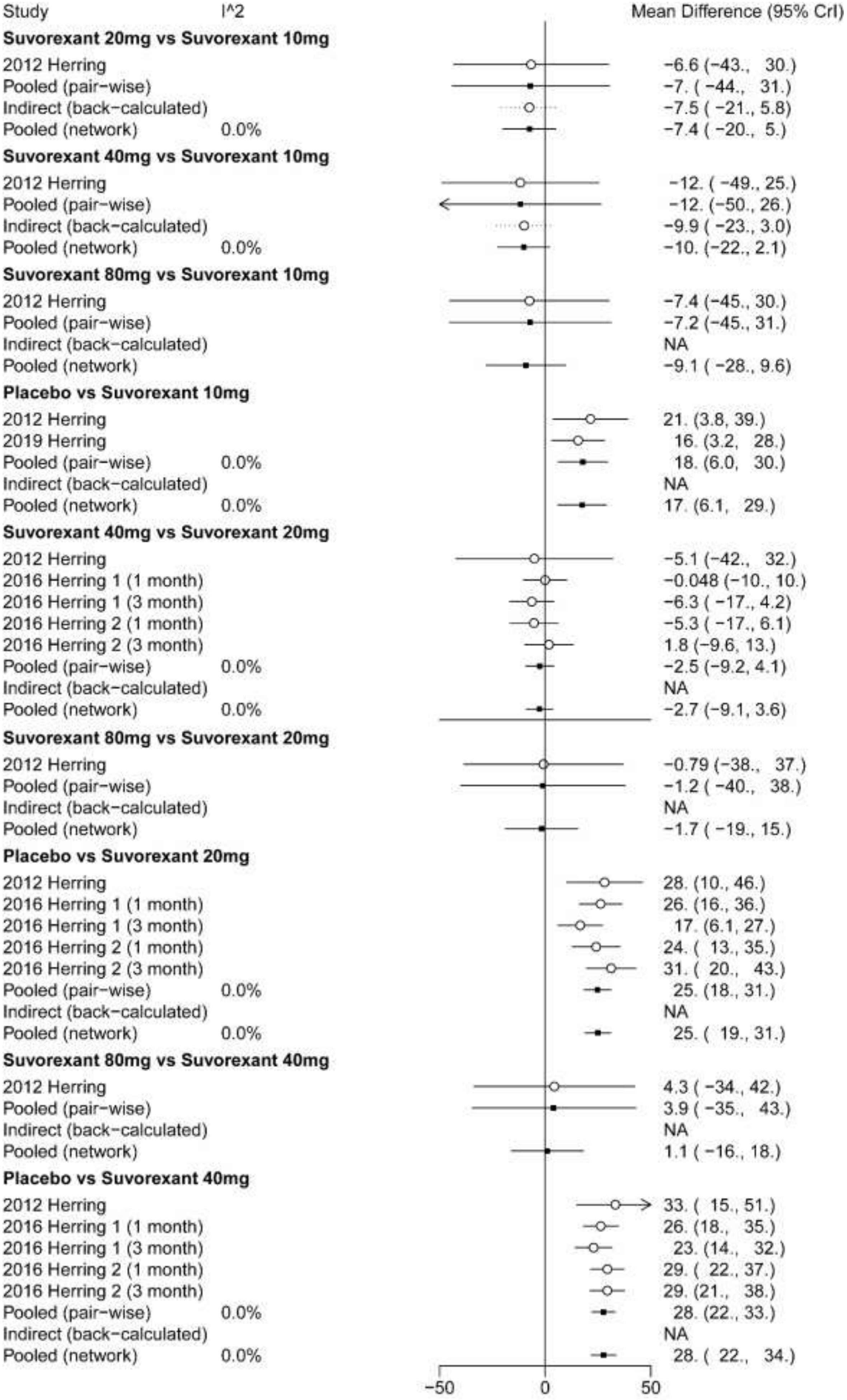

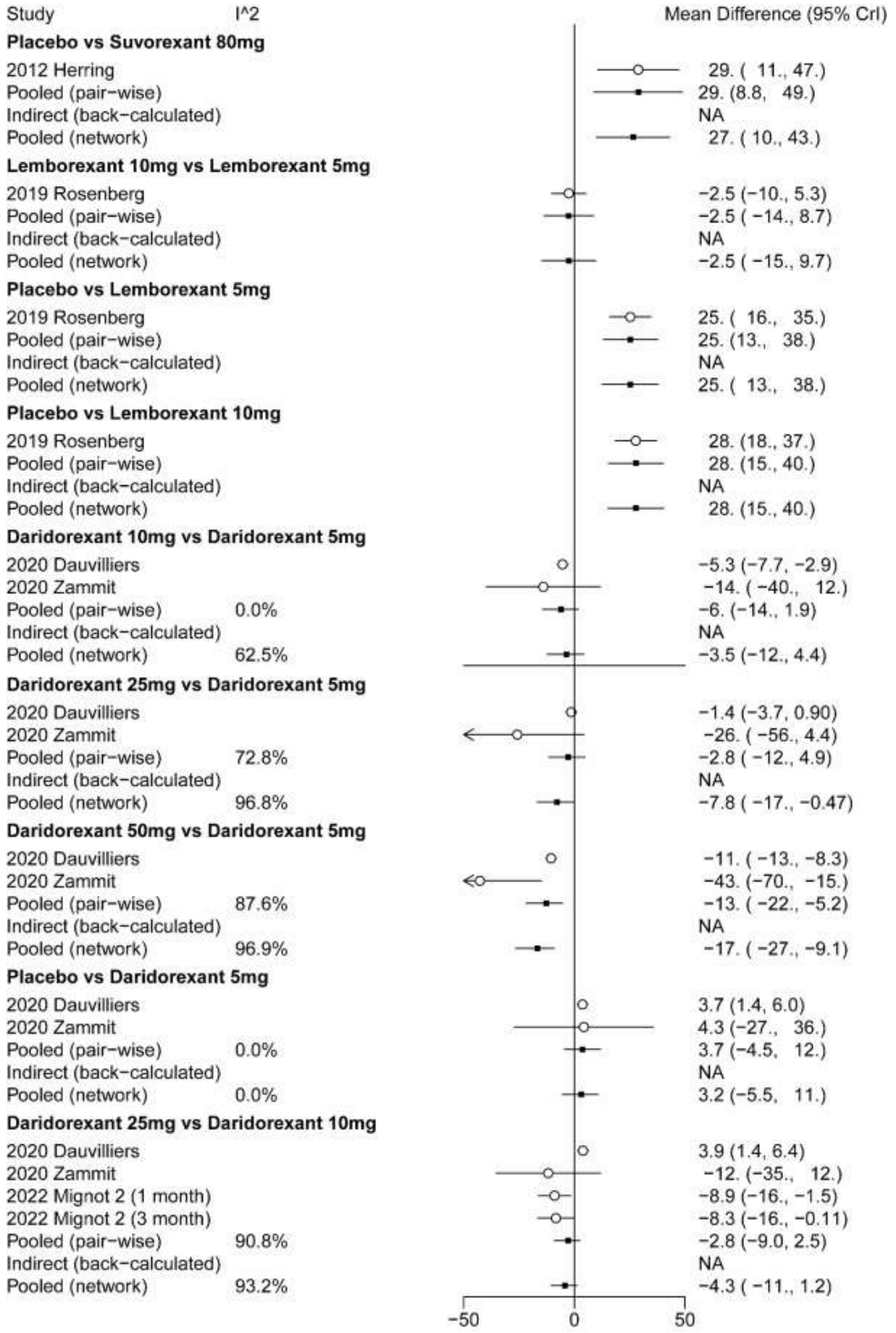
Study
I^2
Mean Difference (95% Crl)
Placebo vs Suvorexant 80mg
2012 Herring 29. (11., 47.)
Pooled (pair−wise) 29. (8.8, 49.)
Indirect (back−calculated) NA
Pooled (network) 27. (10., 43.)
Lemborexant 10mg vs Lemborexant 5mg
2019 Rosenberg −2.5 (−10., 5.3)
Pooled (pair−wise) −2.5 (−14., 8.7)
Indirect (back−calculated) NA
Pooled (network) −2.5 (−15., 9.7)
Placebo vs Lemborexant 5mg
2019 Rosenberg 25. (16., 35.)
Pooled (pair−wise) 25. (13., 38.)
Indirect (back−calculated) NA
Pooled (network) 25. (13., 38.)
Placebo vs Lemborexant 10mg
2019 Rosenberg 28. (18., 37.)
Pooled (pair−wise) 28. (15., 40.)
Indirect (back−calculated) NA
Pooled (network) 28. (15., 40.)
Daridorexant 10mg vs Daridorexant 5mg
2020 Dauvilliers −5.3 (−7.7, −2.9)
2020 Zammit −14. (−40., 12.)
Pooled (pair−wise) 0.0% −6. (−14., 1.9)
Indirect (back−calculated) NA
Pooled (network) 62.5% −3.5 (−12., 4.4)
Daridorexant 25mg vs Daridorexant 5mg
2020 Dauvilliers −1.4 (−3.7, 0.90)
2020 Zammit −26. (−56., 4.4)
Pooled (pair−wise) 72.8% −2.8 (−12., 4.9)
Indirect (back−calculated) NA
Pooled (network) 96.8% −7.8 (−17., −0.47)
Daridorexant 50mg vs Daridorexant 5mg
2020 Dauvilliers −11. (−13., −8.3)
2020 Zammit −43. (−70., −15.)
Pooled (pair−wise) 87.6% −13. (−22., −5.2)
Indirect (back−calculated) NA
Pooled (network) 96.9% −17. (−27., −9.1)
Placebo vs Daridorexant 5mg
2020 Dauvilliers 3.7 (1.4, 6.0)
2020 Zammit 4.3 (−27., 36.)
Pooled (pair−wise) 0.0% 3.7 (−4.5, 12.)
Indirect (back−calculated) NA
Pooled (network) 0.0% 3.2 (−5.5, 11.)
Daridorexant 25mg vs Daridorexant 10mg
2020 Dauvilliers 3.9 (1.4, 6.4)
2020 Zammit −12. (−35., 12.)
2022 Mignot 2 (1 month) −8.9 (−16., −1.5)
2022 Mignot 2 (3 month) −8.3 (−16., −0.11)
Pooled (pair−wise) 90.8% −2.8 (−9.0, 2.5)
Indirect (back−calculated) NA
Pooled (network) 93.2% −4.3 (−11., 1.2)
−50
0
50

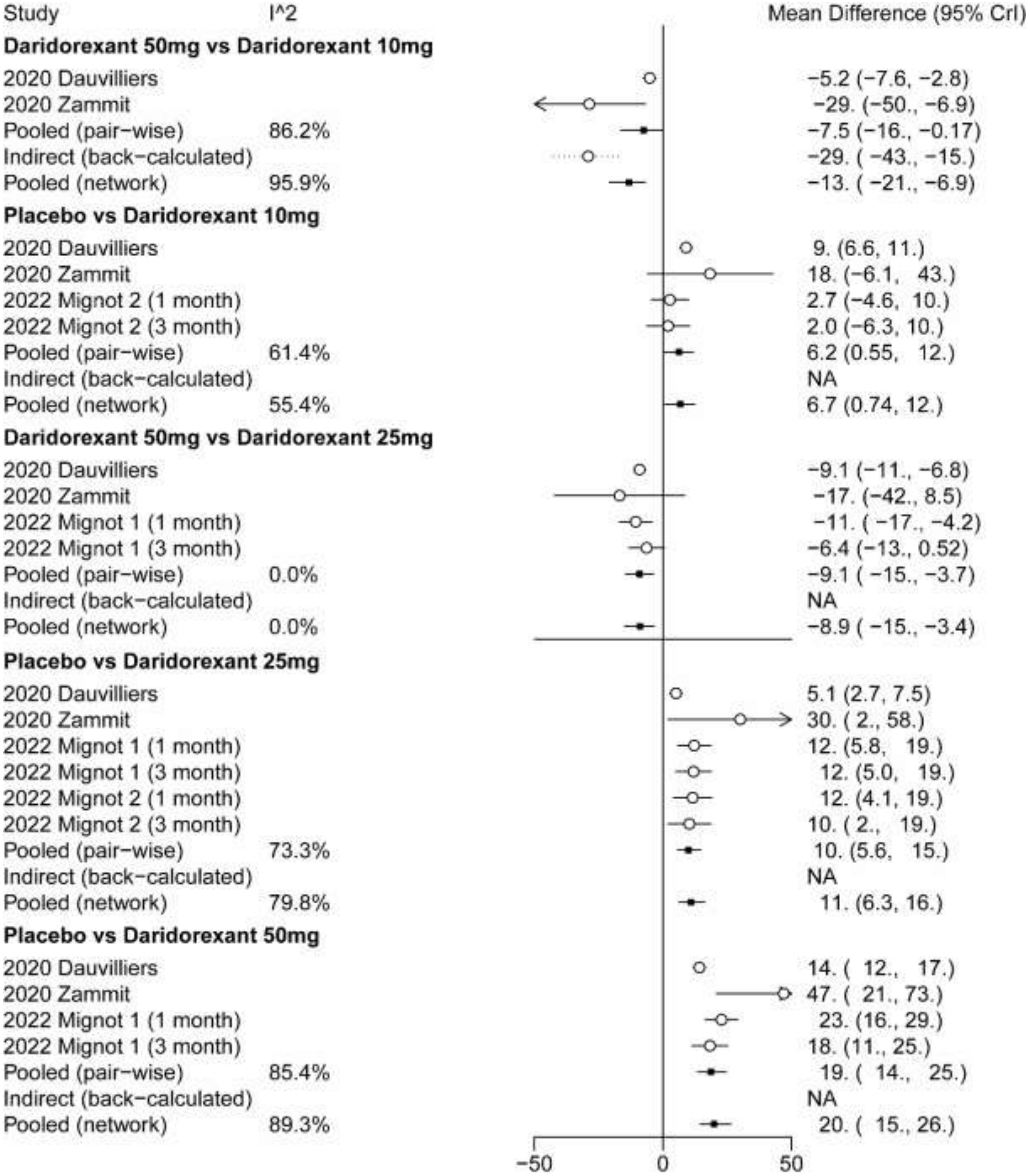

Study
I^2
Mean Difference (95% CrI)
Daridorexant 50mg vs Daridorexant 10mg
2020 Dauvilliers −5.2 (−7.6, −2.8)
2020 Zammit −29. (−50., −6.9)
Pooled (pair−wise) 86.2% −7.5 (−16., −0.17)
Indirect (back−calculated) −29. (−43., −15.)
Pooled (network) 95.9% −13. (−21., −6.9)
Placebo vs Daridorexant 10mg
2020 Dauvilliers 9. (6.6, 11.)
2020 Zammit 18. (−6.1, 43.)
2022 Mignot 2 (1 month) 2.7 (−4.6, 10.)
2022 Mignot 2 (3 month) 2.0 (−6.3, 10.)
Pooled (pair−wise) 61.4% 6.2 (0.55, 12.)
Indirect (back−calculated) NA
Pooled (network) 55.4% 6.7 (0.74, 12.)
Daridorexant 50mg vs Daridorexant 25mg
2020 Dauvilliers −9.1 (−11., −6.8)
2020 Zammit −17. (−42., 8.5)
2022 Mignot 1 (1 month) −11. (−17., −4.2)
2022 Mignot 1 (3 month) −6.4 (−13., 0.52)
Pooled (pair−wise) 0.0% −9.1 (−15., −3.7)
Indirect (back−calculated) NA
Pooled (network) 0.0% −8.9 (−15., −3.4)
Placebo vs Daridorexant 25mg
2020 Dauvilliers 5.1 (2.7, 7.5)
2020 Zammit 30. (2., 58.)
2022 Mignot 1 (1 month) 12. (5.8, 19.)
2022 Mignot 1 (3 month) 12. (5.0, 19.)
2022 Mignot 2 (1 month) 12. (4.1, 19.)
2022 Mignot 2 (3 month) 10. (2., 19.)
Pooled (pair−wise) 73.3% 10. (5.6, 15.)
Indirect (back−calculated) NA
Pooled (network) 79.8% 11. (6.3, 16.)
Placebo vs Daridorexant 50mg
2020 Dauvilliers 14. (12., 17.)
2020 Zammit 47. (21., 73.)
2022 Mignot 1 (1 month) 23. (16., 29.)
2022 Mignot 1 (3 month) 18. (11., 25.)
Pooled (pair−wise) 85.4% 19. (14., 25.)
Indirect (back−calculated) NA
Pooled (network) 89.3% 20. (15., 26.)
−50
0
50

eFigure 23: Forest plots for the heterogeneity: sWASO.

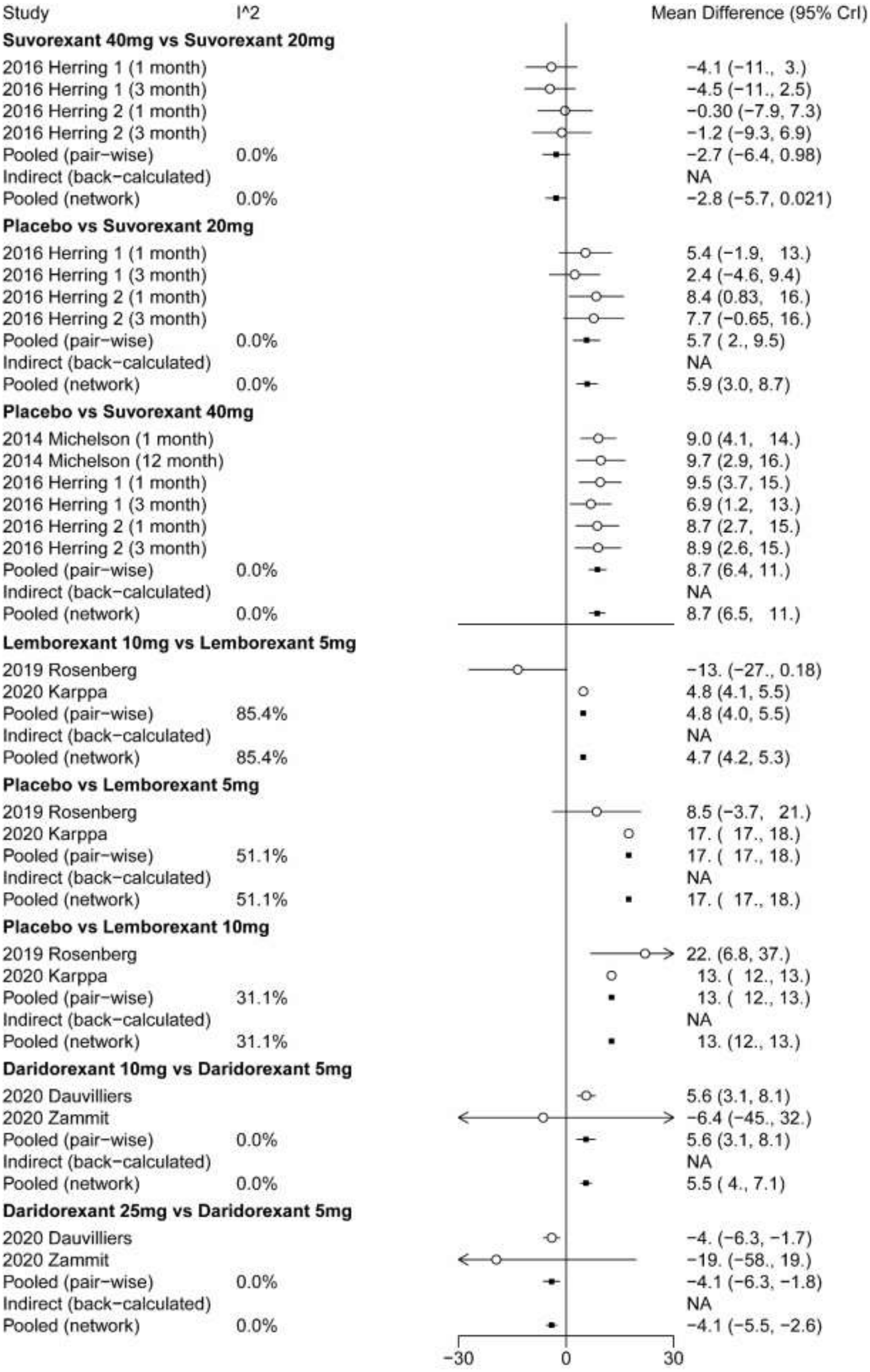

Study	I^2	Mean Difference (95% CrI)
**Daridorexant 50mg vs Daridorexant 5mg**		
2020 Dauvilliers		−7.9 (−10., −5.5)
2020 Zammit		−19. (−61., 23.)
Pooled (pair−wise)	0.0%	−7.9 (−10., −5.5)
Indirect (back−calculated)		NA
Pooled (network)	0.0%	−7.9 (−9.5, −6.4)
**Placebo vs Daridorexant 5mg**		
2020 Dauvilliers		3.7 (1.4, 6.0)
2020 Zammit		8. (−35., 51.)
Pooled (pair−wise)	0.0%	3.7 (1.4, 6.0)
Indirect (back−calculated)		NA
Pooled (network)	0.0%	3.7 (2.2, 5.2)
**Daridorexant 25mg vs Daridorexant 10mg**		
2020 Dauvilliers		−9.6 (−12., −7.2)
2020 Zammit		−13. (−48., 22.)
Pooled (pair−wise)	0.0%	−9.6 (−12., −7.2)
Indirect (back−calculated)		NA
Pooled (network)	0.0%	−9.6 (−11., −8.1)
**Daridorexant 50mg vs Daridorexant 10mg**		
2020 Dauvilliers		−14. (−16., −11.)
2020 Zammit		−12. (−51., 26.)
Pooled (pair−wise)	0.0%	−14. (−16., −11.)
Indirect (back−calculated)		NA
Pooled (network)	0.0%	−13. (−15., −12.)
**Placebo vs Daridorexant 10mg**		
2020 Dauvilliers		−1.9 (−4.4, 0.60)
2020 Zammit		14. (−25., 54.)
Pooled (pair−wise)	0.0%	−1.8 (−4.3, 0.66)
Indirect (back−calculated)		NA
Pooled (network)	0.0%	−1.8 (−3.4, −0.29)
**Daridorexant 50mg vs Daridorexant 25mg**		
2020 Dauvilliers		−3.9 (−6.2, −1.6)
2020 Zammit		0.64 (−37., 39.)
Pooled (pair−wise)	0.0%	−3.9 (−6.2, −1.5)
Indirect (back−calculated)		NA
Pooled (network)	0.0%	−3.9 (−5.4, −2.4)
**Placebo vs Daridorexant 25mg**		
2020 Dauvilliers		7.7 (5.4, 10.)
2020 Zammit		27. (−12., 67.)
Pooled (pair−wise)	0.0%	7.8 (5.5, 10.)
Indirect (back−calculated)		NA
Pooled (network)	0.0%	7.8 (6.3, 9.2)
**Placebo vs Daridorexant 50mg**		
2020 Dauvilliers		12. (9.2, 14.)
2020 Zammit		27. (−16., 70.)
Pooled (pair−wise)	0.0%	12. (9.2, 14.)
Indirect (back−calculated)		NA
Pooled (network)	0.0%	12. (10., 13.)

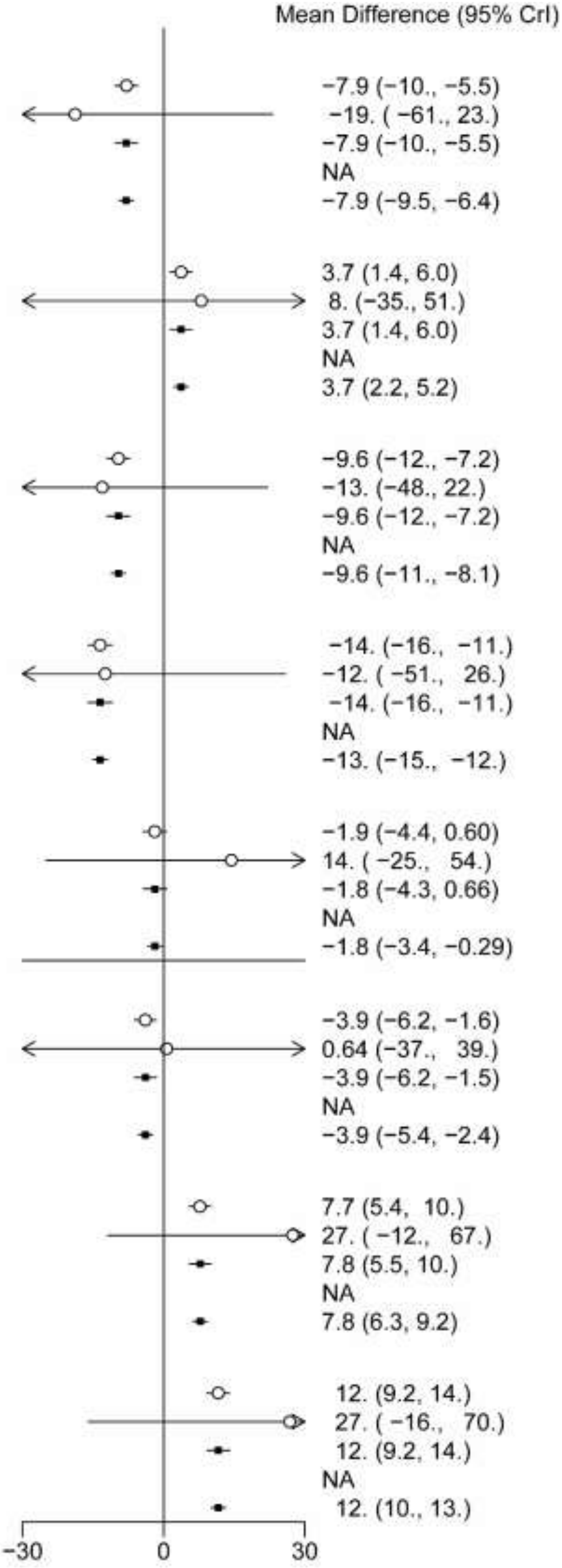

eFigure 24: Forest plots for the heterogeneity: TST.

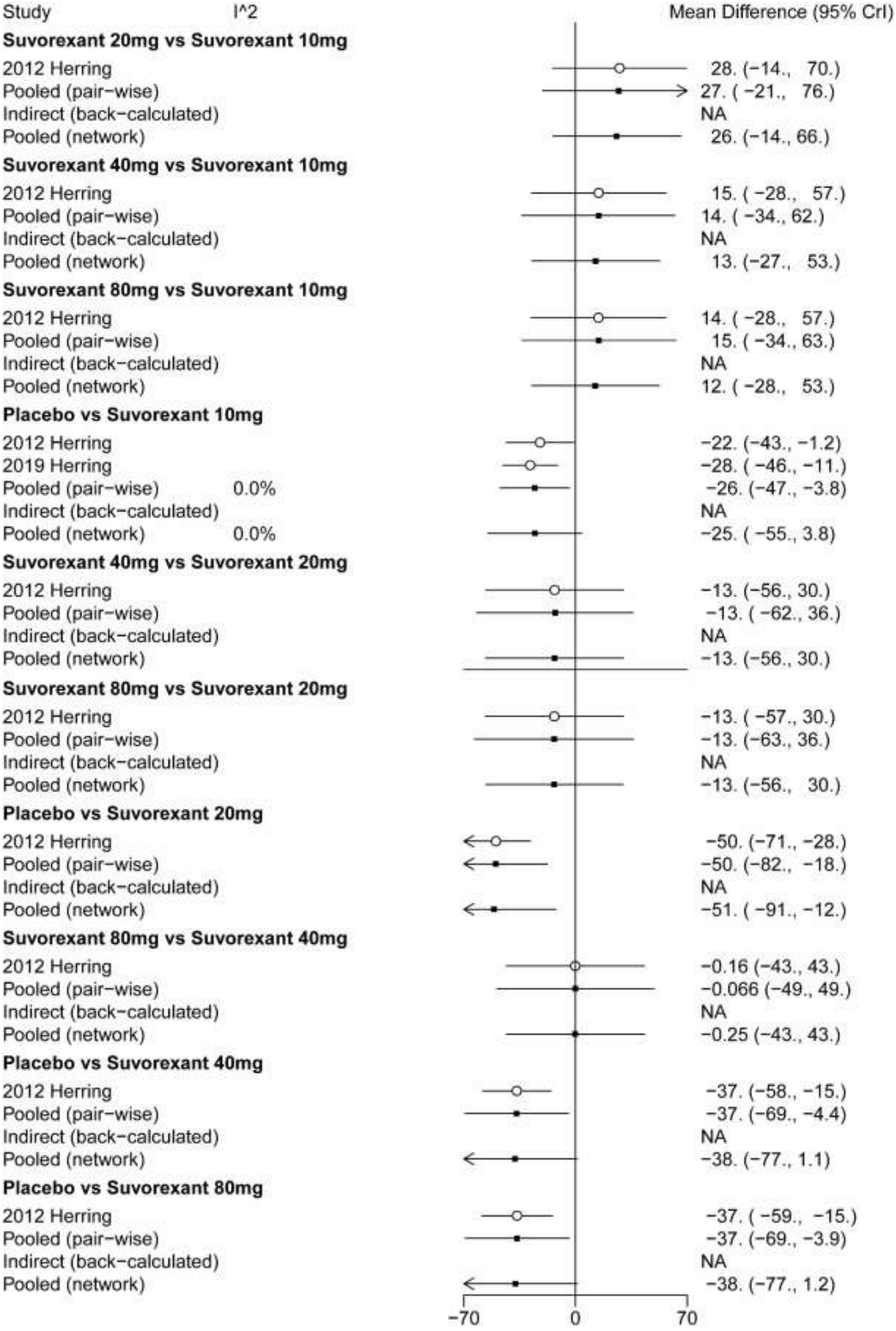

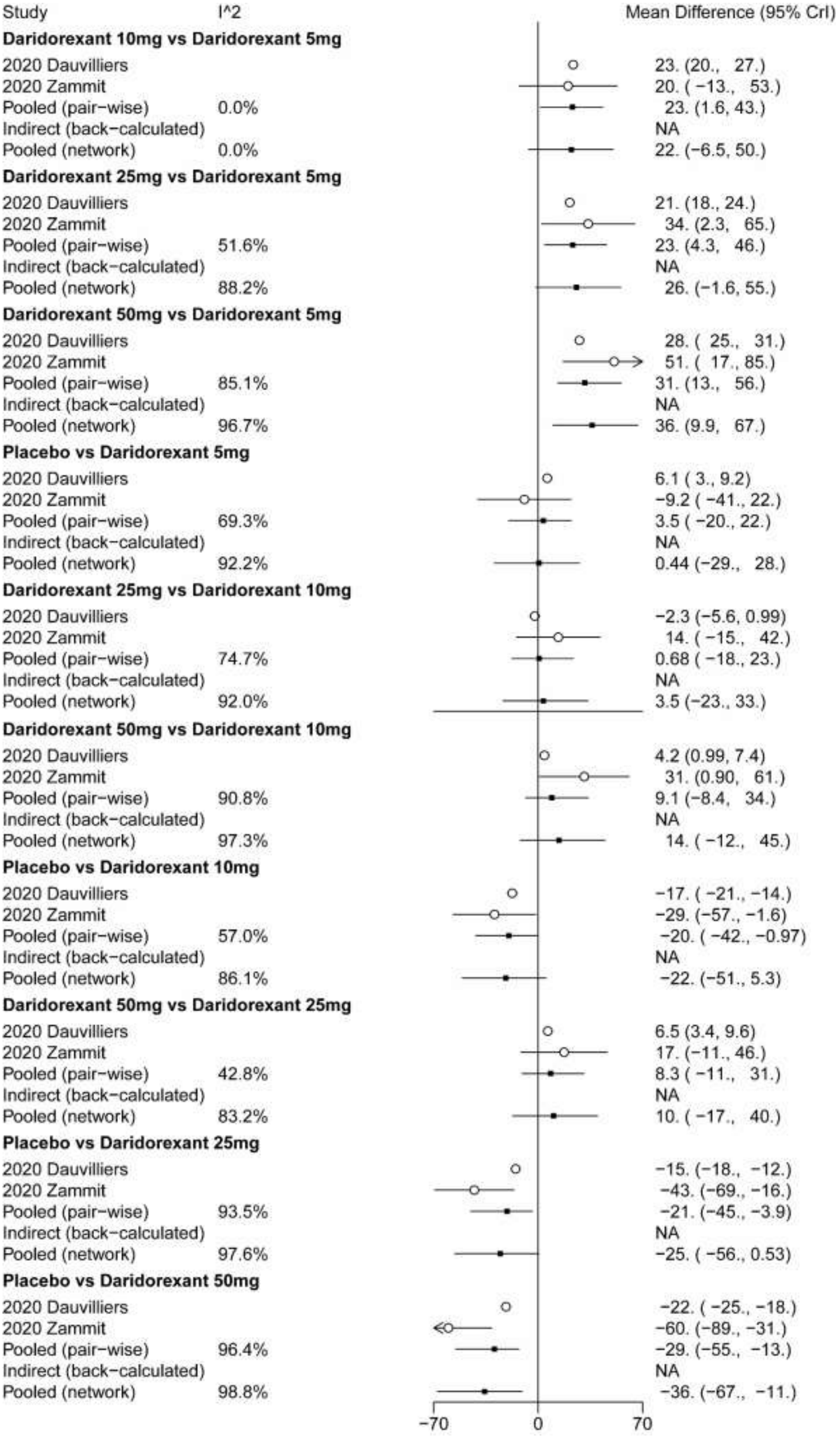
Study
I^2
Mean Difference (95% CrI)
Daridorexant 10mg vs Daridorexant 5mg
2020 Dauvilliers 23. (20., 27.)
2020 Zammit 20. (−13., 53.)
Pooled (pair−wise) 0.0% 23. (1.6, 43.)
Indirect (back−calculated) NA
Pooled (network) 0.0% 22. (−6.5, 50.)
Daridorexant 25mg vs Daridorexant 5mg
2020 Dauvilliers 21. (18., 24.)
2020 Zammit 34. (2.3, 65.)
Pooled (pair−wise) 51.6% 23. (4.3, 46.)
Indirect (back−calculated) NA
Pooled (network) 88.2% 26. (−1.6, 55.)
Daridorexant 50mg vs Daridorexant 5mg
2020 Dauvilliers 28. (25., 31.)
2020 Zammit 51. (17., 85.)
Pooled (pair−wise) 85.1% 31. (13., 56.)
Indirect (back−calculated) NA
Pooled (network) 96.7% 36. (9.9, 67.)
Placebo vs Daridorexant 5mg
2020 Dauvilliers 6.1 (3., 9.2)
2020 Zammit −9.2 (−41., 22.)
Pooled (pair−wise) 69.3% 3.5 (−20., 22.)
Indirect (back−calculated) NA
Pooled (network) 92.2% 0.44 (−29., 28.)
Daridorexant 25mg vs Daridorexant 10mg
2020 Dauvilliers −2.3 (−5.6, 0.99)
2020 Zammit 14. (−15., 42.)
Pooled (pair−wise) 74.7% 0.68 (−18., 23.)
Indirect (back−calculated) NA
Pooled (network) 92.0% 3.5 (−23., 33.)
Daridorexant 50mg vs Daridorexant 10mg
2020 Dauvilliers 4.2 (0.99, 7.4)
2020 Zammit 31. (0.90, 61.)
Pooled (pair−wise) 90.8% 9.1 (−8.4, 34.)
Indirect (back−calculated) NA
Pooled (network) 97.3% 14. (−12., 45.)
Placebo vs Daridorexant 10mg
2020 Dauvilliers −17. (−21., −14.)
2020 Zammit −29. (−57., −1.6)
Pooled (pair−wise) 57.0% −20. (−42., −0.97)
Indirect (back−calculated) NA
Pooled (network) 86.1% −22. (−51., 5.3)
Daridorexant 50mg vs Daridorexant 25mg
2020 Dauvilliers 6.5 (3.4, 9.6)
2020 Zammit 17. (−11., 46.)
Pooled (pair−wise) 42.8% 8.3 (−11., 31.)
Indirect (back−calculated) NA
Pooled (network) 83.2% 10. (−17., 40.)
Placebo vs Daridorexant 25mg
2020 Dauvilliers −15. (−18., −12.)
2020 Zammit −43. (−69., −16.)
Pooled (pair−wise) 93.5% −21. (−45., −3.9)
Indirect (back−calculated) NA
Pooled (network) 97.6% −25. (−56., 0.53)
Placebo vs Daridorexant 50mg
2020 Dauvilliers −22. (−25., −18.)
2020 Zammit −60. (−89., −31.)
Pooled (pair−wise) 96.4% −29. (−55., −13.)
Indirect (back−calculated) NA
Pooled (network) 98.8% −36. (−67., −11.)
−70
0
70

eFigure 25: Forest plots for the heterogeneity: sTST.

Study	I^2	Mean Difference (95% CrI)
**Suvorexant 20mg vs Suvorexant 10mg**		
2012 Herring		−7.3 (−40., 26.)
Pooled (pair−wise)		−7.4 (−40., 26.)
Indirect (back−calculated)		NA
Pooled (network)		8.1 (−5.1, 21.)
**Suvorexant 40mg vs Suvorexant 10mg**		
2012 Herring		24. (−9.3, 57.)
Pooled (pair−wise)		24. (−9.4, 58.)
Indirect (back−calculated)		NA
Pooled (network)		16. (2.5, 29.)
**Suvorexant 80mg vs Suvorexant 10mg**		
2012 Herring		14. (−20., 48.)
Pooled (pair−wise)		14. (−20., 48.)
Indirect (back−calculated)		NA
Pooled (network)		14. (−3.3, 31.)
**Placebo vs Suvorexant 10mg**		
2012 Herring		−5.5 (−22., 11.)
Pooled (pair−wise)		−5.5 (−22., 11.)
Indirect (back−calculated)		NA
Pooled (network)		−7.5 (−20., 5.5)
**Suvorexant 40mg vs Suvorexant 20mg**		
2012 Herring		31. (−3.3, 66.)
2016 Herring 1 (1 month)		3.2 (−7.4, 14.)
2016 Herring 1 (3 month)		9.1 (−1.9, 20.)
2016 Herring 2 (1 month)		5.3 (−6.4, 17.)
2016 Herring 2 (3 month)		3. (−10., 16.)
Pooled (pair−wise)	0.0%	6. (0.32, 12.)
Indirect (back−calculated)		NA
Pooled (network)	0.0%	7.5 (3.2, 12.)
**Suvorexant 80mg vs Suvorexant 20mg**		
2012 Herring		21. (−13., 55.)
Pooled (pair−wise)		21. (−13., 55.)
Indirect (back−calculated)		NA
Pooled (network)		5.7 (−7.9, 19.)
**Placebo vs Suvorexant 20mg**		
2012 Herring		1.8 (−15., 19.)
2016 Herring 1 (1 month)		−16. (−27., −5.4)
2016 Herring 1 (3 month)		−11. (−22., 0.65)
2016 Herring 2 (1 month)		−21. (−33., −9.0)
2016 Herring 2 (3 month)		−22. (−36., −8.6)
Pooled (pair−wise)	36.6%	−15. (−21., −9.4)
Indirect (back−calculated)		NA
Pooled (network)	37.0%	−16. (−20., −11.)
**Suvorexant 80mg vs Suvorexant 40mg**		
2012 Herring		−10. (−46., 25.)
Pooled (pair−wise)		−10. (−46., 25.)
Indirect (back−calculated)		NA
Pooled (network)		−1.7 (−15., 12.)
**Placebo vs Suvorexant 40mg**		
2012 Herring		−30. (−48., −12.)
2014 Michelson (1 month)		−23. (−31., −16.)
2014 Michelson (12 month)		−27. (−39., −16.)
2016 Herring 1 (1 month)		−20. (−28., −11.)
2016 Herring 1 (3 month)		−20. (−29., −11.)
2016 Herring 2 (1 month)		−26. (−35., −17.)
2016 Herring 2 (3 month)		−25. (−35., −15.)
Pooled (pair−wise)	0.0%	−23. (−27., −20.)
Indirect (back−calculated)		NA
Pooled (network)	0.0%	−23. (−26., −20.)

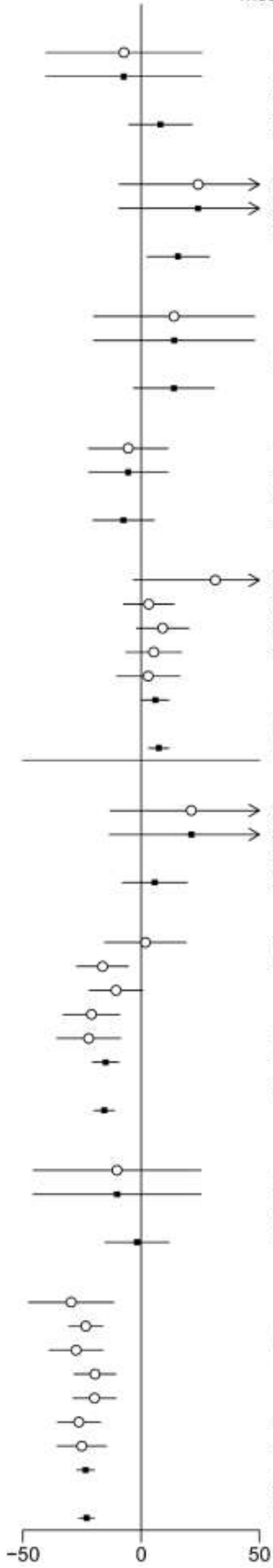

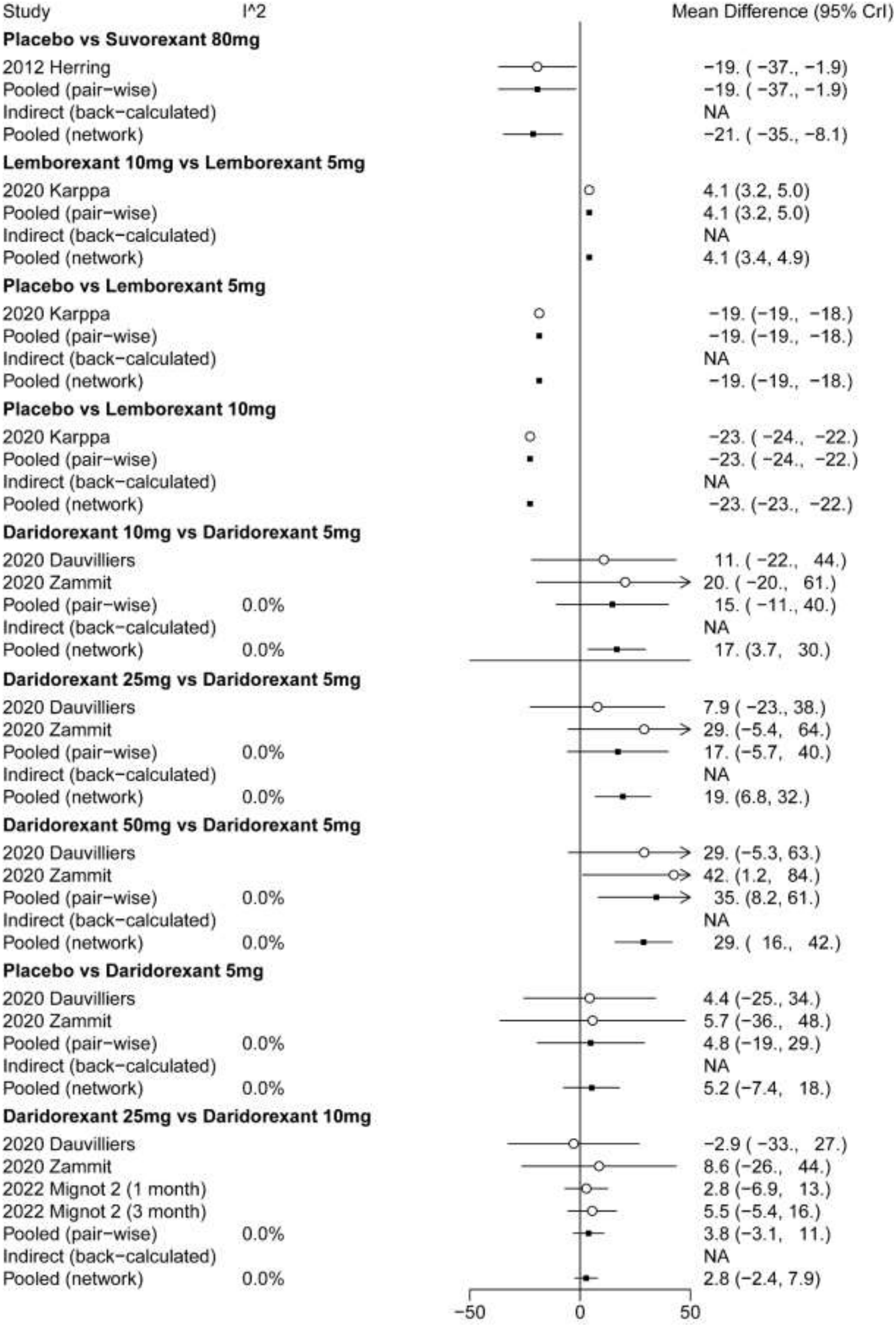
Study
I^2
Mean Difference (95% CrI)
Placebo vs Suvorexant 80mg
2012 Herring −19. (−37., −1.9)
Pooled (pair−wise) −19. (−37., −1.9)
Indirect (back−calculated) NA
Pooled (network) −21. (−35., −8.1)
Lemborexant 10mg vs Lemborexant 5mg
2020 Karppa 4.1 (3.2, 5.0)
Pooled (pair−wise) 4.1 (3.2, 5.0)
Indirect (back−calculated) NA
Pooled (network) 4.1 (3.4, 4.9)
Placebo vs Lemborexant 5mg
2020 Karppa −19. (−19., −18.)
Pooled (pair−wise) −19. (−19., −18.)
Indirect (back−calculated) NA
Pooled (network) −19. (−19., −18.)
Placebo vs Lemborexant 10mg
2020 Karppa −23. (−24., −22.)
Pooled (pair−wise) −23. (−24., −22.)
Indirect (back−calculated) NA
Pooled (network) −23. (−23., −22.)
Daridorexant 10mg vs Daridorexant 5mg
2020 Dauvilliers 11. (−22., 44.)
2020 Zammit 20. (−20., 61.)
Pooled (pair−wise) 0.0% 15. (−11., 40.)
Indirect (back−calculated) NA
Pooled (network) 0.0% 17. (3.7, 30.)
Daridorexant 25mg vs Daridorexant 5mg
2020 Dauvilliers 7.9 (−23., 38.)
2020 Zammit 29. (−5.4, 64.)
Pooled (pair−wise) 0.0% 17. (−5.7, 40.)
Indirect (back−calculated) NA
Pooled (network) 0.0% 19. (6.8, 32.)
Daridorexant 50mg vs Daridorexant 5mg
2020 Dauvilliers 29. (−5.3, 63.)
2020 Zammit 42. (1.2, 84.)
Pooled (pair−wise) 0.0% 35. (8.2, 61.)
Indirect (back−calculated) NA
Pooled (network) 0.0% 29. (16., 42.)
Placebo vs Daridorexant 5mg
2020 Dauvilliers 4.4 (−25., 34.)
2020 Zammit 5.7 (−36., 48.)
Pooled (pair−wise) 0.0% 4.8 (−19., 29.)
Indirect (back−calculated) NA
Pooled (network) 0.0% 5.2 (−7.4, 18.)
Daridorexant 25mg vs Daridorexant 10mg
2020 Dauvilliers −2.9 (−33., 27.)
2020 Zammit 8.6 (−26., 44.)
2022 Mignot 2 (1 month) 2.8 (−6.9, 13.)
2022 Mignot 2 (3 month) 5.5 (−5.4, 16.)
Pooled (pair−wise) 0.0% 3.8 (−3.1, 11.)
Indirect (back−calculated) NA
Pooled (network) 0.0% 2.8 (−2.4, 7.9)
−50
0
50

Study	I^2	Mean Difference (95% CrI)
**Daridorexant 50mg vs Daridorexant 10mg**		
2020 Dauvilliers		18. (−15., 52.)
2020 Zammit		22. (−20., 64.)
Pooled (pair−wise)	0.0%	20. (−6.5, 46.)
Indirect (back−calculated)		12. (4.8, 18.)
Pooled (network)	0.0%	12. (5.5, 19.)
**Placebo vs Daridorexant 10mg**		
2020 Dauvilliers		−6.4 (−36., 23.)
2020 Zammit		−15. (−57., 27.)
2022 Mignot 2 (1 month)		−13. (−23., −3.6)
2022 Mignot 2 (3 month)		−14. (−25., −2.7)
Pooled (pair−wise)	0.0%	−13. (−20., −6.1)
Indirect (back−calculated)		NA
Pooled (network)	0.0%	−11. (−17., −6.2)
**Daridorexant 50mg vs Daridorexant 25mg**		
2020 Dauvilliers		21. (−10., 53.)
2020 Zammit		13. (−22., 49.)
2022 Mignot 1 (1 month)		9.4 (−0.0033, 19.)
2022 Mignot 1 (3 month)		9.9 (−1.4, 21.)
Pooled (pair−wise)	0.0%	10. (3.4, 17.)
Indirect (back−calculated)		NA
Pooled (network)	0.0%	9.4 (4.2, 15.)
**Placebo vs Daridorexant 25mg**		
2020 Dauvilliers		−3.5 (−31., 24.)
2020 Zammit		−23. (−60., 13.)
2022 Mignot 1 (1 month)		−13. (−22., −3.1)
2022 Mignot 1 (3 month)		−9.9 (−21., 1.3)
2022 Mignot 2 (1 month)		−16. (−26., −6.4)
2022 Mignot 2 (3 month)		−19. (−30., −8.1)
Pooled (pair−wise)	0.0%	−14. (−19., −9.2)
Indirect (back−calculated)		NA
Pooled (network)	0.0%	−14. (−18., −10.)
**Placebo vs Daridorexant 50mg**		
2020 Dauvilliers		−25. (−55., 5.8)
2020 Zammit		−37. (−80., 6.4)
2022 Mignot 1 (1 month)		−22. (−31., −13.)
2022 Mignot 1 (3 month)		−20. (−31., −8.5)
Pooled (pair−wise)	0.0%	−22. (−29., −15.)
Indirect (back−calculated)		NA
Pooled (network)	0.0%	−24. (−29., −18.)

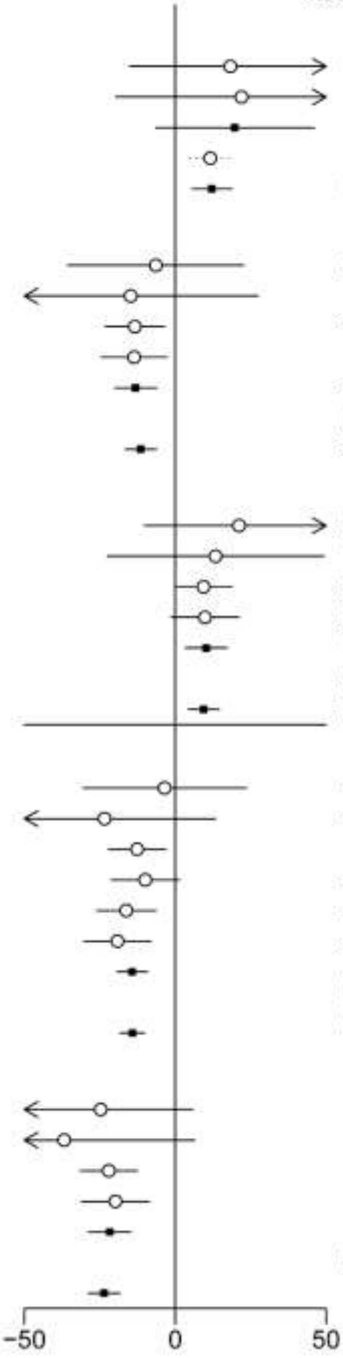

eFigure 26: Forest plots for the heterogeneity: ISI.

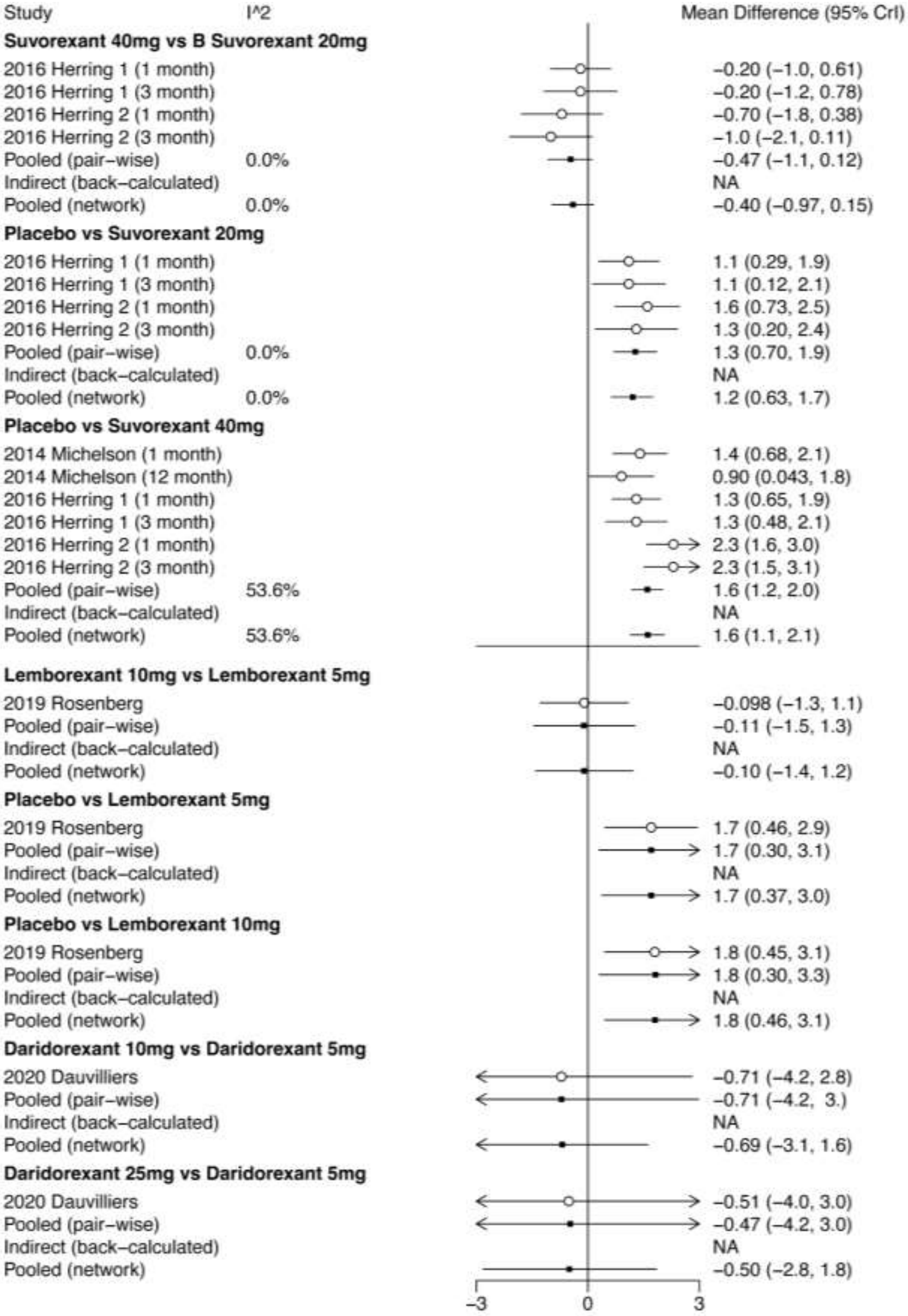

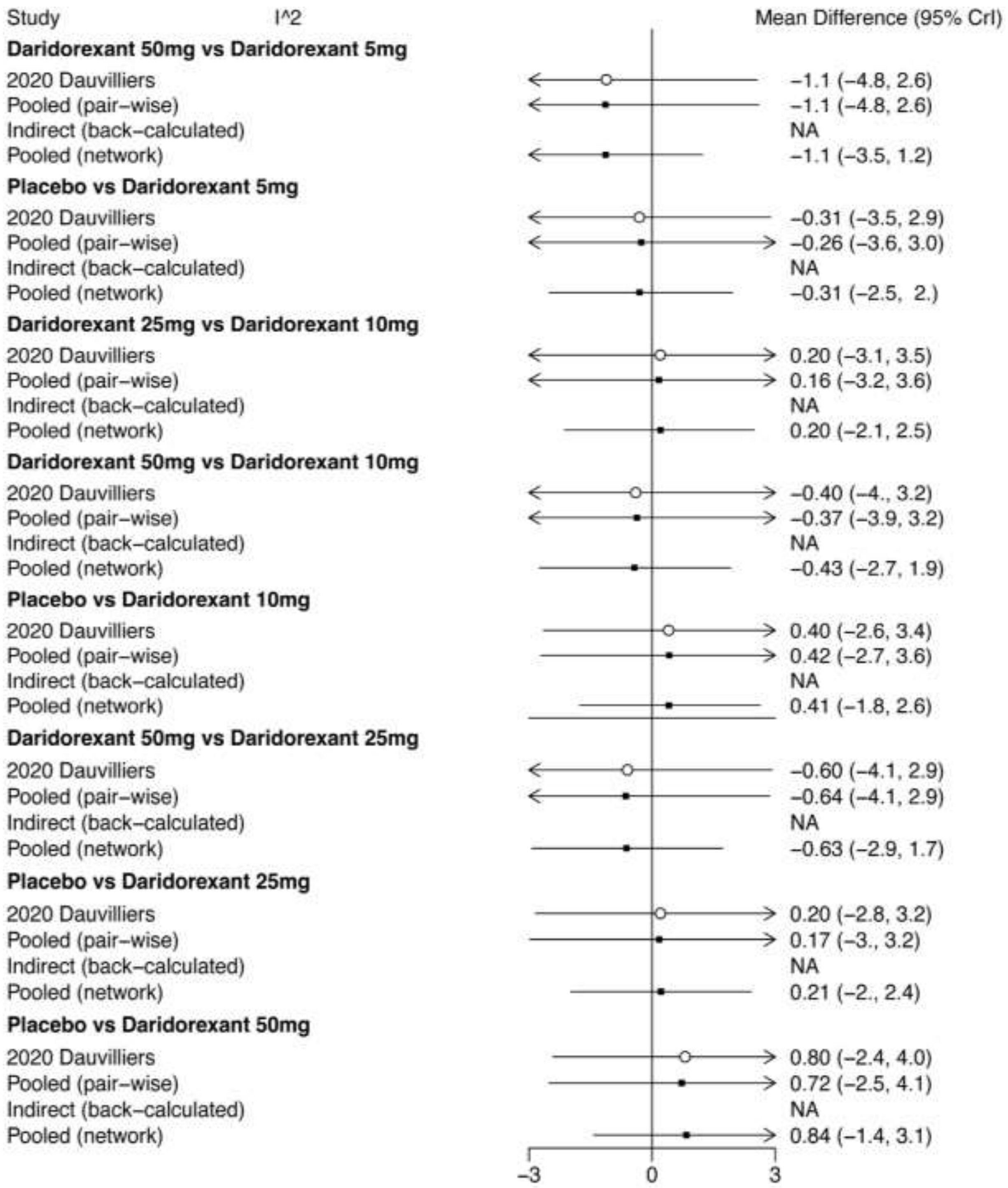
Study
I^2
Mean Difference (95% CrI)
Daridorexant 50mg vs Daridorexant 5mg
2020 Dauvilliers
Pooled (pair–wise)
Indirect (back–calculated)
Pooled (network)
−1.1 (−4.8, 2.6)
−1.1 (−4.8, 2.6)
NA
−1.1 (−3.5, 1.2)
Placebo vs Daridorexant 5mg
2020 Dauvilliers
Pooled (pair–wise)
Indirect (back–calculated)
Pooled (network)
−0.31 (−3.5, 2.9)
−0.26 (−3.6, 3.0)
NA
−0.31 (−2.5, 2.)
Daridorexant 25mg vs Daridorexant 10mg
2020 Dauvilliers
Pooled (pair–wise)
Indirect (back–calculated)
Pooled (network)
0.20 (−3.1, 3.5)
0.16 (−3.2, 3.6)
NA
0.20 (−2.1, 2.5)
Daridorexant 50mg vs Daridorexant 10mg
2020 Dauvilliers
Pooled (pair–wise)
Indirect (back–calculated)
Pooled (network)
−0.40 (−4., 3.2)
−0.37 (−3.9, 3.2)
NA
−0.43 (−2.7, 1.9)
Placebo vs Daridorexant 10mg
2020 Dauvilliers
Pooled (pair–wise)
Indirect (back–calculated)
Pooled (network)
0.40 (−2.6, 3.4)
0.42 (−2.7, 3.6)
NA
0.41 (−1.8, 2.6)
Daridorexant 50mg vs Daridorexant 25mg
2020 Dauvilliers
Pooled (pair–wise)
Indirect (back–calculated)
Pooled (network)
−0.60 (−4.1, 2.9)
−0.64 (−4.1, 2.9)
NA
−0.63 (−2.9, 1.7)
Placebo vs Daridorexant 25mg
2020 Dauvilliers
Pooled (pair–wise)
Indirect (back–calculated)
Pooled (network)
0.20 (−2.8, 3.2)
0.17 (−3., 3.2)
NA
0.21 (−2., 2.4)
Placebo vs Daridorexant 50mg
2020 Dauvilliers
Pooled (pair–wise)
Indirect (back–calculated)
Pooled (network)
0.80 (−2.4, 4.0)
0.72 (−2.5, 4.1)
NA
0.84 (−1.4, 3.1)
−3
0
3

eFigure 27: Forest plots for the heterogeneity: AEs.

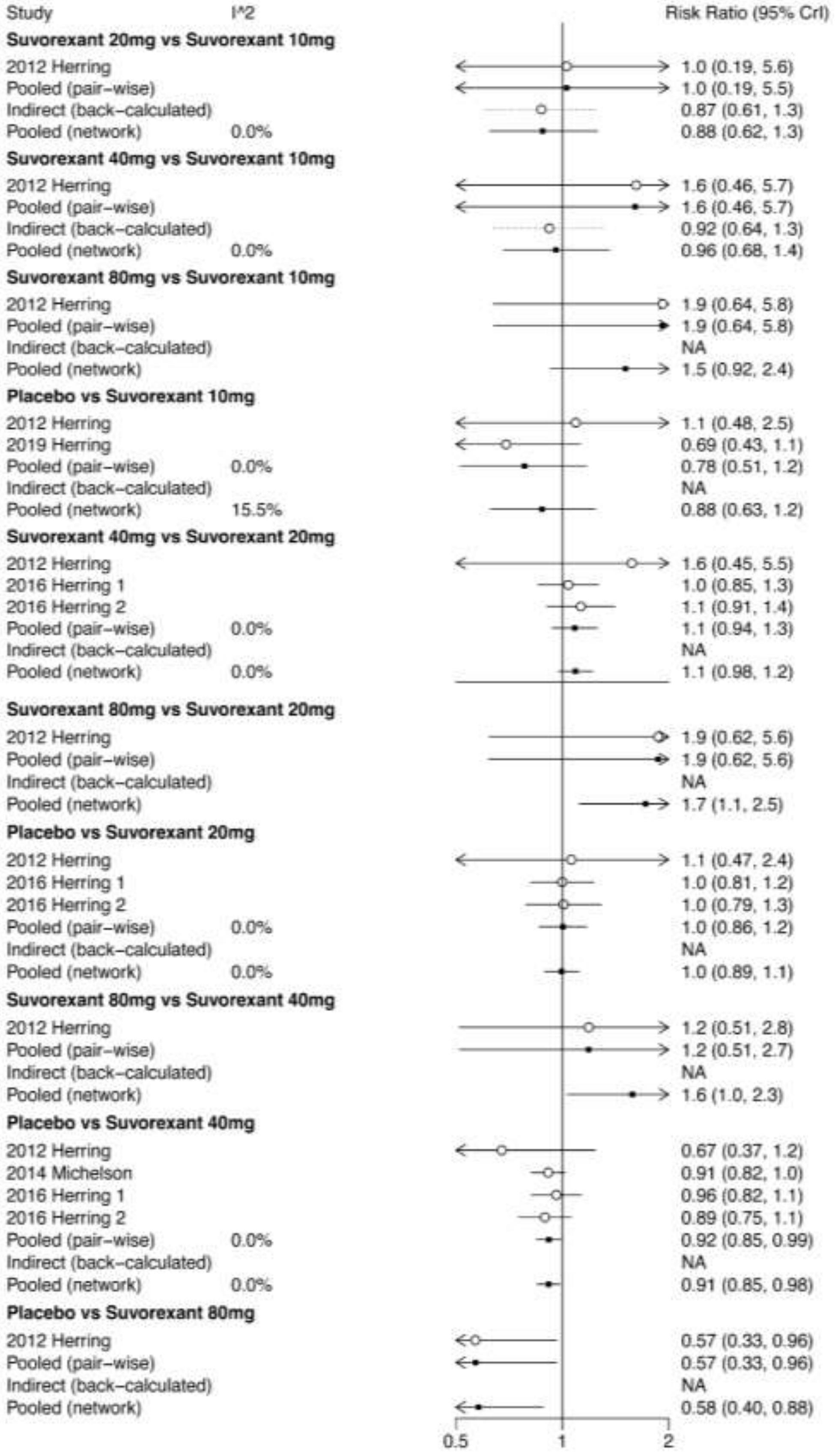

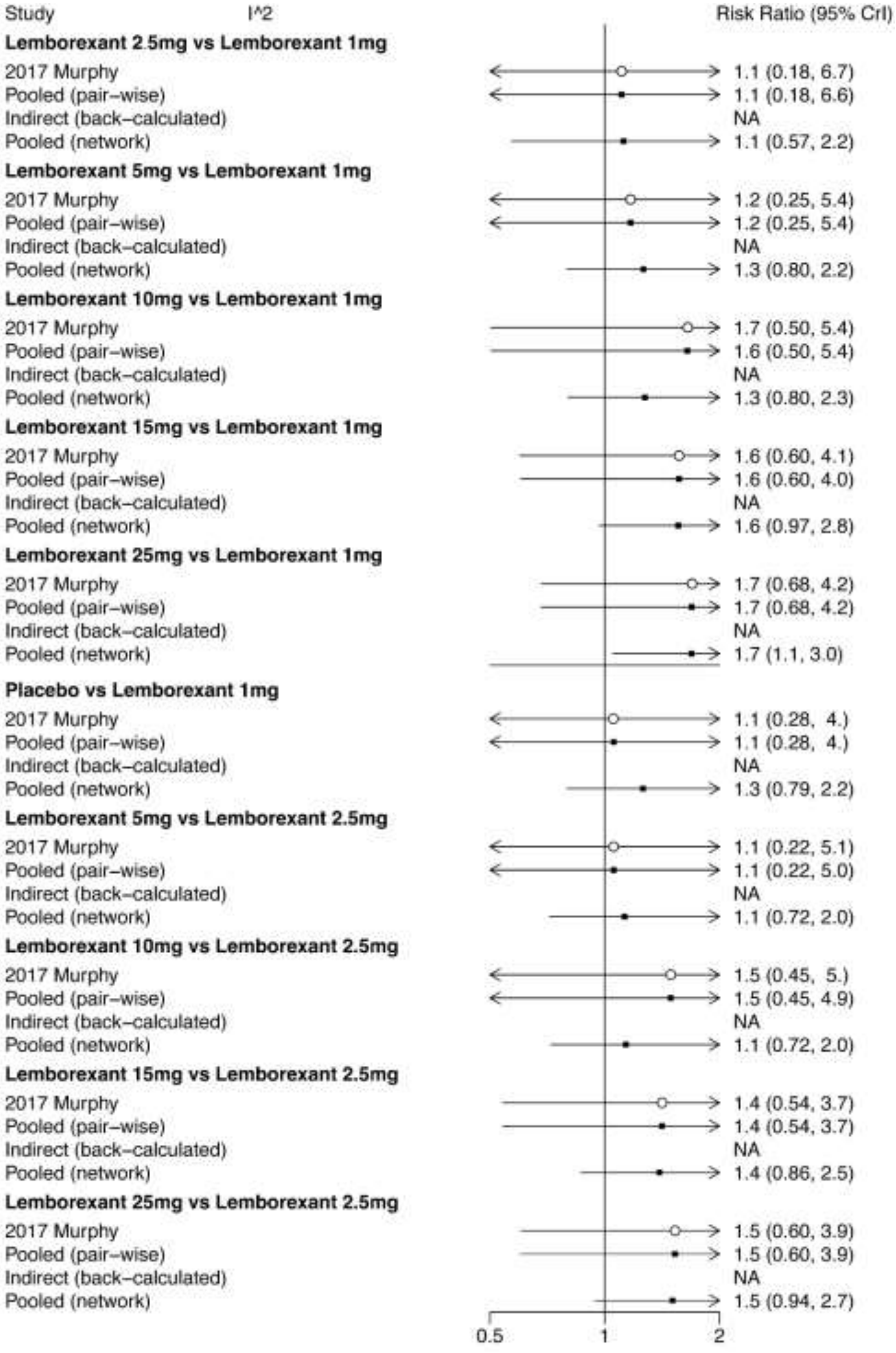
Study
I^2
Risk Ratio (95% CrI)
Lemborexant 2.5mg vs Lemborexant 1mg
2017 Murphy
1.1 (0.18, 6.7)
Pooled (pair–wise)
1.1 (0.18, 6.6)
Indirect (back–calculated)
NA
Pooled (network)
1.1 (0.57, 2.2)
Lemborexant 5mg vs Lemborexant 1mg
2017 Murphy
1.2 (0.25, 5.4)
Pooled (pair–wise)
1.2 (0.25, 5.4)
Indirect (back–calculated)
NA
Pooled (network)
1.3 (0.80, 2.2)
Lemborexant 10mg vs Lemborexant 1mg
2017 Murphy
1.7 (0.50, 5.4)
Pooled (pair–wise)
1.6 (0.50, 5.4)
Indirect (back–calculated)
NA
Pooled (network)
1.3 (0.80, 2.3)
Lemborexant 15mg vs Lemborexant 1mg
2017 Murphy
1.6 (0.60, 4.1)
Pooled (pair–wise)
1.6 (0.60, 4.0)
Indirect (back–calculated)
NA
Pooled (network)
1.6 (0.97, 2.8)
Lemborexant 25mg vs Lemborexant 1mg
2017 Murphy
1.7 (0.68, 4.2)
Pooled (pair–wise)
1.7 (0.68, 4.2)
Indirect (back–calculated)
NA
Pooled (network)
1.7 (1.1, 3.0)
Placebo vs Lemborexant 1mg
2017 Murphy
1.1 (0.28, 4.)
Pooled (pair–wise)
1.1 (0.28, 4.)
Indirect (back–calculated)
NA
Pooled (network)
1.3 (0.79, 2.2)
Lemborexant 5mg vs Lemborexant 2.5mg
2017 Murphy
1.1 (0.22, 5.1)
Pooled (pair–wise)
1.1 (0.22, 5.0)
Indirect (back–calculated)
NA
Pooled (network)
1.1 (0.72, 2.0)
Lemborexant 10mg vs Lemborexant 2.5mg
2017 Murphy
1.5 (0.45, 5.)
Pooled (pair–wise)
1.5 (0.45, 4.9)
Indirect (back–calculated)
NA
Pooled (network)
1.1 (0.72, 2.0)
Lemborexant 15mg vs Lemborexant 2.5mg
2017 Murphy
1.4 (0.54, 3.7)
Pooled (pair–wise)
1.4 (0.54, 3.7)
Indirect (back–calculated)
NA
Pooled (network)
1.4 (0.86, 2.5)
Lemborexant 25mg vs Lemborexant 2.5mg
2017 Murphy
1.5 (0.60, 3.9)
Pooled (pair–wise)
1.5 (0.60, 3.9)
Indirect (back–calculated)
NA
Pooled (network)
1.5 (0.94, 2.7)
0.5
1
2

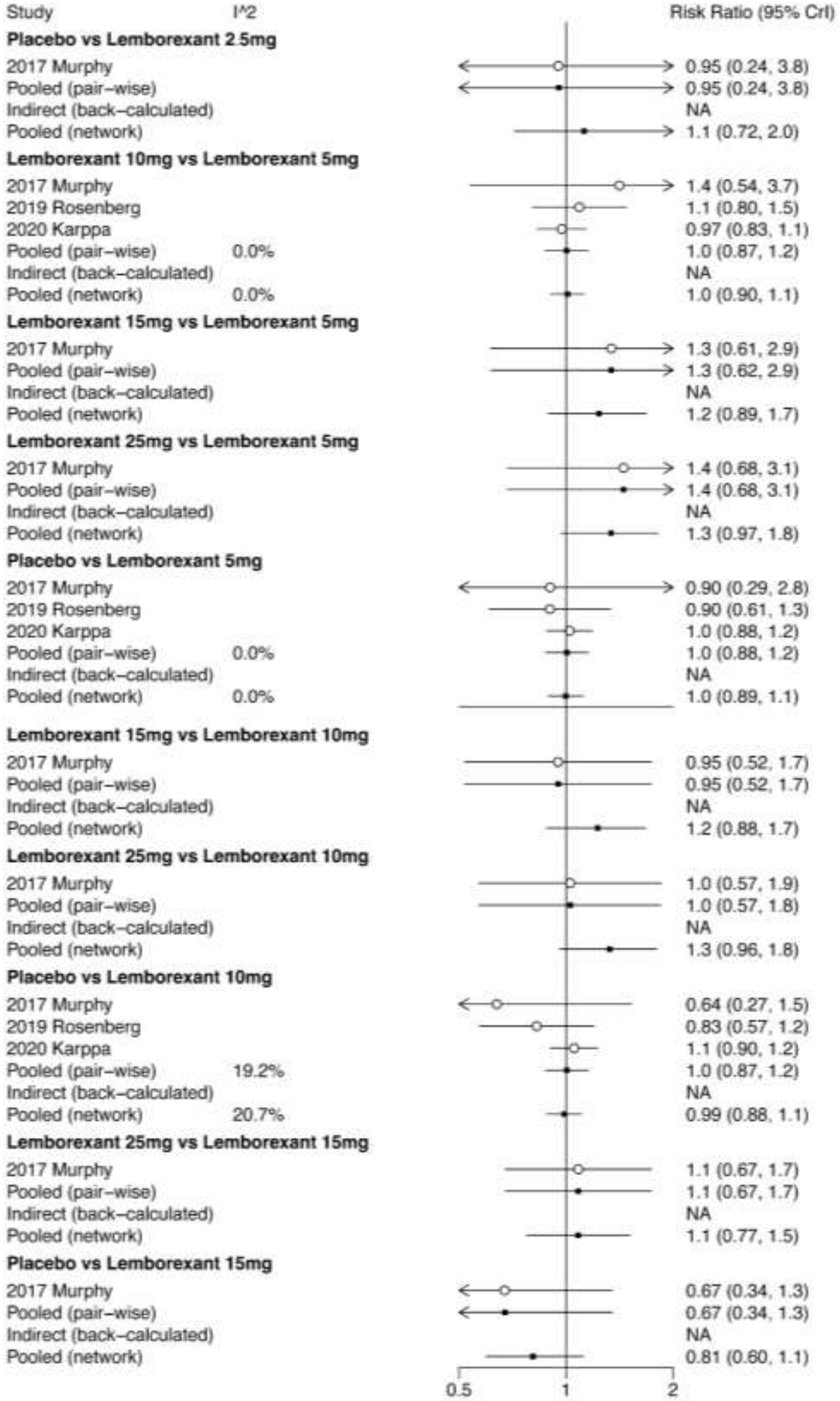

Study
I^2
Risk Ratio (95% CrI)
Placebo vs Lemborexant 2.5mg
2017 Murphy 0.95 (0.24, 3.8)
Pooled (pair−wise) 0.95 (0.24, 3.8)
Indirect (back−calculated) NA
Pooled (network) 1.1 (0.72, 2.0)
Lemborexant 10mg vs Lemborexant 5mg
2017 Murphy 1.4 (0.54, 3.7)
2019 Rosenberg 1.1 (0.80, 1.5)
2020 Karppa 0.97 (0.83, 1.1)
Pooled (pair−wise) 0.0% 1.0 (0.87, 1.2)
Indirect (back−calculated) NA
Pooled (network) 0.0% 1.0 (0.90, 1.1)
Lemborexant 15mg vs Lemborexant 5mg
2017 Murphy 1.3 (0.61, 2.9)
Pooled (pair−wise) 1.3 (0.62, 2.9)
Indirect (back−calculated) NA
Pooled (network) 1.2 (0.89, 1.7)
Lemborexant 25mg vs Lemborexant 5mg
2017 Murphy 1.4 (0.68, 3.1)
Pooled (pair−wise) 1.4 (0.68, 3.1)
Indirect (back−calculated) NA
Pooled (network) 1.3 (0.97, 1.8)
Placebo vs Lemborexant 5mg
2017 Murphy 0.90 (0.29, 2.8)
2019 Rosenberg 0.90 (0.61, 1.3)
2020 Karppa 1.0 (0.88, 1.2)
Pooled (pair−wise) 0.0% 1.0 (0.88, 1.2)
Indirect (back−calculated) NA
Pooled (network) 0.0% 1.0 (0.89, 1.1)
Lemborexant 15mg vs Lemborexant 10mg
2017 Murphy 0.95 (0.52, 1.7)
Pooled (pair−wise) 0.95 (0.52, 1.7)
Indirect (back−calculated) NA
Pooled (network) 1.2 (0.88, 1.7)
Lemborexant 25mg vs Lemborexant 10mg
2017 Murphy 1.0 (0.57, 1.9)
Pooled (pair−wise) 1.0 (0.57, 1.8)
Indirect (back−calculated) NA
Pooled (network) 1.3 (0.96, 1.8)
Placebo vs Lemborexant 10mg
2017 Murphy 0.64 (0.27, 1.5)
2019 Rosenberg 0.83 (0.57, 1.2)
2020 Karppa 1.1 (0.90, 1.2)
Pooled (pair−wise) 19.2% 1.0 (0.87, 1.2)
Indirect (back−calculated) NA
Pooled (network) 20.7% 0.99 (0.88, 1.1)
Lemborexant 25mg vs Lemborexant 15mg
2017 Murphy 1.1 (0.67, 1.7)
Pooled (pair−wise) 1.1 (0.67, 1.7)
Indirect (back−calculated) NA
Pooled (network) 1.1 (0.77, 1.5)
Placebo vs Lemborexant 15mg
2017 Murphy 0.67 (0.34, 1.3)
Pooled (pair−wise) 0.67 (0.34, 1.3)
Indirect (back−calculated) NA
Pooled (network) 0.81 (0.60, 1.1)
0.5
1
2

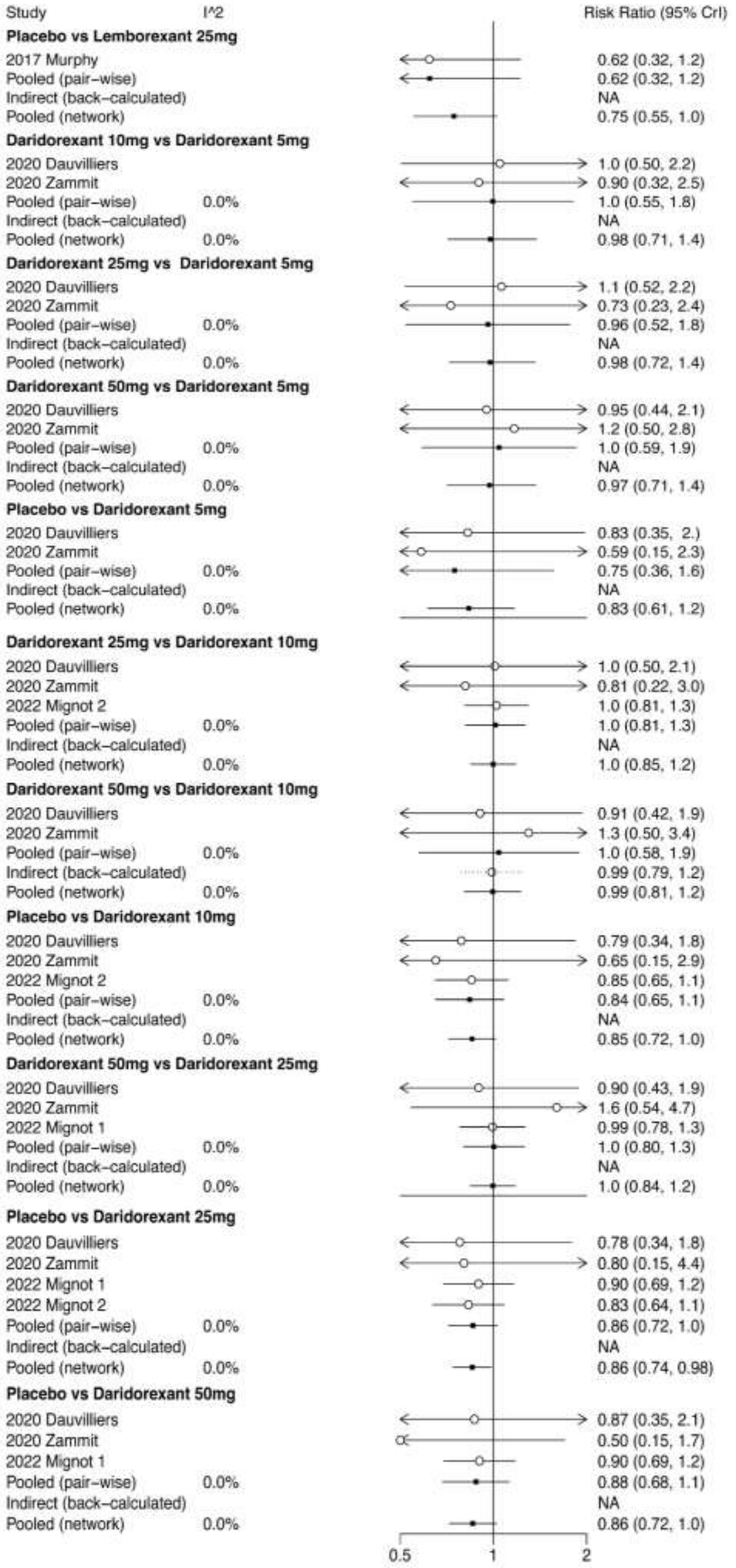

Study
I^2
Risk Ratio (95% CrI)
Placebo vs Lemborexant 25mg
2017 Murphy 0.62 (0.32, 1.2)
Pooled (pair–wise) 0.62 (0.32, 1.2)
Indirect (back–calculated) NA
Pooled (network) 0.75 (0.55, 1.0)
Daridorexant 10mg vs Daridorexant 5mg
2020 Dauvilliers 1.0 (0.50, 2.2)
2020 Zammit 0.90 (0.32, 2.5)
Pooled (pair–wise) 0.0% 1.0 (0.55, 1.8)
Indirect (back–calculated) NA
Pooled (network) 0.0% 0.98 (0.71, 1.4)
Daridorexant 25mg vs Daridorexant 5mg
2020 Dauvilliers 1.1 (0.52, 2.2)
2020 Zammit 0.73 (0.23, 2.4)
Pooled (pair–wise) 0.0% 0.96 (0.52, 1.8)
Indirect (back–calculated) NA
Pooled (network) 0.0% 0.98 (0.72, 1.4)
Daridorexant 50mg vs Daridorexant 5mg
2020 Dauvilliers 0.95 (0.44, 2.1)
2020 Zammit 1.2 (0.50, 2.8)
Pooled (pair–wise) 0.0% 1.0 (0.59, 1.9)
Indirect (back–calculated) NA
Pooled (network) 0.0% 0.97 (0.71, 1.4)
Placebo vs Daridorexant 5mg
2020 Dauvilliers 0.83 (0.35, 2.)
2020 Zammit 0.59 (0.15, 2.3)
Pooled (pair–wise) 0.0% 0.75 (0.36, 1.6)
Indirect (back–calculated) NA
Pooled (network) 0.0% 0.83 (0.61, 1.2)
Daridorexant 25mg vs Daridorexant 10mg
2020 Dauvilliers 1.0 (0.50, 2.1)
2020 Zammit 0.81 (0.22, 3.0)
2022 Mignot 2 1.0 (0.81, 1.3)
Pooled (pair–wise) 0.0% 1.0 (0.81, 1.3)
Indirect (back–calculated) NA
Pooled (network) 0.0% 1.0 (0.85, 1.2)
Daridorexant 50mg vs Daridorexant 10mg
2020 Dauvilliers 0.91 (0.42, 1.9)
2020 Zammit 1.3 (0.50, 3.4)
Pooled (pair–wise) 0.0% 1.0 (0.58, 1.9)
Indirect (back–calculated) 0.99 (0.79, 1.2)
Pooled (network) 0.0% 0.99 (0.81, 1.2)
Placebo vs Daridorexant 10mg
2020 Dauvilliers 0.79 (0.34, 1.8)
2020 Zammit 0.65 (0.15, 2.9)
2022 Mignot 2 0.85 (0.65, 1.1)
Pooled (pair–wise) 0.0% 0.84 (0.65, 1.1)
Indirect (back–calculated) NA
Pooled (network) 0.0% 0.85 (0.72, 1.0)
Daridorexant 50mg vs Daridorexant 25mg
2020 Dauvilliers 0.90 (0.43, 1.9)
2020 Zammit 1.6 (0.54, 4.7)
2022 Mignot 1 0.99 (0.78, 1.3)
Pooled (pair–wise) 0.0% 1.0 (0.80, 1.3)
Indirect (back–calculated) NA
Pooled (network) 0.0% 1.0 (0.84, 1.2)
Placebo vs Daridorexant 25mg
2020 Dauvilliers 0.78 (0.34, 1.8)
2020 Zammit 0.80 (0.15, 4.4)
2022 Mignot 1 0.90 (0.69, 1.2)
2022 Mignot 2 0.83 (0.64, 1.1)
Pooled (pair–wise) 0.0% 0.86 (0.72, 1.0)
Indirect (back–calculated) NA
Pooled (network) 0.0% 0.86 (0.74, 0.98)
Placebo vs Daridorexant 50mg
2020 Dauvilliers 0.87 (0.35, 2.1)
2020 Zammit 0.50 (0.15, 1.7)
2022 Mignot 1 0.90 (0.69, 1.2)
Pooled (pair–wise) 0.0% 0.88 (0.68, 1.1)
Indirect (back–calculated) NA
Pooled (network) 0.0% 0.86 (0.72, 1.0)
0.5
1
2

eFigure 28: Forest plots for the inconsistency: LPS.

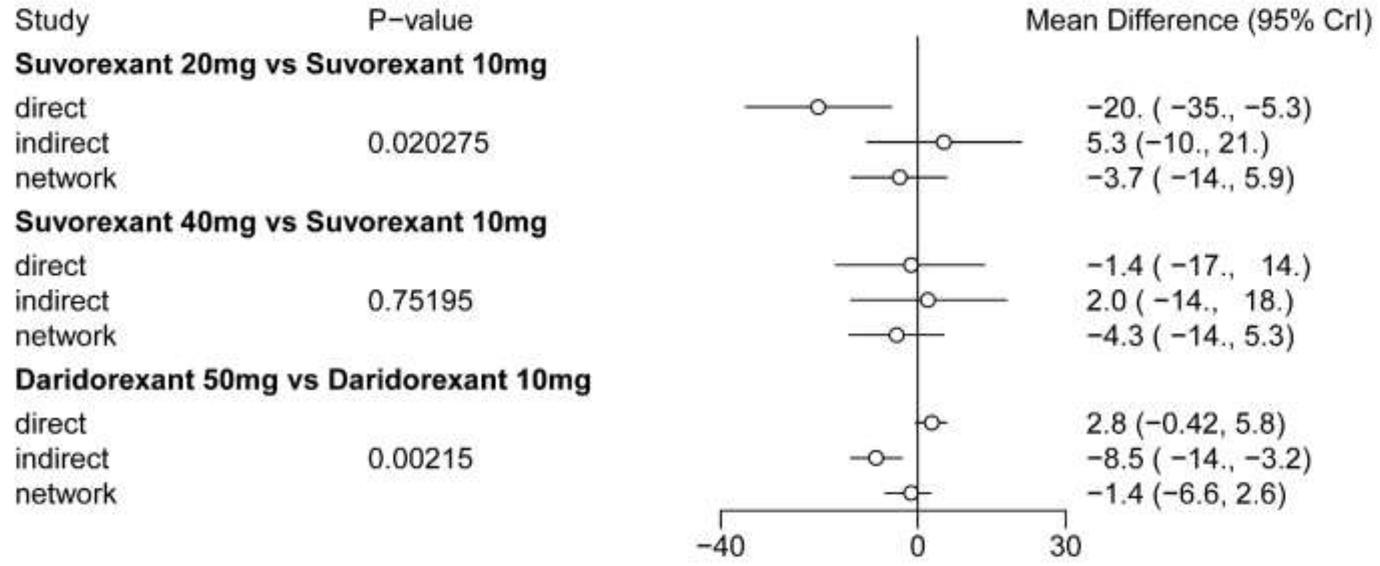

eFigure 29: Forest plots for the inconsistency: WASO.

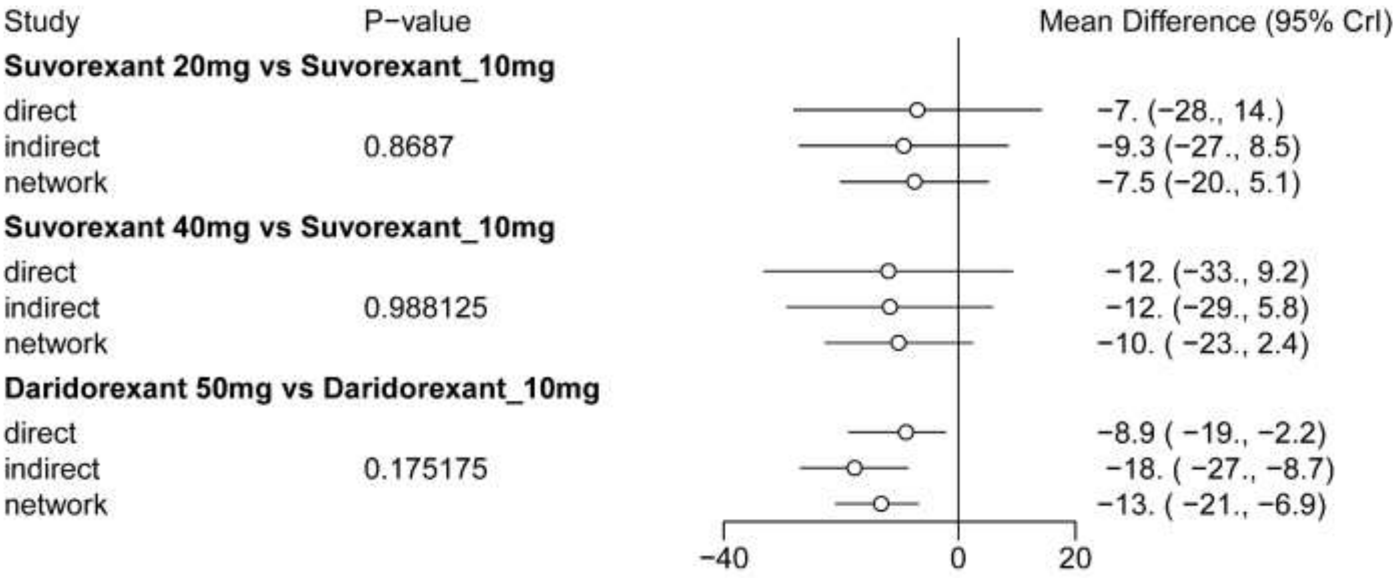

eFigure 30: Forest plots for the inconsistency: sTST.

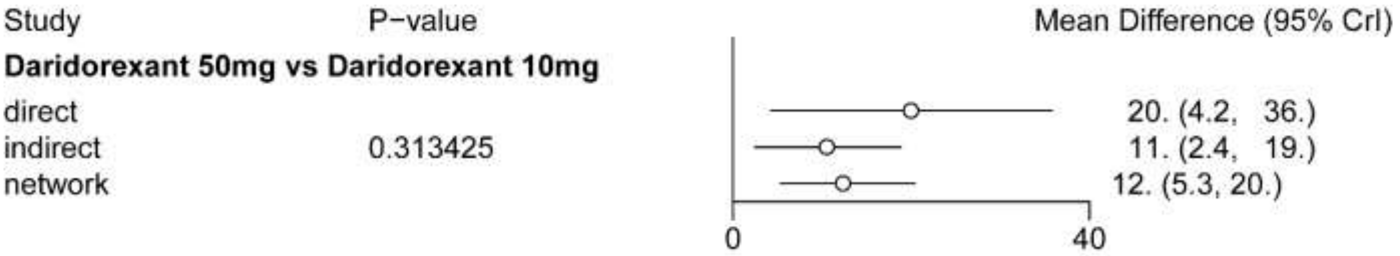

eFigure 31: Forest plots for the inconsistency: AEs.

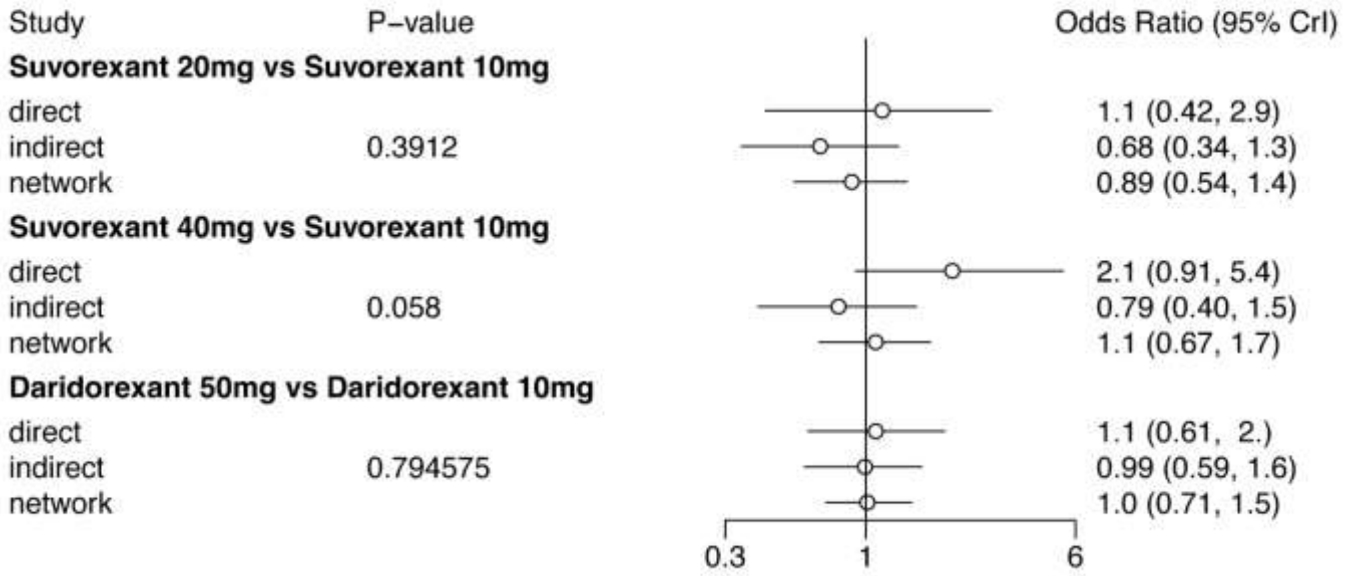

eFigure 32: Forest plots for the inconsistency: SAEs.

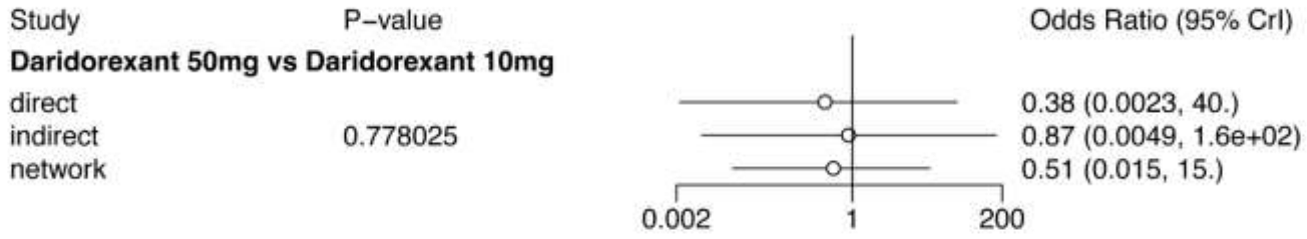

Supplement: Supplementary file 1 [file DataSheet1.PDF]
